# DNA Metabarcoding for Quality Control of Basil, Oregano, and Paprika

**DOI:** 10.3389/fpls.2021.665618

**Published:** 2021-06-04

**Authors:** Ancuţa Cristina Raclariu-Manolică, Jarl Andreas Anmarkrud, Marcin Kierczak, Nima Rafati, Birgitte Lisbeth Graae Thorbek, Audun Schrøder-Nielsen, Hugo J. de Boer

**Affiliations:** ^1^Natural History Museum, University of Oslo, Oslo, Norway; ^2^Stejarul Research Centre for Biological Sciences, National Institute of Research and Development for Biological Sciences, Piatra Neamt, Romania; ^3^Researgene, Uppsala, Sweden

**Keywords:** authentication, DNA metabarcoding, food control, food safety, high-throughput sequencing, molecular identification

## Abstract

Herbs and spices are some of the most vulnerable products in terms of fraud and adulteration in the food sector. Although standard analytical methods are accurate for quality control of specific lead or marker compounds, they cannot accurately assess the entire species composition of many marketed products. Complementary analytical approaches are thus often used for comprehensive screening of herbs and spices. In this study we evaluate DNA metabarcoding for the identification and authentication of 62 products, containing basil, oregano, and paprika collected from different retailers and importers in Norway. Our results show varying degrees of discrepancy between the constituent species and those listed on the product labels, despite high product authenticity. We suggest the false positives result from the sensitivity of DNA metabarcoding and filtering thresholds should be integrated into protocols to reduce false positives. Our results highlight how integrating DNA metabarcoding into the toolbox of analytical methods for quality control of fresh and/or processed plant-based food can improve product quality.

## Introduction

Herbs and spices have been used for food and beverage flavoring since the beginning of human history. Herbs generally refer to the leafy parts of a plant, and spices are derived from any other plant part, including the root, stem, bulb, bark or seeds. Herbs and spices may derive from the same plant and they are usually rich sources of phytochemicals. Some herbs and spices may be categorized as functional foods or nutraceuticals with health properties beyond basic nutrition, such as a reduction of the risks related to cardiovascular disease, diabetes, obesity, cancer, and Alzheimer’s disease (Tapsell et al., 2006; Viuda-Martos et al., 2011).

Globalization and the search for curative properties are key drivers behind the notable growth in both the value and volume of trade in herbs and spices (Amcham and Trade USA, 2015). The global market of these commodities is projected to grow in the near future to US$6.5 billion, with Asia and Europe the largest consumer markets worldwide (CBI, 2018).

Herbs and spices products are however some of the most vulnerable segments within the food sector (Galvin-King et al., 2018; van Ruth et al., 2018). Apart from their popularity and long historical use, there are increasing concerns over the product quality and safety (Galvin-King et al., 2018). There are several recent cases of adulteration involving the deliberate inclusion of substances whose presence is not legally declared and leaving out expensive ingredients (Galvin-King et al., 2018). Additionally food safety concerns can arise from issues related to chemical, microbial and physical hazards. Another major issue is the adulteration of herbs and spices. This is driven by the increasing level of consumption which exceeds the supply capacity as well as price increases, especially when complex supply chains are involved (Black et al., 2016; Galvin-King et al., 2018).

Herbs and spices as high-price commodities are among some of the most reported products involved in food fraud (Silvis et al., 2017; Galvin-King et al., 2018). The effects of a food fraud scandal can be detrimental for a business operator and result in varying degrees of prejudice and public distrust toward the industry sector in which it occurs (Aung and Chang, 2014). Some herbs and spices are especially well documented to be susceptible to substitution with unauthorized synthetic flavor and colors, including the use of the Sudan Red G dye, which is illegal and comes with genotoxic and carcinogenic effects, to color chili, curry and curcuma (Cornet et al., 2006; Galvin-King et al., 2020).

Generally a fast, reliable and comprehensive analytical approach is required to detect the authenticity and integrity of food items. Also, efficient traceability tools are necessary to minimize the production and distribution of unsafe or poor quality products in order to protect the consumer’s health and confidence (Aung and Chang, 2014). However, many important issues influence the quality of the herbs and spices. These need to be carefully considered when deciding on the analytical method for quality control. Herbs and spices are often marketed as complex mixtures containing one or more species that have been through many processing steps. The resulting phytochemical diversity across these products usually complicates further investigations, posing serious challenges to the accuracy of the quality control. Thus, modern research in food science requires innovative approaches using advanced analytical tools for comprehensive screening and analysis to detect and prevent fraud in the industry (Galvin-King et al., 2018).

With DNA metabarcoding, or multispecies identification with extracellular and/or total DNA extracted from complex samples containing DNA of different origins (Taberlet et al., 2012) followed by high throughput sequencing (HTS), it is possible to analyze species composition in mixtures of DNA in a range of products. This includes environmental samples (Sogin et al., 2006; Epp et al., 2012; Nielsen and Wall, 2013) or marketed herbal medicines (Coghlan et al., 2015; Ivanova et al., 2016; Raclariu et al., 2017a, b, 2018b; Anthoons et al., 2020). DNA metabarcoding is a highly sensitive method, and *a priori* and *a posteriori* data can inform and aid interpretation of metabarcoding results. *A priori* and *a posteriori* data can help to classify positive identifications that are relevant to consumer safety, as well as contaminants that are not likely to be present in significant quantities. It will also help to interpret false negative detections and make assumptions on the possible cause of the overlooked species.

In this study we develop and test DNA metabarcoding for identifying plant species in marketed herbs and spices. We targeted two herbs, oregano *(Origanum vulgare* L., Lamiaceae), and basil (*Ocimum basilicum* L., Lamiaceae) and one spice, paprika (*Capsicum annuum* L., Solanaceae), purchased in Norway. These target plant species were selected due to their popularity and leading consumer preference. Despite the fact that Europe is a large producer of native herbs, such as basil and oregano, these are still imported in large quantities as European production fails to meet the growing market demand (CBI, 2018).

Our hypothesis was that DNA metabarcoding can detect plant ingredients in herbs and spices-based marketed products, but that blind use of these molecular identification methods can result in an underestimation of product quality. Specific research objectives were to (i) evaluate DNA metabarcoding for detecting substitution in marketed herbs and spices; (ii) assess the use of *a priori* and *a posteriori* data to enhance interpretation of DNA metabarcoding data; (iii) contribute to developing a complementary tool to secure the food supply chain against accidental substitution and/or deliberate adulteration.

## Materials and Methods

### Samples Collection and Preparation

62 herb and spice samples were examined that included 23 samples of oregano, 21 of paprika, 17 of basil, and one mix of oregano and basil. Information on the samples including label information, but not the producer/importer name, lot number, expiration date, or any other information that could lead to the identification of that specific product can be found in Supplementary File 1: Information about the investigated herbs and spices products.

### DNA Extraction

A 200 mg (dry weight) of each sample was transferred to a 2 ml microcentrifuge tube with two 3 mm tungsten-carbide beads (Qiagen) and ground to a fine powder on a Retsch mill MM 301 (Retsch GmbH, Haan Germany) (2X cycles for 45 s at 25 Hz). Total DNA was extracted from the homogenized contents using a modified CTAB extraction method as described by Doyle and Doyle (1990), and adapted by Raclariu et al. (2017b). The final elution volume was 100 μl.

### DNA Libraries Preparation and Sequencing

The amplicon library and high-throughput sequencing were performed as previously described (Raclariu et al., 2017b) with slight modifications. All amplicon libraries were prepared in three technical replicates on 96-well PCR amplification plates. On each plate we also included negative controls (also in triplicate), including extraction blanks (extraction negative controls, ENC) and PCR controls (PCR negative controls, PNC). The amplicon libraries were prepared using fusion primers for the nuclear ribosomal target sequences, internal transcribed spacer nrITS2. Ion Torrent fusion primers were based on 5.8I2 and 26SE as described by Sun et al. (1994), ITS2_5.8I_F 5′-GCCTGGGCGTCACGC and_26SE_R 5′-CCCGGTTCGCTCGCCGTTAC. The primer match/mismatch was checked before the wet lab work for the target species. The forward primer was labeled with a unique 10 bp multiplex identifier (MID) tag and the reverse primer with a uniform truncated P1 (trP1) tag. Thermal cycling was carried out in 25 μl final reaction volumes, and each reaction contained 1X Q5 hot start high fidelity mastermix (New England Biolabs Inc., United Kingdom), 0.5 μM of each primer (Biolegio, Netherlands), and 1 μl of template DNA. The following thermocycling protocol was used: 30 s of initial denaturation at 98°C, followed by 30 cycles of denaturation at 98°C for 10 s, annealing at 71°C for 30 s, and elongation at 72°C for 30 s, followed by a final elongation step at 72°C for 2 min. Agarose gel electrophoresis was used to inspect amplification of all PCR reactions. Based on the relative intensity of the PCR fragments on the gels, we merged uniform amounts of each amplicon in a separate microcentrifuge tube using a Biomek 4000 Laboratory Automation Workstation (Beckman Coulter, United States) liquid handling system. Each normalized library was size selected using BluePippin (Sage Science, United States) and BDF1510 cassettes, targeting the desired fragment length within the range of 300-550 bp. The library was then purified with 0.8X AMpure XP beads (Beckman Coulter, United States) following the manufacturer’s instructions. The total concentration of the purified pooled amplicon library stock was measured on both Qubit (Invitrogen, United States) with DNA High Sensitivity Kit (Thermo Fischer Scientific, United States) and on Fragment Analyzer^TM^ (Advanced Analytical Technologies, Inc., United States) using the DNF-488 High Sensitivity Genomic DNA Analysis Kit, in order to identify the optimum concentration range for the template preparation. An Ion Chef (Life Technologies (LT), Thermo Fisher Scientific, United States) was used to prepare pooled Ion AmpliSeq libraries (LT) for emulsion PCR and to load the sequencing chips. The sequencing was performed on Ion 318 v2 chips and sequenced on an Ion Torrent PGM (LT). Sequencing data was analyzed and demultiplexed into FASTQ files per sample using Torrent Suite version 5.0.4 (LT).

### Bioinformatics Data Analysis

MultiQC (Ewels et al., 2016) was used to perform quality control of the raw FASTQ files delivered by the sequencing facility. Using the Flexbar tool the reads were demultiplexed first by library barcode and then by the nrITS2 primer sequences used for amplification (Dodt et al., 2012; Roehr et al., 2017). The following selection criteria were used to select the remaining reads: minimal read length of 200 bp, base-quality threshold set to 22, and alignment mode LEFT leaving the right side of the sequence after primer removal. We set the barcode error rate to 2 mismatches per 10 nucleotides. Cutadapt was used to trim the reads to a maximum of 360 bp and merge the reads that were amplified by both the forward and reverse primers into a single file (Martin, 2011). Dada2 was used for the following analysis (Callahan et al., 2016, 2017) taking the amplicon sequence variants (ASV) approach as described in Callahan et al. (2017). The 1.6.0 version of the dada2 R package and R 3.4.3 was used (Kite-eating Tree). In the initial step of the ASV pipeline, we estimated read quality using 5 randomly (seed 77) selected samples (products). After estimating the read quality, we trimmed the 15 left most nucleotides and removed any truncated reads if the base quality dropped to 2. Reads beyond 320bp were discarded. The truncated reads were used to train for base-call error pattern in our dataset. The learned patterns were then used to correct the trimmed and dereplicated reads. Following this, sample inference was performed using minimal Hamming distance within a single cluster set to 3. We set the minimal number of reads to form a cluster to 5. From the resulting samples a set S of the most abundant sequences was created. Every sequence was checked for whether it was a chimera of any two sequences from S, and all chimeric sequences were removed. The species identification was performed by aligning (BLAST) all inferred amplicon sequence variants to the ITS2db (Ankenbrand et al., 2015) database (ver. V, access: June 03, 2018) of ITS2 sequences for green plants (Viridiplantae), using 97% sequence identity threshold. For each ASV, we report the top 4 BLAST hits.

## Results

The success rate in identifying ASVs varied between the samples (see Supplementary File 3: Species identification report and BLAST queries). 8 out of 62 samples (KR18-09, KR18-16, KR18-33, KR18-46, KR18-52, KR18-53, KR18-54 and KR18-60) did not pass our quality and trimming quality criteria, and they yielded no taxonomic identifications after the analysis. The extraction negative controls (ENC) and the PCR negative controls (PNC) did not yield any taxonomic identification.

### Paprika

**FIGURE 1:**
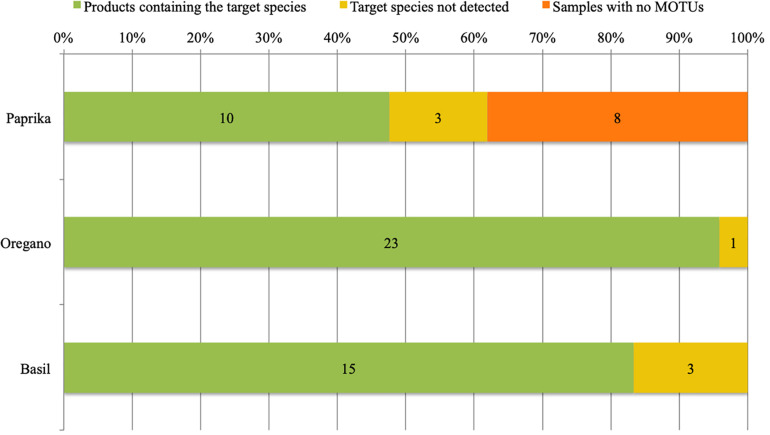
Product fidelity rates per target species using DNA metabarcoding based identification.

13 out of 21 samples with paprika listed as the only ingredient listed on the label yielded taxonomic identifications (See Figure 1, Table 1, and Supplementary File 4: DNA metabarcoding based identification of the products). The low success rate of DNA metabarcoding for paprika identification could be the result of DNA degradation due to excessive heating in the drying and/or smoking process of the peppers. The eight non-identified samples were excluded from further analyses. Of the identified 13 total samples, ten included and three lacked paprika (KR18-07; KR18-23; and KR18-58). However, the samples lacking paprika did include DNA from wind-pollinated plant species (KR18-07; KR18-23) or DNA from wind-spread plant species (KR18-58). Five samples yielded identifications of one to several wind-pollinated species. Three samples yielded identifications for one to several wind-spread species. Three samples yielded identifications for other plant species than those listed on the product label, or reasonably expected through contamination from wind-pollinated or wind-spread species. The detection of *Zea mays* in KR18-03 suggested the presence of corn flour, a common gluten-free filler found in several DNA barcoding studies of herbal medicines.

**TABLE 1:** DNA metabarcoding based authentication of the products labeled as containing paprika.

Sample ID	Paprika	Unlabeled ingredients		
	# of replicates detected	Plant species	# of replicates detected	ASV identifier
**KR18-03**	2	*Zea mays*	3	PTU_ 034
**KR18-04**	3	*–*	–	–
**KR18-07**	0	*Phragmites australis*	2	PTU_305
		*Phragmites japonicus*	2	PTU_305
**KR18-13**	2	*Molinia caerulea*	2	PTU_354
		*Phragmites australis*	2	PTU_354
		*Phragmites japonicus*	2	PTU_354
**KR18-17**	2	*–*	–	–
**KR18-19**	2	*–*	–	–
**KR18-23**	0	*Amaranthus blitoides*	3	PTU_466
		*Amaranthus capensis*	3	PTU_466
		*Amaranthus*	3	PTU_466
		*tuberculatus*		
**KR18-26**	2	*Echinochloa colona*	2	PTU_072
		*Echinochloa crus-galli*	2	PTU_072
**KR18-28**	2	*Galinsoga parviflora*	3	PTU_209
		*Galinsoga quadriradiata*	3	PTU_209
		*Sabazia sarmentosa*	3	PTU_209
**KR18-34**	2	*Convolvulus arvensis*	3	PTU_010
		*Galinsoga parviflora*	3	PTU_209
		*Galinsoga quadriradiata*	3	PTU_209
		*Sabazia sarmentosa*	3	PTU_209
		*Solanum lycopersicum*	3	PTU_115
		*Solanum pennellii*	3	PTU_115
**KR18-38**	2	*Origanum vulgare*	3	PTU_036
**KR18-45**	3	*–*	–	–
**KR18-58**	0	*Galinsoga parviflora*	2	PTU_209
		*Galinsoga quadriradiata*	2	PTU_209
		*Sabazia sarmentosa*	2	PTU_209

### Oregano

All 23 samples of oregano, and the single sample containing a mixture of oregano and basil, could be identified based upon the obtained sequencing results (See Figure 1, Table 2, and Supplementary File 4: DNA metabarcoding based identification of the products). In only a single sample (KR18-01) the target *Origanum vulgare* was not detected. However, the identification of *Thymus* sp. in this product does raise concerns about its quality. 10 samples yielded identifications for one to several wind-pollinated species. 15 samples yielded identification for one to several wind-spread species. 19 samples yielded identifications for other plant species than those listed on the product label, or reasonably expected through contamination from wind-pollinated or wind-spread species. Notable identifications include *Thymus* sp. (KR18-01), *Mentha longifolia* (KR18-44), *Thymus* sp. and *Polygonum* sp. (KR18-47), and *Veronica* sp. (KR18-62). The identification of unexpected species in these samples suggests contamination in the production chain, and likely during harvesting (KR18-44; KR18-47; KR18-62), or processing (*Thymus* sp. in KR18-01 and KR18-47).

**TABLE 2:** DNA metabarcoding based authentication of the products labeled as containing oregano.

Sample ID	Oregano	Unlabeled ingredients		
	# of replicates detected	Plant species	# of replicates detected	ASV identifier
**KR18-01**	0	*Thymus altaicus*	3	PTU_265
		*Thymus marschallianus*	3	PTU_265
		*Thymus mongolicus*	3	PTU_265
		*Thymus pulegioides*	3	PTU_265
**KR18-05**	3	*Arnebia euchroma*	3	PTU_159
		*Tragopogon porrifolius*	3	PTU_159
		*Tragopogon pratensis*	3	PTU_159
		*Convolvulus arvensis*	3	PTU_166
		*Iris ruthenica*	3	PTU_432
		*Lactuca saligna*	3	PTU_432
		*Lactuca sativa*	3	PTU_432
		*Silene vulgaris*	3	PTU_496
		*Vaccaria hispanica*	3	PTU_496
		*Linaria genistifolia*	2	PTU_552
		*Linaria michauxii*	2	PTU_552
		*Linaria salangensis*	2	PTU_552
		*Linaria vulgaris*	2	PTU_552
		*Silene dichotoma*	2	PTU_284
		*Silene fabaria*	2	PTU_284
**KR18-06**	3	*Crepis foetida*	3	PTU_155
		*Linaria genistifolia*	3	PTU_252
		*Linaria japonica*	3	PTU_252
		*Linaria michauxii*	3	PTU_252
		*Linaria vulgaris*	3	PTU_252
		*Convolvulus arvensis*	2	PTU_257
		*Geranium rotundifolium*	2	PTU_616
		*Linaria salangensis*	2	PTU_361
**KR18-10**	3	*Convolvulus arvensis*	2	PTU_362
		*Iris ruthenica*	2	PTU_062
		*Lactuca saligna*	2	PTU_062
		*Lactuca sativa*	2	PTU_062
**KR18-12**	3	*Arnebia euchroma*	3	PTU_188
		*Convolvulus arvensis*	3	PTU_198
		*Crepis foetida*	3	PTU_155
		*Tragopogon porrifolius*	3	PTU_188
		*Tragopogon pratensis*	3	PTU_188
**KR18-14**	3	*Olea europaea*	3	PTU_293
**KR18-15**	3	*–*	–	–
**KR18-21**	3	*Convolvulus arvensis*	3	PTU_098
		*Conyza bonariensis*	3	PTU_110
		*Erigeron rosulatus*	3	PTU_110
		*Echinochloa colona*	3	PTU_123
		*Echinochloa crus-galli*	3	PTU_123
		*Echinochloa oryzicola*	3	PTU_123
		*Polygonum arenastrum*	3	PTU_160
**KR18-24**	3	*Convolvulus arvensis*	2	PTU_218
**KR18-27**	3	*Convolvulus arvensis*	3	PTU_267
		*Arnebia euchroma*	2	PTU_270
		*Phoebe puwenensis*	2	PTU_101
		*Tragopogon dubius*	2	PTU_210
		*Tragopogon pratensis*	2	PTU_210
**KR18-29**	3	*Bidens andicola*	3	PTU_228
		*Bidens aurea*	3	PTU_228
		*Bidens alba*	2	PTU_220
		*Bidens cronquistii*	2	PTU_220
		*Bidens pilosa*	2	PTU_220
**KR18-31**	2	*Convolvulus arvensis*	3	PTU_317
**KR18-35^**	3	*Convolvulus arvensis*	3	PTU_328
		*Echinochloa colona*	3	PTU_049
		*Echinochloa crus-galli*	3	PTU_049
		*Echinochloa oryzicola*	3	PTU_049
**KR18-36**	0	*Bidens andicola*	3	PTU_087
		*Bidens aurea*	3	PTU_087
		*Bromus erectus*	2	PTU_095
		*Bromus sterilis*	2	PTU_095
		*Bromus tectorum*	2	PTU_095
		*Taraxacum amplum*	2	PTU_322
		*Taraxacum officinale*	2	PTU_322
		*Taraxacum sp. CF-2016*	2	PTU_322
**KR18-41**	2	*Cirsium acaule*	3	PTU_538
		*Cirsium arvense*	3	PTU_538
		*Cirsium dissectum*	3	PTU_538
		*Cirsium tuberosum*	3	PTU_538
		*Convolvulus arvensis*	3	PTU_112
		*Melica ciliata*	2	PTU_286
		*Melica nutans*	2	PTU_286
		*Melica picta*	2	PTU_286
**KR18-42**	3	*Knautia degenii*	3	PTU_349
		*Knautia integrifolia*	3	PTU_349
**KR18-44**	3	*Amaranthus blitoides*	3	PTU_149
		*Amaranthus capensis*	3	PTU_149
		*Amaranthus tuberculatus*	3	PTU_149
		*Amaranthus powellii*	3	PTU_117
		*Amaranthus retroflexus*	3	PTU_117
		*Convolvulus arvensis*	3	PTU_407
		*Rhaponticum repens*	3	PTU_048
		*Ziziphora clinopodioides*	2	PTU_048
		*Arnebia euchroma*	2	PTU_130
		*Chenopodium album*	2	PTU_192
		*Iris ruthenica*	2	PTU_062
		*Lactuca saligna*	2	PTU_062
		*Lactuca sativa*	2	PTU_062
		*Mentha longifolia*	2	PTU_235
		*Tragopogon lainzii*	2	PTU_280
		*Tragopogon porrifolius*	2	PTU_291
		*Tragopogon pratensis*	2	PTU_291
**KR18-47**	3	*Convolvulus arvensis*	3	PTU_457
		*Polygonum arenastrum*	3	PTU_170
		*Polygonum aviculare*	3	PTU_170
		*Thymus altaicus*	3	PTU_265
		*Thymus marschallianus*	3	PTU_265
		*Thymus mongolicus*	3	PTU_265
		*Thymus pulegioides*	3	PTU_265
		*Polygonum rurivagum*	2	PTU_431
**KR18-48**	3	*Dactylis glomerata*	2	PTU_334
		*Petrorhagia nanteuilii*	2	PTU_499
**KR18-50**	3	*Bromus erectus*	3	PTU_365
		*Bromus sterilis*	3	PTU_365
		*Bromus tectorum*	3	PTU_365
		*Senecio vulgaris*	3	PTU_194
		*Erodium cicutarium*	2	PTU_510
		*Erodium lebelii*	2	PTU_510
		*Erodium moschatum*	2	PTU_510
**KR18-55**	3	*Filago pyramidata*	3	PTU_352
		*Gnaphalium supinum*	3	PTU_352
		*Logfia gallica*	3	PTU_352
		*Logfia minima*	3	PTU_352
**KR18-57**	3	*Amaranthus blitoides*	3	PTU_149
		*Amaranthus capensis*	3	PTU_149
		*Amaranthus tuberculatus*	3	PTU_149
		*Xanthium sibiricum*	3	PTU_223
		*Xanthium strumarium*	3	PTU_223
		*Arnebia euchroma*	2	PTU_231
		*Convolvulus arvensis*	2	PTU_461
		*Linaria genistifolia*	2	PTU_330
		*Linaria michauxii*	2	PTU_330
		*Linaria salangensis*	2	PTU_330
		*Linaria vulgaris*	2	PTU_330
		*Tragopogon porrifolius*	2	PTU_501
		*Tragopogon pratensis*	2	PTU_501
**KR18-61**	3	*Alopecurus myosuroides*	3	PTU_282
		*Poa trivialis*	3	PTU_282
		*Arbutus canariensis*	3	PTU_292
		*Arnebia euchroma*	3	PTU_319
		*Tragopogon porrifolius*	3	PTU_319
		*Tragopogon pratensis*	3	PTU_319
		*Convolvulus arvensis*	3	PTU_481
		*Iris ruthenica*	3	PTU_062
		*Lactuca saligna*	3	PTU_062
		*Lactuca sativa*	3	PTU_062
		*Scorzonera cana*	3	PTU_393
		*Scorzonera laciniata*	3	PTU_393
		*Salvia spinosa*	2	PTU_358
**KR18-62**	3	*Bromus erectus*	3	PTU_392
		*Bromus sterilis*	3	PTU_392
		*Bromus tectorum*	3	PTU_392
		*Veronica agrestis*	3	PTU_667
		*Veronica persica*	3	PTU_667
		*Bidens andicola*	2	PTU_232
		*Bidens aurea*	2	PTU_232

### Basil

All 17 samples of basil, and the one sample containing a mixture of oregano and basil, yielded DNA based identifications (See Figure 1, Table 3, and Supplementary File 4. DNA metabarcoding results for product identification). Three samples (KR18-32, KR18-51 and KR18-59) did not yield DNA identifications for *Ocimum basilicum*. Again, the identification of other plant species raised concerns about the quality of this product. 14 samples yielded identifications of one to several wind-pollinated species. One sample yielded identification of the wind-spread species. All 17 samples yielded identifications of other plant species than those listed on the product label, or reasonably expected through contamination from wind-pollinated or wind-spread species. Notable identifications include *Origanum vulgare* (KR18-25; KR18-30; KR18-51, KR18-59), *Physalis* sp. (KR18-25), *Ipomoea* sp. (KR18-43; KR18-49).

**TABLE 3:** DNA metabarcoding based authentication of the products labeled as containing basil.

Sample ID	Basil	Unlabeled ingredients		
	# of replicates detected	Plant species	# of replicates detected	ASV identifier
**KR18-02**	3	*Amaranthus hybridus*	3	PTU_083
		*Amaranthus palmeri*	3	PTU_083
		*Amaranthus retroflexus*	3	PTU_083
		*Damrongia orientalis*	3	PTU_253
**KR18-08**	2	*Amaranthus viridis*	3	PTU_023
		*Convolvulus arvensis*	3	PTU_595
		*Echinochloa colona*	3	PTU_409
		*Echinochloa frumentacea*	3	PTU_409
		*Hymenachne grumosa*	3	PTU_047
		*Portulaca oleracea*	3	PTU_051
		*Dactyloctenium aegyptium*	2	PTU_059
		*Damrongia orientalis*	2	PTU_059
**KR18-11**	3	*Amaranthus viridis*	3	PTU_147
		*Damrongia orientalis*	3	PTU_263
		*Amaranthus hybridus*	2	PTU_029
**KR18-18**	3	*Cynodon dactylon*	3	PTU_035
		*Dactyloctenium aegyptium*	3	PTU_187
		*Damrongia orientalis*	3	PTU_344
**KR18-20**	3	*Amaranthus viridis*	3	PTU_147
		*Damrongia orientalis*	3	PTU_015
		*Amaranthus hybridus*	2	PTU_027
**KR18-22**	3	*Damrongia orientalis*	3	PTU_447
**KR18-25**	0	*Amaranthus viridis*	3	PTU_023
		*Damrongia orientalis*	3	PTU_500
		*Echinochloa colona*	3	PTU_409
		*Echinochloa frumentacea*	3	PTU_409
		*Hymenachne grumosa*	3	PTU_061
		*Origanum vulgare*	3	PTU_057
		*Portulaca oleracea*	3	PTU_051
		*Physalis angulata*	2	PTU_276
		*Physalis pubescens*	2	PTU_276
**KR18-30**	3	*Amaranthus hybridus*	3	PTU_207
		*Convolvulus arvensis*	3	PTU_668
		*Damrongia orientalis*	3	PTU_662
		*Echinochloa colona*	3	PTU_409
		*Echinochloa frumentacea*	3	PTU_409
		*Hymenachne grumosa*	3	PTU_090
		*Amaranthus palmeri*	2	PTU_083
		*Amaranthus retroflexus*	2	PTU_083
		*Amaranthus viridis*	2	PTU_023
		*Origanum vulgare*	2	PTU_006
**KR18-32**	0	*Convolvulus arvensis*	3	PTU_668
		*Hymenachne grumosa*	3	PTU_249
		*Amaranthus viridis*	2	PTU_023
		*Damrongia orientalis*	2	PTU_447
**KR18-35^**	2	*Convolvulus arvensis*	3	PTU_457
		*Echinochloa colona*	3	PTU_123
		*Echinochloa crus-galli*	3	PTU_123
		*Echinochloa oryzicola*	3	PTU_123
**KR18-37**	2	*Convolvulus arvensis*	3	PTU_131
		*Dactyloctenium aegyptium*	2	PTU_187
		*Damrongia orientalis*	2	PTU_253
**KR18-39**	3	*Damrongia orientalis*	3	PTU_263
**KR18-40**	3	*Damrongia orientalis*	3	PTU_500
**KR18-43**	3	*Amaranthus hybridus*	3	PTU_146
		*Amaranthus viridis*	3	PTU_146
		*Convolvulus arvensis*	3	PTU_166
		*Dactyloctenium aegyptium*	3	PTU_187
		*Damrongia orientalis*	3	PTU_253
		*Hymenachne grumosa*	3	PTU_249
		*Ipomoea batatas*	2	PTU_165
		*Ipomoea sp. GO-2017*	2	PTU_165
		*Ipomoea triloba*	2	PTU_165
**KR18-49**	3	*Cenchrus americanus*	3	PTU_096
		*Cynodon dactylon*	3	PTU_119
		*Dactyloctenium aegyptium*	3	PTU_187
		*Echinochloa colona*	3	PTU_409
		*Echinochloa frumentacea*	3	PTU_409
		*Ipomoea batatas*	3	PTU_165
		*Ipomoea sp. GO-2017*	3	PTU_165
		*Ipomoea triloba*	3	PTU_165
		*Portulaca oleracea*	3	PTU_051
		*Damrongia orientalis*	2	PTU_500
**KR18-51**	0	*Amaranthus hybridus*	3	PTU_146
		*Amaranthus viridis*	3	PTU_146
		*Convolvulus arvensis*	3	PTU_595
		*Corchorus olitorius*	3	PTU_628
		*Dinebra panicea subsp. brachiata*	3	PTU_251
		*Dinebra panicea subsp. mucronata*	3	PTU_251
		*Dinebra retroflexa*	3	PTU_251
		*Echinochloa colona*	3	PTU_409
		*Echinochloa frumentacea*	3	PTU_409
		*Hymenachne grumosa*	3	PTU_090
		*Leptochloa filiformis*	3	PTU_251
		*Origanum vulgare*	2	PTU_254
**KR18-56**	3	*Damrongia orientalis*	3	PTU_662
**KR18-59**	0	*Amaranthus hybridus*	3	PTU_146
		*Amaranthus viridis*	3	PTU_146
		*Convolvulus arvensis*	3	PTU_010
		*Hymenachne grumosa*	3	PTU_249
		*Origanum vulgare*	3	PTU_339

## Discussion

Overall, DNA metabarcoding detected considerable inconsistencies between the identified species and those listed on the product labels. Our results indicate that only five products (four paprika and one oregano) come with correct label information, and contained precisely the one listed plant-based ingredient. For 47 samples (10 paprika, 22 oregano, 14 basil, and one oregano-basil), the target plant species as well as additional other plant species were detected. For seven samples (three paprika, one oregano, and three basil) the target plant species was not identified. Eight samples (all labeled as containing paprika) did not yield useful information for the taxonomic identification after applying the quality filtering criteria. In summary 54 out of 62 samples contained other species than the ones listed on the product.

The discrepancies between the species detected using DNA metabarcoding and those listed on the product labels require a careful consideration of possible contamination. In this study we used *a priori* information from the product labels to establish a hypothesis of the target species for each product, as well as a putative origin of the raw plant material and cultivation conditions. The origin and cultivation conditions can help us narrow down identifications from the sequencing data. *A posteriori* data in this sense are the putative ASV identifications, i.e., the species detected in the products. Information on the geographic range, growth habit or cultivation status of the detected species can aid in evaluating the results. For instance, in this study, we detect numerous wind-pollinated and wind-spread species, and their presence could be the result of (insignificant) trace contamination. However, when interpreting these results, we should also bear in mind that DNA metabarcoding is a highly sensitive detection method, and even trace amounts of pollen or plant dust left in production equipment can be detected. It is therefore important that herbal product authentication using DNA metabarcoding focuses on the presence of a target species. However, this does require a case-based analysis that considers the experimental steps (e.g., sample preparation, library preparation, and HTS) and post-bioinformatics analysis that may yield false positive or false negative results (Robasky et al., 2014; Ficetola et al., 2015). In this study, technical replicates were used to limit the effects of false positive and false negative results (Robasky et al., 2014; Ficetola et al., 2015). In addition, efforts were made to overcome the amount of sequencing errors associated with the Ion Torrent sequencing platform (Loman et al., 2012). Such sequencing errors may lead to the formation of false ASVs. Hence, we included three technical replicates per sample and applied strict filtering and trimming thresholds for base call length and quality. Speranskaya et al. (2018) performed a comparative study of the two most widely used sequencing platforms, Illumina and Ion Torrent, to assess the composition of herbal teas. The two methods were found to be qualitatively and quantitatively consistent, with a certain level of variation between runs on the same platform which were more likely due to the stochastic dynamics of PCR, or other reactions during the library preparation. The large number of non-listed plant species detected is therefore likely a result of contamination and amplification bias, i.e., PCR chimeras, sequencing errors, or false-positive taxonomic identifications due to error-prone barcode sequences reference databases (Ficetola et al., 2010; Robasky et al., 2014; Pawluczyk et al., 2015). Additionally metabarcoding does not always resolve closely related plant species. This can lead to incorrect identifications if the database used does not contain all possible species. For example, in this study it is very unlikely that the products labeled as containing basil contain *Ocimum americanum*, even though this is actually the result of our data analysis. The presence of this closely related species in our results is more likely due the fact that *Ocimum basilicum* cannot be discriminated from *Ocimum americanum* using the genetic marker internal transcribed spacer 2 (ITS2) due to the low level of interspecific genetic diversity between these species. On the other hand, overlooked species can also be explained by false negative detections, which can be the case if the plant DNA is too heavily degraded or even removed during the production process (Novak et al., 2007; Cheng et al., 2014), there is a poor primer-template match (Piñol et al., 2015), amplification stochasticity due to low DNA concentration (Giguet-covex et al., 2014), or incomplete barcode sequences in the reference databases. In this regard, an interesting result of this study is that we are confident in our negatives for basil and oregano, but not those of paprika. For instance, we can visually observe that the processing of basil and oregano should not degrade its DNA, and thus we expect to detect basil and oregano DNA if the species are present. Consequently, the absence of basil or oregano DNA in a product is a strong indicator that they are truly absent. For paprika powder on the other hand, it is very hard to determine with certainty that these products include paprika. When we cannot detect paprika DNA, and consider all the processing steps that might have degraded or removed the DNA, it is difficult to ascertain whether paprika is truly absent or whether its DNA is degraded beyond the point that amplification of the ITS2 marker is possible. To reduce the uncertainty of the results, we used three technical replicates per sample, in addition to strict filtering and trimming thresholds for base call length and quality, and strict clustering criteria for ASV formation. The use of replicates has been reported to considerably reduce the risk of missing any present taxa at the expense of substantially increasing sequencing costs and time (Ficetola et al., 2015). Moreover, PCR-free approaches have been proposed to overcome limitations associated with amplification and to improve barcoding in taxonomically difficult plant groups in which traditional barcodes do not always provide sufficient taxonomic resolution (Kress and Erickson, 2007; Fazekas et al., 2009). For instance, “extended barcodes” that use low-coverage shotgun sequencing of genomic DNA (Straub et al., 2012; Coissac et al., 2016) and “super-barcodes” that use target capture sequencing (Mamanova et al., 2010; Li et al., 2015) are two PCR-free techniques that can be used for sample and product identification and authentication (Manzanilla et al., 2018; Haiminen, 2019). However, insufficient testing and the unavailability of extensive plastome or mitochondrial genome reference databases currently limit the applicability of these methods for the authentication of complex multi-ingredient plant-based products.

Any authentication strategy for quality control needs to pass analytical validation before it can be used in quality monitoring programs in a regulatory context and/or in supply chain management systems by the industry sector. This is particularly important since the quality, reliability, and consistency of analytical results are used to judge the quality and authenticity of a product. DNA-based methods for molecular diagnosis are some of the most promising prospective standards for quality confidence, though they require further validation (Bruno et al., 2019; Newmaster et al., 2019). Validating DNA metabarcoding is however challenging. Firstly, DNA metabarcoding does not provide information on the active metabolites in the raw plant material or the resulting preparations. This narrows its applicability to the identification of target plant species and confirmation of presence – but not absence – of other species, possible contaminants, and adulterants. Thus, DNA metabarcoding is recommended as a method to complement traditional analytical methods rather than be used alone (Raclariu et al., 2018a). Additionally, DNA metabarcoding cannot quantify relative species abundance since there are a number of confounding factors that can affect read numbers (Staats et al., 2016). Nevertheless, DNA metabarcoding can be used to analyze a diverse range of samples that are very often multi-ingredient and highly processed. It is a powerful method for the non-targeted identification of all taxa simultaneously in a product at any processing or production stage. This represents a key advantage over traditional DNA barcoding that is limited to the identification of single ingredients in raw materials (Raclariu et al., 2018a). However, proper analytical validation of the method is challenging since a product’s various extractions and processing steps can lead to loss, degradation, or mixing of DNA. While some qPCR and DNA barcoding methods are validated and standardized for quality control in commercial applications and regulatory contexts (Sgamma et al., 2017; Newmaster et al., 2019), no DNA metabarcoding protocols are yet established in these areas. Nevertheless, DNA metabarcoding addresses a number of the limitations of the currently used analytical methods for quality control, and we expect that validation studies will contribute to making it applicable in quality control systems.

## Conclusion

Biological identification and authentication approaches based on DNA metabarcoding can be successfully used for the authentication of herbs and spices-based products, for post-marketing control and to provide insight into the total species diversity in processed, multi-ingredient products. The use of DNA metabarcoding in combination with appropriate traditional chemical methods can considerably increase the reliability of the quality control. However, proper analytical validation of DNA metabarcoding is necessary before it can be implemented for molecular diagnostics, both in quality monitoring programs in a regulatory context, and in supply chain management systems by the industry sector.

## Data Availability Statement

The data presented in the study are deposited in the ZENODO repository, accession number 4730123.

## Author Contributions

AR-M, JA, and HB conceived the experiment. AR-M carried out the molecular lab work together with JA, BT and AS-N and wrote the manuscript with HB. MK and NR carried out the bioinformatics analysis. All authors have read and approved the final version of the manuscript.

## Conflict of Interest

NR and MK are both founders of the company Researgene. The remaining authors declare that the research was conducted in the absence of any commercial or financial relationships that could be construed as a potential conflict of interest.

## Supplementary Material

Supplementary File 1 Information about the investigated herbs and spices products.

Supplementary File 2 DNA gel electrophoresis images.

Supplementary File 3 Species identification report and BLAST queries.

Supplementary File 4 DNA metabarcoding results for product identification.
