# Supplementary material for: DNA Metabarcoding for Quality Control of Basil, Oregano, and Paprika
Source: Front Plant Sci. 2021 Jun 4;12:665618. doi: 10.3389/fpls.2021.665618 (PMC8213367; doi:10.3389/fpls.2021.665618)
Supplement: Supplementary File 1 — Information about the investigated herbs and spices products. [file Data_Sheet_1.PDF]

S.1 Information about the investigated herbs and spices products

Sample ID	Package info	Ingredients according to product label
KR18-01	box 20 g	Oregano
KR18-02	box 20 g	Basil
KR18-03	box 35 g	Paprika
KR18-04	box 25 g	Paprika
KR18-05	Glass 9 g	Oregano
KR18-06	box 19 g	Oregano
KR18-07	Glass 40 g	Paprika
KR18-08	Glass 11 g	Basil
KR18-09	Glass 60g	Paprika
KR18-10	Glass 10g	Oregano
KR18-11	Glass 23g	Basil
KR18-12	bag	Oregano
KR18-13	bag	Paprika
KR18-14	bag	Oregano
KR18-15	bag	Oregano
KR18-16	bag	Paprika
KR18-17	bag	Paprika
KR18-18	bag	Basil
KR18-19	bag	Paprika
KR18-20	box	Basil
KR18-21	glass	Oregano
KR18-22	glass	Basil
KR18-23	glass	Paprika
KR18-24	box	Oregano
KR18-25	box	Basil
KR18-26	box	Paprika
KR18-27	box	Oregano
KR18-28	bag	Paprika
KR18-29	bag	Oregano
KR18-30	bag	Basil
KR18-31	bag	Oregano
KR18-32	bag	Basil
KR18-33	bag	Paprika
KR18-34	box	Paprika
KR18-35	box	Oregano and basil
KR18-36	bag 0,5 kg	Oregano
KR18-37	bag 0,5 kg	Basil
KR18-38	bag 0,5 kg	Paprika
KR18-39	box	Basil
KR18-40	bag	Basil
KR18-41	bag	Oregano
KR18-42	box	Oregano
KR18-43	bag	Basil
KR18-44	bag	Oregano
KR18-45	bag	Paprika
KR18-46	Glass	Paprika
KR18-47	box	Oregano
KR18-48	40g	Oregano
KR18-49	100g	Basil
KR18-50	bag	Oregano
KR18-51	bag	Basil
KR18-52	bag	Paprika
KR18-53	bag	Paprika
KR18-54	bag	Paprika
KR18-55	bag	Oregano
KR18-56	bag	Basil
KR18-57	bag	Oregano
KR18-58	bag	Paprika
KR18-59	bag	Basil
KR18-60	box	Paprika
KR18-61	bag	Oregano
KR18-62	bag	Oregano
