# Supplementary material for: DNA Metabarcoding for Quality Control of Basil, Oregano, and Paprika
Source: Front Plant Sci. 2021 Jun 4;12:665618. doi: 10.3389/fpls.2021.665618 (PMC8213367; doi:10.3389/fpls.2021.665618)
Supplement: Supplementary File 2 — DNA gel electrophoresis images. [file Data_Sheet_2.PDF]

S 2. Gel electrophoresis images. This images contains the information on amplification of all 62 amplicon libraries, of all three technical replicates, for ITS2 and the negative controls (also in triplicate), including DNA extraction negative and PCR negative. The gel image was acquired and analyzed using the ChemiDoc™ Touch Imaging System with Image Lab™ Touch Software, version 6.0.0 (Bio-Rad).

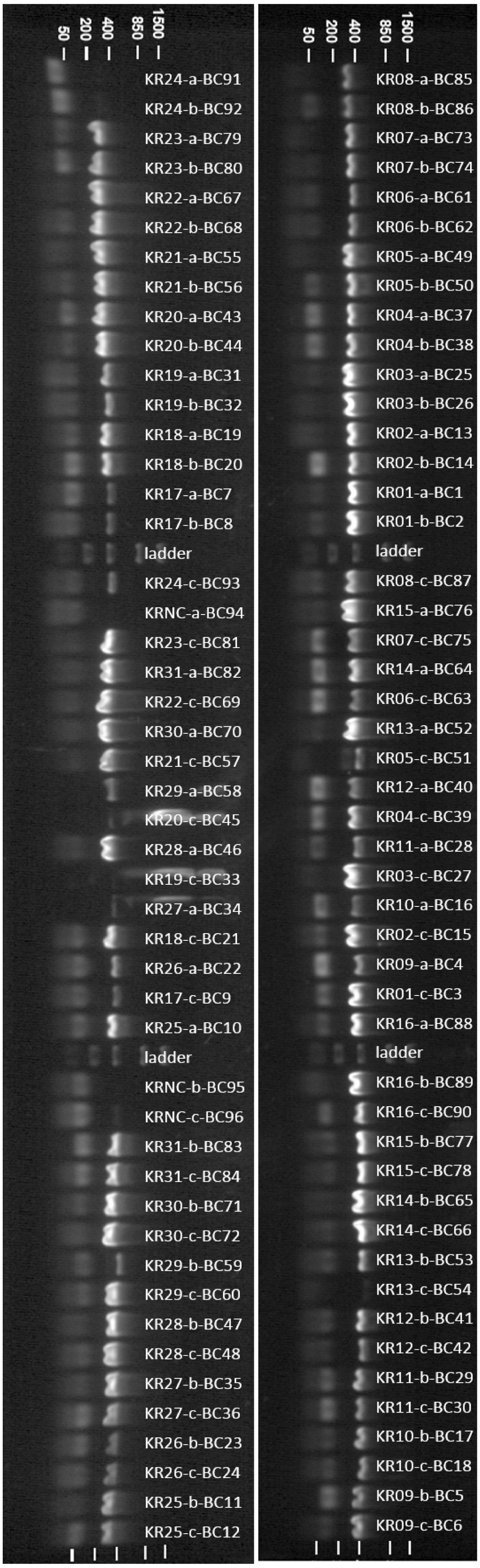

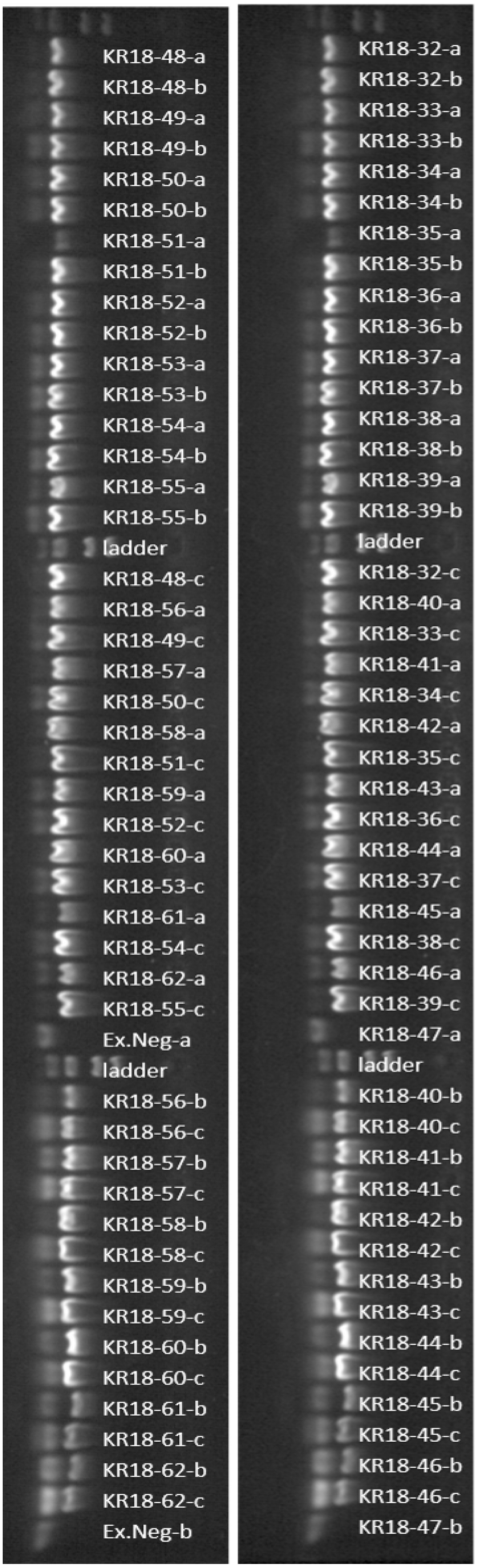
KR18-48-a
KR18-48-b
KR18-49-a
KR18-49-b
KR18-50-a
KR18-50-b
KR18-51-a
KR18-51-b
KR18-52-a
KR18-52-b
KR18-53-a
KR18-53-b
KR18-54-a
KR18-54-b
KR18-55-a
KR18-55-b
ladder
KR18-48-c
KR18-56-a
KR18-49-c
KR18-57-a
KR18-50-c
KR18-58-a
KR18-51-c
KR18-59-a
KR18-52-c
KR18-60-a
KR18-53-c
KR18-61-a
KR18-54-c
KR18-62-a
KR18-55-c
Ex.Neg-a
ladder
KR18-56-b
KR18-56-c
KR18-57-b
KR18-57-c
KR18-58-b
KR18-58-c
KR18-59-b
KR18-59-c
KR18-60-b
KR18-60-c
KR18-61-b
KR18-61-c
KR18-62-b
KR18-62-c
Ex.Neg-b
KR18-32-a
KR18-32-b
KR18-33-a
KR18-33-b
KR18-34-a
KR18-34-b
KR18-35-a
KR18-35-b
KR18-36-a
KR18-36-b
KR18-37-a
KR18-37-b
KR18-38-a
KR18-38-b
KR18-39-a
KR18-39-b
ladder
KR18-32-c
KR18-40-a
KR18-33-c
KR18-41-a
KR18-34-c
KR18-42-a
KR18-35-c
KR18-43-a
KR18-36-c
KR18-44-a
KR18-37-c
KR18-45-a
KR18-38-c
KR18-46-a
KR18-39-c
KR18-47-a
ladder
KR18-40-b
KR18-40-c
KR18-41-b
KR18-41-c
KR18-42-b
KR18-42-c
KR18-43-b
KR18-43-c
KR18-44-b
KR18-44-c
KR18-45-b
KR18-45-c
KR18-46-b
KR18-46-c
KR18-47-b
