# Supplementary material for: DNA Metabarcoding for Quality Control of Basil, Oregano, and Paprika
Source: Front Plant Sci. 2021 Jun 4;12:665618. doi: 10.3389/fpls.2021.665618 (PMC8213367; doi:10.3389/fpls.2021.665618)
Supplement: Supplementary File 3 — Species identification report and BLAST queries. [file Data_Sheet_3.PDF]

S 3. Species identification report and BLAST queries

Contents

Species identifications **4**

Identifications for KR18-01 5
Identifications for KR18-02 7
Identifications for KR18-03 9
Identifications for KR18-04 11
Identifications for KR18-05 13
Identifications for KR18-06 15
Identifications for KR18-07 17
Identifications for KR18-08 19
Identifications for KR18-09 21
Identifications for KR18-10 22
Identifications for KR18-11 24
Identifications for KR18-12 26
Identifications for KR18-13 28
Identifications for KR18-14 30
Identifications for KR18-15 32
Identifications for KR18-16 34
Identifications for KR18-17 35
Identifications for KR18-18 37
Identifications for KR18-19 39
Identifications for KR18-20 41
Identifications for KR18-21 43
Identifications for KR18-22 45
Identifications for KR18-23 47
Identifications for KR18-24 49
Identifications for KR18-25 51
Identifications for KR18-26 53
Identifications for KR18-27 55
Identifications for KR18-28 57
Identifications for KR18-29 59
Identifications for KR18-30 61
Identifications for KR18-31 63
Identifications for KR18-32 65
Identifications for KR18-33 67
Identifications for KR18-34 68
Identifications for KR18-35 70

Identifications for KR18-36 72
Identifications for KR18-37 74
Identifications for KR18-38 76
Identifications for KR18-39 78
Identifications for KR18-40 80
Identifications for KR18-41 82
Identifications for KR18-42 84
Identifications for KR18-43 86
Identifications for KR18-44 88
Identifications for KR18-45 90
Identifications for KR18-46 92
Identifications for KR18-47 93
Identifications for KR18-48 95
Identifications for KR18-49 97
Identifications for KR18-50 99
Identifications for KR18-51 101
Identifications for KR18-52 103
Identifications for KR18-53 104
Identifications for KR18-54 105
Identifications for KR18-55 106
Identifications for KR18-56 108
Identifications for KR18-57 110
Identifications for KR18-58 112
Identifications for KR18-59 114
Identifications for KR18-60 116
Identifications for KR18-61 117
Identifications for KR18-62 119
Identifications for KR18-ENC 121
Identifications for KR18-PNC 122

BLAST queries 123

Session Info 223

BLAST threshold used: 97%.

Species identifications

Identifications for KR18-01

Label: Oregano.

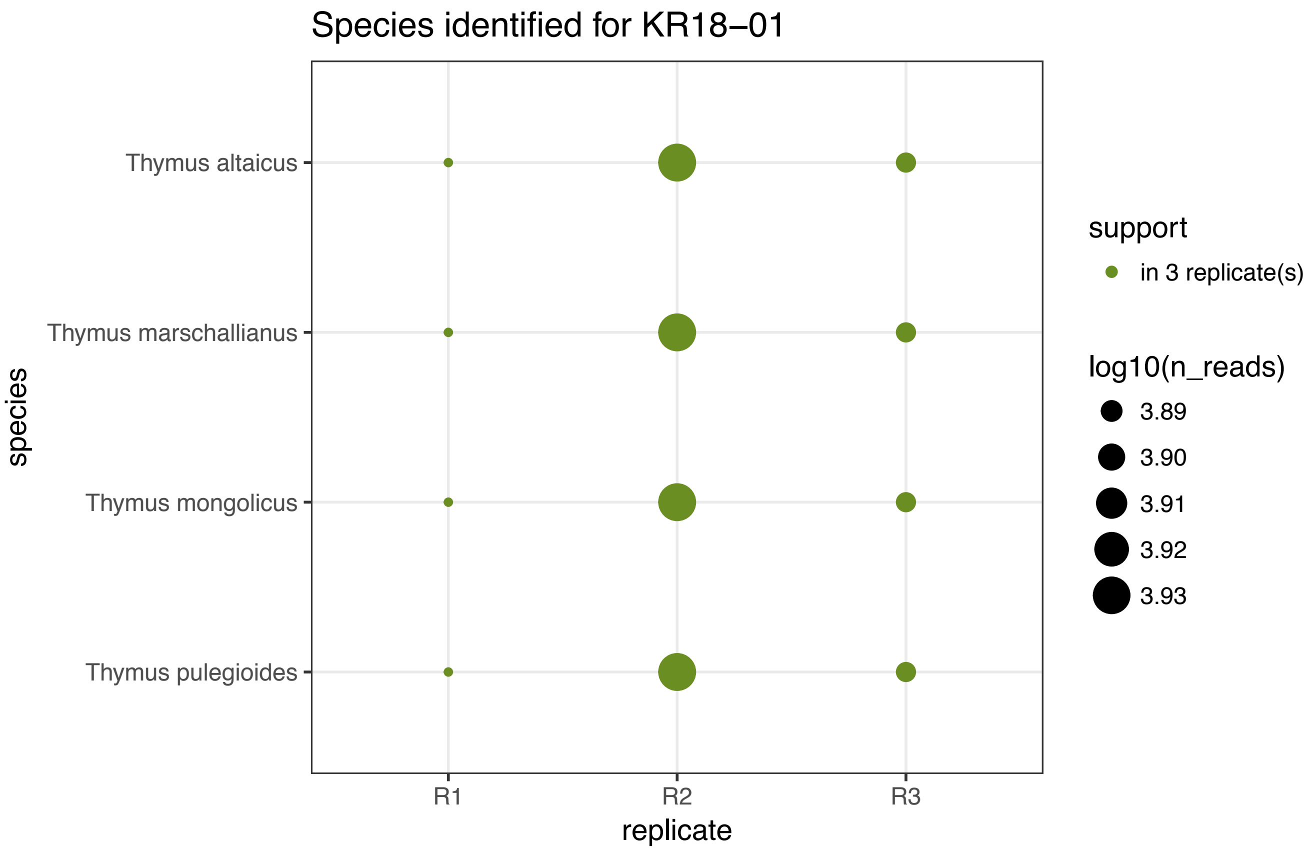

Table 1: Species identified in: KR18-01

species	support
Thymus altaicus	3
Thymus marschallianus	3
Thymus mongolicus	3
Thymus pulegioides	3

Identifications for KR18-02

Label: Basil.

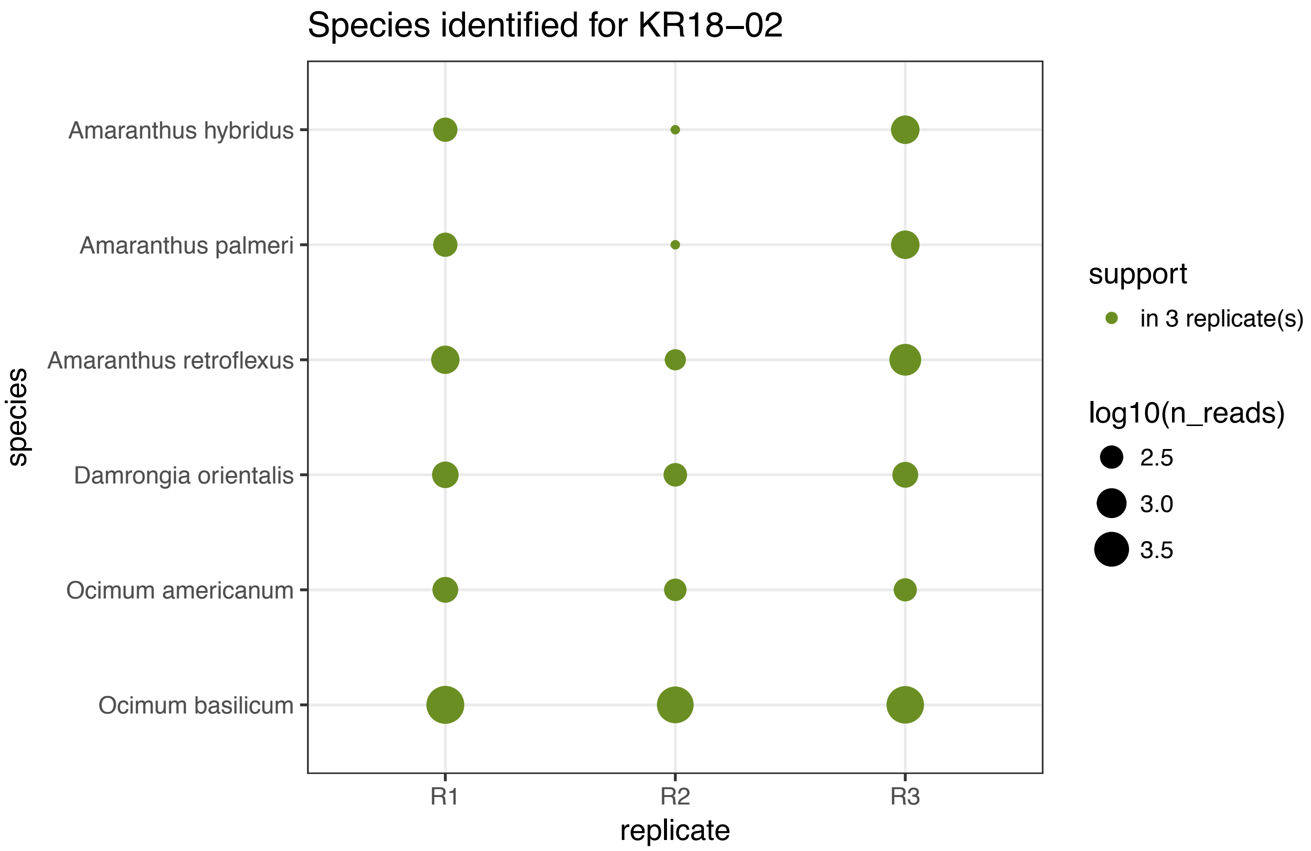

Table 2: Species identified in: KR18-02

species	support
Amaranthus hybridus	3
Amaranthus palmeri	3
Amaranthus retroflexus	3
Damrongia orientalis	3
Ocimum americanum	3
Ocimum basilicum	3

Identifications for KR18-03

Label: Paprika.

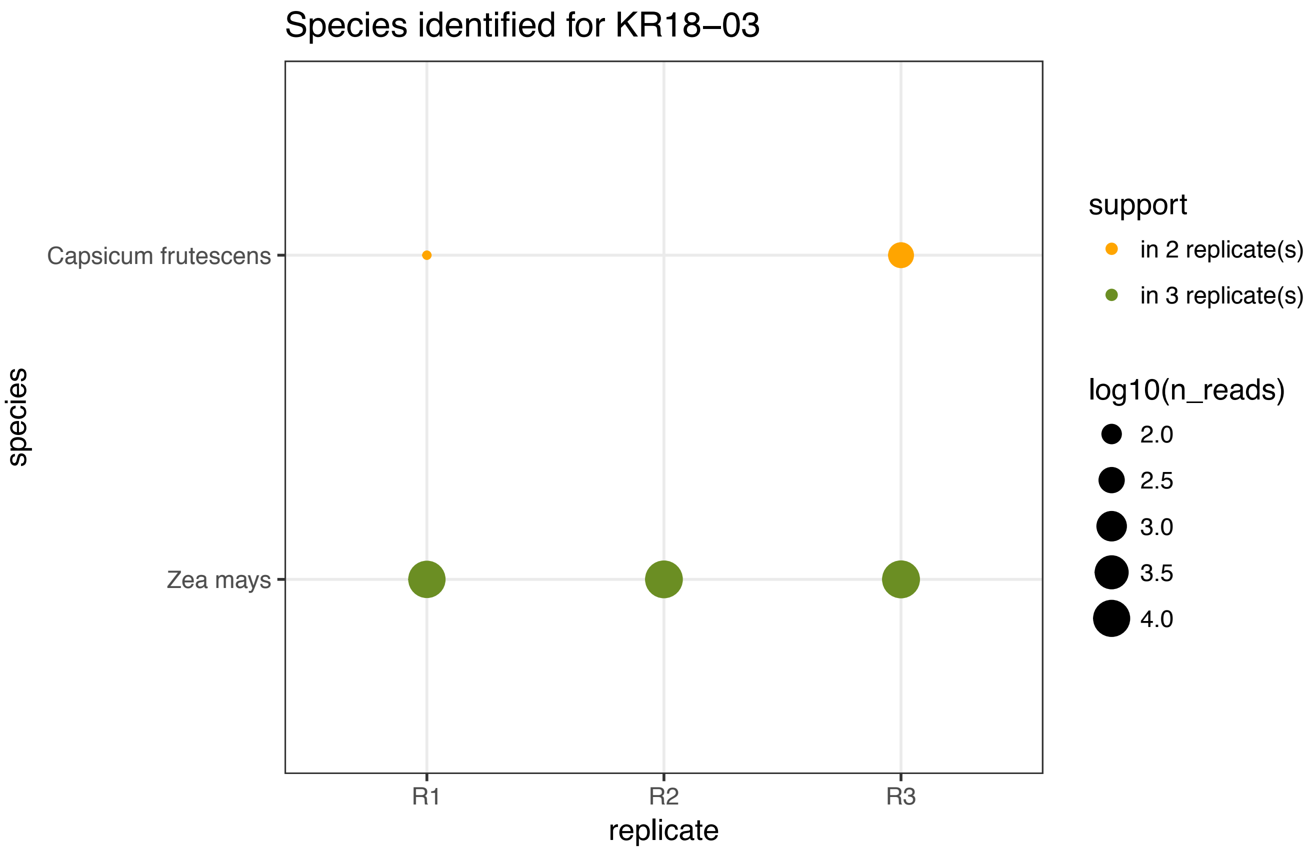

Table 3: Species identified in: KR18-03

species	support
Capsicum frutescens	2
Zea mays	3

Identifications for KR18-04

Label: Paprika.

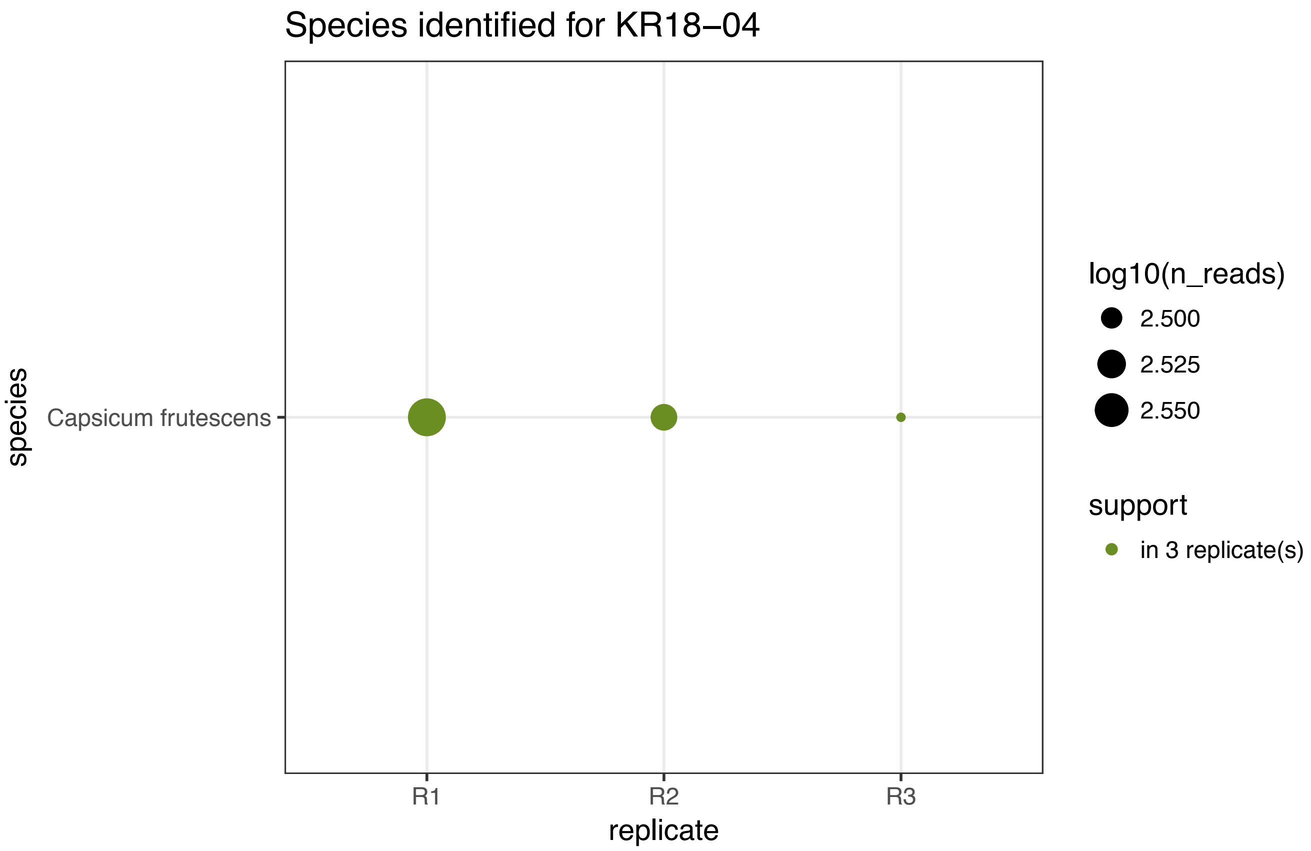

Table 4: Species identified in: KR18-04

species	support
Capsicum frutescens	3

Identifications for KR18-05

Label: Oregano.

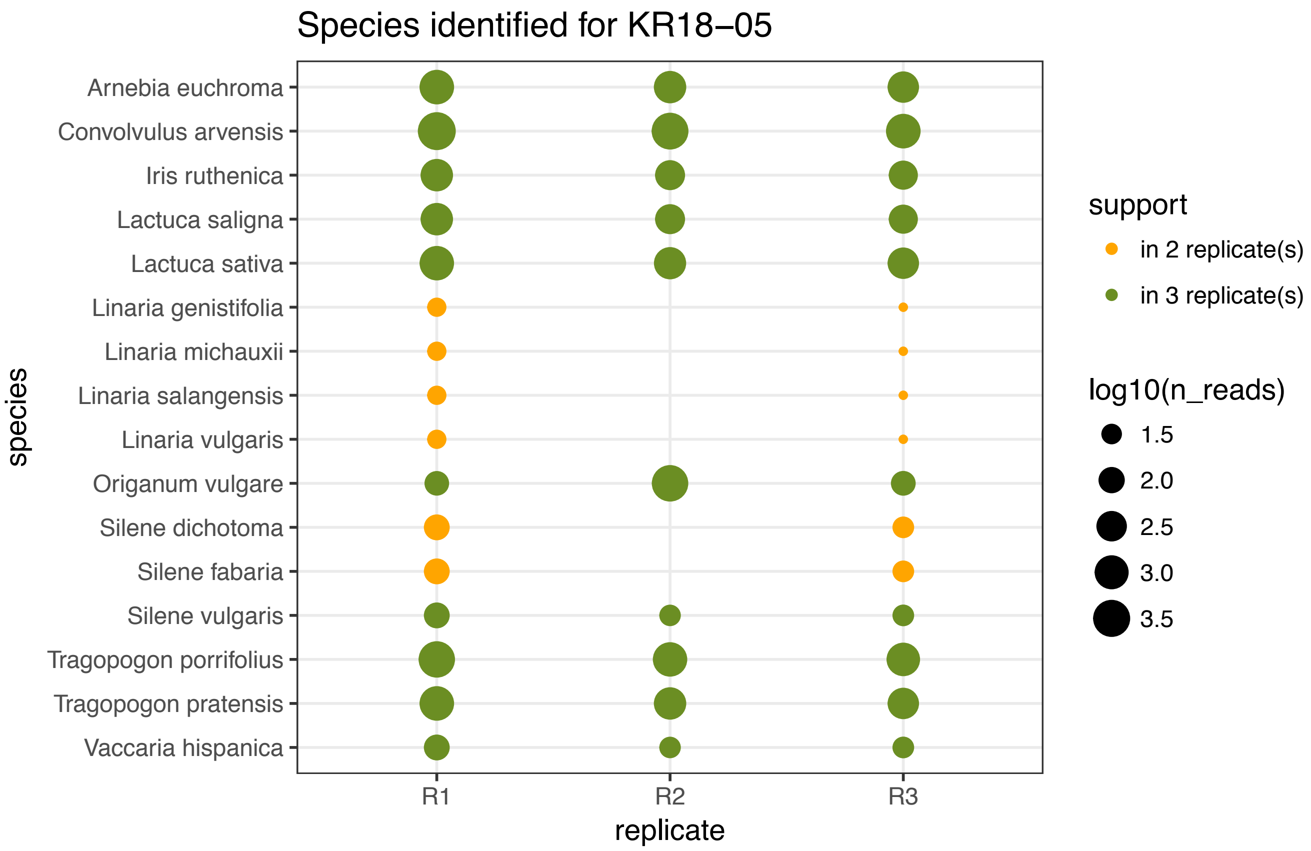

Table 5: Species identified in: KR18-05

species	support
Arnebia euchroma	3
Convolvulus arvensis	3
Iris ruthenica	3
Lactuca saligna	3
Lactuca sativa	3
Linaria genistifolia	2
Linaria michauxii	2
Linaria salangensis	2
Linaria vulgaris	2
Origanum vulgare	3
Silene dichotoma	2
Silene fabaria	2
Silene vulgaris	3
Tragopogon porrifolius	3
Tragopogon pratensis	3
Vaccaria hispanica	3

Identifications for KR18-06

Label: Oregano.

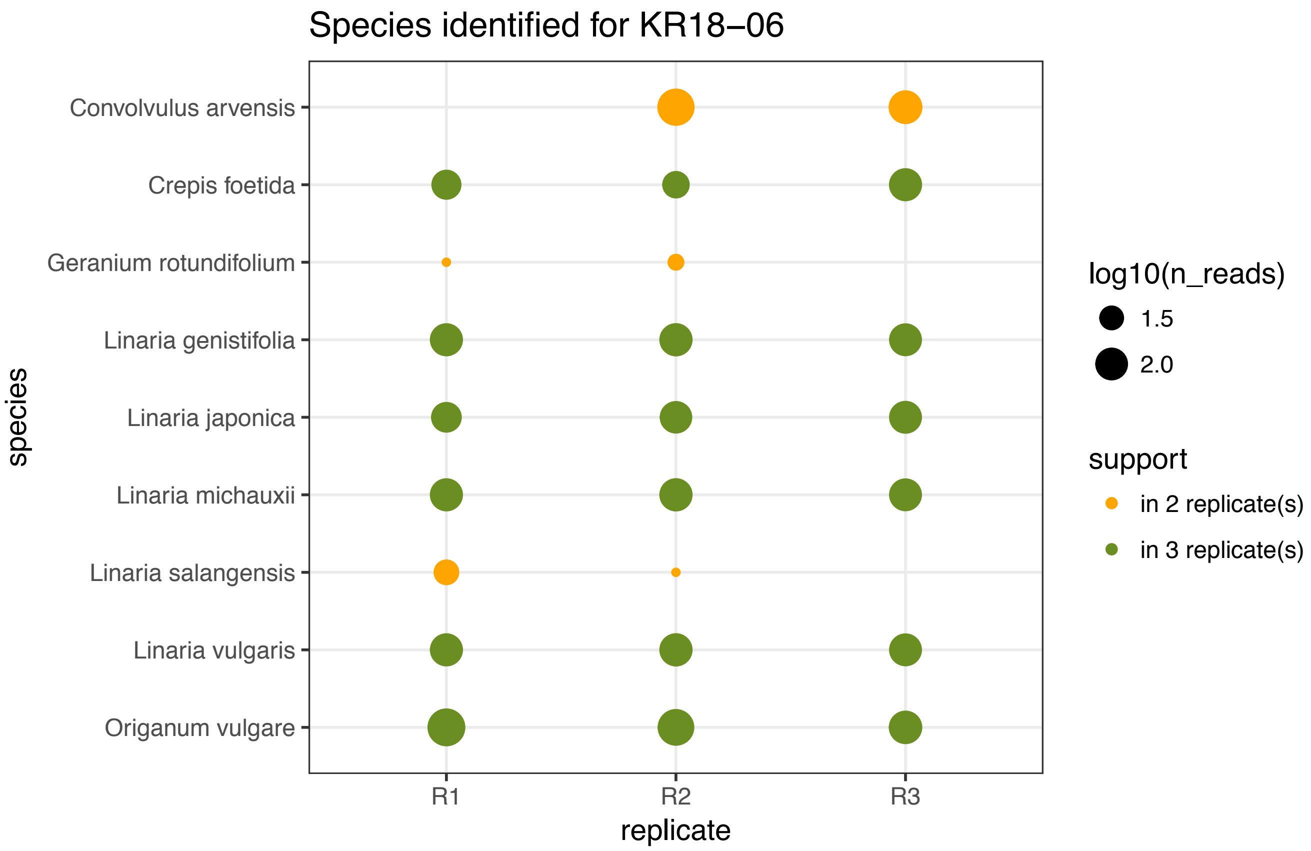

Table 6: Species identified in: KR18-06

species	support
Convolvulus arvensis	2
Crepis foetida	3
Geranium rotundifolium	2
Linaria genistifolia	3
Linaria japonica	3
Linaria michauxii	3
Linaria salangensis	2
Linaria vulgaris	3
Origanum vulgare	3

Identifications for KR18-07

Label: Paprika.

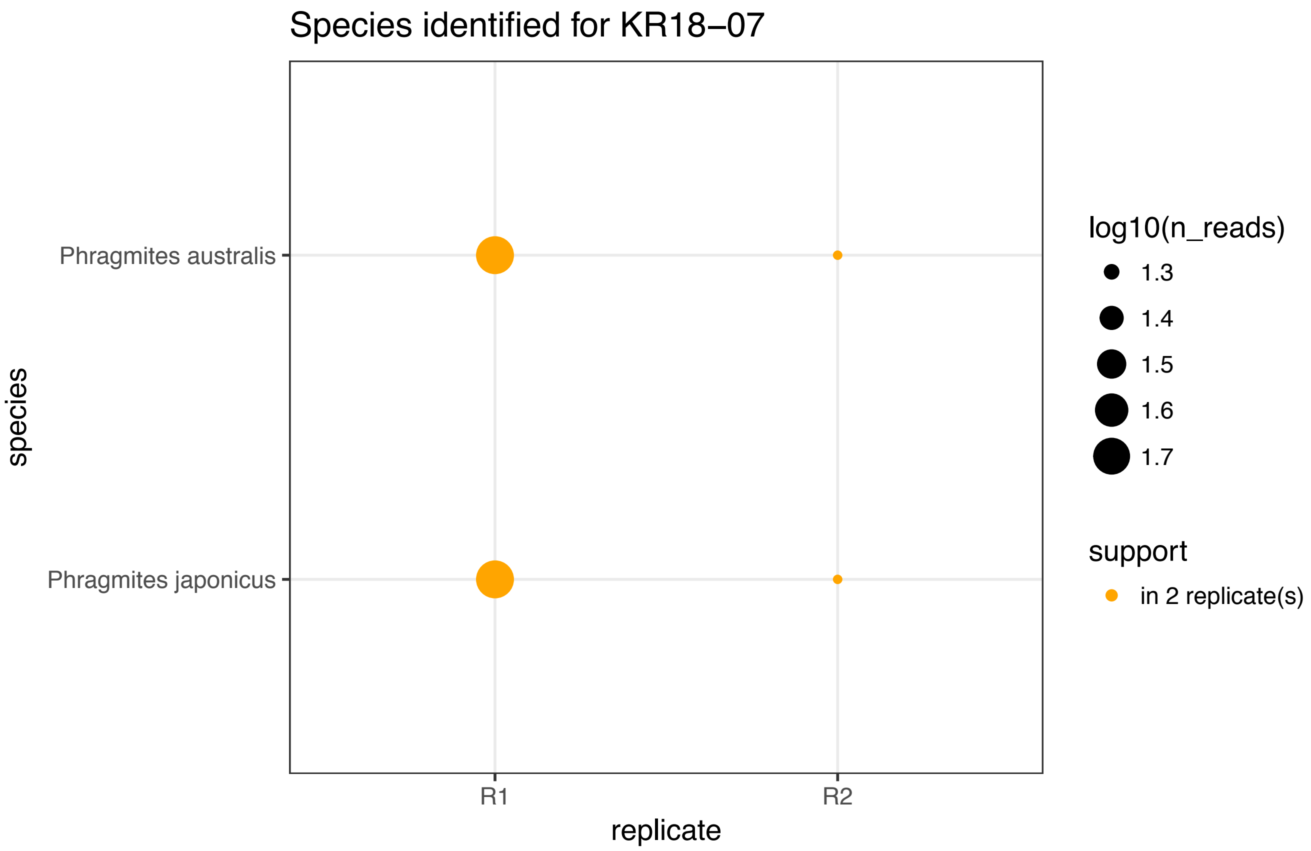

Table 7: Species identified in: KR18-07

species	support
Phragmites australis	2
Phragmites japonicus	2

Identifications for KR18-08

Label: Basil.

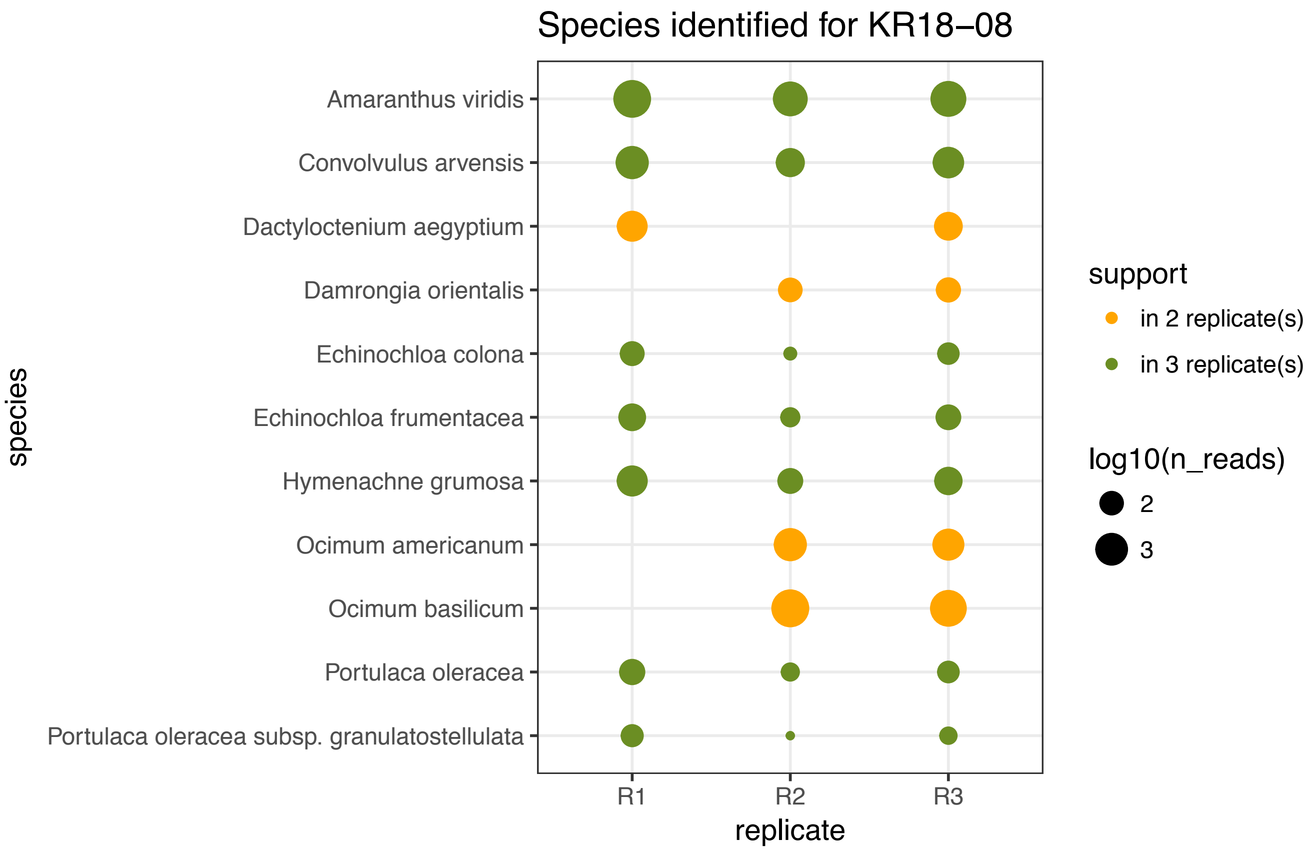

Table 8: Species identified in: KR18-08

species	support
Amaranthus viridis	3
Convolvulus arvensis	3
Dactyloctenium aegyptium	2
Damrongia orientalis	2
Echinochloa colona	3
Echinochloa frumentacea	3
Hymenachne grumosa	3
Ocimum americanum	2
Ocimum basilicum	2
Portulaca oleracea	3
Portulaca oleracea subsp. granulatostellulata	3

Identifications for KR18-09

Label: Paprika. [1] “No species have been identified for KR18-09.”

Identifications for KR18-10

Label: Oregano.

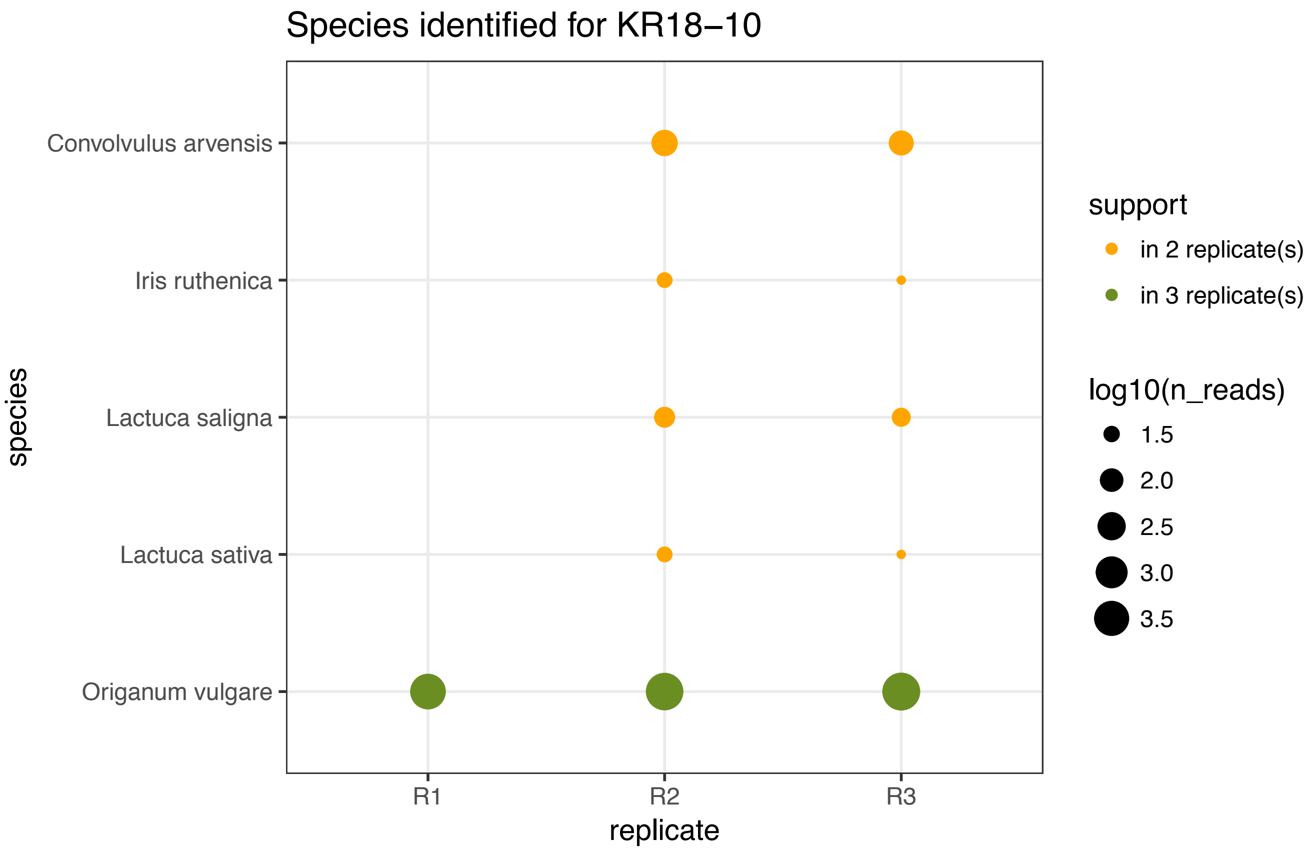

Table 9: Species identified in: KR18-10

species	support
Convolvulus arvensis	2
Iris ruthenica	2
Lactuca saligna	2
Lactuca sativa	2
Origanum vulgare	3

Identifications for KR18-11

Label: Basil.

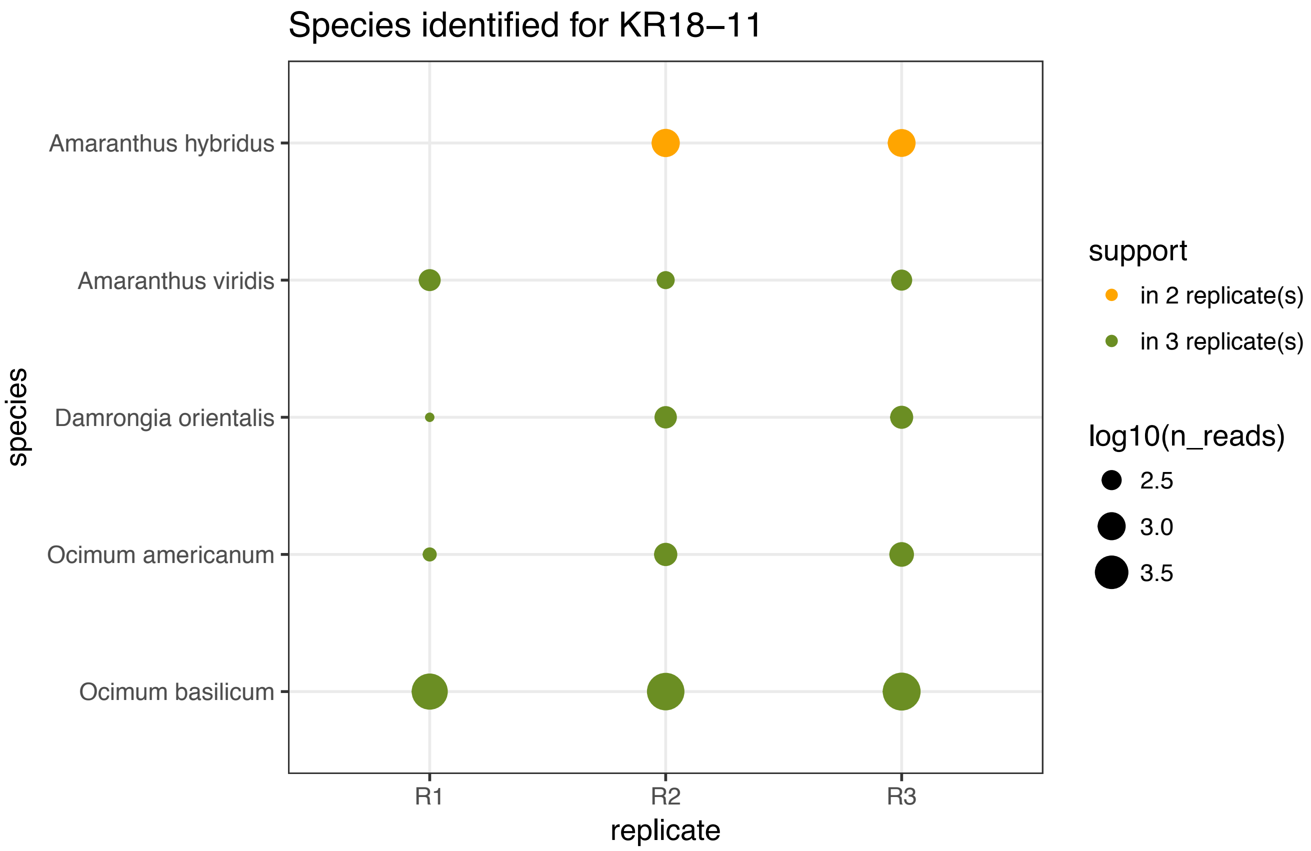

Table 10: Species identified in: KR18-11

species	support
Amaranthus hybridus	2
Amaranthus viridis	3
Damrongia orientalis	3
Ocimum americanum	3
Ocimum basilicum	3

Identifications for KR18-12

Label: Oregano.

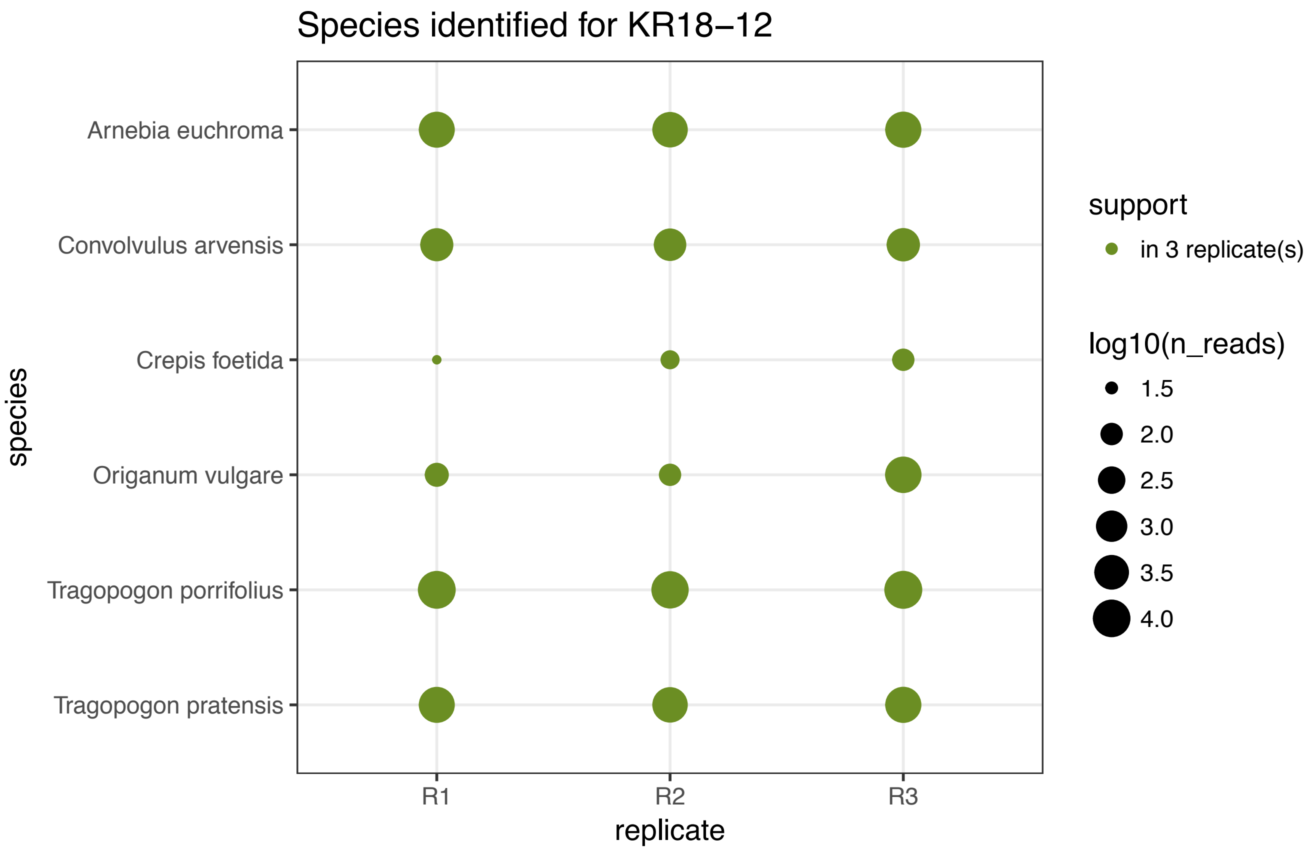

Table 11: Species identified in: KR18-12

species	support
Arnebia euchroma	3
Convolvulus arvensis	3
Crepis foetida	3
Origanum vulgare	3
Tragopogon porrifolius	3
Tragopogon pratensis	3

Identifications for KR18-13

Label: Paprika.

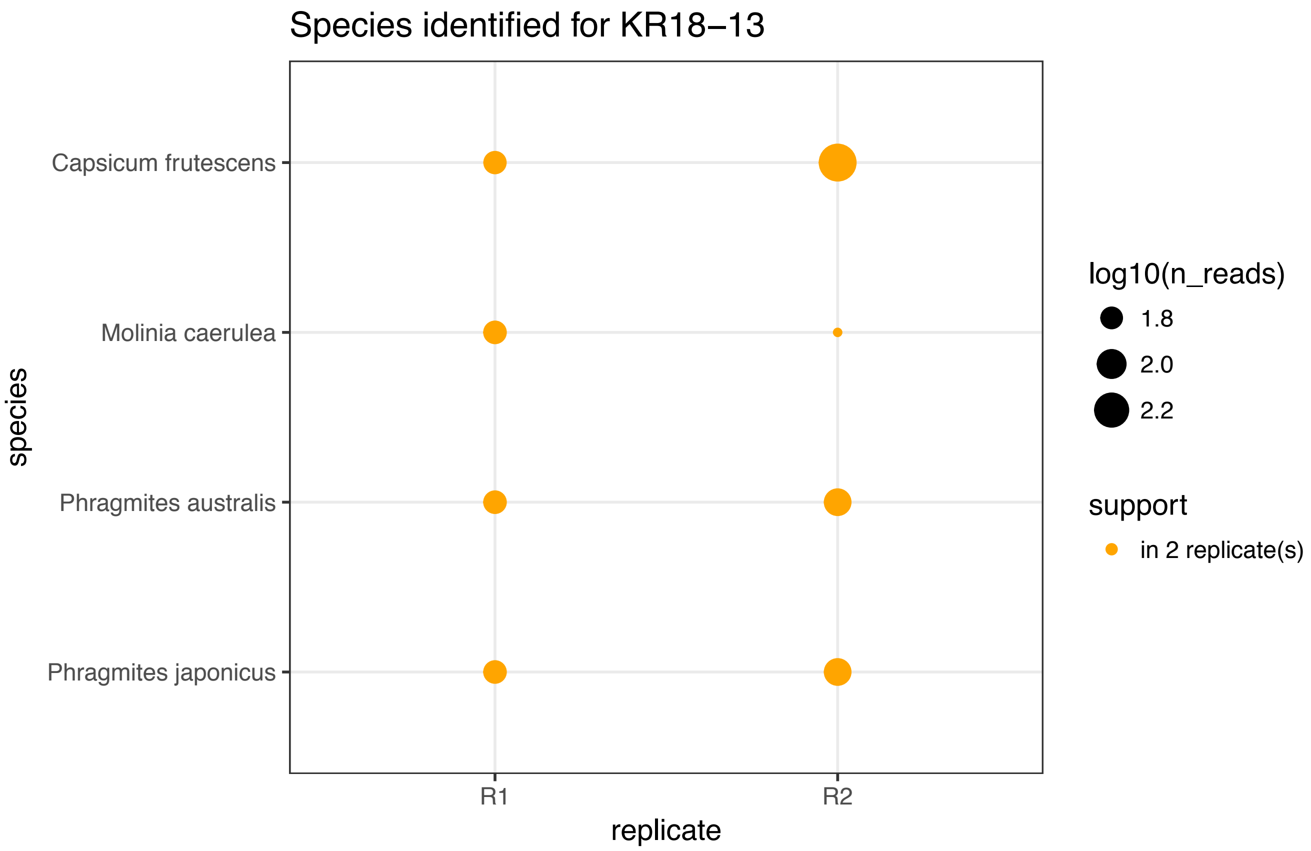

Table 12: Species identified in: KR18-13

species	support
Capsicum frutescens	2
Molinia caerulea	2
Phragmites australis	2
Phragmites japonicus	2

Identifications for KR18-14

Label: Oregano.

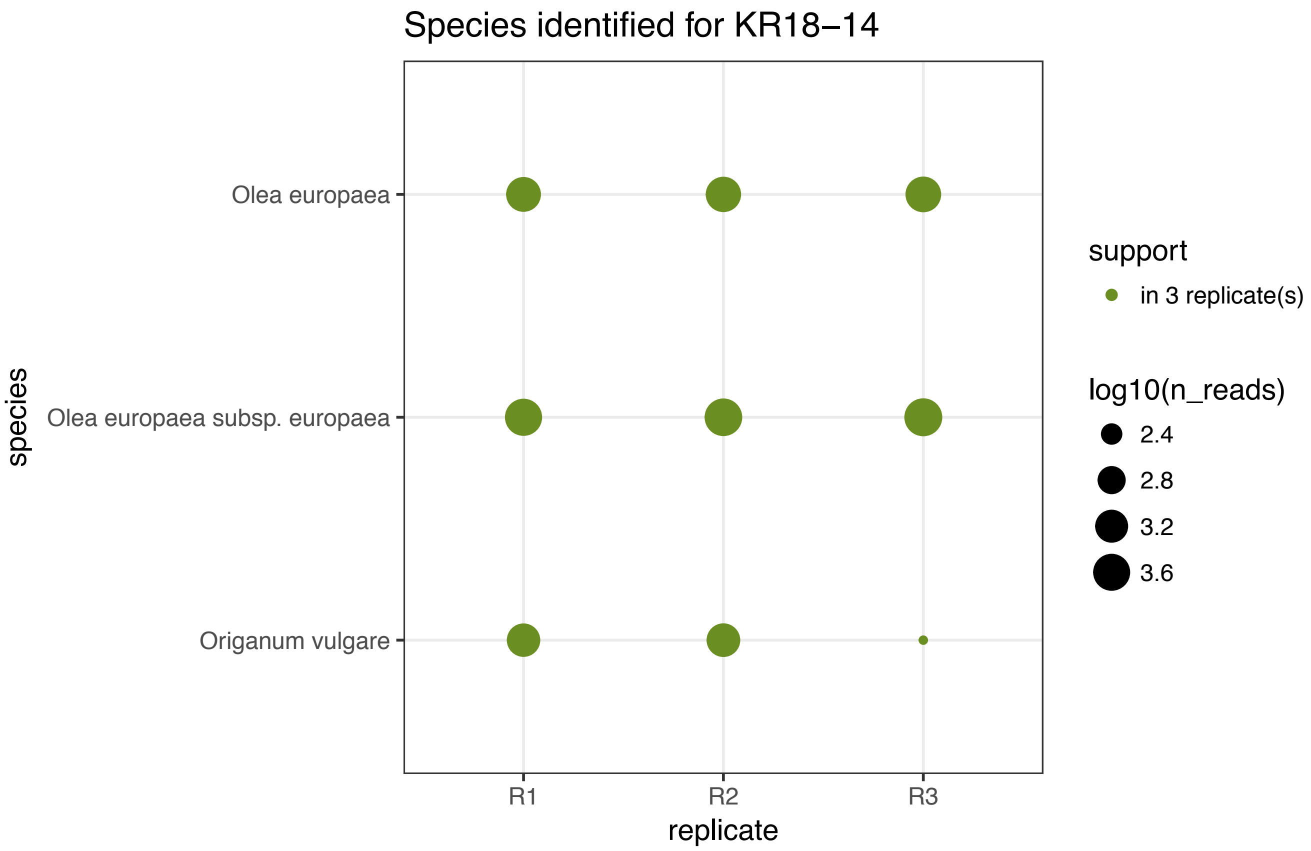

Table 13: Species identified in: KR18-14

species	support
Olea europaea	3
Olea europaea subsp. europaea	3
Origanum vulgare	3

Identifications for KR18-15

Label: Oregano.

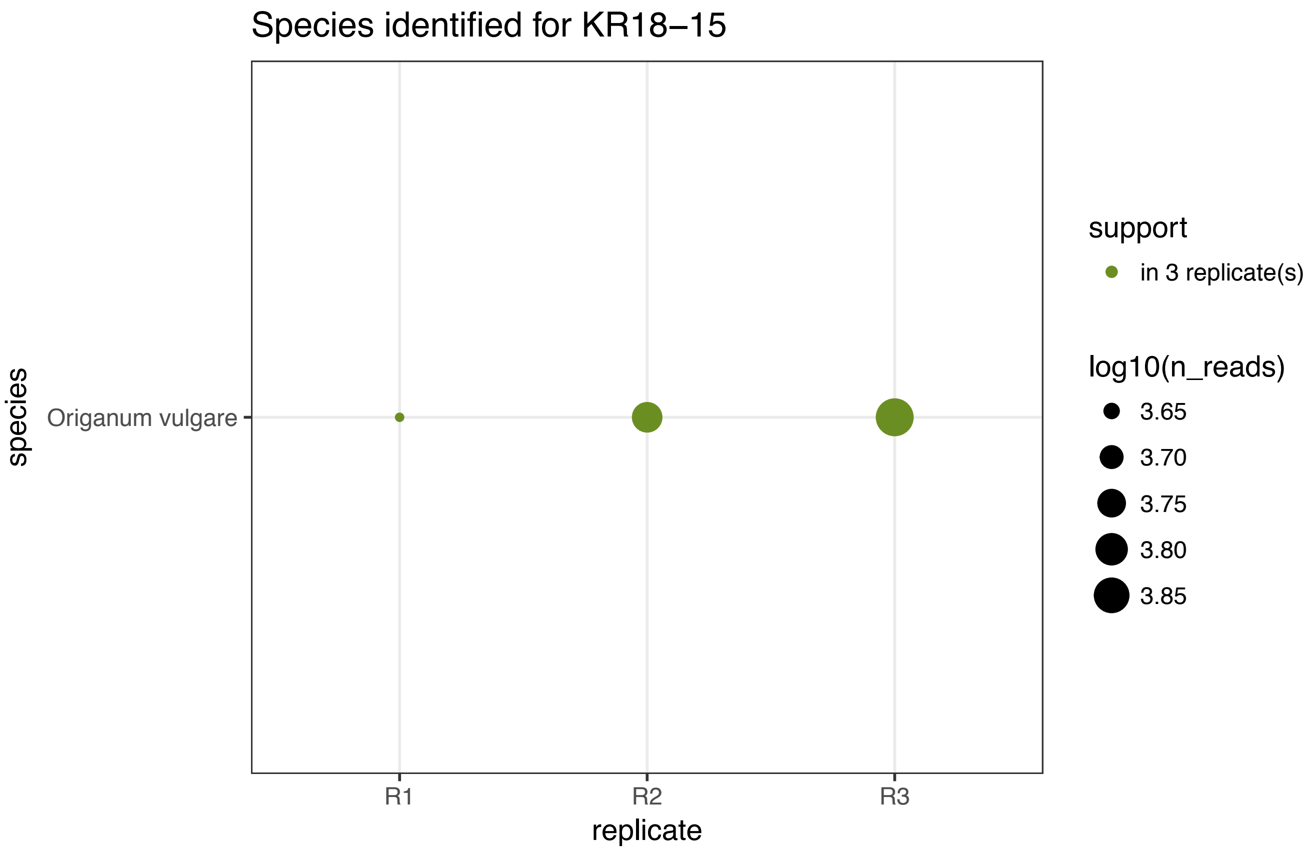

Table 14: Species identified in: KR18-15

species	support
Origanum vulgare	3

Identifications for KR18-16

Label: Paprika. [1] “No species have been identified for KR18-16.”

Identifications for KR18-17

Label: Paprika.

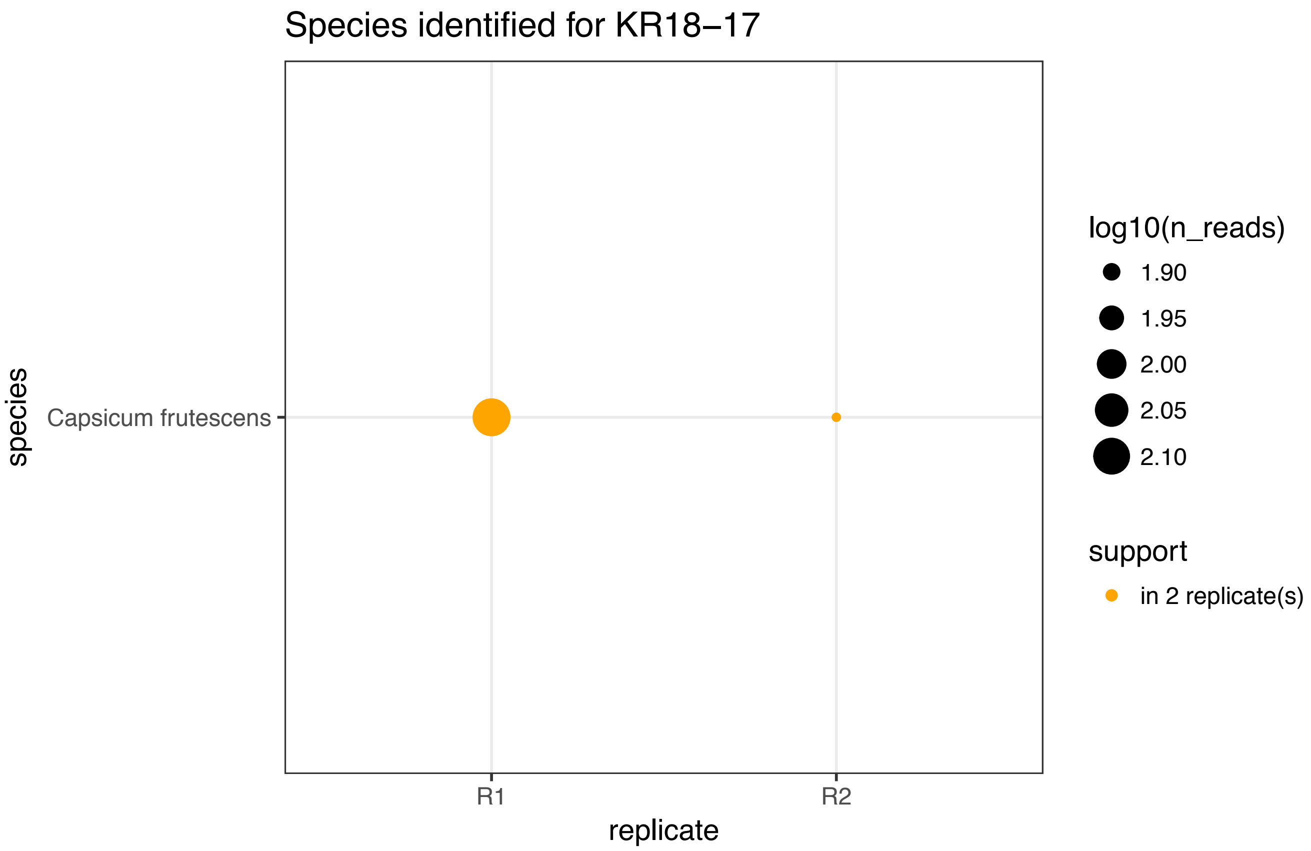

Table 15: Species identified in: KR18-17

species	support
Capsicum frutescens	2

Identifications for KR18-18

Label: Basil.

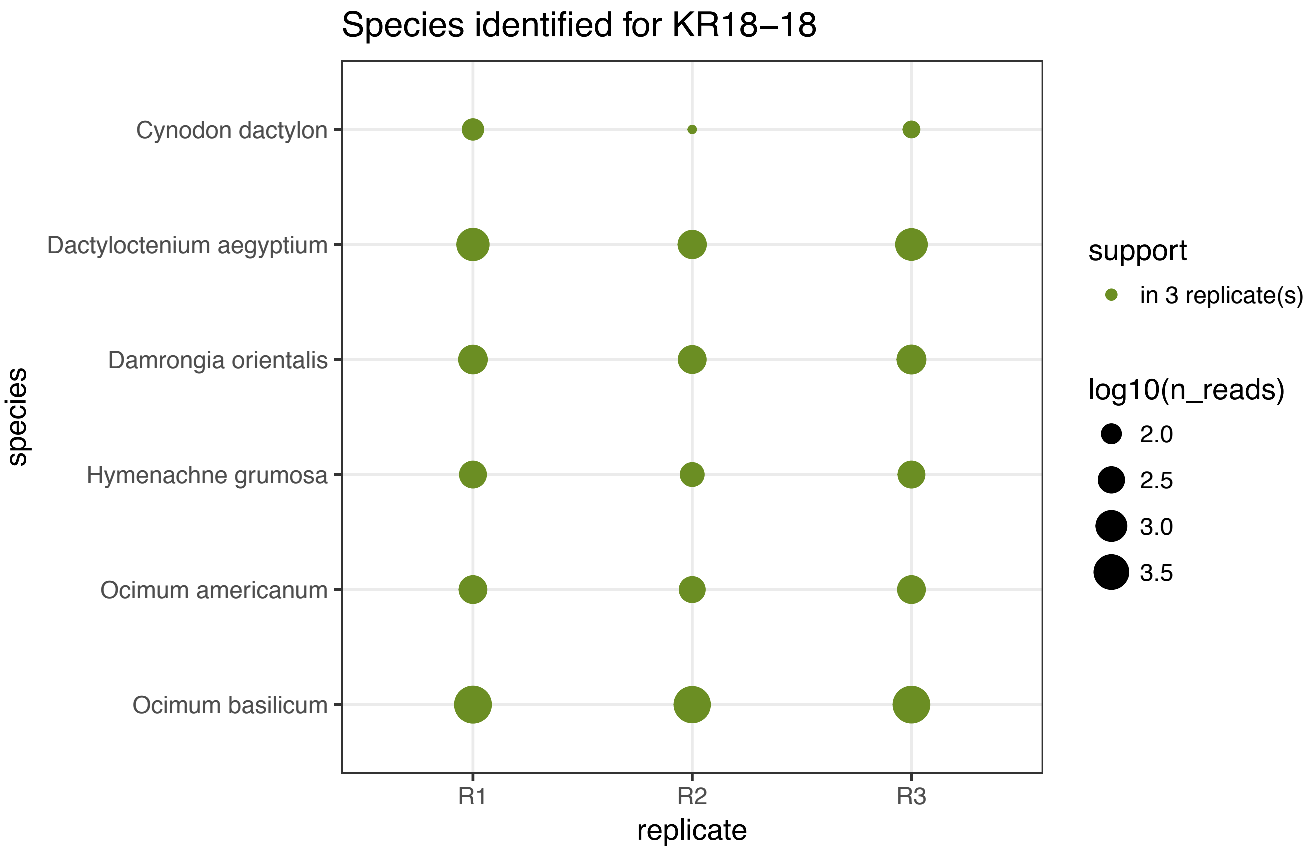

Table 16: Species identified in: KR18-18

species	support
Cynodon dactylon	3
Dactyloctenium aegyptium	3
Damrongia orientalis	3
Hymenachne grumosa	3
Ocimum americanum	3
Ocimum basilicum	3

Identifications for KR18-19

Label: Paprika.

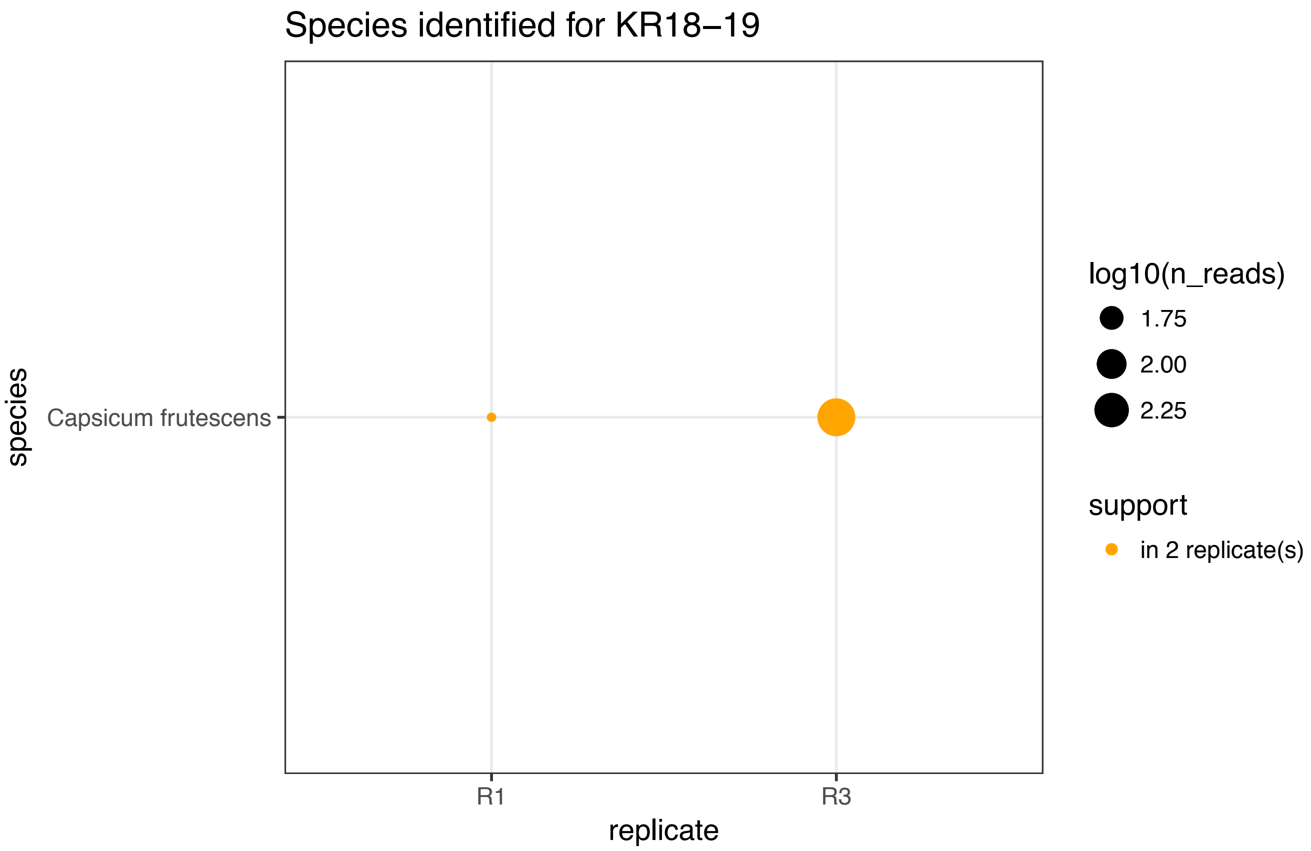

Table 17: Species identified in: KR18-19

species	support
Capsicum frutescens	2

Identifications for KR18-20

Label: Basil.

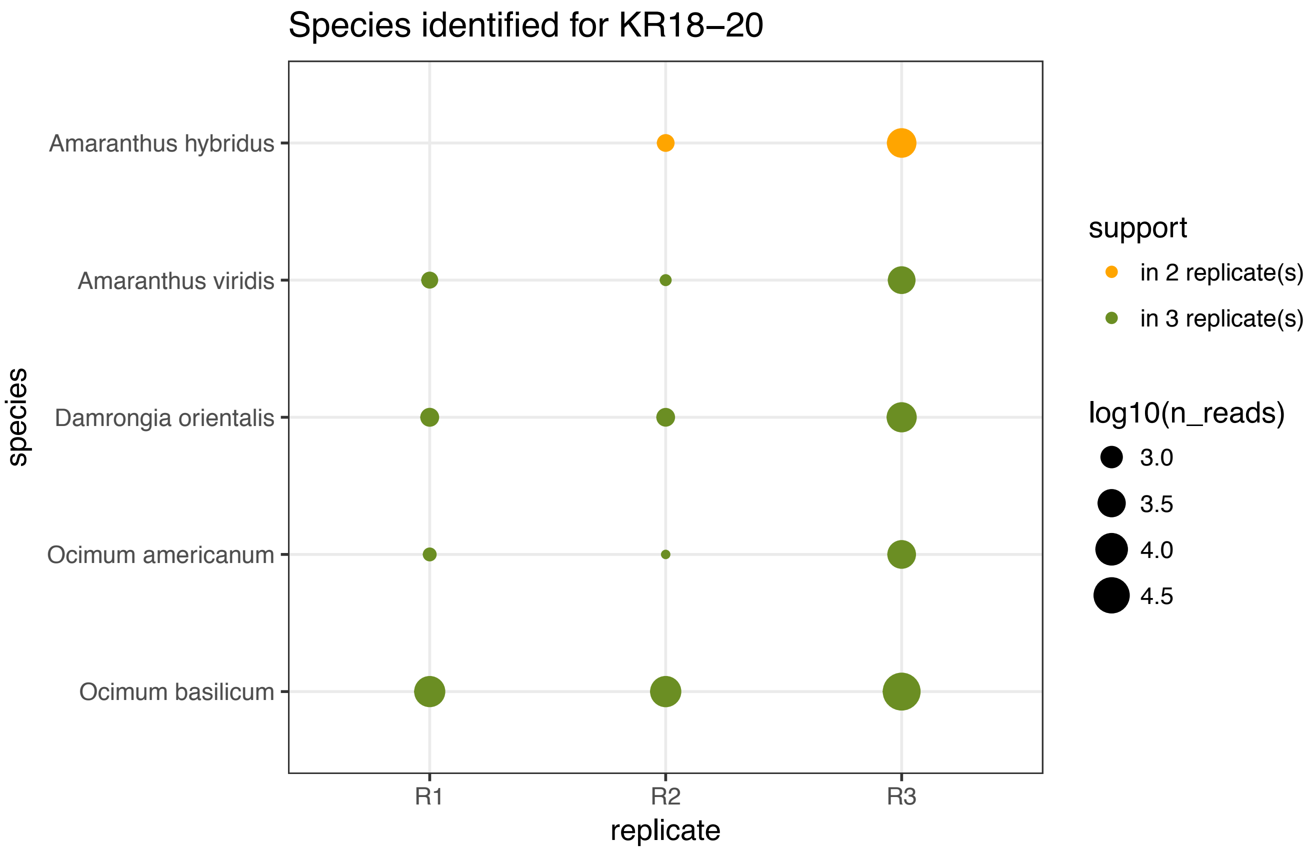

Table 18: Species identified in: KR18-20

species	support
Amaranthus hybridus	2
Amaranthus viridis	3
Damrongia orientalis	3
Ocimum americanum	3
Ocimum basilicum	3

Identifications for KR18-21

Label: Oregano.

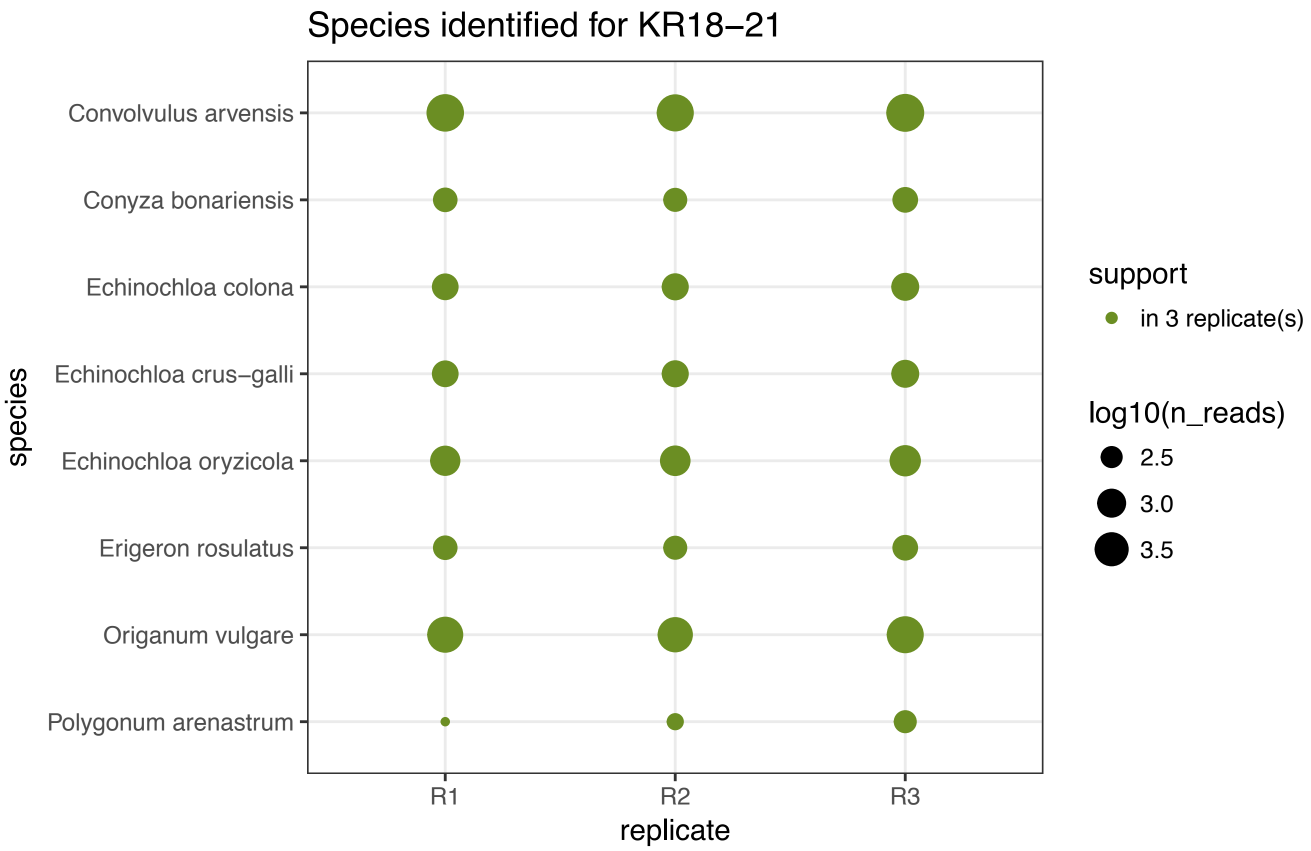

Table 19: Species identified in: KR18-21

species	support
Convolvulus arvensis	3
Conyza bonariensis	3
Echinochloa colona	3
Echinochloa crus-galli	3
Echinochloa oryzicola	3
Erigeron rosulatus	3
Origanum vulgare	3
Polygonum arenastrum	3

Identifications for KR18-22

Label: Basil.

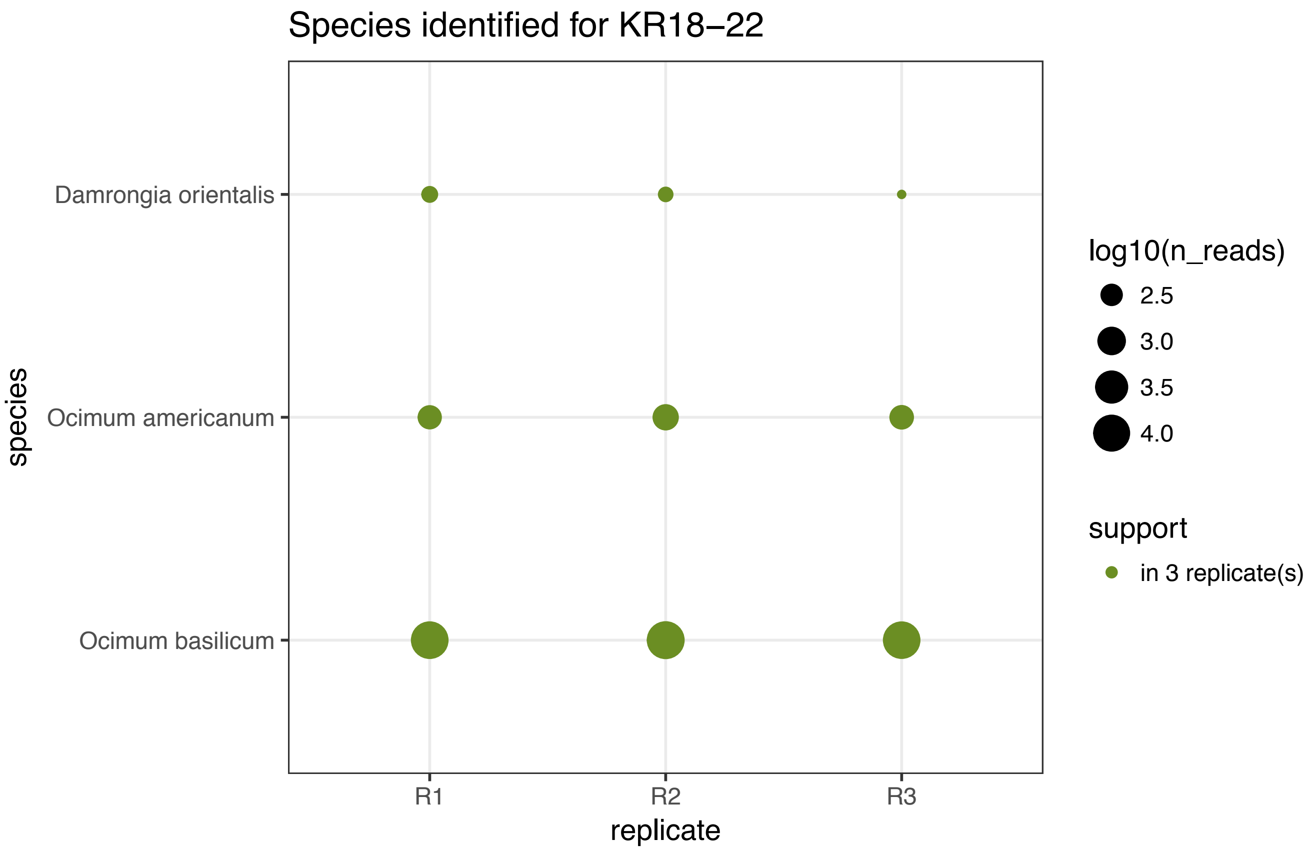

Table 20: Species identified in: KR18-22

species	support
Damrongia orientalis	3
Ocimum americanum	3
Ocimum basilicum	3

Identifications for KR18-23

Label: Paprika.

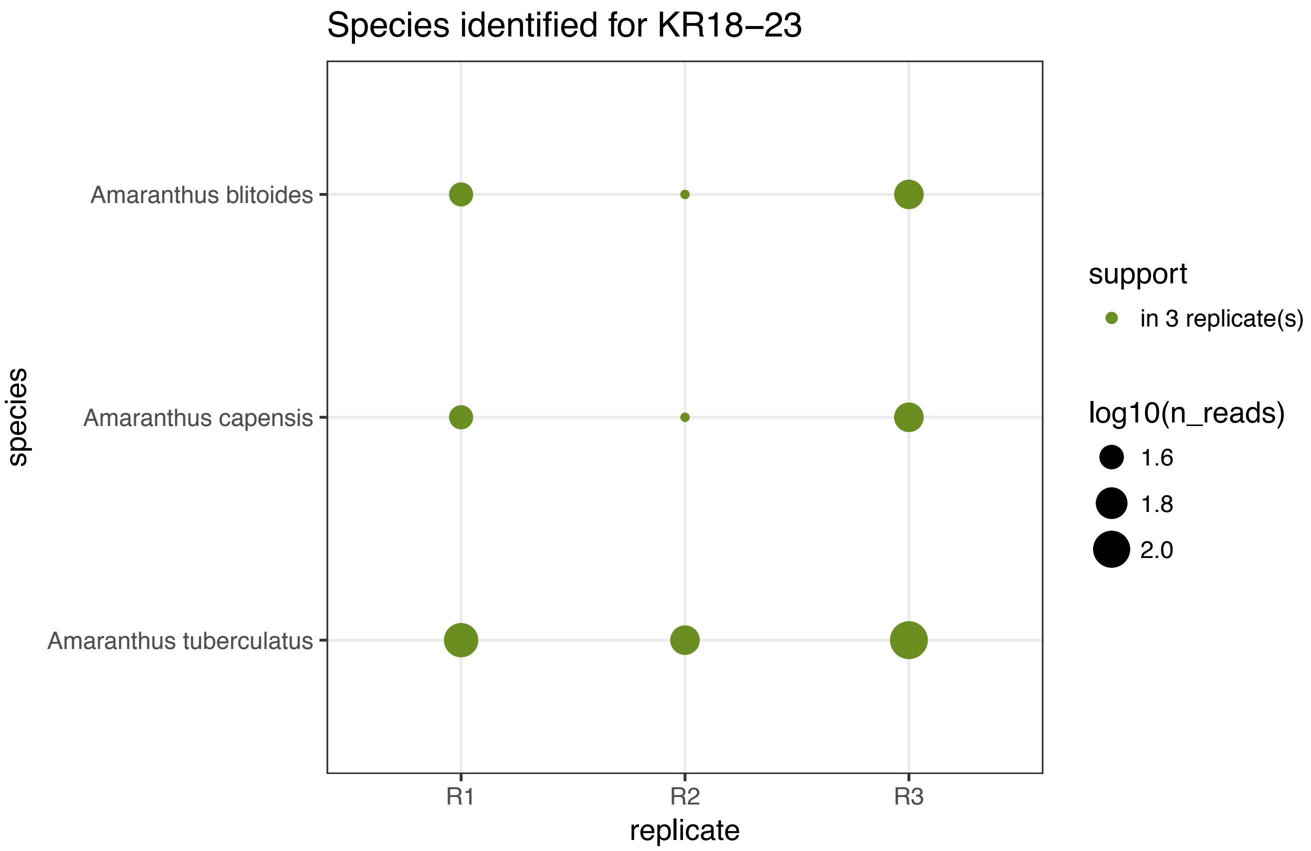

Table 21: Species identified in: KR18-23

species	support
Amaranthus blitoides	3
Amaranthus capensis	3
Amaranthus tuberculatus	3

Identifications for KR18-24

Label: Oregano.

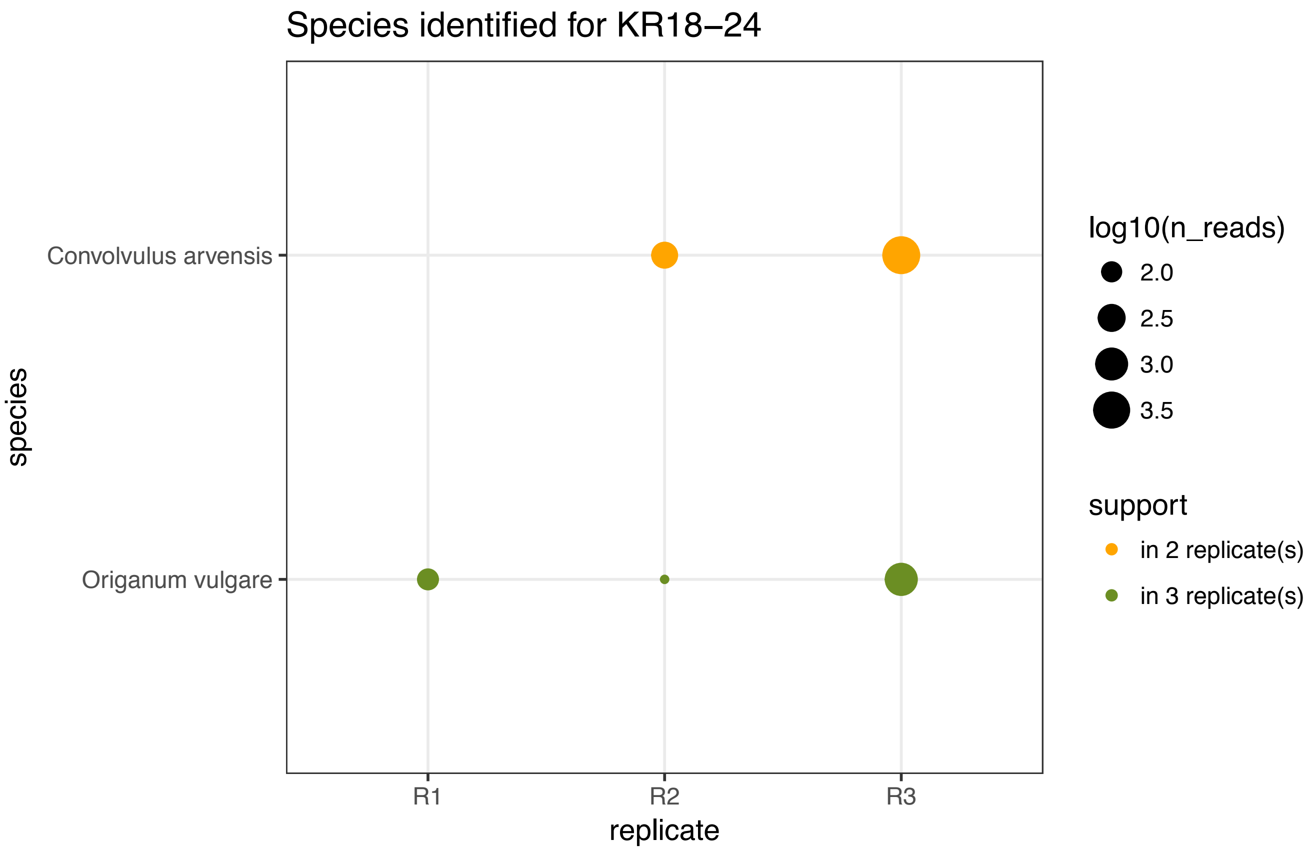

Table 22: Species identified in: KR18-24

species	support
Convolvulus arvensis	2
Origanum vulgare	3

Identifications for KR18-25

Label: Basil.

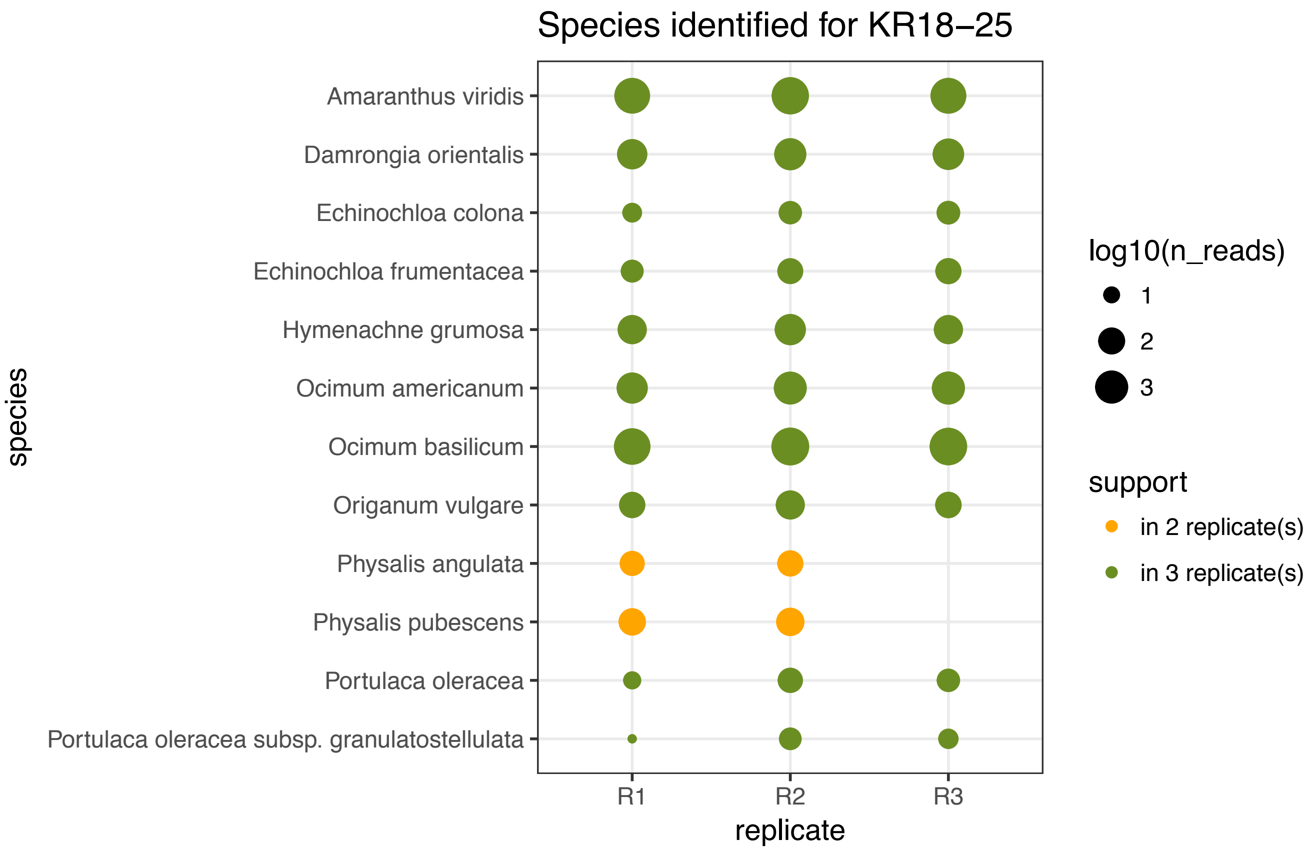

Table 23: Species identified in: KR18-25

species	support
Amaranthus viridis	3
Damrongia orientalis	3
Echinochloa colona	3
Echinochloa frumentacea	3
Hymenachne grumosa	3
Ocimum americanum	3
Ocimum basilicum	3
Origanum vulgare	3
Physalis angulata	2
Physalis pubescens	2
Portulaca oleracea	3
Portulaca oleracea subsp. granulatostellulata	3

Identifications for KR18-26

Label: Paprika.

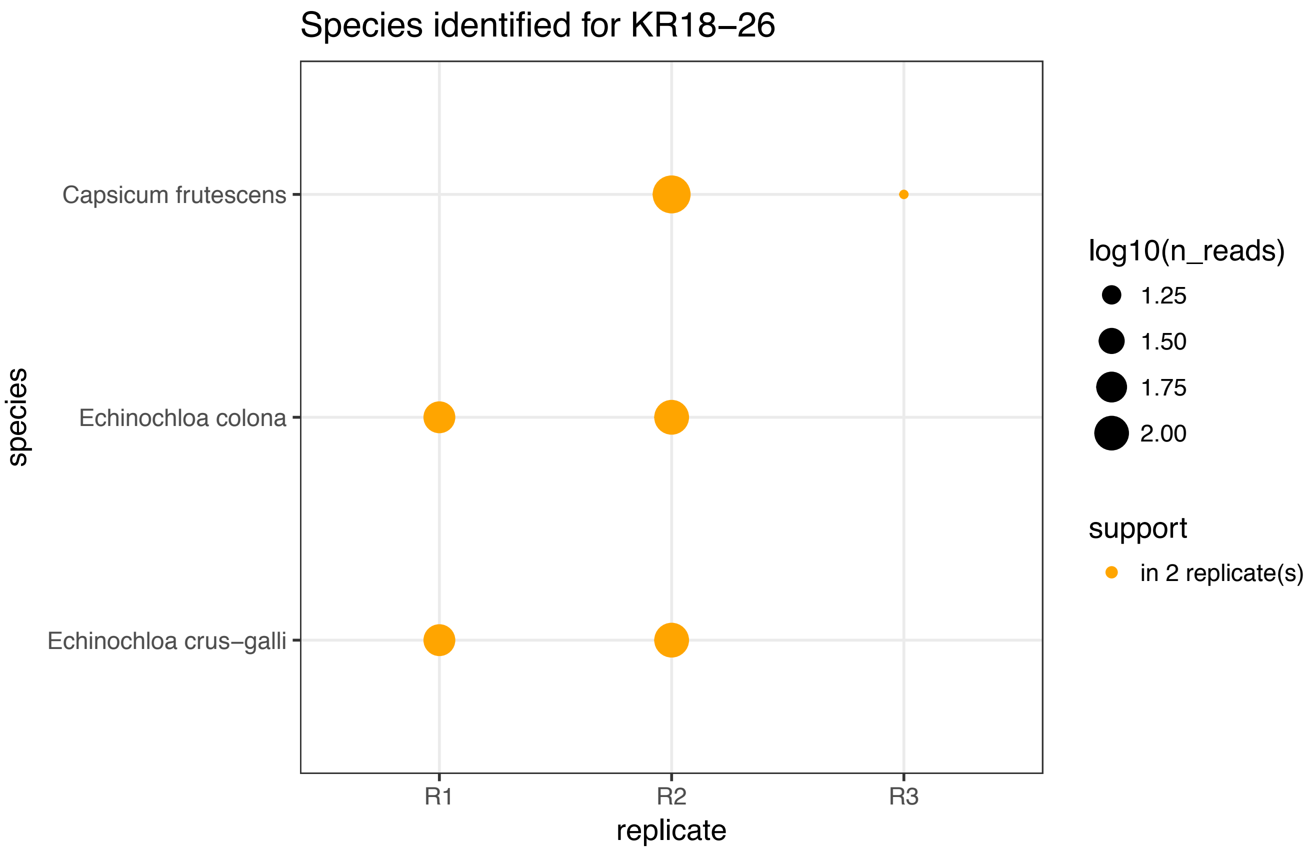

Table 24: Species identified in: KR18-26

species	support
Capsicum frutescens	2
Echinochloa colona	2
Echinochloa crus-galli	2

Identifications for KR18-27

Label: Oregano.

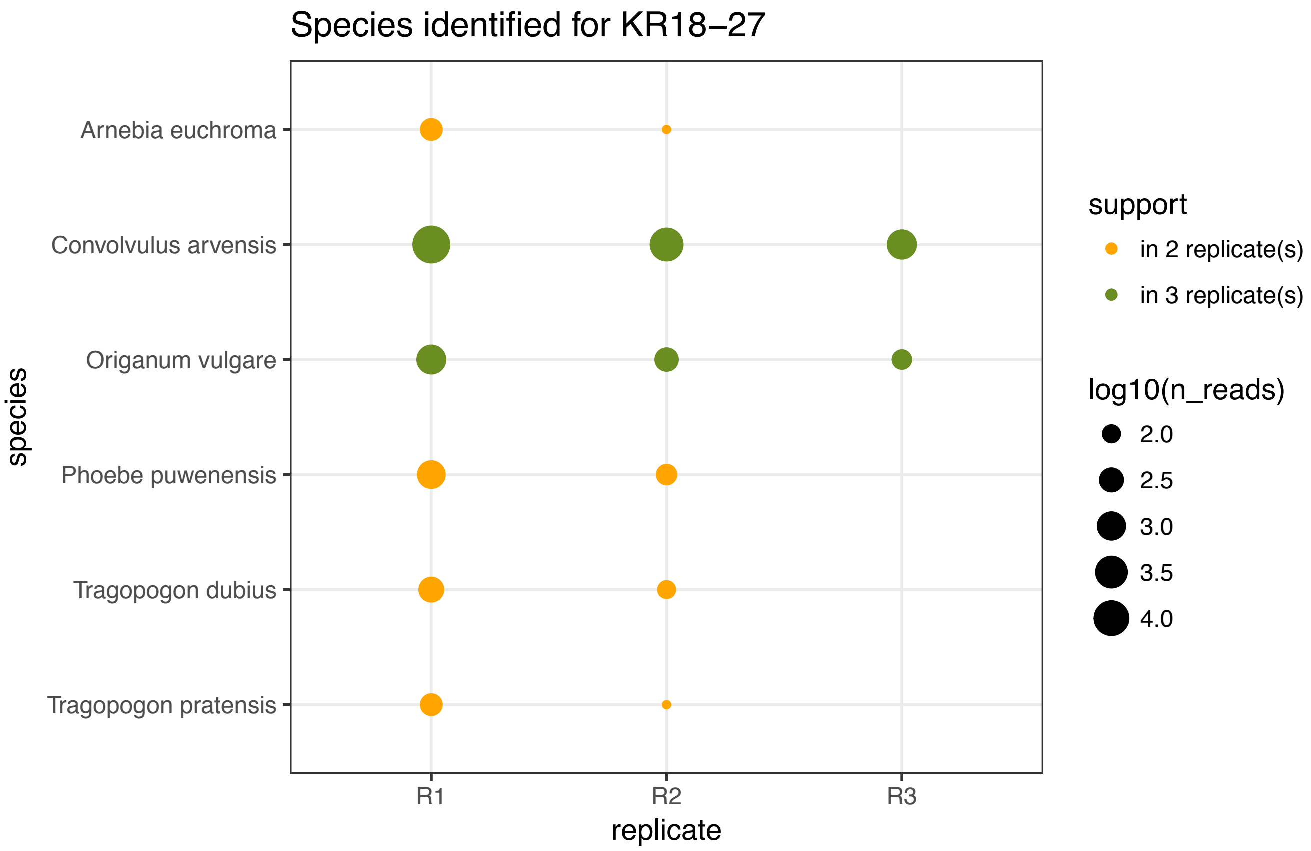

Table 25: Species identified in: KR18-27

species	support
Arnebia euchroma	2
Convolvulus arvensis	3
Origanum vulgare	3
Phoebe puwenensis	2
Tragopogon dubius	2
Tragopogon pratensis	2

Identifications for KR18-28

Label: Paprika.

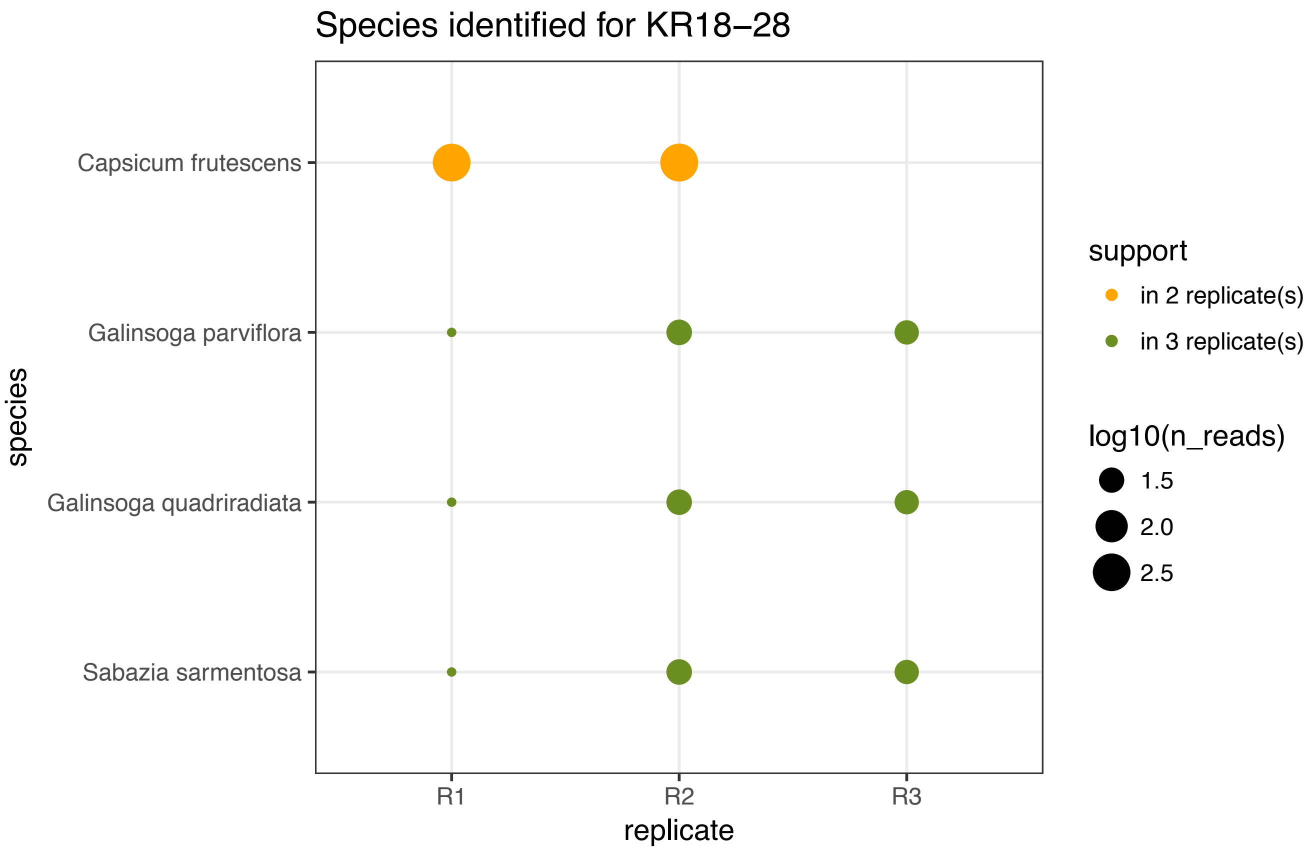

Table 26: Species identified in: KR18-28

species	support
Capsicum frutescens	2
Galinsoga parviflora	3
Galinsoga quadriradiata	3
Sabazia sarmentosa	3

Identifications for KR18-29

Label: Oregano.

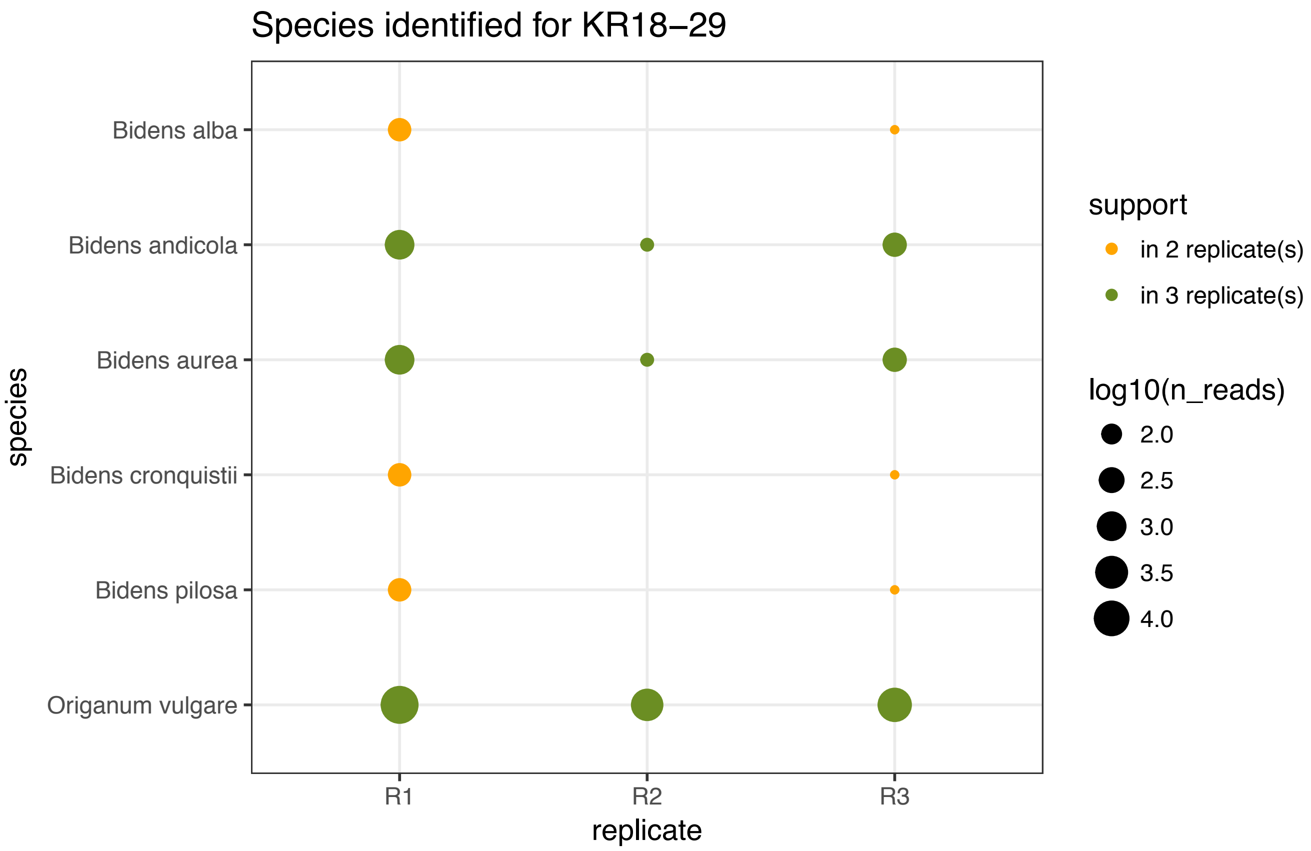

Table 27: Species identified in: KR18-29

species	support
Bidens alba	2
Bidens andicola	3
Bidens aurea	3
Bidens cronquistii	2
Bidens pilosa	2
Origanum vulgare	3

Identifications for KR18-30

Label: Basil.

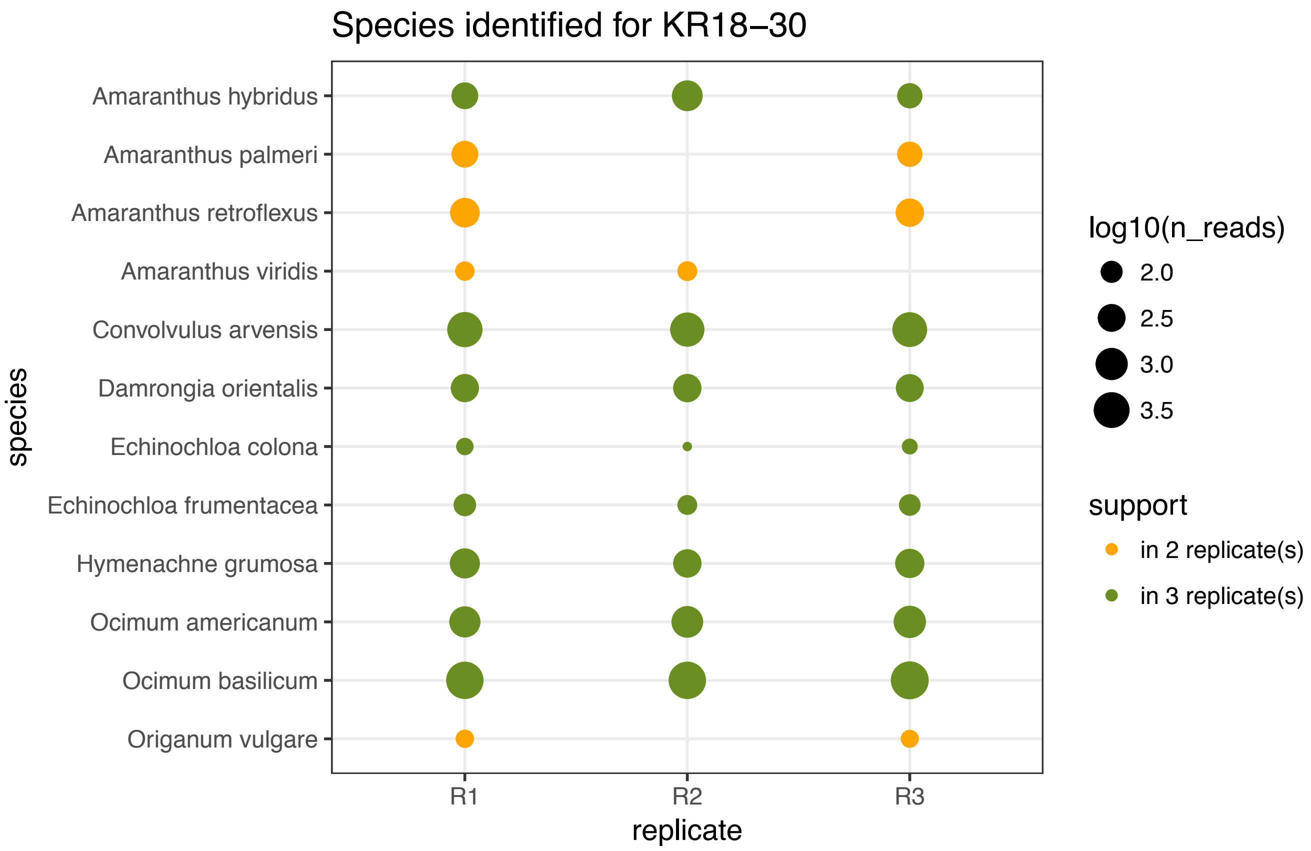

Table 28: Species identified in: KR18-30

species	support
Amaranthus hybridus	3
Amaranthus palmeri	2
Amaranthus retroflexus	2
Amaranthus viridis	2
Convolvulus arvensis	3
Damrongia orientalis	3
Echinochloa colona	3
Echinochloa frumentacea	3
Hymenachne grumosa	3
Ocimum americanum	3
Ocimum basilicum	3
Origanum vulgare	2

Identifications for KR18-31

Label: Oregano.

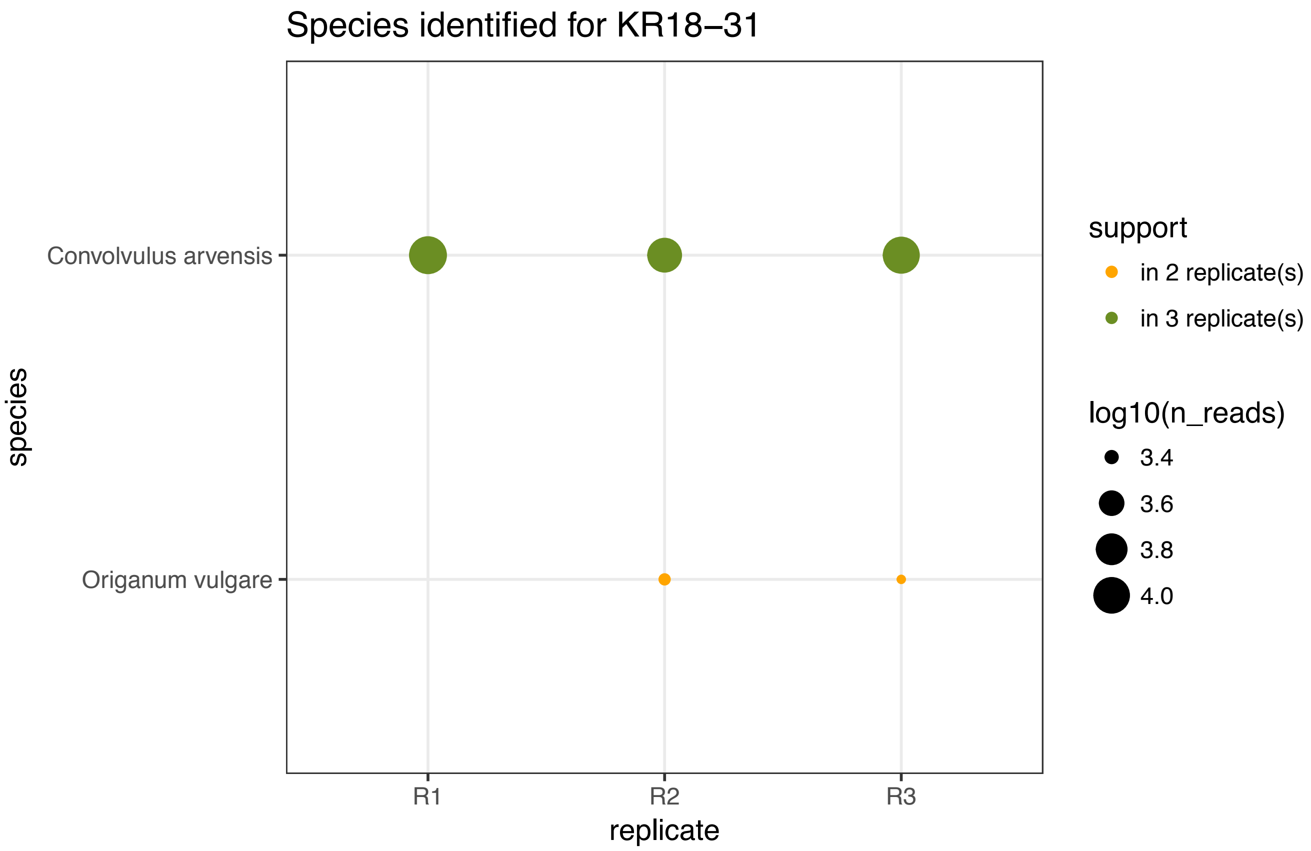

Table 29: Species identified in: KR18-31

species	support
Convolvulus arvensis	3
Origanum vulgare	2

Identifications for KR18-32

Label: Basil.

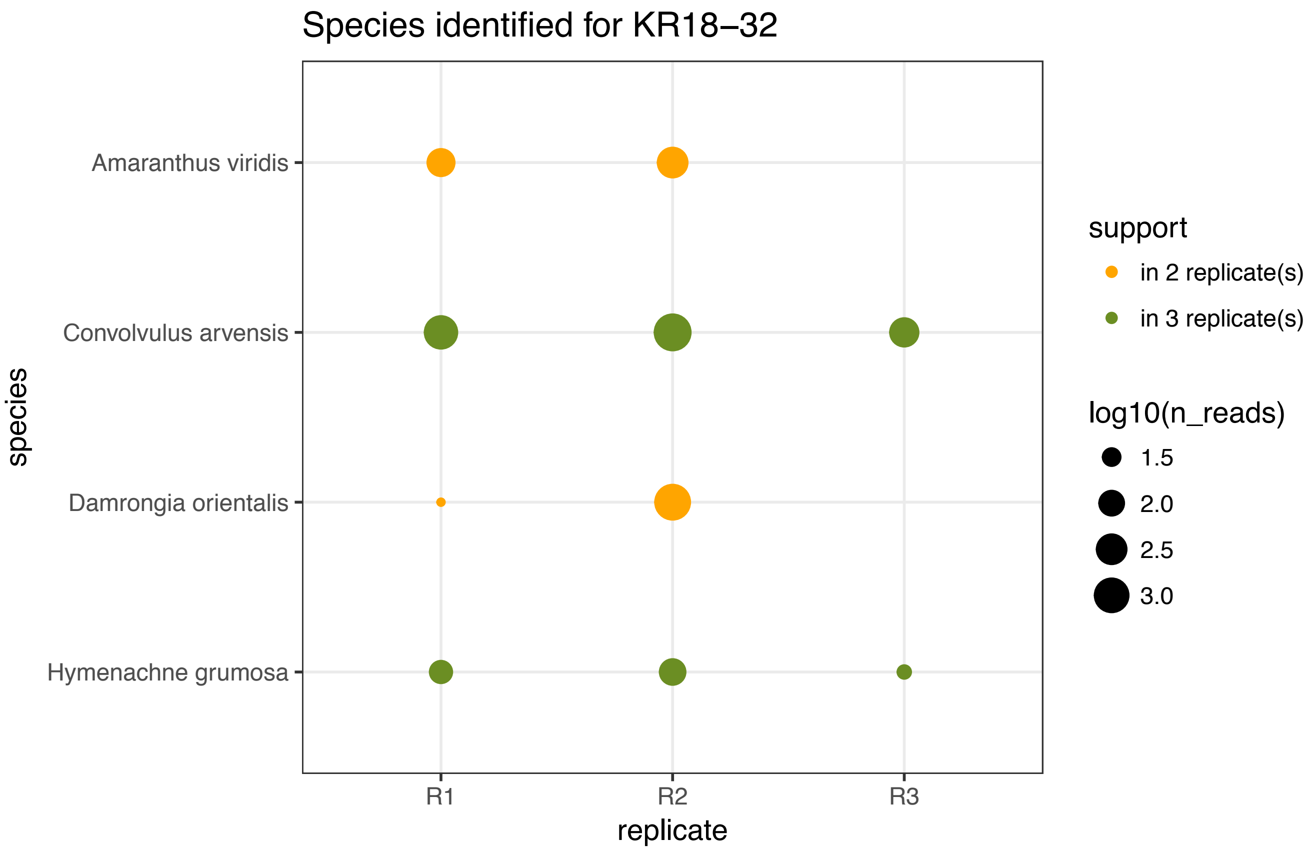

Table 30: Species identified in: KR18-32

species	support
Amaranthus viridis	2
Convolvulus arvensis	3
Damrongia orientalis	2
Hymenachne grumosa	3

Identifications for KR18-33

Label: Paprika. [1] “No species have been identified for KR18-33.”

Identifications for KR18-34

Label: Paprika.

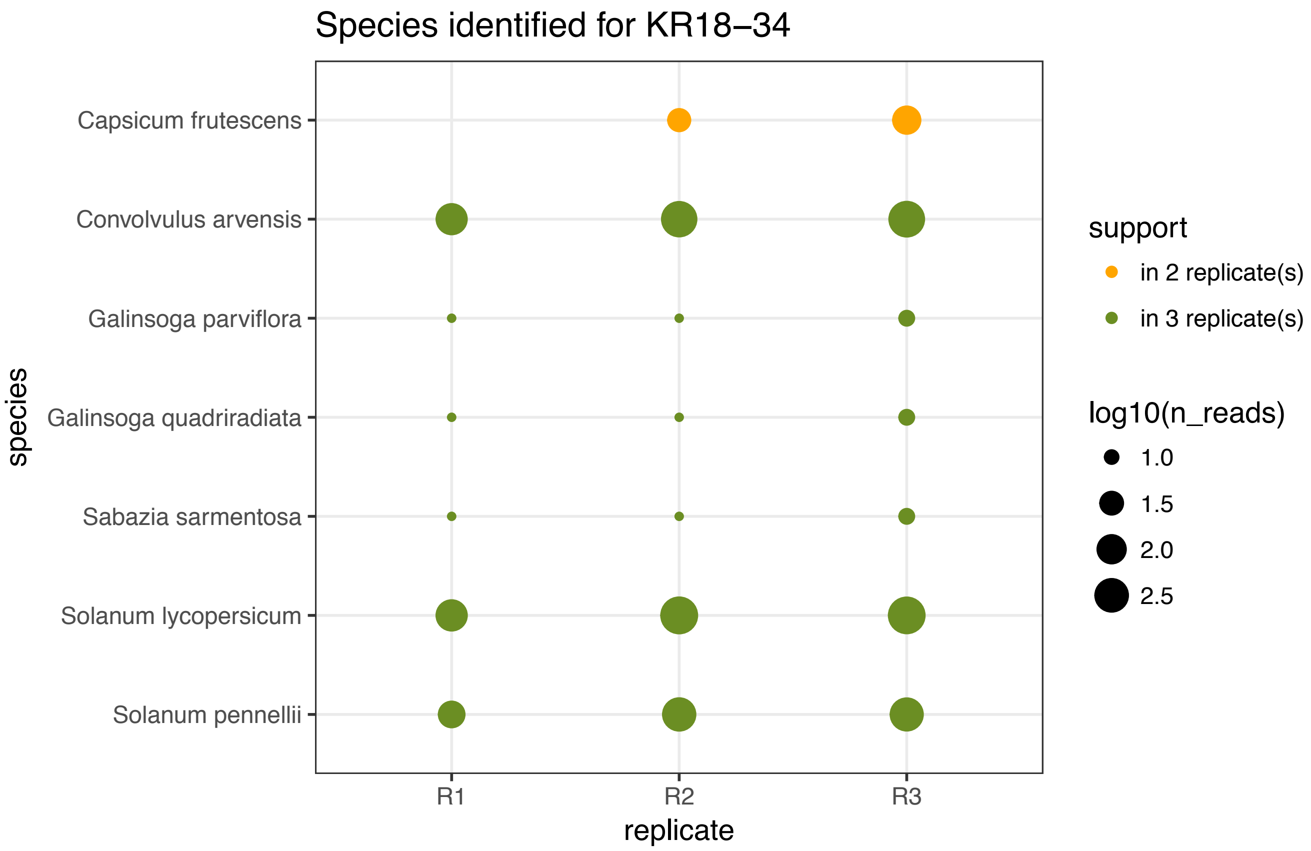

Table 31: Species identified in: KR18-34

species	support
Capsicum frutescens	2
Convolvulus arvensis	3
Galinsoga parviflora	3
Galinsoga quadriradiata	3
Sabazia sarmentosa	3
Solanum lycopersicum	3
Solanum pennellii	3

Identifications for KR18-35

Label: Oregano and Basil.

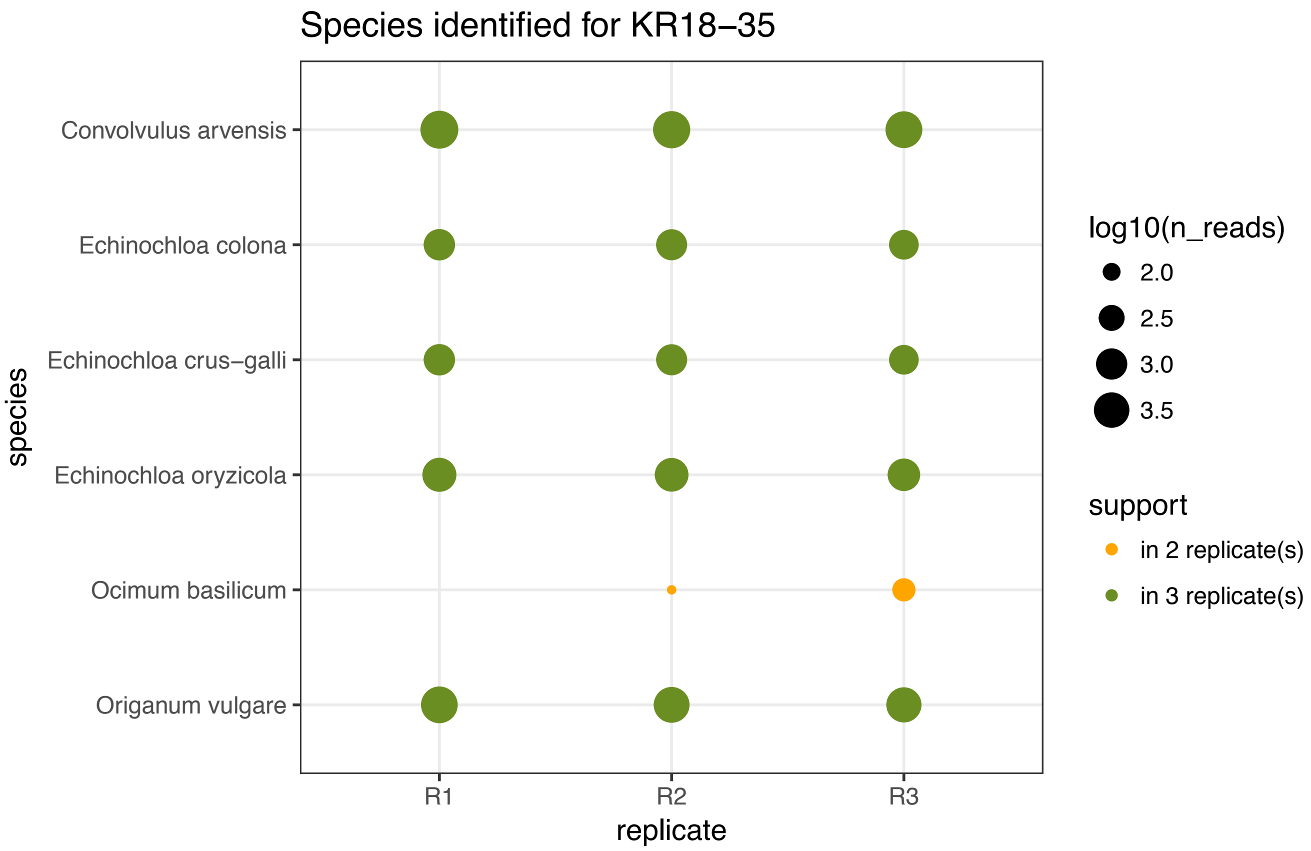

Table 32: Species identified in: KR18-35

species	support
Convolvulus arvensis	3
Echinochloa colona	3
Echinochloa crus-galli	3
Echinochloa oryzicola	3
Ocimum basilicum	2
Origanum vulgare	3

Identifications for KR18-36

Label: Oregano.

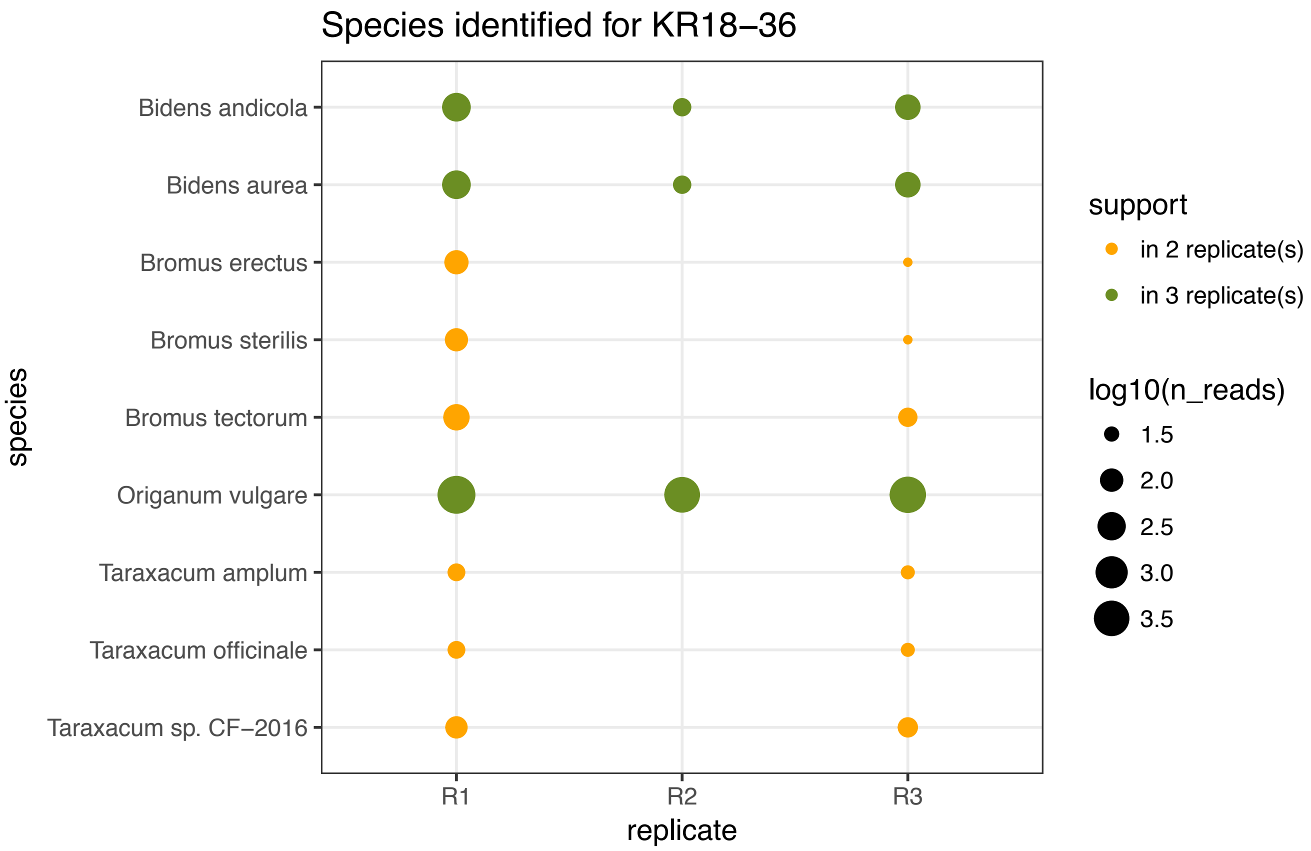

Table 33: Species identified in: KR18-36

species	support
Bidens andicola	3
Bidens aurea	3
Bromus erectus	2
Bromus sterilis	2
Bromus tectorum	2
Origanum vulgare	3
Taraxacum amplum	2
Taraxacum officinale	2
Taraxacum sp. CF-2016	2

Identifications for KR18-37

Label: Basil.

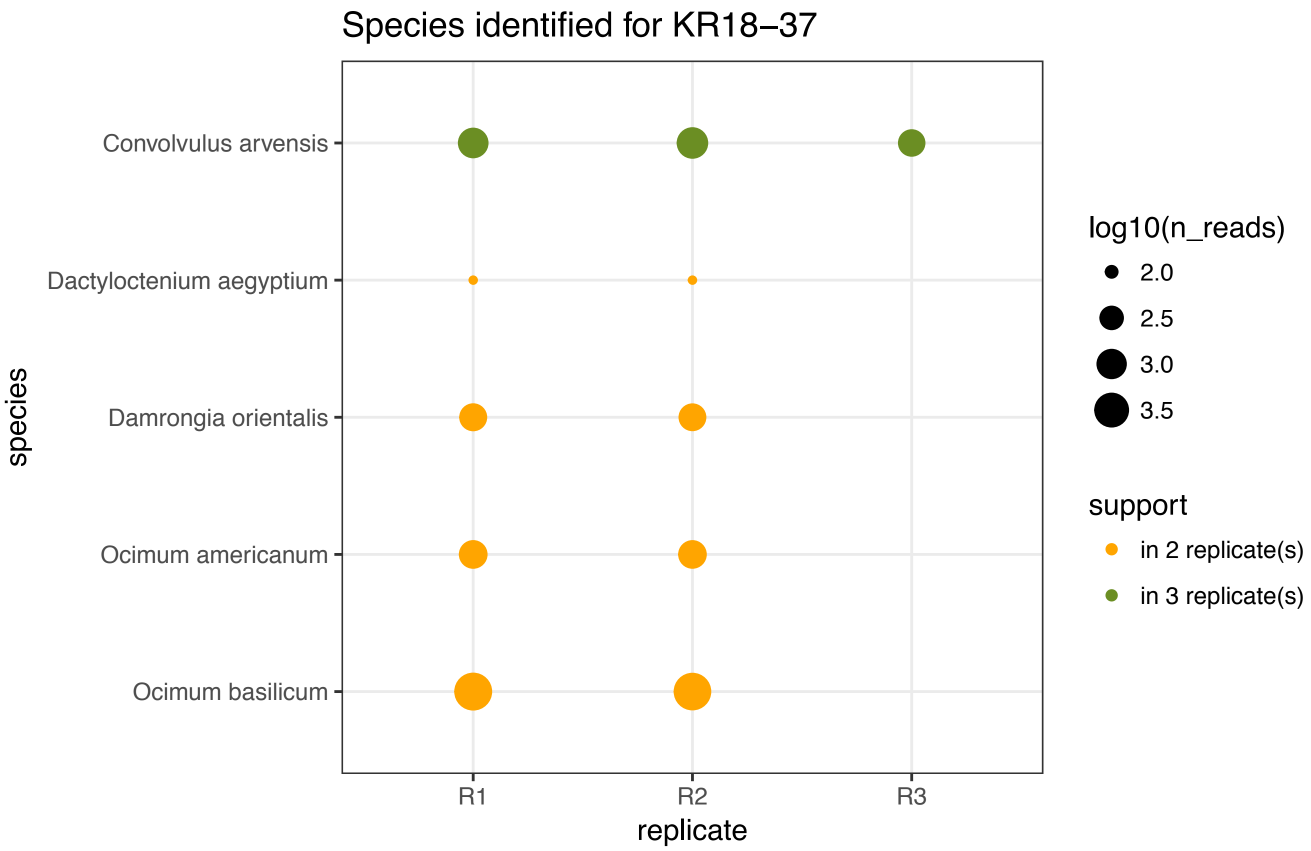

Table 34: Species identified in: KR18-37

species	support
Convolvulus arvensis	3
Dactyloctenium aegyptium	2
Damrongia orientalis	2
Ocimum americanum	2
Ocimum basilicum	2

Identifications for KR18-38

Label: Paprika.

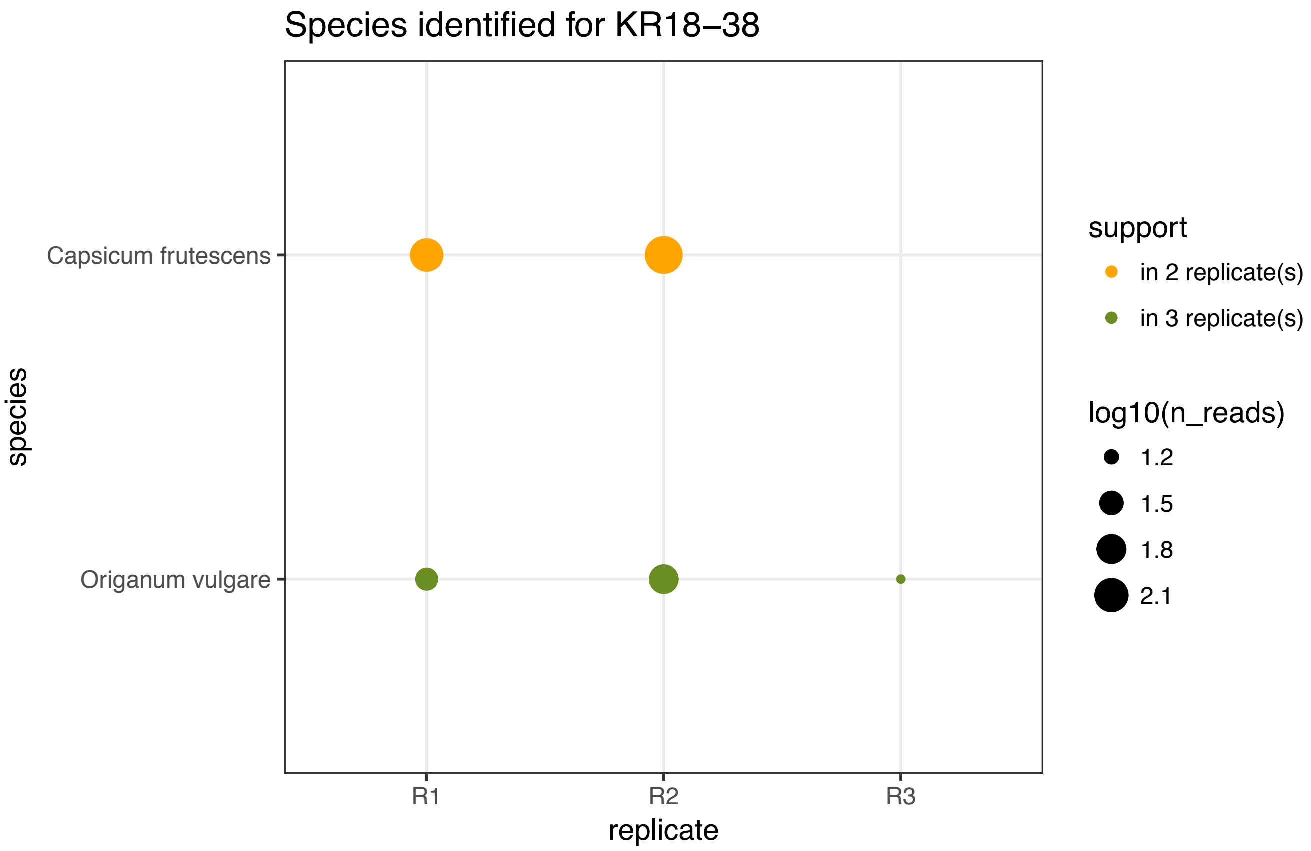

Table 35: Species identified in: KR18-38

species	support
Capsicum frutescens	2
Origanum vulgare	3

Identifications for KR18-39

Label: Basil.

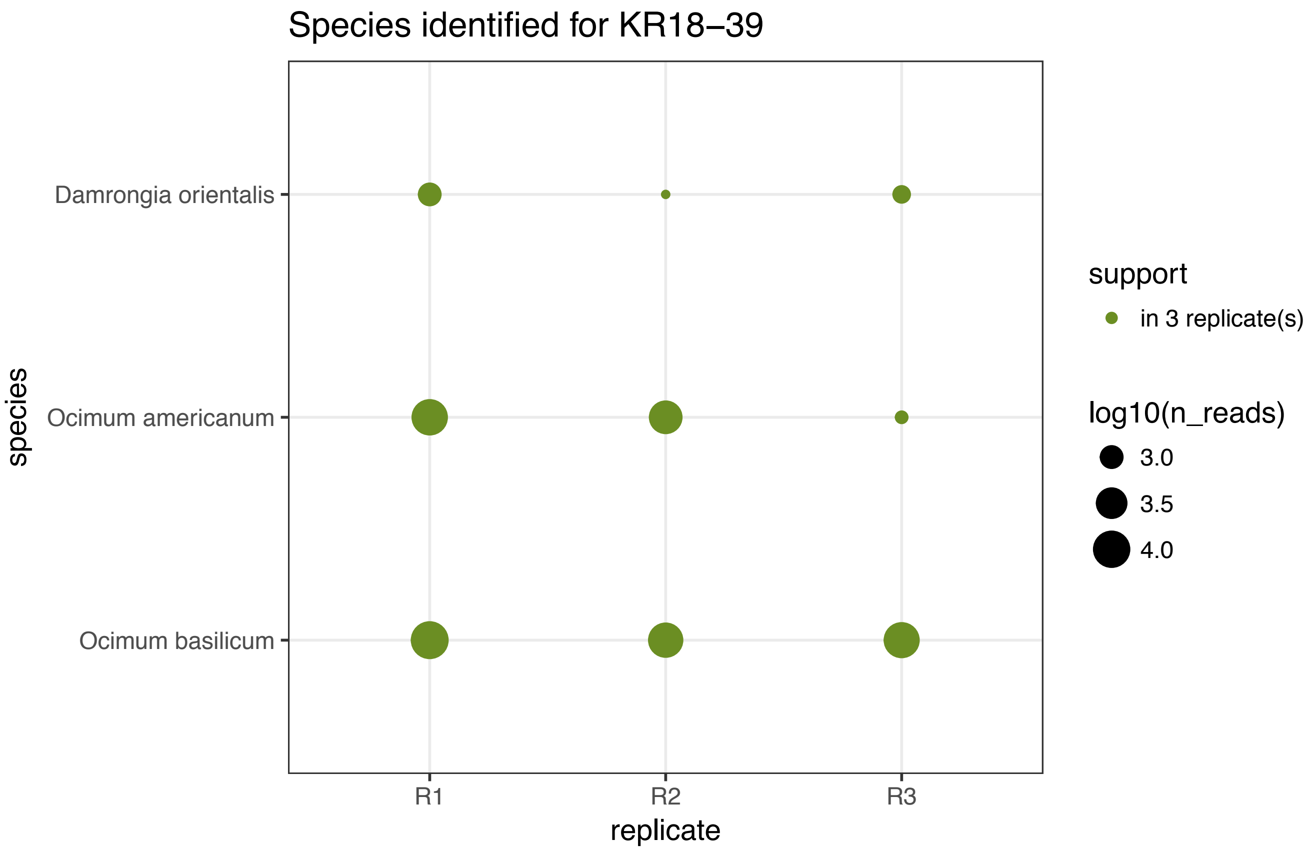

Table 36: Species identified in: KR18-39

species	support
Damrongia orientalis	3
Ocimum americanum	3
Ocimum basilicum	3

Identifications for KR18-40

Label: Basil.

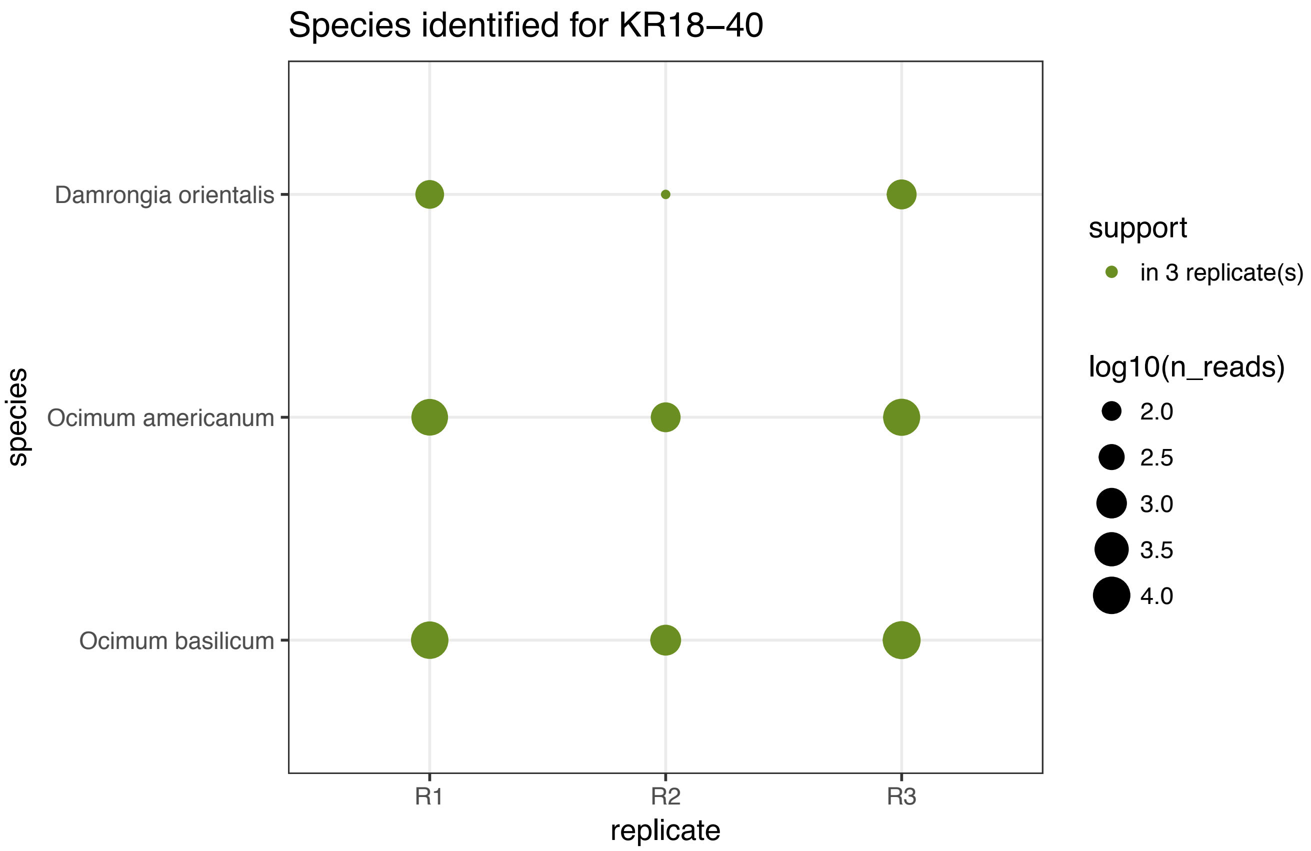

Table 37: Species identified in: KR18-40

species	support
Damrongia orientalis	3
Ocimum americanum	3
Ocimum basilicum	3

Identifications for KR18-41

Label: Oregano.

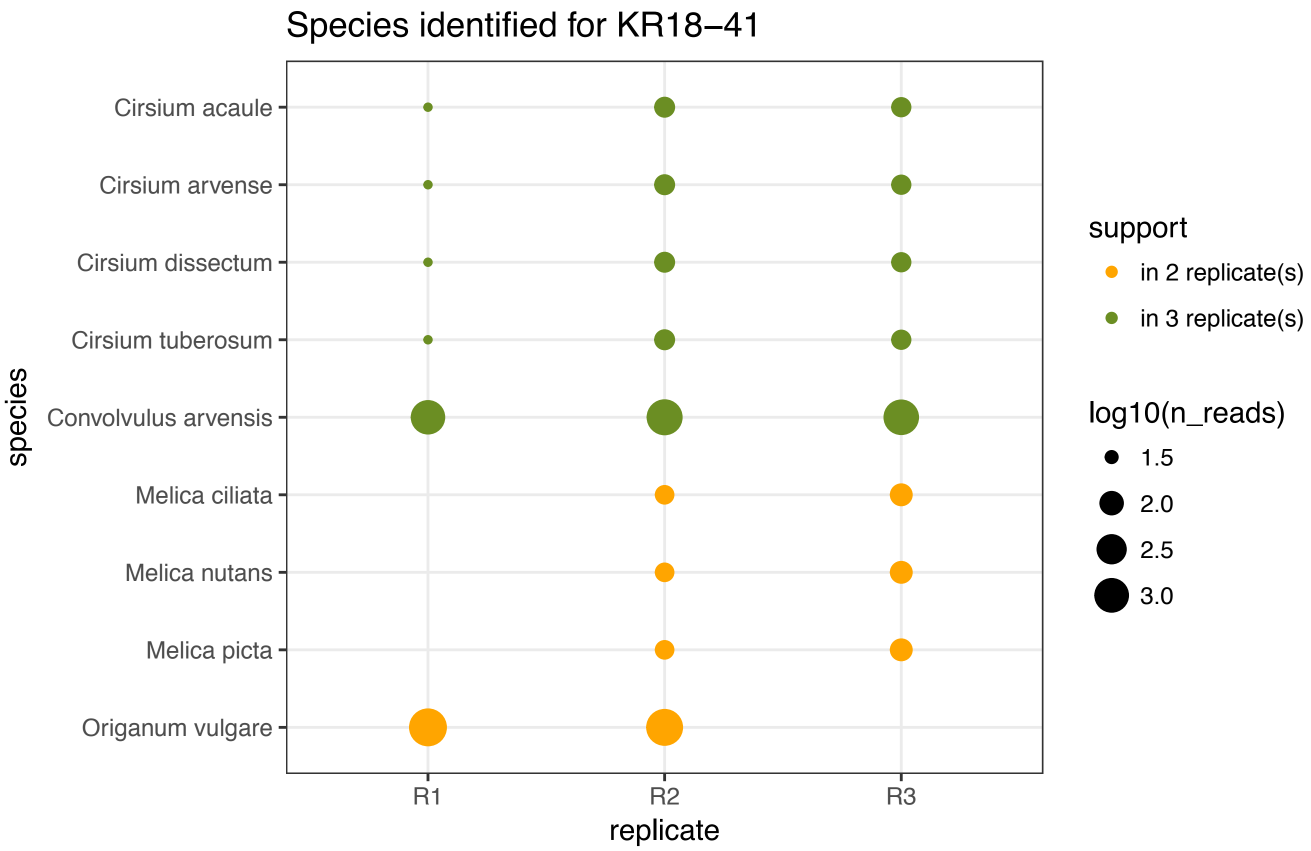

Table 38: Species identified in: KR18-41

species	support
Cirsium acaule	3
Cirsium arvense	3
Cirsium dissectum	3
Cirsium tuberosum	3
Convolvulus arvensis	3
Melica ciliata	2
Melica nutans	2
Melica picta	2
Origanum vulgare	2

Identifications for KR18-42

Label: Oregano.

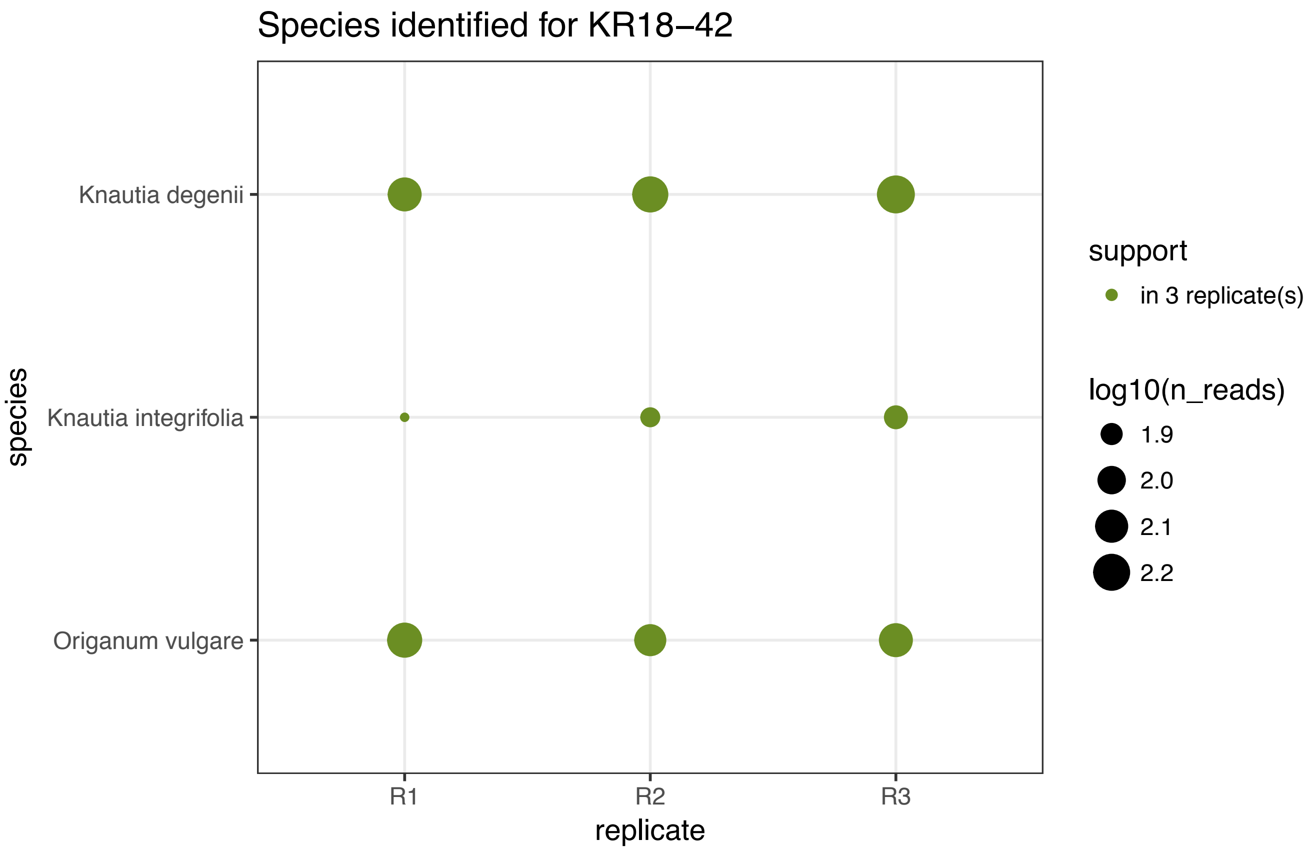

Table 39: Species identified in: KR18-42

species	support
Knautia degenii	3
Knautia integrifolia	3
Origanum vulgare	3

Identifications for KR18-43

Label: Basil.

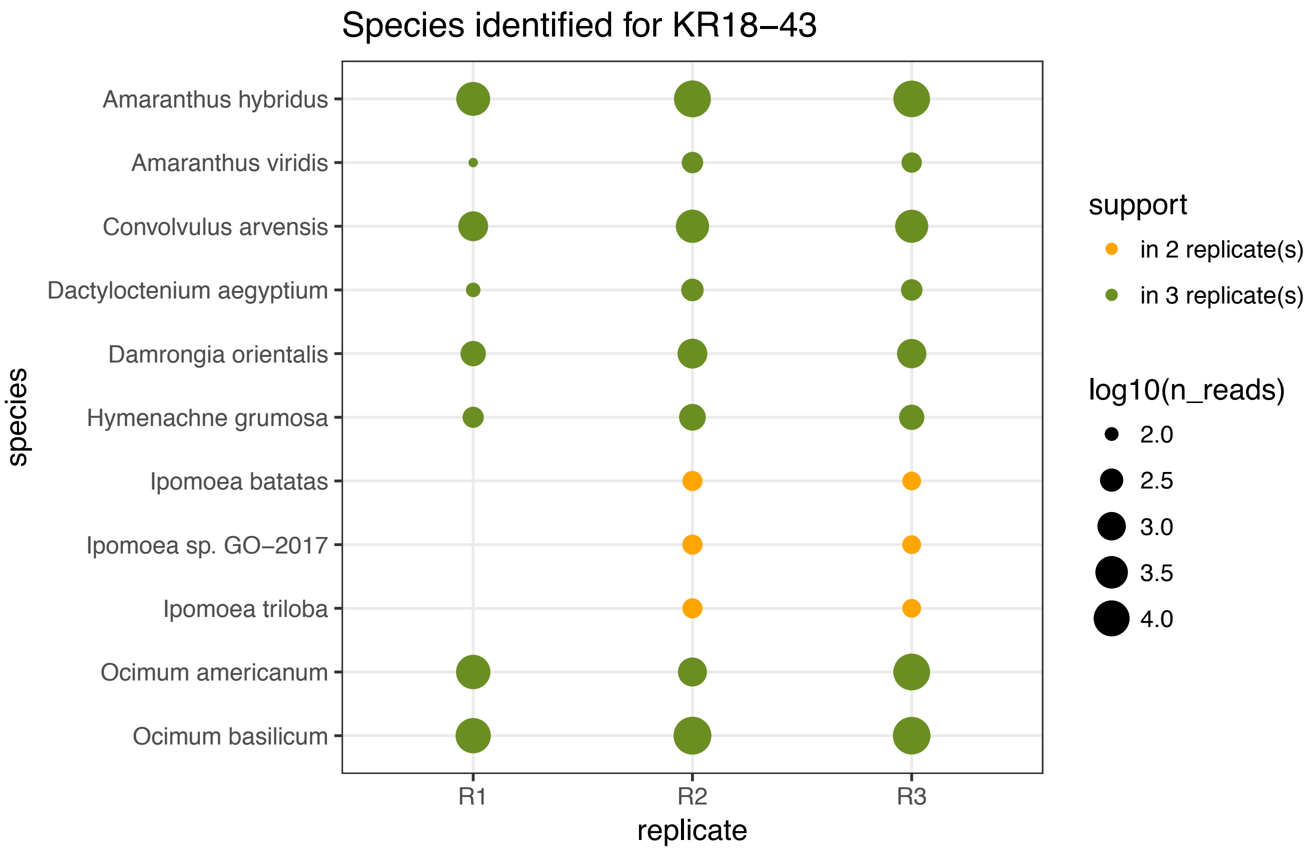

Table 40: Species identified in: KR18-43

species	support
Amaranthus hybridus	3
Amaranthus viridis	3
Convolvulus arvensis	3
Dactyloctenium aegyptium	3
Damrongia orientalis	3
Hymenachne grumosa	3
Ipomoea batatas	2
Ipomoea sp. GO-2017	2
Ipomoea triloba	2
Ocimum americanum	3
Ocimum basilicum	3

Identifications for KR18-44

Label: Oregano.

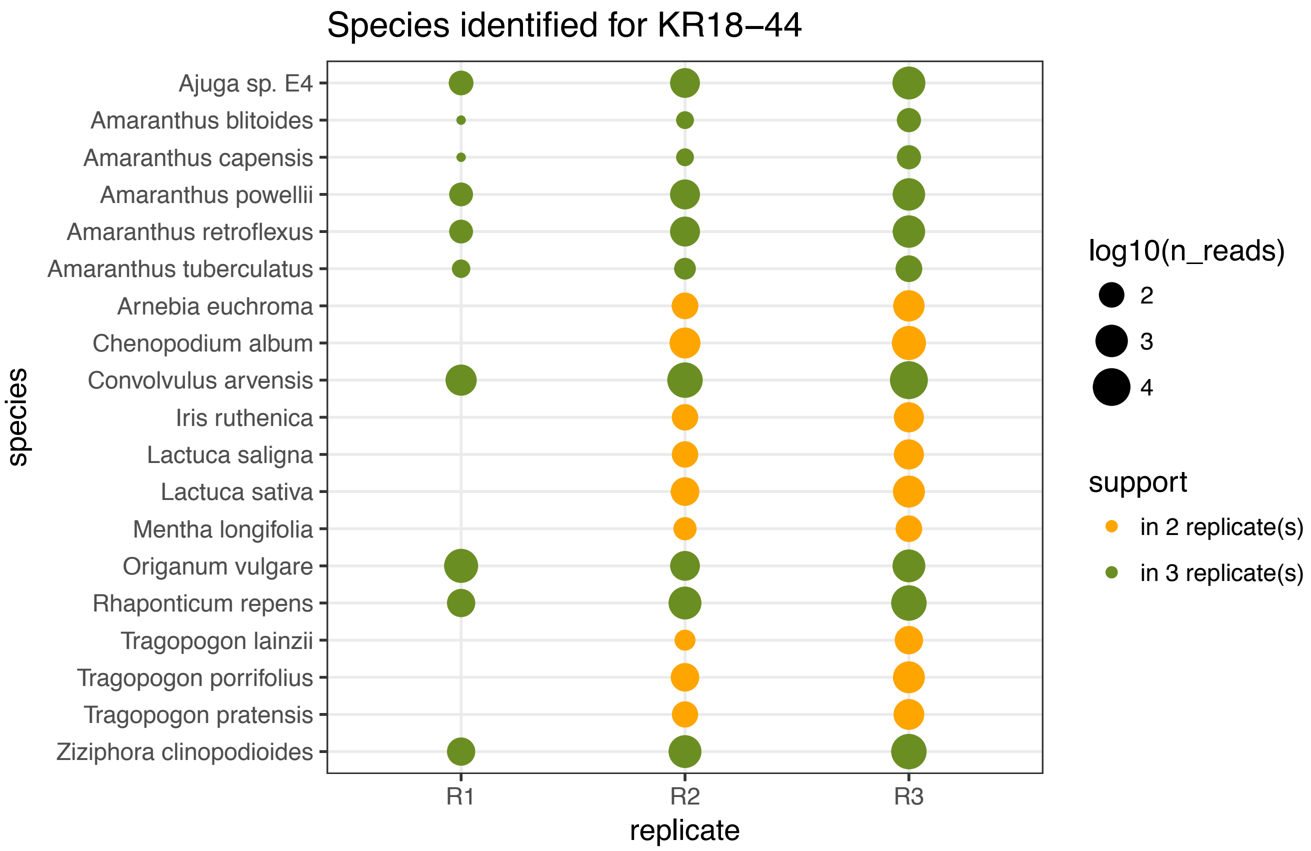

Table 41: Species identified in: KR18-44

species	support
Ajuga sp. E4	3
Amaranthus blitoides	3
Amaranthus capensis	3
Amaranthus powellii	3
Amaranthus retroflexus	3
Amaranthus tuberculatus	3
Arnebia euchroma	2
Chenopodium album	2
Convolvulus arvensis	3
Iris ruthenica	2
Lactuca saligna	2
Lactuca sativa	2
Mentha longifolia	2
Origanum vulgare	3
Rhaponticum repens	3
Tragopogon lainzii	2
Tragopogon porrifolius	2
Tragopogon pratensis	2
Ziziphora clinopodioides	3

Identifications for KR18-45

Label: Paprika.

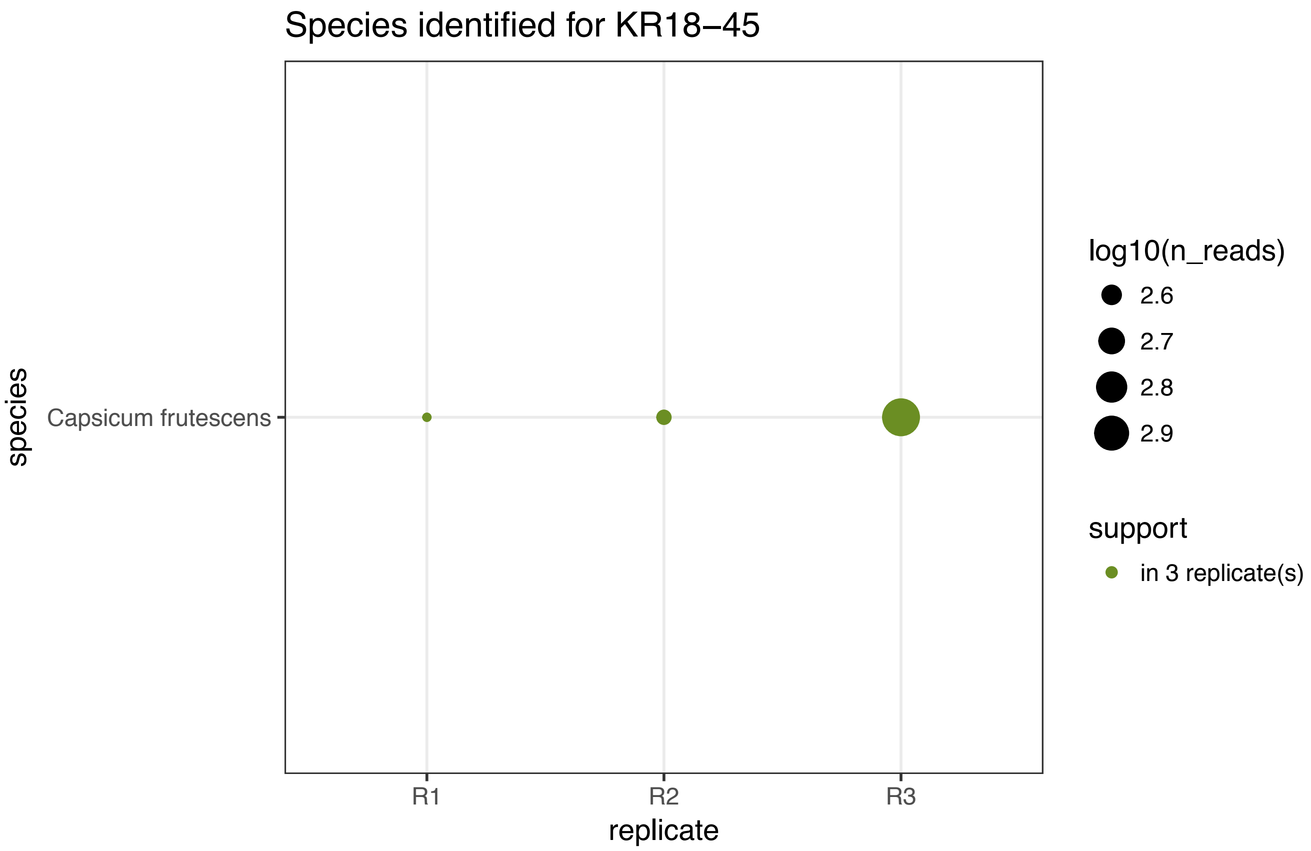

Table 42: Species identified in: KR18-45

species	support
Capsicum frutescens	3

Identifications for KR18-46

Label: Paprika. [1] “No species have been identified for KR18-46.”

Identifications for KR18-47

Label: Oregano.

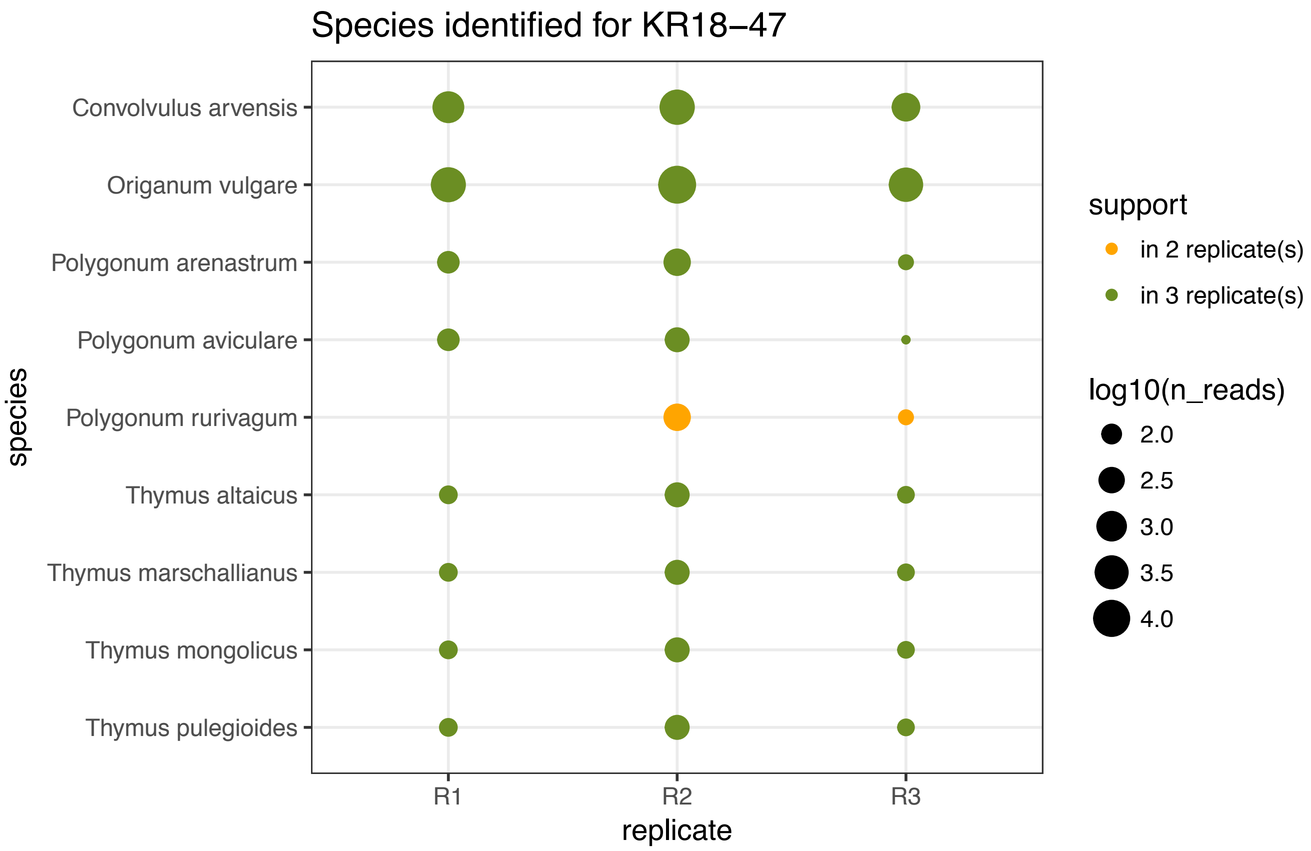

Table 43: Species identified in: KR18-47

species	support
Convolvulus arvensis	3
Origanum vulgare	3
Polygonum arenastrum	3
Polygonum aviculare	3
Polygonum rurivagum	2
Thymus altaicus	3
Thymus marschallianus	3
Thymus mongolicus	3
Thymus pulegioides	3

Identifications for KR18-48

Label: Oregano.

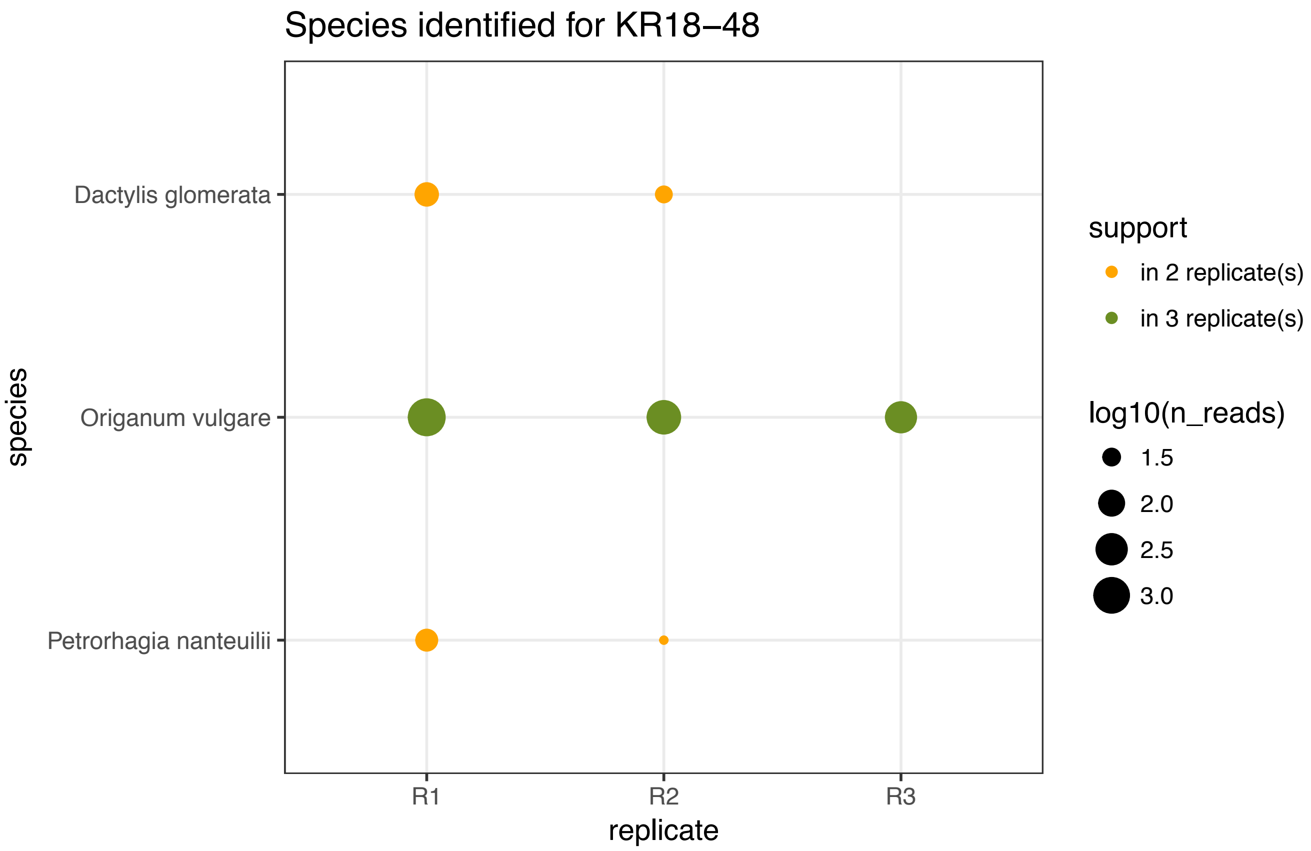

Table 44: Species identified in: KR18-48

species	support
Dactylis glomerata	2
Origanum vulgare	3
Petrorhagia nanteuilii	2

Identifications for KR18-49

Label: Basil.

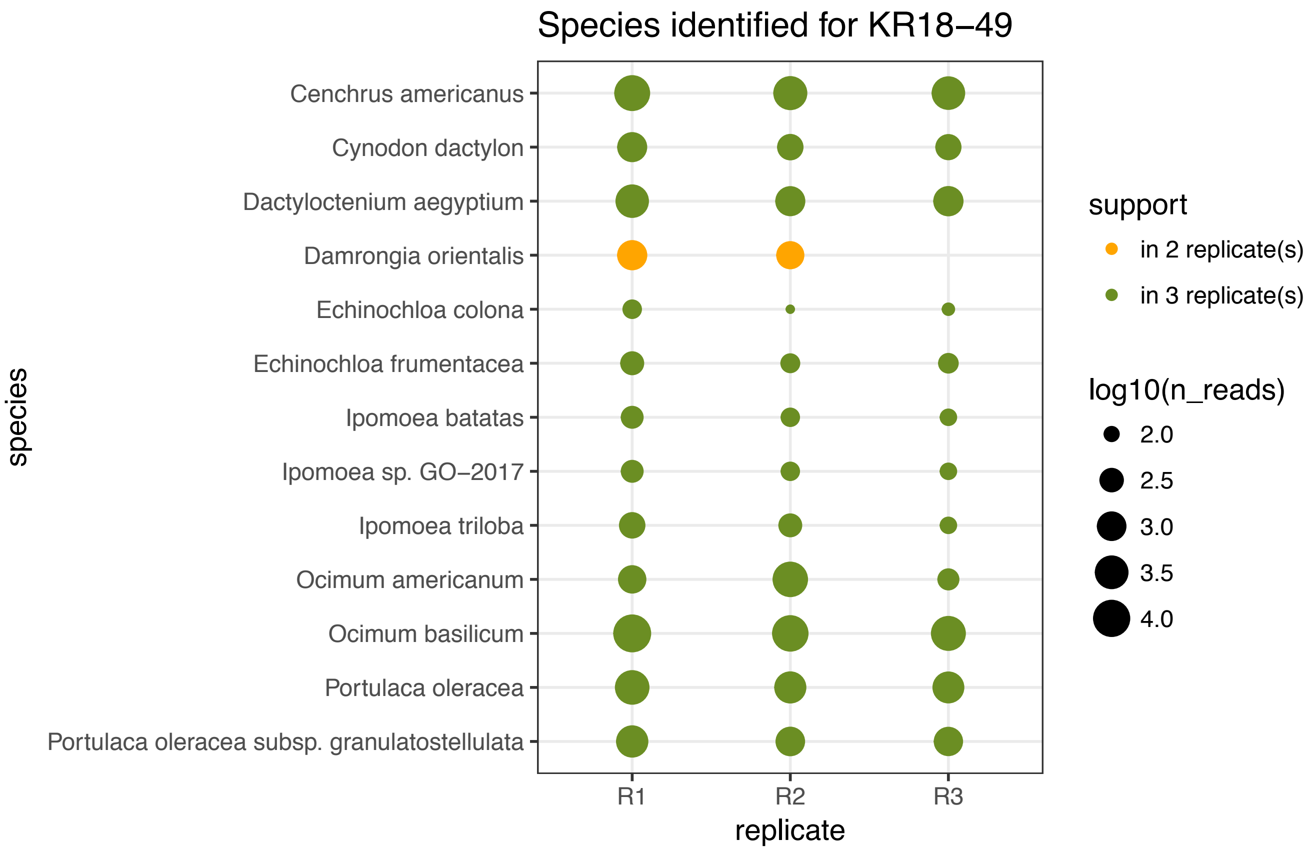

Table 45: Species identified in: KR18-49

species	support
Cenchrus americanus	3
Cynodon dactylon	3
Dactyloctenium aegyptium	3
Damrongia orientalis	2
Echinochloa colona	3
Echinochloa frumentacea	3
Ipomoea batatas	3
Ipomoea sp. GO-2017	3
Ipomoea triloba	3
Ocimum americanum	3
Ocimum basilicum	3
Portulaca oleracea	3
Portulaca oleracea subsp. granulatostellulata	3

Identifications for KR18-50

Label: Oregano.

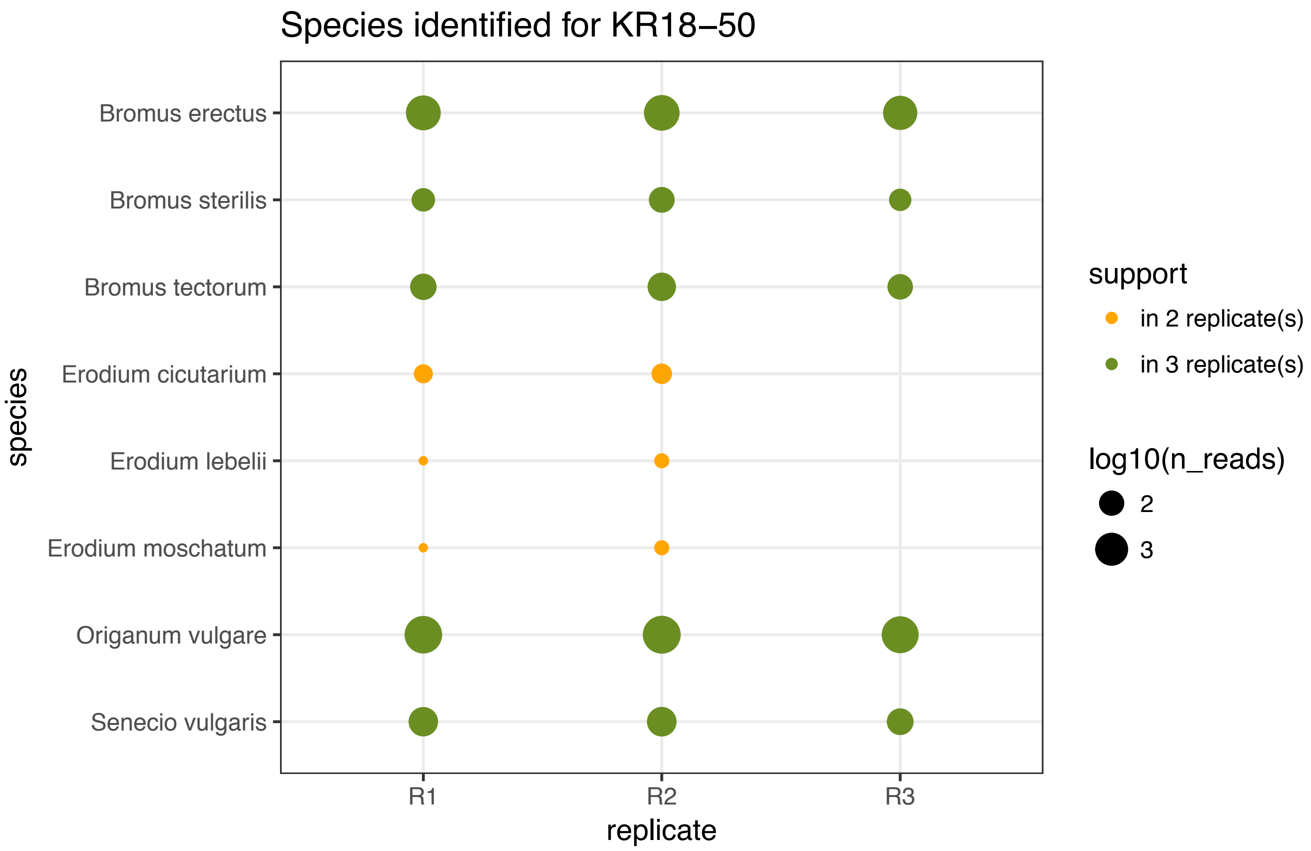

Table 46: Species identified in: KR18-50

species	support
Bromus erectus	3
Bromus sterilis	3
Bromus tectorum	3
Erodium cicutarium	2
Erodium lebelii	2
Erodium moschatum	2
Origanum vulgare	3
Senecio vulgaris	3

Identifications for KR18-51

Label: Basil.

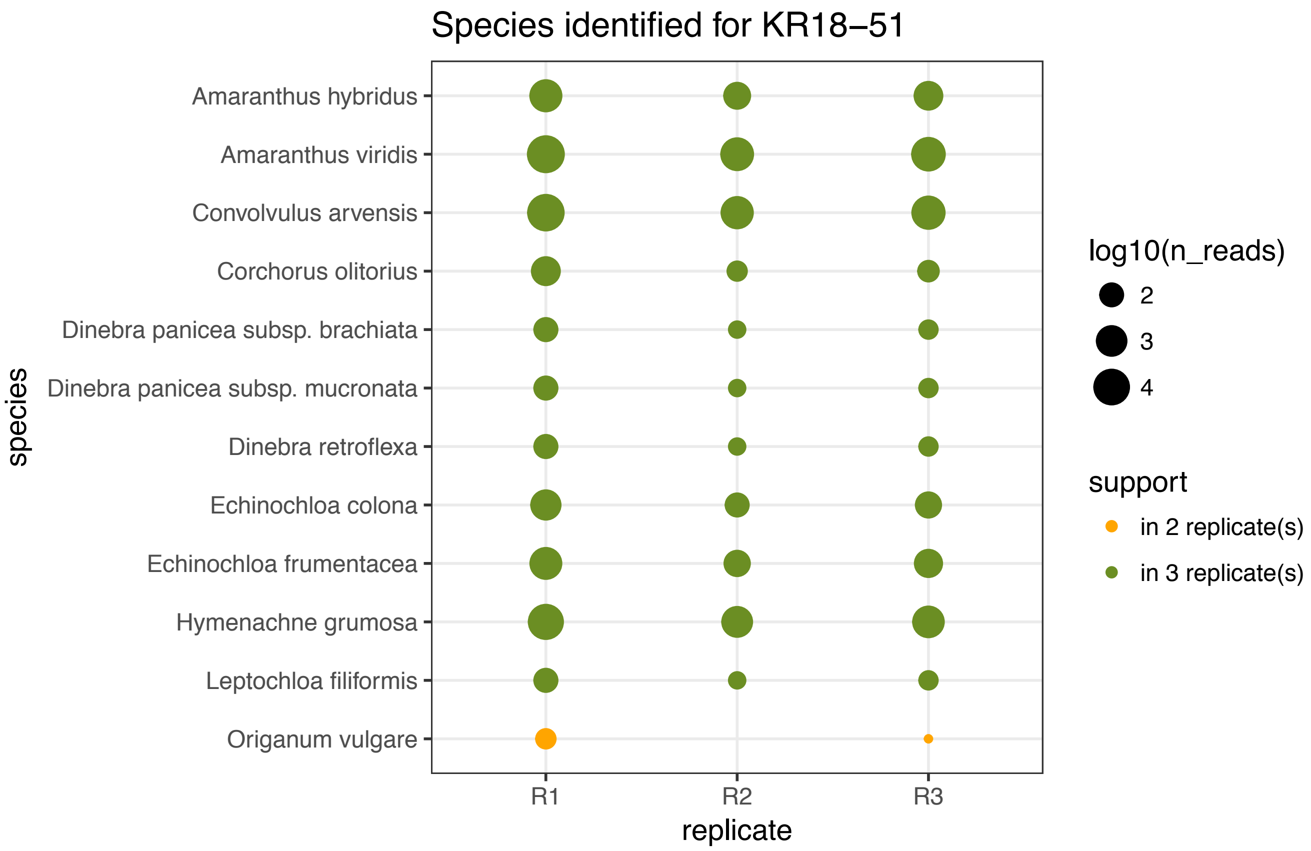

Table 47: Species identified in: KR18-51

species	support
Amaranthus hybridus	3
Amaranthus viridis	3
Convolvulus arvensis	3
Corchorus olitorius	3
Dinebra panicea subsp. brachiata	3
Dinebra panicea subsp. mucronata	3
Dinebra retroflexa	3
Echinochloa colona	3
Echinochloa frumentacea	3
Hymenachne grumosa	3
Leptochloa filiformis	3
Origanum vulgare	2

Identifications for KR18-52

Label: Paprika. [1] “No species have been identified for KR18-52.”

Identifications for KR18-53

Label: Paprika. [1] “No species have been identified for KR18-53.”

Identifications for KR18-54

Label: Paprika. [1] “No species have been identified for KR18-54.”

Identifications for KR18-55

Label: Oregano.

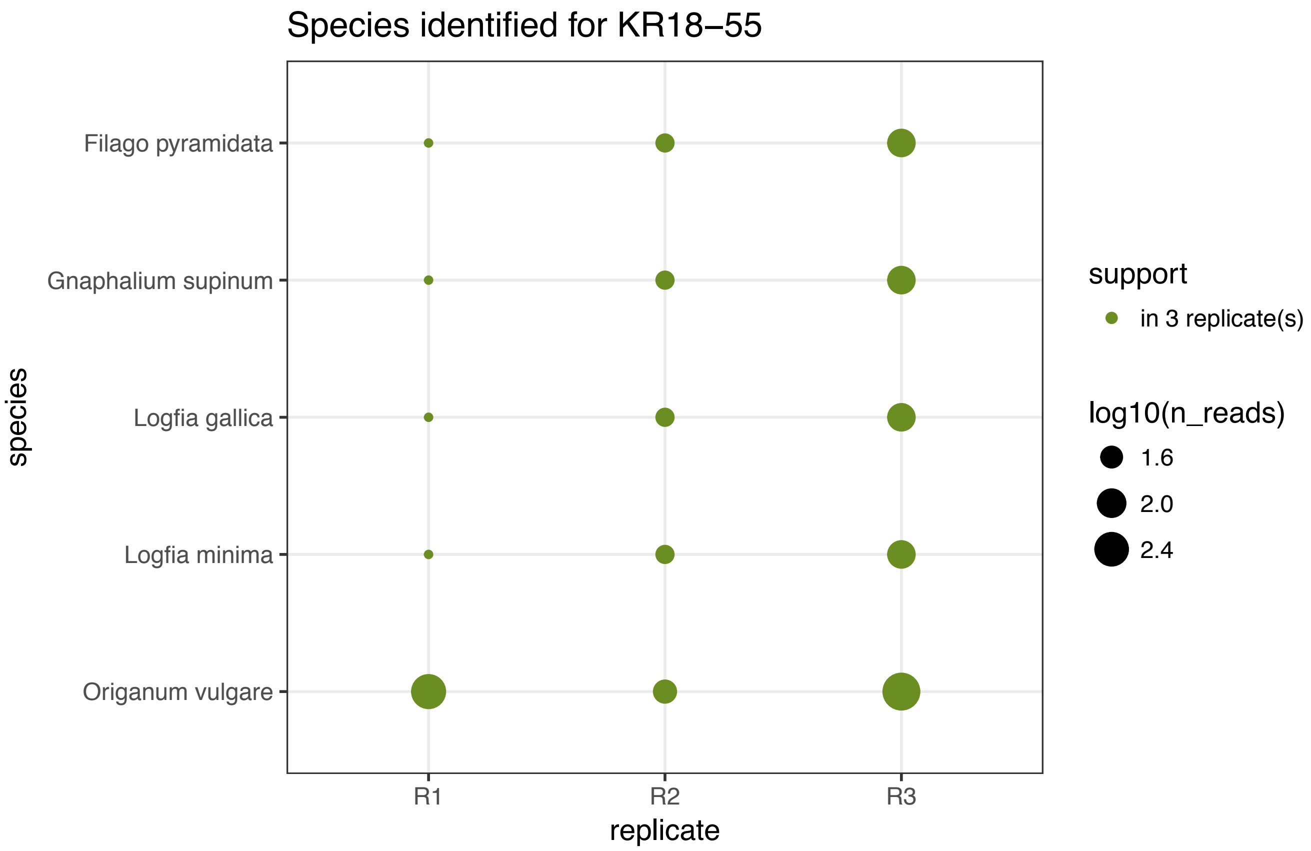

Table 48: Species identified in: KR18-55

species	support
Filago pyramidata	3
Gnaphalium supinum	3
Logfia gallica	3
Logfia minima	3
Origanum vulgare	3

Identifications for KR18-56

Label: Basil.

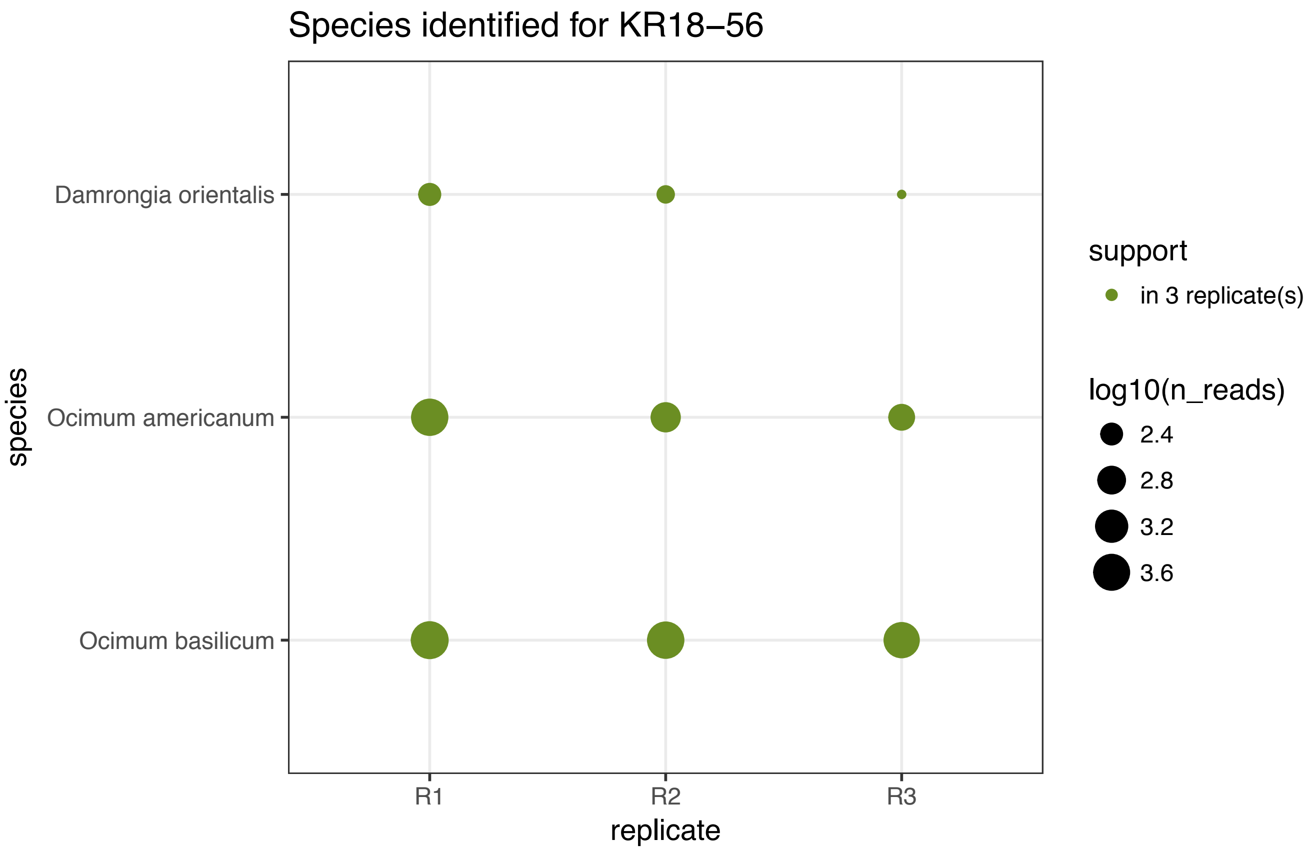

Table 49: Species identified in: KR18-56

species	support
Damrongia orientalis	3
Ocimum americanum	3
Ocimum basilicum	3

Identifications for KR18-57

Label: Oregano.

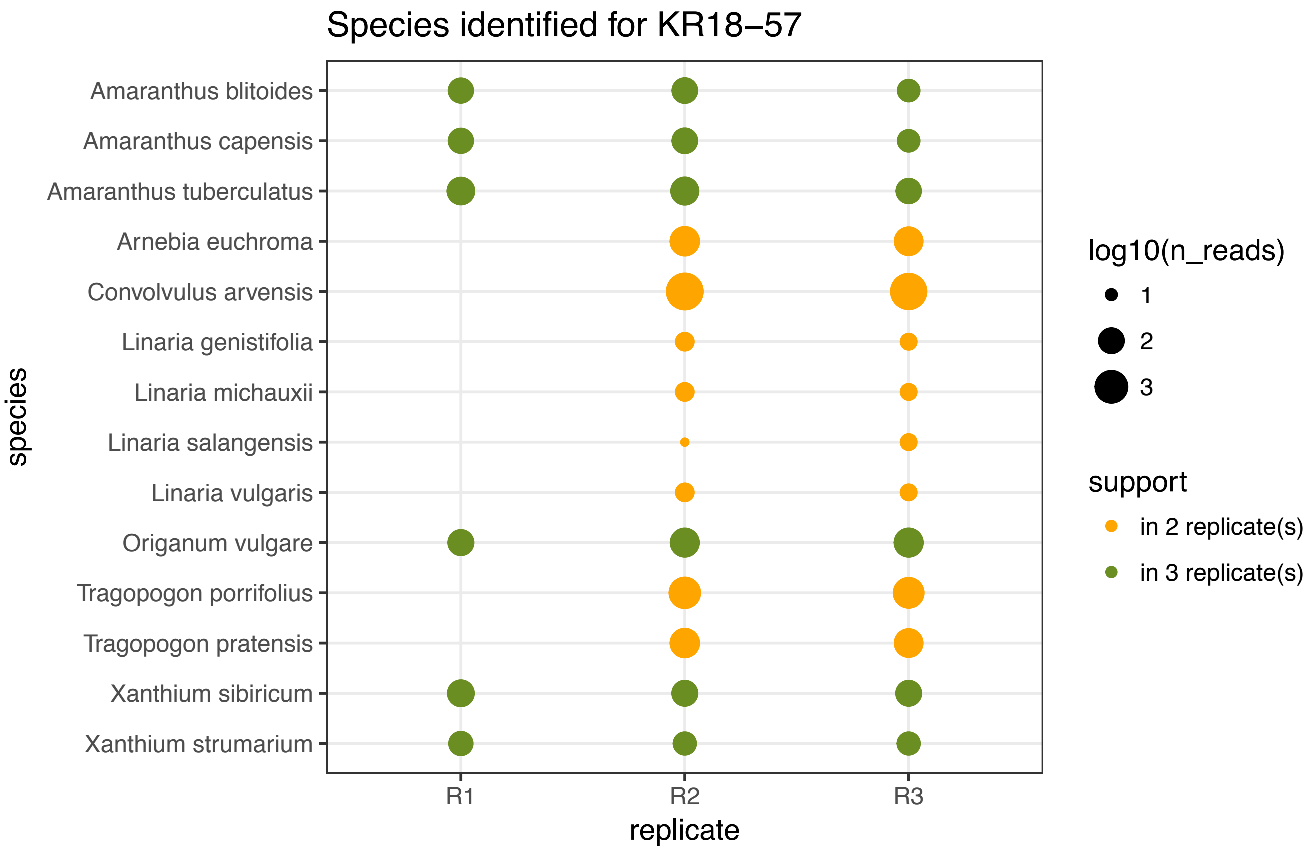

Table 50: Species identified in: KR18-57

species	support
Amaranthus blitoides	3
Amaranthus capensis	3
Amaranthus tuberculatus	3
Arnebia euchroma	2
Convolvulus arvensis	2
Linaria genistifolia	2
Linaria michauxii	2
Linaria salangensis	2
Linaria vulgaris	2
Origanum vulgare	3
Tragopogon porrifolius	2
Tragopogon pratensis	2
Xanthium sibiricum	3
Xanthium strumarium	3

Identifications for KR18-58

Label: Paprika.

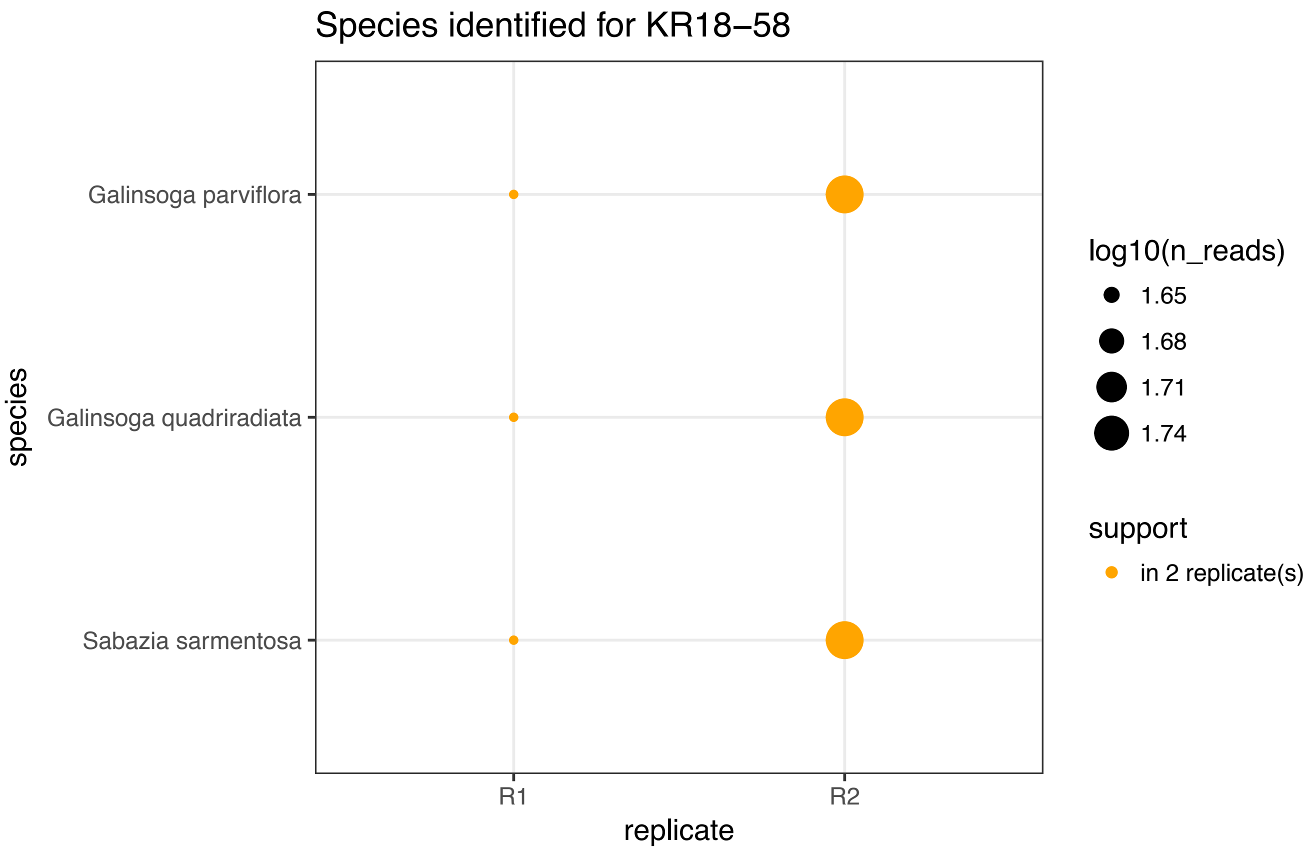

Table 51: Species identified in: KR18-58

species	support
Galinsoga parviflora	2
Galinsoga quadriradiata	2
Sabazia sarmentosa	2

Identifications for KR18-59

Label: Basil.

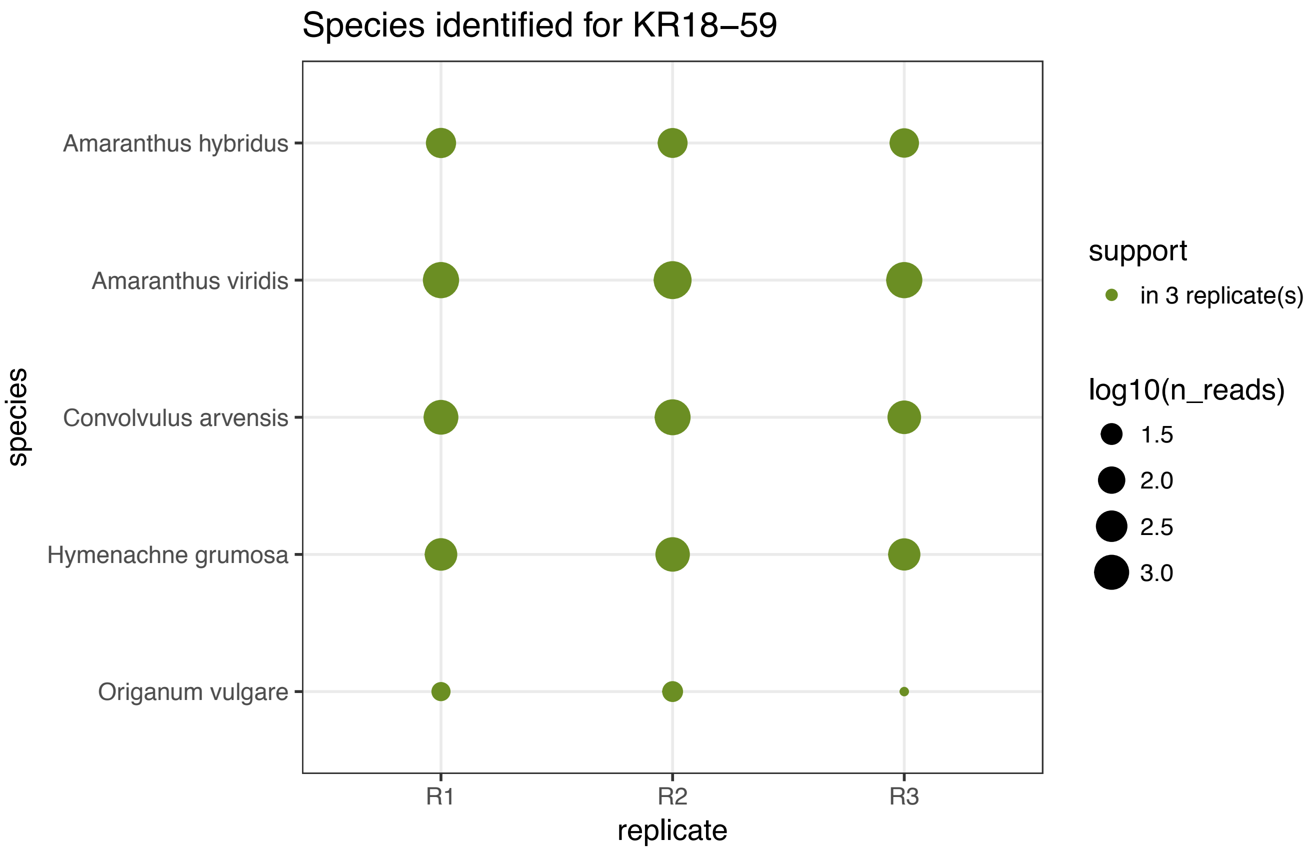

Table 52: Species identified in: KR18-59

species	support
Amaranthus hybridus	3
Amaranthus viridis	3
Convolvulus arvensis	3
Hymenachne grumosa	3
Origanum vulgare	3

Identifications for KR18-60

Label: Paprika. [1] “No species have been identified for KR18-60.”

Identifications for KR18-61

Label: Oregano.

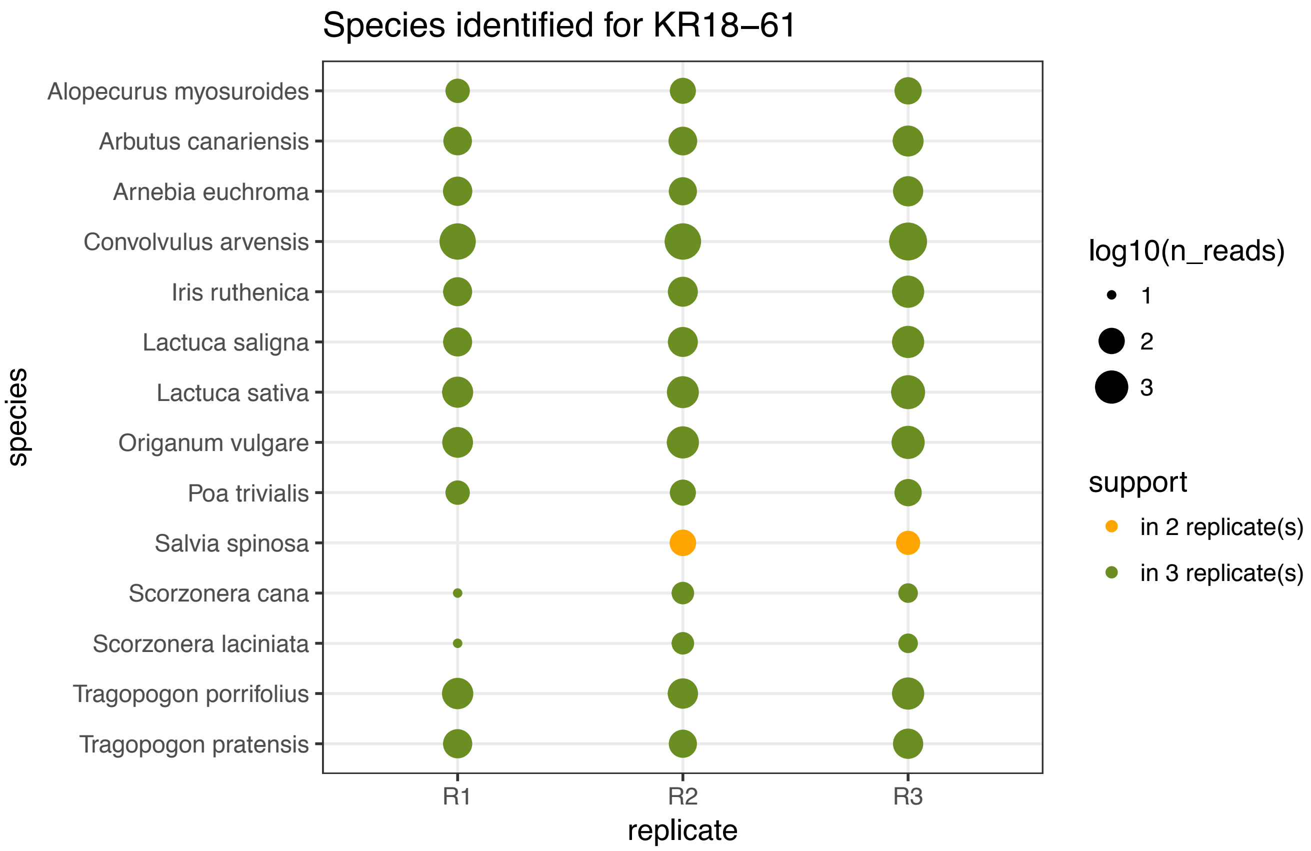

Table 53: Species identified in: KR18-61

species	support
Alopecurus myosuroides	3
Arbutus canariensis	3
Arnebia euchroma	3
Convolvulus arvensis	3
Iris ruthenica	3
Lactuca saligna	3
Lactuca sativa	3
Origanum vulgare	3
Poa trivialis	3
Salvia spinosa	2
Scorzonera cana	3
Scorzonera laciniata	3
Tragopogon porrifolius	3
Tragopogon pratensis	3

Identifications for KR18-62

Label: Oregano.

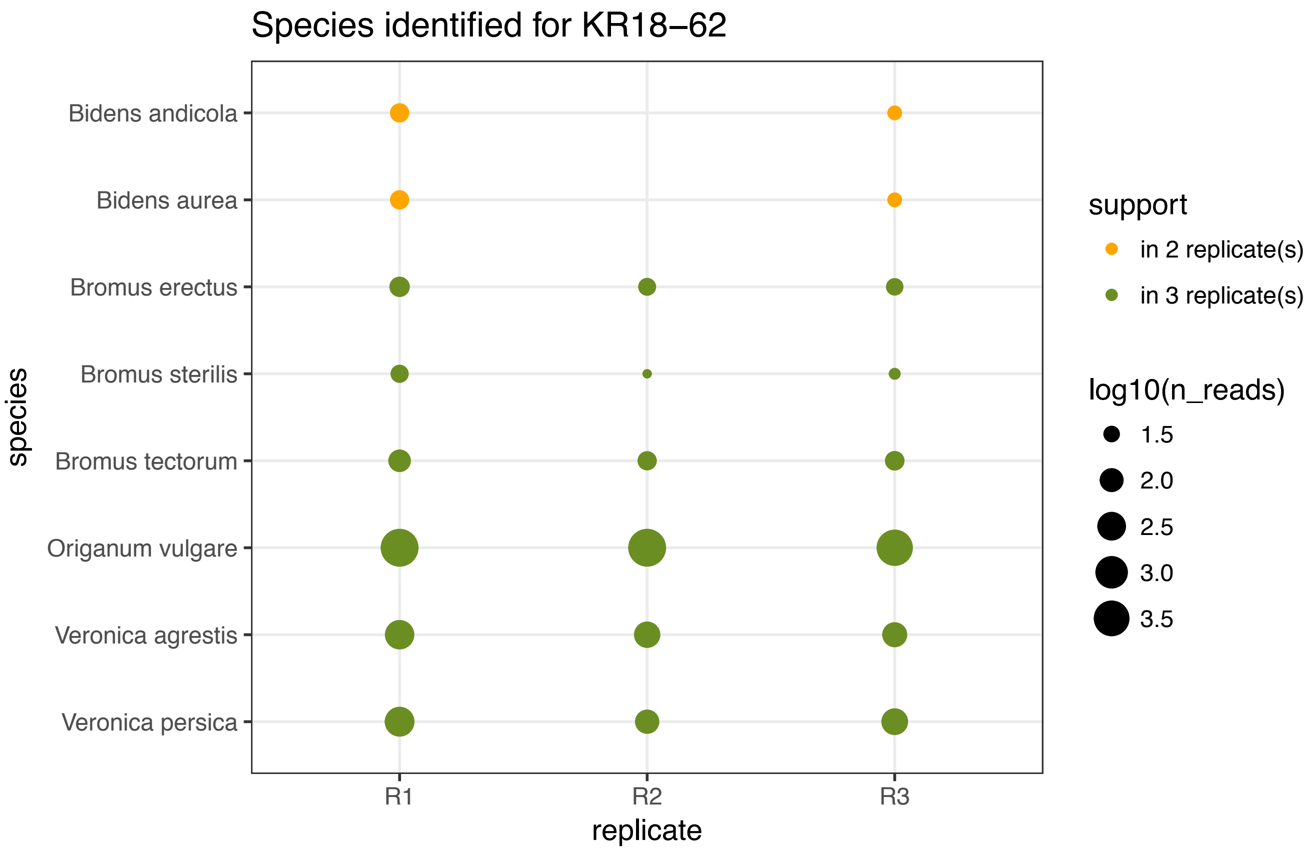

Table 54: Species identified in: KR18-62

species	support
Bidens andicola	2
Bidens aurea	2
Bromus erectus	3
Bromus sterilis	3
Bromus tectorum	3
Origanum vulgare	3
Veronica agrestis	3
Veronica persica	3

Identifications for KR18-ENC

Label: NA. [1] “No species have been identified for KR18-ENC.”

Identifications for KR18-PNC

Label: NA. [1] “No species have been identified for KR18-PNC.”

BLAST queries

qseqid	sseqid	stitle	pident	length
PTU_001	KX166238	Solanum nigrum	94.66	262
PTU_001	KF454049	Solanum nigrum	94.66	262
PTU_001	KY652843	Solanum nigrum	94.27	262
PTU_001	KX282258	Lycium shawii	92.11	266
PTU_002	NA	NA	NA	NA
PTU_003	KF454238	Ocimum basilicum	99.28	277
PTU_003	KX096056	Ocimum americanum	96.75	277
PTU_003	KX096059	Ocimum basilicum	94.93	276
PTU_003	JF301414	Martianthus leucocephalus	83.15	273
PTU_004	KF454118	Origanum vulgare	100.00	302
PTU_004	KX166553	Thymus pulegioides	92.08	303
PTU_004	KF454518	Thymus marschallianus	92.05	302
PTU_004	KC295045	Thymus mongolicus	91.72	302
PTU_005	KF454118	Origanum vulgare	96.68	301
PTU_005	KX166553	Thymus pulegioides	90.76	303
PTU_005	KF454518	Thymus marschallianus	90.73	302
PTU_005	KF454516	Thymus altaicus	90.73	302
PTU_006	KF454118	Origanum vulgare	96.01	301
PTU_006	KX166553	Thymus pulegioides	90.43	303
PTU_006	KF454518	Thymus marschallianus	90.40	302
PTU_006	KF454516	Thymus altaicus	90.40	302
PTU_007	KF454238	Ocimum basilicum	99.64	277
PTU_007	KX096056	Ocimum americanum	96.40	278
PTU_007	KX096059	Ocimum basilicum	94.58	277
PTU_007	JF301414	Martianthus leucocephalus	83.15	273
PTU_008	KF454118	Origanum vulgare	98.01	302

qseqid	sseqid	stitle	pident	length
PTU_008	KX166553	Thymus pulegioides	91.75	303
PTU_008	KF454518	Thymus marschallianus	91.72	302
PTU_008	KC295045	Thymus mongolicus	91.39	302
PTU_009	KX096059	Ocimum basilicum	100.00	271
PTU_009	KX096056	Ocimum americanum	96.01	276
PTU_009	KF454238	Ocimum basilicum	94.95	277
PTU_009	JF301414	Martianthus leucocephalus	82.78	273
PTU_010	KF454072	Convolvulus arvensis	98.97	291
PTU_010	KF454079	Convolvulus arvensis	98.63	291
PTU_010	KF454077	Convolvulus arvensis	98.63	291
PTU_010	586598656	Convolvulus arvensis	98.58	212
PTU_011	KX166238	Solanum nigrum	95.04	262
PTU_011	KF454049	Solanum nigrum	95.04	262
PTU_011	KY652843	Solanum nigrum	94.27	262
PTU_011	KF454093	Lycium ruthenicum	92.65	272
PTU_012	KF454238	Ocimum basilicum	99.64	277
PTU_012	KX096056	Ocimum americanum	97.11	277
PTU_012	KX096059	Ocimum basilicum	95.29	276
PTU_012	JF301414	Martianthus leucocephalus	83.52	273
PTU_013	KF454238	Ocimum basilicum	99.64	276
PTU_013	KX096056	Ocimum americanum	97.10	276
PTU_013	KX096059	Ocimum basilicum	95.27	275
PTU_013	JF301414	Martianthus leucocephalus	83.52	273
PTU_014	KX282189	Helianthemum lippii	91.67	264
PTU_014	KX282184	Helianthemum kahiricum	91.67	264
PTU_014	GU327673	Helianthemum syriacum	91.29	264
PTU_014	KC698933	Helianthemum hirtum	91.25	263

qseqid	sseqid	stitle	pident	length
PTU_015	KX096059	Ocimum basilicum	96.31	271
PTU_015	KF454238	Ocimum basilicum	93.50	277
PTU_015	KX096056	Ocimum americanum	92.39	276
PTU_015	KU203796	Damrongia orientalis	98.90	91
PTU_016	KF454118	Origanum vulgare	96.35	301
PTU_016	KX166553	Thymus pulegioides	91.09	303
PTU_016	KF454518	Thymus marschallianus	91.06	302
PTU_016	KF454516	Thymus altaicus	91.09	303
PTU_017	KX166238	Solanum nigrum	95.06	263
PTU_017	KF454049	Solanum nigrum	95.06	263
PTU_017	KY652843	Solanum nigrum	94.68	263
PTU_017	KX282258	Lycium shawii	91.07	291
PTU_018	KF454518	Thymus marschallianus	99.01	302
PTU_018	KX166553	Thymus pulegioides	98.68	303
PTU_018	KC295045	Thymus mongolicus	98.68	302
PTU_018	KF454516	Thymus altaicus	98.34	302
PTU_019	KF454118	Origanum vulgare	97.01	301
PTU_019	KX166553	Thymus pulegioides	91.09	303
PTU_019	KF454518	Thymus marschallianus	91.06	302
PTU_019	KF454516	Thymus altaicus	91.06	302
PTU_020	NA	NA	NA	NA
PTU_021	KX096059	Ocimum basilicum	96.35	274
PTU_021	KX096056	Ocimum americanum	95.64	275
PTU_021	KF454238	Ocimum basilicum	94.93	276
PTU_021	JF301483	Hyptis pulegioides	81.29	294
PTU_022	KF241280	Arnebia euchroma	99.31	291
PTU_022	KX166735	Tragopogon pratensis	98.63	291

qseqid	sseqid	stitle	pident	length
PTU_022	317451650	Tragopogon porrifolius	100.00	210
PTU_022	317451649	Tragopogon porrifolius	100.00	210
PTU_023	588283767	Amaranthus viridis	100.00	210
PTU_023	663084921	Amaranthus viridis	100.00	206
PTU_023	663084887	Amaranthus viridis	100.00	206
PTU_023	663084893	Amaranthus viridis	100.00	206
PTU_024	KX166238	Solanum nigrum	95.04	262
PTU_024	KF454049	Solanum nigrum	95.04	262
PTU_024	KY652843	Solanum nigrum	94.66	262
PTU_024	KX282258	Lycium shawii	91.03	290
PTU_025	KX096059	Ocimum basilicum	99.63	271
PTU_025	KX096056	Ocimum americanum	95.65	276
PTU_025	KF454238	Ocimum basilicum	94.58	277
PTU_025	JF301414	Martianthus leucocephalus	82.42	273
PTU_026	KX096056	Ocimum americanum	97.10	276
PTU_026	KX096059	Ocimum basilicum	97.09	275
PTU_026	KF454238	Ocimum basilicum	96.39	277
PTU_026	KX534371	Ocimum basilicum	83.96	293
PTU_027	22773674	Hymenachne grumosa	98.05	205
PTU_027	KX282065	Cenchrus ciliaris	88.50	287
PTU_027	LT593966	Brachiaria fragrans	88.42	285
PTU_027	KX282323	Cenchrus ramosissimus	87.37	285
PTU_028	KX096059	Ocimum basilicum	98.52	270
PTU_028	KX096056	Ocimum americanum	96.00	275
PTU_028	KF454238	Ocimum basilicum	94.57	276
PTU_028	JF301414	Martianthus leucocephalus	83.15	273
PTU_029	151547462	Amaranthus hybridus	100.00	209

qseqid	sseqid	stitle	pident	length
PTU_029	67078902	Amaranthus hybridus	100.00	207
PTU_029	663084929	Amaranthus hybridus	100.00	205
PTU_029	663084895	Amaranthus hybridus	100.00	205
PTU_030	LT593966	Brachiaria fragrans	92.96	270
PTU_030	LT593967	Chasechloa egregia	92.19	269
PTU_030	LT593967	Chasechloa egregia	92.19	269
PTU_030	KX689291	Digitaria horizontalis	92.57	269
PTU_031	KX166238	Solanum nigrum	95.44	263
PTU_031	KF454049	Solanum nigrum	95.44	263
PTU_031	KY652843	Solanum nigrum	94.68	263
PTU_031	KX282258	Lycium shawii	92.88	267
PTU_032	KF454118	Origanum vulgare	96.68	301
PTU_032	KX166553	Thymus pulegioides	91.06	302
PTU_032	KF454518	Thymus marschallianus	91.03	301
PTU_032	KF454516	Thymus altaicus	91.06	302
PTU_033	KF454238	Ocimum basilicum	98.19	276
PTU_033	KX096056	Ocimum americanum	97.10	276
PTU_033	KX096059	Ocimum basilicum	95.27	275
PTU_033	JF301483	Hyptis pulegioides	81.63	294
PTU_034	EU955045	Zea mays	98.96	289
PTU_034	BT016655	Zea mays	98.95	285
PTU_034	AJ309824	Zea mays	97.22	288
PTU_034	195610665	Zea mays	98.57	210
PTU_035	KX282104	Cynodon dactylon	96.76	278
PTU_035	KP205451	Cynodon dactylon	96.40	278
PTU_035	3850293	Cynodon dactylon	95.90	195
PTU_035	3850289	Cynodon dactylon	95.38	195

qseqid	sseqid	stitle	pident	length
PTU_036	KF454118	Origanum vulgare	96.68	301
PTU_036	KX166553	Thymus pulegioides	90.40	302
PTU_036	KF454518	Thymus marschallianus	90.37	301
PTU_036	KF454516	Thymus altaicus	90.37	301
PTU_037	KF454118	Origanum vulgare	95.35	301
PTU_037	KX166553	Thymus pulegioides	90.76	303
PTU_037	KF454518	Thymus marschallianus	90.73	302
PTU_037	KF454516	Thymus altaicus	90.76	303
PTU_038	KX166238	Solanum nigrum	94.39	196
PTU_038	KF454049	Solanum nigrum	94.39	196
PTU_038	KY652843	Solanum nigrum	93.88	196
PTU_038	KX346971	Solanum lyratum	92.31	195
PTU_039	KX096059	Ocimum basilicum	99.63	270
PTU_039	KX096056	Ocimum americanum	95.64	275
PTU_039	KF454238	Ocimum basilicum	94.57	276
PTU_039	JF301414	Martianthus leucocephalus	82.42	273
PTU_040	KX282107	Dichanthium annulatum	92.63	285
PTU_040	KX689356	Chrysopogon argutus	92.63	285
PTU_040	KX282217	Imperata cylindrica	92.63	285
PTU_040	KF184927	Saccharum hybrid cultivar	92.63	285
PTU_041	KX282107	Dichanthium annulatum	92.28	285
PTU_041	KX689356	Chrysopogon argutus	92.28	285
PTU_041	KX282217	Imperata cylindrica	92.28	285
PTU_041	KF184927	Saccharum hybrid cultivar	92.28	285
PTU_042	KF454118	Origanum vulgare	96.35	301
PTU_042	KX166553	Thymus pulegioides	90.43	303
PTU_042	KF454518	Thymus marschallianus	90.40	302

qseqid	sseqid	stitle	pident	length
PTU_042	KF454516	Thymus altaicus	90.40	302
PTU_043	KY968831	Cenchrus purpureus	95.44	285
PTU_043	49066367	Cenchrus americanus	98.52	203
PTU_043	KX282065	Cenchrus ciliaris	88.58	289
PTU_043	49066365	Cenchrus americanus	97.52	202
PTU_044	KX096059	Ocimum basilicum	96.72	274
PTU_044	KX096056	Ocimum americanum	96.00	275
PTU_044	KF454238	Ocimum basilicum	94.93	276
PTU_044	JF301483	Hyptis pulegioides	80.95	294
PTU_045	37650547	Olea europaea subsp. europaea	99.03	207
PTU_045	460109779	Olea europaea subsp. europaea	98.06	206
PTU_045	560592114	Olea europaea	98.41	189
PTU_045	460109844	Olea europaea subsp. cuspidata	96.39	194
PTU_046	KX166238	Solanum nigrum	93.89	262
PTU_046	KF454049	Solanum nigrum	93.89	262
PTU_046	KY652843	Solanum nigrum	93.51	262
PTU_046	KX282258	Lycium shawii	91.73	266
PTU_047	KX096059	Ocimum basilicum	98.52	271
PTU_047	KX096056	Ocimum americanum	96.01	276
PTU_047	KF454238	Ocimum basilicum	94.58	277
PTU_047	JF301414	Martianthus leucocephalus	83.15	273
PTU_048	KC295044	Ziziphora clinopodioides	99.65	285
PTU_048	KF454088	Rhaponticum repens	99.30	285
PTU_048	KX167169	Centaurea scabiosa	92.20	282
PTU_048	KX167603	Centaurea scabiosa	92.20	282
PTU_049	157886934	Echinochloa crus-galli	99.52	207
PTU_049	7271144	Echinochloa colona	99.52	207

qseqid	sseqid	stitle	pident	length
PTU_049	166062950	Echinochloa oryzicola	100.00	203
PTU_049	166062970	Echinochloa oryzicola	100.00	203
PTU_050	KF454118	Origanum vulgare	99.34	302
PTU_050	KX166553	Thymus pulegioides	91.78	304
PTU_050	KF454518	Thymus marschallianus	91.75	303
PTU_050	KC295045	Thymus mongolicus	91.42	303
PTU_051	KF241305	Portulaca oleracea	100.00	285
PTU_051	693584052	Portulaca oleracea	100.00	206
PTU_051	KF454245	Portulaca grandiflora	90.53	285
PTU_051	378408120	Portulaca oleracea subsp. granulatostellulata	100.00	202
PTU_052	KJ131557	Secale cereale	100.00	284
PTU_052	JF489233	Secale cereale	99.30	284
PTU_052	KX165429	Elymus repens	95.74	282
PTU_052	KX166524	Elymus athericus	95.39	282
PTU_053	KX166014	Chenopodium ficifolium	96.61	295
PTU_053	KX165819	Halimione portulacoides	95.53	291
PTU_053	KX166563	Halimione portulacoides	95.19	291
PTU_053	JN187957	Atriplex sp. Yeelirrie Station	93.49	292
PTU_054	KX166238	Solanum nigrum	94.66	262
PTU_054	KF454049	Solanum nigrum	94.66	262
PTU_054	KY652843	Solanum nigrum	94.27	262
PTU_054	KX282258	Lycium shawii	92.48	266
PTU_055	KX166238	Solanum nigrum	94.68	263
PTU_055	KF454049	Solanum nigrum	94.68	263
PTU_055	KY652843	Solanum nigrum	94.30	263
PTU_055	316986388	Capsicum frutescens	98.60	215
PTU_056	KF454238	Ocimum basilicum	99.28	277

qseqid	sseqid	stitle	pident	length
PTU_056	KX096056	Ocimum americanum	96.75	277
PTU_056	KX096059	Ocimum basilicum	94.93	276
PTU_056	JF301414	Martianthus leucocephalus	83.21	274
PTU_057	KF454118	Origanum vulgare	97.99	298
PTU_057	KX166553	Thymus pulegioides	91.64	299
PTU_057	KF454518	Thymus marschallianus	91.61	298
PTU_057	KC295045	Thymus mongolicus	91.28	298
PTU_058	KX166238	Solanum nigrum	93.56	264
PTU_058	KF454049	Solanum nigrum	93.56	264
PTU_058	KY652843	Solanum nigrum	92.80	264
PTU_058	KX346971	Solanum lyratum	92.02	263
PTU_059	KX689286	Dactyloctenium ctenoides	93.48	276
PTU_059	139539074	Dactyloctenium aegyptium	100.00	195
PTU_059	295152800	Dactyloctenium aegyptium	100.00	195
PTU_059	560592084	Dactyloctenium aegyptium	98.46	195
PTU_060	KX166238	Solanum nigrum	93.92	263
PTU_060	KF454049	Solanum nigrum	93.92	263
PTU_060	KY652843	Solanum nigrum	93.16	263
PTU_060	KX346971	Solanum lyratum	92.37	262
PTU_061	22773674	Hymenachne grumosa	98.05	205
PTU_061	KX282065	Cenchrus ciliaris	87.46	287
PTU_061	LT593966	Brachiaria fragrans	87.37	285
PTU_061	KX282323	Cenchrus ramosissimus	86.32	285
PTU_062	KF454303	Iris ruthenica	100.00	293
PTU_062	KM210323	Lactuca sativa	99.66	293
PTU_062	KY952679	Lactuca sativa	98.98	293
PTU_062	KX167323	Lactuca saligna	97.61	293

qseqid	sseqid	stitle	pident	length
PTU_063	KF454118	Origanum vulgare	96.01	301
PTU_063	KX166553	Thymus pulegioides	90.43	303
PTU_063	KF454518	Thymus marschallianus	90.40	302
PTU_063	KF454516	Thymus altaicus	90.40	302
PTU_064	KX096056	Ocimum americanum	95.27	275
PTU_064	KX096059	Ocimum basilicum	95.27	275
PTU_064	KF454238	Ocimum basilicum	94.95	277
PTU_064	KX167152	Betonica officinalis	80.22	268
PTU_065	KX096059	Ocimum basilicum	96.35	274
PTU_065	KX096056	Ocimum americanum	95.64	275
PTU_065	KF454238	Ocimum basilicum	94.95	277
PTU_065	JF301483	Hyptis pulegioides	81.29	294
PTU_066	KX166238	Solanum nigrum	94.68	263
PTU_066	KF454049	Solanum nigrum	94.68	263
PTU_066	KY652843	Solanum nigrum	93.92	263
PTU_066	KF454093	Lycium ruthenicum	91.35	289
PTU_067	KF454118	Origanum vulgare	96.35	301
PTU_067	KX166553	Thymus pulegioides	91.42	303
PTU_067	KF454518	Thymus marschallianus	91.39	302
PTU_067	KF454516	Thymus altaicus	91.39	302
PTU_068	KX166238	Solanum nigrum	95.80	262
PTU_068	KF454049	Solanum nigrum	95.80	262
PTU_068	KY652843	Solanum nigrum	95.04	262
PTU_068	KF454093	Lycium ruthenicum	92.65	272
PTU_069	KX096056	Ocimum americanum	97.09	275
PTU_069	KX096059	Ocimum basilicum	97.08	274
PTU_069	KF454238	Ocimum basilicum	96.38	276

qseqid	sseqid	stitle	pident	length
PTU_069	KX534371	Ocimum basilicum	83.96	293
PTU_070	37650547	Olea europaea subsp. europaea	99.03	206
PTU_070	460109779	Olea europaea subsp. europaea	98.05	205
PTU_070	560592114	Olea europaea	98.41	189
PTU_070	460109844	Olea europaea subsp. cuspidata	96.39	194
PTU_071	KX166238	Solanum nigrum	95.42	262
PTU_071	KF454049	Solanum nigrum	95.42	262
PTU_071	KY652843	Solanum nigrum	94.66	262
PTU_071	KX282258	Lycium shawii	92.86	266
PTU_072	459256895	Echinochloa frumentacea	100.00	205
PTU_072	560592089	Echinochloa colona	100.00	205
PTU_072	459256898	Echinochloa frumentacea	100.00	205
PTU_072	LT593966	Brachiaria fragrans	91.85	270
PTU_073	KX165886	Bromus erectus	97.88	283
PTU_073	KX165557	Bromus sterilis	96.82	283
PTU_073	KX282048	Bromus tectorum	96.47	283
PTU_073	KX282047	Bromus tectorum	96.47	283
PTU_074	KF454118	Origanum vulgare	96.35	301
PTU_074	KX166553	Thymus pulegioides	90.43	303
PTU_074	KF454518	Thymus marschallianus	90.40	302
PTU_074	KF454516	Thymus altaicus	90.40	302
PTU_075	KF454238	Ocimum basilicum	98.19	277
PTU_075	KX096056	Ocimum americanum	97.83	277
PTU_075	KX096059	Ocimum basilicum	96.74	276
PTU_075	JF301414	Martianthus leucocephalus	83.52	273
PTU_076	KF454297	Gypsophila altissima	93.17	278
PTU_076	KF454299	Gypsophila altissima	92.81	278

qseqid	sseqid	stitle	pident	length
PTU_076	KX282174	Gypsophila capillaris	90.22	276
PTU_076	JF421553	Vaccaria hispanica	90.11	273
PTU_077	638920304	Ajuga sp. E4	98.98	197
PTU_077	KP718623	Paulownia coreana	86.86	274
PTU_077	KP718625	Paulownia tomentosa	86.50	274
PTU_077	KY067660	Scrophularia arguta	85.56	270
PTU_078	KX166238	Solanum nigrum	95.04	262
PTU_078	KF454049	Solanum nigrum	95.04	262
PTU_078	KY652843	Solanum nigrum	94.27	262
PTU_078	KX282258	Lycium shawii	92.48	266
PTU_079	KX166238	Solanum nigrum	95.06	263
PTU_079	KF454049	Solanum nigrum	95.06	263
PTU_079	KY652843	Solanum nigrum	94.30	263
PTU_079	KF454093	Lycium ruthenicum	92.67	273
PTU_080	KX096059	Ocimum basilicum	97.07	273
PTU_080	KF454238	Ocimum basilicum	94.24	278
PTU_080	KX096056	Ocimum americanum	93.17	278
PTU_080	JF301414	Martianthus leucocephalus	82.35	272
PTU_081	KX165886	Bromus erectus	97.88	283
PTU_081	KX165557	Bromus sterilis	96.82	283
PTU_081	KX282048	Bromus tectorum	96.47	283
PTU_081	KX282047	Bromus tectorum	96.47	283
PTU_082	KF454446	Xanthium sibiricum	94.48	290
PTU_082	KF241289	Xanthium sibiricum	94.48	290
PTU_082	KF454449	Xanthium strumarium	94.14	290
PTU_082	KF767534	Helianthus annuus	92.59	270
PTU_083	151547462	Amaranthus hybridus	99.52	209

qseqid	sseqid	stitle	pident	length
PTU_083	663084892	Amaranthus retroflexus	100.00	205
PTU_083	663084904	Amaranthus palmeri	100.00	205
PTU_083	663084926	Amaranthus retroflexus	100.00	205
PTU_084	KX166238	Solanum nigrum	89.02	255
PTU_084	KF454049	Solanum nigrum	89.02	255
PTU_084	KY652843	Solanum nigrum	88.63	255
PTU_084	KX282258	Lycium shawii	87.20	250
PTU_085	KF454118	Origanum vulgare	96.01	301
PTU_085	KX166553	Thymus pulegioides	90.76	303
PTU_085	KF454518	Thymus marschallianus	90.73	302
PTU_085	KF454516	Thymus altaicus	90.76	303
PTU_086	KF454118	Origanum vulgare	96.68	301
PTU_086	KX166553	Thymus pulegioides	90.76	303
PTU_086	KF454518	Thymus marschallianus	90.73	302
PTU_086	KF454516	Thymus altaicus	90.73	302
PTU_087	KY968830	Bidens alba	94.48	290
PTU_087	KY968833	Bidens pilosa	96.28	269
PTU_087	5771500	Bidens aurea	99.51	205
PTU_087	2329931	Bidens andicola	100.00	201
PTU_088	KF454118	Origanum vulgare	95.00	300
PTU_088	KX166553	Thymus pulegioides	94.68	301
PTU_088	KF454518	Thymus marschallianus	94.67	300
PTU_088	KC295045	Thymus mongolicus	94.33	300
PTU_089	KF454118	Origanum vulgare	96.45	282
PTU_089	KX166553	Thymus pulegioides	90.14	284
PTU_089	KF454518	Thymus marschallianus	90.11	283
PTU_089	KF454516	Thymus altaicus	90.14	284

qseqid	sseqid	stitle	pident	length
PTU_090	22773674	Hymenachne grumosa	97.56	205
PTU_090	KX282065	Cenchrus ciliaris	88.15	287
PTU_090	LT593966	Brachiaria fragrans	88.07	285
PTU_090	KX282323	Cenchrus ramosissimus	87.02	285
PTU_091	KX096059	Ocimum basilicum	96.49	171
PTU_091	KF454238	Ocimum basilicum	93.96	182
PTU_092	KF454118	Origanum vulgare	95.68	301
PTU_092	KX166553	Thymus pulegioides	90.43	303
PTU_092	KF454518	Thymus marschallianus	90.40	302
PTU_092	KF454516	Thymus altaicus	90.40	302
PTU_093	KF454118	Origanum vulgare	97.01	301
PTU_093	KX166553	Thymus pulegioides	91.39	302
PTU_093	KF454518	Thymus marschallianus	91.36	301
PTU_093	KF454516	Thymus altaicus	91.36	301
PTU_094	KF454118	Origanum vulgare	99.01	302
PTU_094	KX166553	Thymus pulegioides	91.09	303
PTU_094	KF454518	Thymus marschallianus	91.06	302
PTU_094	KC295045	Thymus mongolicus	90.73	302
PTU_095	KX165886	Bromus erectus	97.88	283
PTU_095	KX165557	Bromus sterilis	96.82	283
PTU_095	KX282048	Bromus tectorum	96.47	283
PTU_095	KX282047	Bromus tectorum	96.47	283
PTU_096	KY968831	Cenchrus purpureus	94.74	285
PTU_096	49066367	Cenchrus americanus	98.03	203
PTU_096	KX282065	Cenchrus ciliaris	88.24	289
PTU_096	49066365	Cenchrus americanus	97.03	202
PTU_097	560592114	Olea europaea	98.07	207

qseqid	sseqid	stitle	pident	length
PTU_097	460109844	Olea europaea subsp. cuspidata	96.19	210
PTU_097	37650547	Olea europaea subsp. europaea	94.86	214
PTU_097	460109779	Olea europaea subsp. europaea	96.89	193
PTU_098	KF454079	Convolvulus arvensis	98.63	291
PTU_098	KF454077	Convolvulus arvensis	98.63	291
PTU_098	KF454072	Convolvulus arvensis	98.28	291
PTU_098	586598656	Convolvulus arvensis	97.64	212
PTU_099	KX282217	Imperata cylindrica	93.66	284
PTU_099	KX282107	Dichanthium annulatum	92.25	284
PTU_099	KF184927	Saccharum hybrid cultivar	91.23	285
PTU_099	KX282227	Triticum monococcum	90.28	288
PTU_100	KX165886	Bromus erectus	97.17	283
PTU_100	KX165557	Bromus sterilis	96.11	283
PTU_100	KX282048	Bromus tectorum	95.76	283
PTU_100	KX282047	Bromus tectorum	95.76	283
PTU_101	KR532484	Phoebe puwenensis	97.00	300
PTU_101	KR532293	Litsea garrettii	96.67	300
PTU_101	KR531672	Actinodaphne obovata	95.00	300
PTU_101	KU940043	Neolitsea dealbata	96.48	284
PTU_102	KF454118	Origanum vulgare	96.10	282
PTU_102	KX166553	Thymus pulegioides	89.79	284
PTU_102	KF454518	Thymus marschallianus	89.75	283
PTU_102	KF454516	Thymus altaicus	89.79	284
PTU_103	KX096059	Ocimum basilicum	99.63	272
PTU_103	KX096056	Ocimum americanum	95.67	277
PTU_103	KF454238	Ocimum basilicum	94.60	278
PTU_103	JF301414	Martianthus leucocephalus	82.48	274

qseqid	sseqid	stitle	pident	length
PTU_104	KT948617	Chenopodium album	98.63	293
PTU_104	KX166014	Chenopodium ficifolium	94.24	295
PTU_104	311235766	Chenopodium album	99.55	220
PTU_104	KX165819	Halimione portulacoides	92.23	283
PTU_105	KX166238	Solanum nigrum	89.45	256
PTU_105	KF454049	Solanum nigrum	89.45	256
PTU_105	KY652843	Solanum nigrum	89.06	256
PTU_105	KX282258	Lycium shawii	87.60	250
PTU_106	KX282316	Paronychia arabica	92.39	276
PTU_106	KX282315	Paronychia arabica	93.54	263
PTU_106	KX282411	Gymnocarpos sclerocephalus	86.64	262
PTU_106	67078937	Paronychia canadensis	92.93	184
PTU_107	KF454118	Origanum vulgare	96.35	301
PTU_107	KX166553	Thymus pulegioides	89.77	303
PTU_107	KF454518	Thymus marschallianus	89.74	302
PTU_107	KF454516	Thymus altaicus	89.74	302
PTU_108	KF454118	Origanum vulgare	97.01	301
PTU_108	KX166553	Thymus pulegioides	91.39	302
PTU_108	KF454518	Thymus marschallianus	91.36	301
PTU_108	KF454516	Thymus altaicus	91.36	301
PTU_109	KF052129	Sideritis scardica	94.33	300
PTU_109	KF052135	Sideritis raeseri	93.67	300
PTU_109	KX166736	Stachys sylvatica	92.69	301
PTU_109	15429081	Sideritis romana	95.96	223
PTU_110	AB851489	Erigeron annuus	95.26	274
PTU_110	KX166456	Erigeron borealis	94.87	273
PTU_110	4731984	Conyza bonariensis	99.48	193

qseqid	sseqid	stitle	pident	length
PTU_110	4731987	Erigeron rosulatus	98.45	193
PTU_111	KF454118	Origanum vulgare	97.67	301
PTU_111	KX166553	Thymus pulegioides	90.73	302
PTU_111	KF454518	Thymus marschallianus	90.70	301
PTU_111	KF454516	Thymus altaicus	90.70	301
PTU_112	KF454079	Convolvulus arvensis	98.28	291
PTU_112	KF454077	Convolvulus arvensis	98.28	291
PTU_112	KF454072	Convolvulus arvensis	97.94	291
PTU_112	586598656	Convolvulus arvensis	97.64	212
PTU_113	KX166238	Solanum nigrum	95.06	263
PTU_113	KF454049	Solanum nigrum	95.06	263
PTU_113	KY652843	Solanum nigrum	94.30	263
PTU_113	KF454093	Lycium ruthenicum	92.67	273
PTU_114	KX166238	Solanum nigrum	94.30	263
PTU_114	KF454049	Solanum nigrum	94.30	263
PTU_114	KY652843	Solanum nigrum	93.92	263
PTU_114	316986388	Capsicum frutescens	98.60	215
PTU_115	AC215459	Solanum lycopersicum	100.00	286
PTU_115	X52265	Solanum lycopersicum	100.00	286
PTU_115	HG975515	Solanum lycopersicum	100.00	286
PTU_115	HG975441	Solanum pennellii	97.91	287
PTU_116	KF454118	Origanum vulgare	99.67	302
PTU_116	KX166553	Thymus pulegioides	91.75	303
PTU_116	KF454518	Thymus marschallianus	91.72	302
PTU_116	KC295045	Thymus mongolicus	91.39	302
PTU_117	663084936	Amaranthus retroflexus	100.00	205
PTU_117	663084899	Amaranthus retroflexus	100.00	205

qseqid	sseqid	stitle	pident	length
PTU_117	663084909	Amaranthus powellii	100.00	205
PTU_117	663084875	Amaranthus powellii	100.00	205
PTU_118	NA	NA	NA	NA
PTU_119	KX282104	Cynodon dactylon	100.00	278
PTU_119	KP205451	Cynodon dactylon	98.56	278
PTU_119	3282462	Cynodon dactylon	100.00	195
PTU_119	109693371	Cynodon dactylon	100.00	194
PTU_120	KX096059	Ocimum basilicum	96.49	171
PTU_120	KF454238	Ocimum basilicum	93.51	185
PTU_121	KX166238	Solanum nigrum	93.92	263
PTU_121	KY652843	Solanum nigrum	93.54	263
PTU_121	KF454049	Solanum nigrum	93.54	263
PTU_121	KU724227	Withania somnifera	91.63	263
PTU_122	KX166238	Solanum nigrum	94.66	262
PTU_122	KF454049	Solanum nigrum	94.66	262
PTU_122	KY652843	Solanum nigrum	93.89	262
PTU_122	KF454093	Lycium ruthenicum	91.94	273
PTU_123	157886934	Echinochloa crus-galli	99.52	207
PTU_123	7271144	Echinochloa colona	99.52	207
PTU_123	166062950	Echinochloa oryzicola	100.00	203
PTU_123	166062970	Echinochloa oryzicola	100.00	203
PTU_124	KX166238	Solanum nigrum	94.30	263
PTU_124	KF454049	Solanum nigrum	94.30	263
PTU_124	KY652843	Solanum nigrum	93.92	263
PTU_124	316986388	Capsicum frutescens	98.60	215
PTU_125	KX166238	Solanum nigrum	94.66	262
PTU_125	KF454049	Solanum nigrum	94.66	262

qseqid	sseqid	stitle	pident	length
PTU_125	KY652843	Solanum nigrum	94.27	262
PTU_125	KX282258	Lycium shawii	90.69	290
PTU_126	KF454077	Convolvulus arvensis	99.44	180
PTU_126	KF454072	Convolvulus arvensis	99.44	180
PTU_126	586598656	Convolvulus arvensis	99.44	180
PTU_126	45925786	Convolvulus arvensis	99.44	180
PTU_127	KX166238	Solanum nigrum	93.54	263
PTU_127	KF454049	Solanum nigrum	93.54	263
PTU_127	KY652843	Solanum nigrum	93.16	263
PTU_127	KF454093	Lycium ruthenicum	90.81	272
PTU_128	KX166238	Solanum nigrum	94.30	263
PTU_128	KF454049	Solanum nigrum	94.30	263
PTU_128	KY652843	Solanum nigrum	93.92	263
PTU_128	KF454093	Lycium ruthenicum	93.05	259
PTU_129	BT016655	Zea mays	98.25	286
PTU_129	AJ309824	Zea mays	98.25	285
PTU_129	EU955045	Zea mays	97.92	288
PTU_129	KX282217	Imperata cylindrica	89.93	288
PTU_130	KF241280	Arnebia euchroma	99.31	288
PTU_130	KX166735	Tragopogon pratensis	98.61	288
PTU_130	317451650	Tragopogon porrifolius	100.00	210
PTU_130	317451649	Tragopogon porrifolius	100.00	210
PTU_131	KF454079	Convolvulus arvensis	97.95	292
PTU_131	KF454072	Convolvulus arvensis	97.60	292
PTU_131	KF454077	Convolvulus arvensis	97.26	292
PTU_131	586598656	Convolvulus arvensis	96.71	213
PTU_132	KX165633	Glechoma hederacea	89.05	283

qseqid	sseqid	stitle	pident	length
PTU_132	26190454	Nepeta cataria	94.09	203
PTU_132	26190443	Nepeta congesta var. cryptantha	94.12	204
PTU_132	26190460	Nepeta scrophularioides	94.09	203
PTU_133	KX166238	Solanum nigrum	93.92	263
PTU_133	KF454049	Solanum nigrum	93.92	263
PTU_133	KY652843	Solanum nigrum	93.54	263
PTU_133	KF454093	Lycium ruthenicum	91.18	272
PTU_134	KX166238	Solanum nigrum	93.18	264
PTU_134	KF454049	Solanum nigrum	93.18	264
PTU_134	KY652843	Solanum nigrum	92.42	264
PTU_134	KX346971	Solanum lyratum	91.98	262
PTU_135	KX282217	Imperata cylindrica	92.96	284
PTU_135	KX282107	Dichanthium annulatum	92.25	284
PTU_135	KF184927	Saccharum hybrid cultivar	91.23	285
PTU_135	KX282227	Triticum monococcum	90.28	288
PTU_136	KX096059	Ocimum basilicum	95.96	272
PTU_136	KF454238	Ocimum basilicum	93.17	278
PTU_136	KX096056	Ocimum americanum	92.06	277
PTU_136	GU726292	Anisomeles indica	79.70	271
PTU_137	KF454079	Convolvulus arvensis	98.63	291
PTU_137	KF454077	Convolvulus arvensis	98.63	291
PTU_137	KF454072	Convolvulus arvensis	98.28	291
PTU_137	586598656	Convolvulus arvensis	98.11	212
PTU_138	KX096059	Ocimum basilicum	95.20	271
PTU_138	KF454238	Ocimum basilicum	94.22	277
PTU_138	KX096056	Ocimum americanum	93.48	276
PTU_138	JF301414	Martianthus leucocephalus	82.35	272

qseqid	sseqid	stitle	pident	length
PTU_139	JF831213	Muehlenbeckia gracillima	95.77	260
PTU_139	JF831210	K.L.Wilson & Makinson	95.37	259
PTU_139	JF831211	Muehlenbeckia diclina	93.10	261
PTU_139	JF831221	K.L.Wilson & Makinson	92.78	263
PTU_140	KX166238	Solanum nigrum	93.16	263
PTU_140	KF454049	Solanum nigrum	93.16	263
PTU_140	KY652843	Solanum nigrum	92.40	263
PTU_140	KF454093	Lycium ruthenicum	91.89	259
PTU_141	KX166238	Solanum nigrum	89.35	263
PTU_141	KF454049	Solanum nigrum	89.35	263
PTU_141	KY652843	Solanum nigrum	88.97	263
PTU_141	316986388	Capsicum frutescens	93.02	215
PTU_142	KX166238	Solanum nigrum	94.30	263
PTU_142	KF454049	Solanum nigrum	94.30	263
PTU_142	KY652843	Solanum nigrum	93.92	263
PTU_142	316986388	Capsicum frutescens	98.14	215
PTU_143	KX166397	Calystegia soldanella	91.16	294
PTU_143	LC085876	Calystegia hederacea	90.82	294
PTU_143	KX165837	Calystegia sepium	90.48	294
PTU_143	KF454079	Convolvulus arvensis	89.38	292
PTU_144	KX166238	Solanum nigrum	90.49	263
PTU_144	KF454049	Solanum nigrum	90.49	263
PTU_144	KY652843	Solanum nigrum	90.11	263
PTU_144	316986388	Capsicum frutescens	94.39	214
PTU_145	KX166238	Solanum nigrum	94.27	262
PTU_145	KF454049	Solanum nigrum	94.27	262
PTU_145	KY652843	Solanum nigrum	93.89	262

qseqid	sseqid	stitle	pident	length
PTU_145	KX282258	Lycium shawii	92.11	266
PTU_146	MF171466	Ipomoea sp. GO-2017	99.29	283
PTU_146	JQ916065	Ipomoea batatas	99.29	280
PTU_146	KX689306	Ipomoea violacea	91.27	275
PTU_146	514856968	Ipomoea triloba	99.03	207
PTU_147	588283767	Amaranthus viridis	100.00	210
PTU_147	663084921	Amaranthus viridis	100.00	206
PTU_147	663084887	Amaranthus viridis	100.00	206
PTU_147	663084893	Amaranthus viridis	100.00	206
PTU_148	KX166238	Solanum nigrum	94.27	262
PTU_148	KF454049	Solanum nigrum	94.27	262
PTU_148	KY652843	Solanum nigrum	93.89	262
PTU_148	KX282258	Lycium shawii	92.11	266
PTU_149	663084878	Amaranthus tuberculatus	100.00	209
PTU_149	663084873	Amaranthus capensis	100.00	209
PTU_149	663084912	Amaranthus tuberculatus	100.00	209
PTU_149	663084944	Amaranthus blitoides	100.00	209
PTU_150	AB851489	Erigeron annuus	95.62	274
PTU_150	KX166456	Erigeron borealis	94.87	273
PTU_150	4731984	Conyza bonariensis	100.00	193
PTU_150	4731987	Erigeron rosulatus	98.45	193
PTU_151	KY968831	Cenchrus purpureus	92.61	284
PTU_151	KX282065	Cenchrus ciliaris	88.93	289
PTU_151	KY968838	Melinis repens	88.85	287
PTU_151	KX282323	Cenchrus ramosissimus	89.13	276
PTU_152	KX166238	Solanum nigrum	94.30	263
PTU_152	KF454049	Solanum nigrum	94.30	263

qseqid	sseqid	stitle	pident	length
PTU_152	KY652843	Solanum nigrum	93.54	263
PTU_152	KX346971	Solanum lyratum	92.75	262
PTU_153	KF454118	Origanum vulgare	95.68	301
PTU_153	KX166553	Thymus pulegioides	90.10	303
PTU_153	KF454518	Thymus marschallianus	90.07	302
PTU_153	KF454516	Thymus altaicus	90.07	302
PTU_154	KX096059	Ocimum basilicum	92.31	273
PTU_154	KX096056	Ocimum americanum	92.00	275
PTU_154	KF454238	Ocimum basilicum	91.30	276
PTU_154	378406718	Hyptis argyrophylla	86.16	159
PTU_155	KX166505	Crepis foetida	99.66	295
PTU_155	KX166910	Crepis paludosa	93.22	295
PTU_155	KX166909	Crepis paludosa	92.88	295
PTU_155	KX166259	Crepis mollis	90.51	295
PTU_156	KX096059	Ocimum basilicum	98.53	272
PTU_156	KX096056	Ocimum americanum	94.58	277
PTU_156	KF454238	Ocimum basilicum	94.24	278
PTU_156	JF301414	Martianthus leucocephalus	82.78	273
PTU_157	KX282217	Imperata cylindrica	92.96	284
PTU_157	KX282107	Dichanthium annulatum	92.61	284
PTU_157	KF184927	Saccharum hybrid cultivar	91.23	285
PTU_157	KX282227	Triticum monococcum	90.62	288
PTU_158	KX166238	Solanum nigrum	92.75	262
PTU_158	KF454049	Solanum nigrum	92.75	262
PTU_158	KY652843	Solanum nigrum	92.37	262
PTU_158	KX282258	Lycium shawii	90.77	260
PTU_159	KF241280	Arnebia euchroma	98.97	291

qseqid	sseqid	stitle	pident	length
PTU_159	KX166735	Tragopogon pratensis	98.63	291
PTU_159	317451650	Tragopogon porrifolius	99.52	210
PTU_159	317451649	Tragopogon porrifolius	99.52	210
PTU_160	KX166814	Polygonum arenastrum	98.87	265
PTU_160	KF530289	Polygonum aviculare	95.09	265
PTU_160	KX166588	Polygonum aviculare	94.72	265
PTU_160	284811160	Polygonum rurivagum	99.45	182
PTU_161	KX165886	Bromus erectus	97.17	283
PTU_161	KX165557	Bromus sterilis	96.11	283
PTU_161	KX282048	Bromus tectorum	95.76	283
PTU_161	KX282047	Bromus tectorum	95.76	283
PTU_162	KX166238	Solanum nigrum	92.22	257
PTU_162	KY652843	Solanum nigrum	91.83	257
PTU_162	KF454049	Solanum nigrum	91.83	257
PTU_162	KX346971	Solanum lyratum	89.45	256
PTU_163	KU350154	Arbutus canariensis	97.33	300
PTU_163	KX165505	Arctous alpina	89.07	302
PTU_163	KU350164	Comarostaphylis diversifolia	88.67	300
PTU_163	KF419121	Comarostaphylis arbutoides	88.74	293
PTU_164	KY652843	Solanum nigrum	90.60	266
PTU_164	KX166238	Solanum nigrum	90.23	266
PTU_164	KF454049	Solanum nigrum	90.23	266
PTU_164	KF454093	Lycium ruthenicum	89.13	276
PTU_165	MF171466	Ipomoea sp. GO-2017	98.95	287
PTU_165	JQ916065	Ipomoea batatas	98.61	287
PTU_165	514856968	Ipomoea triloba	99.04	208
PTU_165	514856965	Ipomoea triloba	99.04	208

qseqid	sseqid	stitle	pident	length
PTU_166	KF454072	Convolvulus arvensis	97.59	291
PTU_166	KF454079	Convolvulus arvensis	97.25	291
PTU_166	KF454077	Convolvulus arvensis	97.25	291
PTU_166	586598656	Convolvulus arvensis	98.58	212
PTU_167	KX096056	Ocimum americanum	90.58	276
PTU_167	KX096059	Ocimum basilicum	90.51	274
PTU_167	KF454238	Ocimum basilicum	90.25	277
PTU_167	378406718	Hyptis argyrophylla	96.00	100
PTU_168	KT948624	Veronica persica	100.00	279
PTU_168	LC027918	Veronica persica	99.64	279
PTU_168	KX165593	Veronica agrestis	96.74	276
PTU_168	KX165590	Veronica agrestis	96.38	276
PTU_169	KC295045	Thymus mongolicus	100.00	302
PTU_169	KF454518	Thymus marschallianus	99.67	302
PTU_169	KX166553	Thymus pulegioides	99.34	303
PTU_169	KF454516	Thymus altaicus	99.01	302
PTU_170	KX166814	Polygonum arenastrum	99.25	265
PTU_170	KF530289	Polygonum aviculare	94.72	265
PTU_170	KX166588	Polygonum aviculare	94.34	265
PTU_170	JF831221	K.L.Wilson & Makinson	89.55	268
PTU_171	KX166238	Solanum nigrum	93.00	257
PTU_171	KF454049	Solanum nigrum	93.00	257
PTU_171	KY652843	Solanum nigrum	92.61	257
PTU_171	316986388	Capsicum frutescens	96.28	215
PTU_172	KX166238	Solanum nigrum	95.06	263
PTU_172	KF454049	Solanum nigrum	95.06	263
PTU_172	KY652843	Solanum nigrum	94.30	263

qseqid	sseqid	stitle	pident	length
PTU_172	KF454093	Lycium ruthenicum	91.94	273
PTU_173	KM210331	Lavandula angustifolia	91.00	300
PTU_173	KX282394	Salvia spinosa	86.38	279
PTU_173	KX166755	Salvia pratensis	86.07	280
PTU_173	KC591663	Acanthomintha lanceolata	85.97	278
PTU_174	KF052129	Sideritis scardica	94.00	300
PTU_174	KF052135	Sideritis raeseri	93.33	300
PTU_174	KX166736	Stachys sylvatica	92.36	301
PTU_174	15429081	Sideritis romana	95.52	223
PTU_175	KF454118	Origanum vulgare	96.01	301
PTU_175	KX166553	Thymus pulegioides	90.76	303
PTU_175	KF454518	Thymus marschallianus	90.73	302
PTU_175	KF454516	Thymus altaicus	90.73	302
PTU_176	KF454118	Origanum vulgare	96.35	301
PTU_176	KX166553	Thymus pulegioides	91.09	303
PTU_176	KF454518	Thymus marschallianus	91.06	302
PTU_176	KF454516	Thymus altaicus	91.06	302
PTU_177	KX165593	Veronica agrestis	98.19	276
PTU_177	KX165590	Veronica agrestis	97.83	276
PTU_177	LC027918	Veronica persica	96.77	279
PTU_177	KT948624	Veronica persica	96.77	279
PTU_178	KX166238	Solanum nigrum	93.92	263
PTU_178	KF454049	Solanum nigrum	93.92	263
PTU_178	KY652843	Solanum nigrum	93.54	263
PTU_178	316986388	Capsicum frutescens	97.67	215
PTU_179	KX166814	Polygonum arenastrum	99.25	265
PTU_179	KF530289	Polygonum aviculare	94.74	266

qseqid	sseqid	stitle	pident	length
PTU_179	KX166588	Polygonum aviculare	94.36	266
PTU_179	JF831221	K.L.Wilson & Makinson	89.55	268
PTU_180	KF454296	Daucus carota	91.13	293
PTU_180	KF454292	Daucus carota	90.78	293
PTU_180	FJ150184	Daucus carota	90.94	287
PTU_180	X17534	Daucus carota	90.44	293
PTU_181	KX166238	Solanum nigrum	93.89	262
PTU_181	KF454049	Solanum nigrum	93.89	262
PTU_181	KY652843	Solanum nigrum	93.51	262
PTU_181	KX282258	Lycium shawii	91.73	266
PTU_182	KX282189	Helianthemum lippii	93.56	264
PTU_182	KX282184	Helianthemum kahiricum	93.56	264
PTU_182	GU327673	Helianthemum syriacum	93.56	264
PTU_182	GU327674	Helianthemum syriacum	93.18	264
PTU_183	KX282181	Haloxylon salicornicum	92.42	277
PTU_183	KX282180	Haloxylon salicornicum	92.39	276
PTU_183	KX282182	Haloxylon salicornicum	92.03	276
PTU_183	KX282091	Cornulaca monacantha	91.34	277
PTU_184	KX165886	Bromus erectus	98.58	281
PTU_184	KX165557	Bromus sterilis	97.51	281
PTU_184	KX282048	Bromus tectorum	97.15	281
PTU_184	KX282047	Bromus tectorum	97.15	281
PTU_185	204306817	Corchorus olitorius	100.00	222
PTU_185	83282752	Corchorus olitorius	100.00	222
PTU_185	83282751	Corchorus capsularis	91.93	223
PTU_185	219881458	Corchorus pseudo-olitorius	91.52	224
PTU_186	KX166238	Solanum nigrum	94.30	263

qseqid	sseqid	stitle	pident	length
PTU_186	KF454049	Solanum nigrum	94.30	263
PTU_186	KY652843	Solanum nigrum	93.92	263
PTU_186	316986388	Capsicum frutescens	98.14	215
PTU_187	KX689286	Dactyloctenium ctenoides	93.45	275
PTU_187	139539074	Dactyloctenium aegyptium	99.49	195
PTU_187	295152800	Dactyloctenium aegyptium	99.49	195
PTU_187	560592084	Dactyloctenium aegyptium	97.95	195
PTU_188	KF241280	Arnebia euchroma	98.61	288
PTU_188	KX166735	Tragopogon pratensis	97.92	288
PTU_188	317451650	Tragopogon porrifolius	99.05	210
PTU_188	317451649	Tragopogon porrifolius	99.05	210
PTU_189	KX166238	Solanum nigrum	91.37	255
PTU_189	KY652843	Solanum nigrum	90.98	255
PTU_189	KF454049	Solanum nigrum	90.98	255
PTU_189	KX282258	Lycium shawii	88.76	258
PTU_190	KX689291	Digitaria horizontalis	96.80	281
PTU_190	KX282108	Digitaria ciliaris	94.66	281
PTU_190	LT593966	Brachiaria fragrans	90.59	287
PTU_190	LT593967	Chasechloa egregia	90.21	286
PTU_191	KF241280	Arnebia euchroma	96.91	291
PTU_191	KX166735	Tragopogon pratensis	96.22	291
PTU_191	317451650	Tragopogon porrifolius	100.00	210
PTU_191	317451649	Tragopogon porrifolius	100.00	210
PTU_192	KT948617	Chenopodium album	100.00	208
PTU_192	323574403	Chenopodium environmental sample	100.00	208
PTU_192	283466107	Chenopodium giganteum	100.00	208
PTU_192	283466105	Chenopodium album	100.00	208

qseqid	sseqid	stitle	pident	length
PTU_193	KF454088	Rhaponticum repens	98.23	283
PTU_193	KC295044	Ziziphora clinopodioides	98.23	283
PTU_193	KX167169	Centaurea scabiosa	90.91	286
PTU_193	KX167603	Centaurea scabiosa	90.91	286
PTU_194	KX165772	Senecio vulgaris	99.65	284
PTU_194	KX166575	Senecio vulgaris	99.30	284
PTU_194	KT948631	Senecio vulgaris	99.30	284
PTU_194	KX282420	Senecio glaucus	94.83	290
PTU_195	KX282217	Imperata cylindrica	94.01	284
PTU_195	KX282107	Dichanthium annulatum	92.61	284
PTU_195	KF184927	Saccharum hybrid cultivar	91.58	285
PTU_195	KX282227	Triticum monococcum	90.62	288
PTU_196	KX282189	Helianthemum lippii	90.91	264
PTU_196	KX282184	Helianthemum kahiricum	90.91	264
PTU_196	GU327673	Helianthemum syriacum	90.49	263
PTU_196	KC698933	Helianthemum hirtum	90.49	263
PTU_197	KX166238	Solanum nigrum	91.63	263
PTU_197	KF454049	Solanum nigrum	91.63	263
PTU_197	KY652843	Solanum nigrum	91.25	263
PTU_197	316986388	Capsicum frutescens	95.75	212
PTU_198	KF454077	Convolvulus arvensis	100.00	176
PTU_198	KF454072	Convolvulus arvensis	100.00	176
PTU_198	586598656	Convolvulus arvensis	100.00	176
PTU_198	45925786	Convolvulus arvensis	100.00	176
PTU_199	KX166238	Solanum nigrum	95.82	263
PTU_199	KF454049	Solanum nigrum	95.82	263
PTU_199	KY652843	Solanum nigrum	95.06	263

qseqid	sseqid	stitle	pident	length
PTU_199	KF454093	Lycium ruthenicum	92.67	273
PTU_200	KX282189	Helianthemum lippii	91.29	264
PTU_200	KX282184	Helianthemum kahiricum	91.29	264
PTU_200	GU327673	Helianthemum syriacum	90.87	263
PTU_200	KC698933	Helianthemum hirtum	90.87	263
PTU_201	KF454118	Origanum vulgare	96.35	301
PTU_201	KX166553	Thymus pulegioides	90.73	302
PTU_201	KF454518	Thymus marschallianus	90.70	301
PTU_201	KF454516	Thymus altaicus	90.73	302
PTU_202	KX166238	Solanum nigrum	92.80	250
PTU_202	KF454049	Solanum nigrum	92.80	250
PTU_202	KY652843	Solanum nigrum	92.40	250
PTU_202	KX282258	Lycium shawii	89.14	267
PTU_203	KX166238	Solanum nigrum	95.04	262
PTU_203	KF454049	Solanum nigrum	95.04	262
PTU_203	KY652843	Solanum nigrum	94.27	262
PTU_203	KF454093	Lycium ruthenicum	91.91	272
PTU_204	KF454049	Solanum nigrum	94.68	263
PTU_204	KX166238	Solanum nigrum	94.30	263
PTU_204	KY652843	Solanum nigrum	93.92	263
PTU_204	KX282258	Lycium shawii	92.51	267
PTU_205	KF454238	Ocimum basilicum	96.55	174
PTU_205	KX096059	Ocimum basilicum	94.74	171
PTU_206	KX166238	Solanum nigrum	90.49	263
PTU_206	KF454049	Solanum nigrum	90.49	263
PTU_206	KY652843	Solanum nigrum	90.11	263
PTU_206	316986388	Capsicum frutescens	94.88	215

qseqid	sseqid	stitle	pident	length
PTU_207	151547462	Amaranthus hybridus	99.52	209
PTU_207	663084892	Amaranthus retroflexus	100.00	205
PTU_207	663084904	Amaranthus palmeri	100.00	205
PTU_207	663084926	Amaranthus retroflexus	100.00	205
PTU_208	KX166238	Solanum nigrum	95.04	262
PTU_208	KF454049	Solanum nigrum	95.04	262
PTU_208	KY652843	Solanum nigrum	94.27	262
PTU_208	KF454093	Lycium ruthenicum	91.32	288
PTU_209	224552045	Galinsoga parviflora	100.00	216
PTU_209	308229127	Galinsoga quadriradiata	100.00	216
PTU_209	671706816	Sabazia sarmentosa	99.10	221
PTU_209	KT004475	Barleria repens	89.77	264
PTU_210	KF241280	Arnebia euchroma	98.63	292
PTU_210	KX166735	Tragopogon pratensis	97.95	292
PTU_210	126153720	Tragopogon dubius	100.00	211
PTU_210	290796685	Tragopogon dubius	100.00	211
PTU_211	KX166238	Solanum nigrum	94.68	263
PTU_211	KF454049	Solanum nigrum	94.68	263
PTU_211	KY652843	Solanum nigrum	93.92	263
PTU_211	KF454093	Lycium ruthenicum	90.97	288
PTU_212	KF454118	Origanum vulgare	96.35	301
PTU_212	KX166553	Thymus pulegioides	90.43	303
PTU_212	KF454518	Thymus marschallianus	90.40	302
PTU_212	KF454516	Thymus altaicus	90.43	303
PTU_213	KF454238	Ocimum basilicum	99.27	275
PTU_213	KX096056	Ocimum americanum	96.73	275
PTU_213	KX096059	Ocimum basilicum	94.89	274

qseqid	sseqid	stitle	pident	length
PTU_213	JF301414	Martianthus leucocephalus	83.15	273
PTU_214	KX096056	Ocimum americanum	91.67	276
PTU_214	KX096059	Ocimum basilicum	91.27	275
PTU_214	KF454238	Ocimum basilicum	90.61	277
PTU_214	KY858250	Alsobia dianthiflora	78.38	259
PTU_215	KF454118	Origanum vulgare	96.35	301
PTU_215	KX166553	Thymus pulegioides	89.77	303
PTU_215	KF454518	Thymus marschallianus	89.74	302
PTU_215	KF454516	Thymus altaicus	89.77	303
PTU_216	KJ131569	Triticum turgidum	100.00	285
PTU_216	KJ131560	Triticum turgidum	99.65	285
PTU_216	KJ131565	Triticum aestivum	99.30	285
PTU_216	KJ131556	Triticum turgidum	99.30	285
PTU_217	KU350154	Arbutus canariensis	96.03	302
PTU_217	KX165505	Arctous alpina	88.45	303
PTU_217	KU350164	Comarostaphylis diversifolia	87.75	302
PTU_217	KF419121	Comarostaphylis arbutoides	88.14	295
PTU_218	KX166397	Calystegia soldanella	90.82	294
PTU_218	LC085876	Calystegia hederacea	90.48	294
PTU_218	KX165837	Calystegia sepium	90.14	294
PTU_218	KF454079	Convolvulus arvensis	90.07	292
PTU_219	KF454118	Origanum vulgare	95.36	302
PTU_219	KX166553	Thymus pulegioides	90.13	304
PTU_219	KF454518	Thymus marschallianus	90.10	303
PTU_219	KF454516	Thymus altaicus	90.10	303
PTU_220	KY968830	Bidens alba	98.62	290
PTU_220	KY968833	Bidens pilosa	99.63	269

qseqid	sseqid	stitle	pident	length
PTU_220	KX165434	Bidens cernua	92.51	267
PTU_220	18028503	Bidens cronquistii	99.51	206
PTU_221	KU350154	Arbutus canariensis	97.00	300
PTU_221	KX165505	Arctous alpina	89.11	303
PTU_221	KU350185	Xylococcus bicolor	88.37	301
PTU_221	KU350164	Comarostaphylis diversifolia	88.45	303
PTU_222	KX166238	Solanum nigrum	94.68	263
PTU_222	KF454049	Solanum nigrum	94.68	263
PTU_222	KY652843	Solanum nigrum	93.92	263
PTU_222	316986388	Capsicum frutescens	98.14	215
PTU_223	KF454446	Xanthium sibiricum	94.54	293
PTU_223	KF241289	Xanthium sibiricum	94.54	293
PTU_223	KF454449	Xanthium strumarium	94.20	293
PTU_223	KF767534	Helianthus annuus	92.67	273
PTU_224	KX166238	Solanum nigrum	95.06	263
PTU_224	KF454049	Solanum nigrum	95.06	263
PTU_224	KY652843	Solanum nigrum	94.30	263
PTU_224	KX282258	Lycium shawii	92.51	267
PTU_225	KX096059	Ocimum basilicum	95.56	270
PTU_225	KX096056	Ocimum americanum	94.55	275
PTU_225	KF454238	Ocimum basilicum	94.20	276
PTU_225	KX534371	Ocimum basilicum	82.88	292
PTU_226	KX166238	Solanum nigrum	93.89	262
PTU_226	KF454049	Solanum nigrum	93.89	262
PTU_226	KY652843	Solanum nigrum	93.51	262
PTU_226	KX282258	Lycium shawii	91.73	266
PTU_227	KU350154	Arbutus canariensis	99.66	298

qseqid	sseqid	stitle	pident	length
PTU_227	KX165505	Arctous alpina	89.63	299
PTU_227	KU350164	Comarostaphylis diversifolia	89.60	298
PTU_227	KF419121	Comarostaphylis arbutoides	89.69	291
PTU_228	KY968830	Bidens alba	94.43	287
PTU_228	KY968833	Bidens pilosa	96.24	266
PTU_228	5771500	Bidens aurea	99.51	205
PTU_228	2329931	Bidens andicola	100.00	201
PTU_229	KX096056	Ocimum americanum	95.65	276
PTU_229	KX096059	Ocimum basilicum	95.64	275
PTU_229	KF454238	Ocimum basilicum	95.31	277
PTU_229	JF301483	Hyptis pulegioides	81.29	294
PTU_230	KY652843	Solanum nigrum	91.19	261
PTU_230	KX166238	Solanum nigrum	90.80	261
PTU_230	KF454049	Solanum nigrum	90.80	261
PTU_230	KX282258	Lycium shawii	89.92	258
PTU_231	KF241280	Arnebia euchroma	96.91	291
PTU_231	KX166735	Tragopogon pratensis	96.22	291
PTU_231	317451650	Tragopogon porrifolius	100.00	210
PTU_231	317451649	Tragopogon porrifolius	100.00	210
PTU_232	KY968830	Bidens alba	94.14	290
PTU_232	KY968833	Bidens pilosa	95.91	269
PTU_232	5771500	Bidens aurea	99.02	205
PTU_232	2329931	Bidens andicola	99.50	201
PTU_233	KF454118	Origanum vulgare	99.01	302
PTU_233	KX166553	Thymus pulegioides	91.45	304
PTU_233	KF454518	Thymus marschallianus	91.42	303
PTU_233	KC295045	Thymus mongolicus	91.09	303

qseqid	sseqid	stitle	pident	length
PTU_234	KX166238	Solanum nigrum	94.68	263
PTU_234	KF454049	Solanum nigrum	94.68	263
PTU_234	KY652843	Solanum nigrum	94.30	263
PTU_234	KX282258	Lycium shawii	90.72	291
PTU_235	KX167814	Mentha pulegium	94.37	302
PTU_235	KC591663	Acanthomintha lanceolata	93.67	300
PTU_235	KC591662	Acanthomintha ilicifolia	93.00	300
PTU_235	56069061	Mentha longifolia	100.00	219
PTU_236	KF454118	Origanum vulgare	98.01	302
PTU_236	KX166553	Thymus pulegioides	91.09	303
PTU_236	KF454518	Thymus marschallianus	91.06	302
PTU_236	KC295045	Thymus mongolicus	90.73	302
PTU_237	KX282179	Halothamnus iraqensis	94.12	272
PTU_237	KX282181	Haloxylon salicornicum	92.00	275
PTU_237	KX282182	Haloxylon salicornicum	91.64	275
PTU_237	KX282180	Haloxylon salicornicum	91.27	275
PTU_238	KF454118	Origanum vulgare	99.01	302
PTU_238	KX166553	Thymus pulegioides	91.42	303
PTU_238	KF454518	Thymus marschallianus	91.39	302
PTU_238	KC295045	Thymus mongolicus	91.06	302
PTU_239	KX166238	Solanum nigrum	89.69	262
PTU_239	KF454049	Solanum nigrum	89.69	262
PTU_239	KY652843	Solanum nigrum	89.31	262
PTU_239	KX346971	Solanum lyratum	87.79	262
PTU_240	KJ131556	Triticum turgidum	100.00	285
PTU_240	KJ131570	Triticum spelta	99.65	285
PTU_240	KJ131560	Triticum turgidum	99.65	285

qseqid	sseqid	stitle	pident	length
PTU_240	KJ131569	Triticum turgidum	99.30	285
PTU_241	LT593966	Brachiaria fragrans	91.48	270
PTU_241	KX689291	Digitaria horizontalis	91.45	269
PTU_241	LT593967	Chasechloa egregia	90.33	269
PTU_241	LT593967	Chasechloa egregia	90.33	269
PTU_242	KY968831	Cenchrus purpureus	89.93	288
PTU_242	KX282323	Cenchrus ramosissimus	89.20	287
PTU_242	KX282065	Cenchrus ciliaris	89.24	288
PTU_242	KY968838	Melinis repens	89.20	287
PTU_243	KX166238	Solanum nigrum	92.72	261
PTU_243	KF454049	Solanum nigrum	92.72	261
PTU_243	KY652843	Solanum nigrum	92.34	261
PTU_243	KX656898	Lycium barbarum	90.70	258
PTU_244	KX166238	Solanum nigrum	94.30	263
PTU_244	KF454049	Solanum nigrum	94.30	263
PTU_244	KY652843	Solanum nigrum	93.92	263
PTU_244	316986388	Capsicum frutescens	98.14	215
PTU_245	KX166238	Solanum nigrum	94.27	262
PTU_245	KF454049	Solanum nigrum	94.27	262
PTU_245	KY652843	Solanum nigrum	93.89	262
PTU_245	316986388	Capsicum frutescens	98.14	215
PTU_246	LT593966	Brachiaria fragrans	91.29	287
PTU_246	KX282323	Cenchrus ramosissimus	91.30	276
PTU_246	KX282065	Cenchrus ciliaris	90.59	287
PTU_246	KY968838	Melinis repens	89.90	287
PTU_247	KX282181	Haloxylon salicornicum	92.06	277
PTU_247	KX282180	Haloxylon salicornicum	92.06	277

qseqid	sseqid	stitle	pident	length
PTU_247	KX282182	Haloxylon salicornicum	91.70	277
PTU_247	KX282091	Cornulaca monacantha	90.97	277
PTU_248	KX166238	Solanum nigrum	94.27	262
PTU_248	KF454049	Solanum nigrum	94.27	262
PTU_248	KY652843	Solanum nigrum	93.89	262
PTU_248	KF454093	Lycium ruthenicum	93.02	258
PTU_249	22773674	Hymenachne grumosa	97.56	205
PTU_249	LT593966	Brachiaria fragrans	87.02	285
PTU_249	KX282065	Cenchrus ciliaris	86.41	287
PTU_249	KX282310	Panicum turgidum	85.96	285
PTU_250	KX166238	Solanum nigrum	94.25	261
PTU_250	KF454049	Solanum nigrum	94.25	261
PTU_250	KY652843	Solanum nigrum	93.87	261
PTU_250	KX282258	Lycium shawii	92.64	258
PTU_251	295152695	Leptochloa filiformis	99.49	198
PTU_251	409690876	Dinebra panicea subsp. mucronata	100.00	192
PTU_251	139539097	Dinebra panicea subsp. brachiata	98.99	198
PTU_251	295152881	Dinebra retroflexa	97.47	198
PTU_252	KX165842	Linaria vulgaris	99.30	285
PTU_252	KT031859	Linaria genistifolia	98.95	285
PTU_252	KT031864	Linaria japonica	98.60	285
PTU_252	KT031866	Linaria michauxii	98.25	285
PTU_253	KX096056	Ocimum americanum	93.38	272
PTU_253	KX096059	Ocimum basilicum	92.99	271
PTU_253	KF454238	Ocimum basilicum	92.31	273
PTU_253	KU203796	Damrongia orientalis	97.80	91
PTU_254	KF454118	Origanum vulgare	96.35	301

qseqid	sseqid	stitle	pident	length
PTU_254	KX166553	Thymus pulegioides	89.77	303
PTU_254	KF454518	Thymus marschallianus	89.74	302
PTU_254	KF454516	Thymus altaicus	89.74	302
PTU_255	KX096056	Ocimum americanum	93.84	276
PTU_255	KX096059	Ocimum basilicum	93.82	275
PTU_255	KF454238	Ocimum basilicum	93.14	277
PTU_255	378406713	Hyptis leptostachys	90.77	130
PTU_256	KX166238	Solanum nigrum	94.27	262
PTU_256	KF454049	Solanum nigrum	94.27	262
PTU_256	KY652843	Solanum nigrum	93.89	262
PTU_256	316986388	Capsicum frutescens	97.67	215
PTU_257	KF454079	Convolvulus arvensis	98.28	291
PTU_257	KF454077	Convolvulus arvensis	98.28	291
PTU_257	KF454072	Convolvulus arvensis	97.94	291
PTU_257	586598656	Convolvulus arvensis	98.11	212
PTU_258	KX096056	Ocimum americanum	92.39	276
PTU_258	KX096059	Ocimum basilicum	92.00	275
PTU_258	KF454238	Ocimum basilicum	91.34	277
PTU_258	KX534371	Ocimum basilicum	80.89	293
PTU_259	KF454049	Solanum nigrum	86.85	251
PTU_259	KY652843	Solanum nigrum	86.45	251
PTU_259	KX166238	Solanum nigrum	86.45	251
PTU_259	KF454097	Lycium barbarum	84.76	269
PTU_260	KY652843	Solanum nigrum	94.66	131
PTU_260	KX282258	Lycium shawii	94.53	128
PTU_260	KX166238	Solanum nigrum	93.89	131
PTU_260	KF454097	Lycium barbarum	94.53	128

qseqid	sseqid	stitle	pident	length
PTU_261	KF454118	Origanum vulgare	96.35	301
PTU_261	KX166553	Thymus pulegioides	90.43	303
PTU_261	KF454518	Thymus marschallianus	90.40	302
PTU_261	KF454516	Thymus altaicus	90.40	302
PTU_262	KX166238	Solanum nigrum	94.68	263
PTU_262	KF454049	Solanum nigrum	94.68	263
PTU_262	KY652843	Solanum nigrum	94.30	263
PTU_262	KX282258	Lycium shawii	90.72	291
PTU_263	KX096059	Ocimum basilicum	92.25	271
PTU_263	KX096056	Ocimum americanum	91.94	273
PTU_263	KF454238	Ocimum basilicum	92.72	261
PTU_263	KU203796	Damrongia orientalis	97.80	91
PTU_264	24306624	Salvia pachyphylla	84.31	102
PTU_264	24306625	Salvia pachyphylla	84.31	102
PTU_265	KF454518	Thymus marschallianus	95.36	302
PTU_265	KX166553	Thymus pulegioides	95.05	303
PTU_265	KC295045	Thymus mongolicus	95.03	302
PTU_265	KF454516	Thymus altaicus	94.70	302
PTU_266	KF454118	Origanum vulgare	97.01	301
PTU_266	KX166553	Thymus pulegioides	90.43	303
PTU_266	KF454518	Thymus marschallianus	90.40	302
PTU_266	KF454516	Thymus altaicus	90.40	302
PTU_267	KF454079	Convolvulus arvensis	97.94	291
PTU_267	KF454077	Convolvulus arvensis	97.94	291
PTU_267	KF454072	Convolvulus arvensis	97.59	291
PTU_267	586598656	Convolvulus arvensis	97.64	212
PTU_268	KX166292	Knautia arvensis	96.22	291

qseqid	sseqid	stitle	pident	length
PTU_268	594615212	Knautia degenii	100.00	207
PTU_268	594615213	Knautia degenii	100.00	207
PTU_268	594615207	Knautia integrifolia	98.55	207
PTU_269	LT593966	Brachiaria fragrans	91.64	287
PTU_269	KX282323	Cenchrus ramosissimus	91.67	276
PTU_269	KX282065	Cenchrus ciliaris	90.94	287
PTU_269	KY968838	Melinis repens	90.24	287
PTU_270	KF241280	Arnebia euchroma	98.62	289
PTU_270	KX166735	Tragopogon pratensis	97.92	289
PTU_270	126153720	Tragopogon dubius	100.00	211
PTU_270	290796685	Tragopogon dubius	100.00	211
PTU_271	KX096059	Ocimum basilicum	91.76	267
PTU_271	KX096056	Ocimum americanum	91.45	269
PTU_271	KF454238	Ocimum basilicum	90.74	270
PTU_271	JF301414	Martianthus leucocephalus	79.41	272
PTU_272	KX282069	Centaurea mesopotamica	95.74	282
PTU_272	KX282072	Centaurea pseudosinaica	94.68	282
PTU_272	KX282071	Centaurea pseudosinaica	94.68	282
PTU_272	KX166785	Centaurea calcitrapa	94.33	282
PTU_273	KX166238	Solanum nigrum	94.27	262
PTU_273	KF454049	Solanum nigrum	94.27	262
PTU_273	KY652843	Solanum nigrum	93.89	262
PTU_273	316986388	Capsicum frutescens	98.14	215
PTU_274	KX166238	Solanum nigrum	92.02	263
PTU_274	KF454049	Solanum nigrum	92.02	263
PTU_274	KY652843	Solanum nigrum	91.63	263
PTU_274	KX282258	Lycium shawii	91.51	259

qseqid	sseqid	stitle	pident	length
PTU_275	KX166238	Solanum nigrum	93.10	261
PTU_275	KF454049	Solanum nigrum	93.10	261
PTU_275	KY652843	Solanum nigrum	92.72	261
PTU_275	KX282258	Lycium shawii	91.09	258
PTU_276	56407380	Physalis angulata	99.01	202
PTU_276	56407422	Physalis pubescens	97.51	201
PTU_276	56407421	Physalis pubescens	97.01	201
PTU_276	KU724227	Withania somnifera	89.67	271
PTU_277	KX166814	Polygonum arenastrum	98.12	266
PTU_277	KF530289	Polygonum aviculare	95.11	266
PTU_277	KX166588	Polygonum aviculare	94.74	266
PTU_277	284811074	Polygonum aviculare	98.91	183
PTU_278	KX166238	Solanum nigrum	95.44	263
PTU_278	KF454049	Solanum nigrum	95.44	263
PTU_278	KY652843	Solanum nigrum	94.68	263
PTU_278	KX282258	Lycium shawii	92.88	267
PTU_279	KX282189	Helianthemum lippii	93.94	264
PTU_279	KX282184	Helianthemum kahiricum	93.94	264
PTU_279	GU327673	Helianthemum syriacum	93.94	264
PTU_279	GU327674	Helianthemum syriacum	93.56	264
PTU_280	KF241280	Arnebia euchroma	97.60	292
PTU_280	KX166735	Tragopogon pratensis	96.92	292
PTU_280	317451625	Tragopogon lainzii	100.00	213
PTU_280	317451630	Tragopogon lainzii	100.00	213
PTU_281	KX166238	Solanum nigrum	92.34	261
PTU_281	KF454049	Solanum nigrum	92.34	261
PTU_281	KY652843	Solanum nigrum	91.95	261

qseqid	sseqid	stitle	pident	length
PTU_281	KX282258	Lycium shawii	90.31	258
PTU_282	KX166151	Poa trivialis	99.29	281
PTU_282	KT948627	Alopecurus myosuroides	98.93	281
PTU_282	KX166725	Poa compressa	95.70	279
PTU_282	KX166880	Poa nemoralis	95.34	279
PTU_283	KX166238	Solanum nigrum	93.54	263
PTU_283	KF454049	Solanum nigrum	93.54	263
PTU_283	KY652843	Solanum nigrum	92.78	263
PTU_283	KX346971	Solanum lyratum	92.34	261
PTU_284	X86848	Silene dichotoma	97.22	288
PTU_284	KY624401	Vaccaria hispanica	97.54	285
PTU_284	KX166380	Silene vulgaris	97.81	274
PTU_284	X86851	Silene fabaria	97.45	274
PTU_285	KX282189	Helianthemum lippii	93.56	264
PTU_285	KX282184	Helianthemum kahiricum	93.56	264
PTU_285	GU327673	Helianthemum syriacum	93.56	264
PTU_285	GU327674	Helianthemum syriacum	93.18	264
PTU_286	KX167359	Melica nutans	98.25	285
PTU_286	KX167318	Melica uniflora	96.14	285
PTU_286	685210821	Melica ciliata	99.03	206
PTU_286	356649999	Melica picta	98.03	203
PTU_287	KX166238	Solanum nigrum	95.44	263
PTU_287	KF454049	Solanum nigrum	95.44	263
PTU_287	KY652843	Solanum nigrum	94.68	263
PTU_287	KU724227	Withania somnifera	93.16	263
PTU_288	KX166238	Solanum nigrum	94.30	263
PTU_288	KF454049	Solanum nigrum	94.30	263

qseqid	sseqid	stitle	pident	length
PTU_288	KY652843	Solanum nigrum	93.54	263
PTU_288	KX346971	Solanum lyratum	92.75	262
PTU_289	KX282189	Helianthemum lippii	93.18	264
PTU_289	KX282184	Helianthemum kahiricum	93.18	264
PTU_289	GU327673	Helianthemum syriacum	93.18	264
PTU_289	GU327674	Helianthemum syriacum	92.80	264
PTU_290	KX166595	Dianthus deltoides	94.68	282
PTU_290	JF421492	Dianthus chinensis	94.31	281
PTU_290	JF421491	Dianthus chinensis	93.95	281
PTU_290	KX167086	Dianthus armeria	93.62	282
PTU_291	KF241280	Arnebia euchroma	98.28	291
PTU_291	KX166735	Tragopogon pratensis	97.59	291
PTU_291	317451650	Tragopogon porrifolius	98.57	210
PTU_291	317451649	Tragopogon porrifolius	98.57	210
PTU_292	KU350154	Arbutus canariensis	97.33	300
PTU_292	KX165505	Arctous alpina	89.07	302
PTU_292	KU350164	Comarostaphylis diversifolia	88.67	300
PTU_292	KF419121	Comarostaphylis arbutoides	88.74	293
PTU_293	37650547	Olea europaea subsp. europaea	98.07	207
PTU_293	460109779	Olea europaea subsp. europaea	97.09	206
PTU_293	560592114	Olea europaea	97.35	189
PTU_293	460109844	Olea europaea subsp. cuspidata	95.36	194
PTU_294	KX096059	Ocimum basilicum	91.24	274
PTU_294	KX096056	Ocimum americanum	90.58	276
PTU_294	KF454238	Ocimum basilicum	89.89	277
PTU_294	KU203783	Dorcoceras hygrometricum	94.44	90
PTU_295	KY652843	Solanum nigrum	92.61	257

qseqid	sseqid	stitle	pident	length
PTU_295	KX166238	Solanum nigrum	92.22	257
PTU_295	KF454049	Solanum nigrum	92.22	257
PTU_295	KF454093	Lycium ruthenicum	89.01	273
PTU_296	KX166431	Sorbus leyana	91.93	285
PTU_296	KX166119	Sorbus minima	91.93	285
PTU_296	KX165881	Sorbus cuneifolia	91.93	285
PTU_296	KX166102	Sorbus pseudofennica	91.58	285
PTU_297	JF831213	Muehlenbeckia gracillima	95.40	261
PTU_297	JF831210	K.L.Wilson & Makinson	95.00	260
PTU_297	JF831221	K.L.Wilson & Makinson	92.42	264
PTU_297	JF831211	Muehlenbeckia diclina	92.75	262
PTU_298	MF171486	Ipomoea carnea	91.79	280
PTU_298	MF171643	Ipomoea marabensis	92.54	268
PTU_298	MF171725	Ipomoea marabensis	92.16	268
PTU_298	MF171594	Ipomoea cavalcantei	92.16	268
PTU_299	KX166238	Solanum nigrum	94.64	261
PTU_299	KF454049	Solanum nigrum	94.64	261
PTU_299	KY652843	Solanum nigrum	94.25	261
PTU_299	KX282258	Lycium shawii	93.02	258
PTU_300	KX166238	Solanum nigrum	94.62	260
PTU_300	KF454049	Solanum nigrum	94.62	260
PTU_300	KY652843	Solanum nigrum	94.23	260
PTU_300	KX282258	Lycium shawii	93.00	257
PTU_301	KX096059	Ocimum basilicum	91.51	271
PTU_301	KX096056	Ocimum americanum	89.49	276
PTU_301	KF454238	Ocimum basilicum	89.53	277
PTU_301	KU203783	Dorcoceras hygrometricum	100.00	86

qseqid	sseqid	stitle	pident	length
PTU_302	KX165891	Sanguisorba officinalis	94.16	274
PTU_302	JF421542	Sanguisorba officinalis	93.80	274
PTU_302	JF421541	Sanguisorba officinalis	93.80	274
PTU_302	KX166425	Potentilla erecta	91.30	276
PTU_303	KF454238	Ocimum basilicum	96.55	174
PTU_303	KX096059	Ocimum basilicum	94.74	171
PTU_304	KX165883	Cirsium arvense	100.00	291
PTU_304	KX167061	Cirsium tuberosum	97.58	289
PTU_304	KX167382	Cirsium acaule	97.23	289
PTU_304	KX167205	Cirsium dissectum	97.23	289
PTU_305	KX282324	Phragmites australis	98.22	281
PTU_305	LC085881	Phragmites japonicus	97.51	281
PTU_305	KX167579	Molinia caerulea	97.81	274
PTU_305	HQ329791	Eragrostis walteri	94.81	270
PTU_306	KF454118	Origanum vulgare	96.68	301
PTU_306	KX166553	Thymus pulegioides	91.09	303
PTU_306	KF454518	Thymus marschallianus	91.06	302
PTU_306	KF454516	Thymus altaicus	91.06	302
PTU_307	KX166238	Solanum nigrum	92.37	262
PTU_307	KF454049	Solanum nigrum	92.37	262
PTU_307	KY652843	Solanum nigrum	91.98	262
PTU_307	316986388	Capsicum frutescens	96.28	215
PTU_308	KX166238	Solanum nigrum	93.46	260
PTU_308	KF454049	Solanum nigrum	93.46	260
PTU_308	KY652843	Solanum nigrum	93.08	260
PTU_308	316986388	Capsicum frutescens	98.14	215
PTU_309	KX282181	Haloxylon salicornicum	91.70	277

qseqid	sseqid	stitle	pident	length
PTU_309	KX282180	Haloxylon salicornicum	91.70	277
PTU_309	KX282182	Haloxylon salicornicum	91.34	277
PTU_309	KX282091	Cornulaca monacantha	90.61	277
PTU_310	KM210331	Lavandula angustifolia	87.23	282
PTU_310	KX282394	Salvia spinosa	85.66	279
PTU_310	KX166755	Salvia pratensis	85.61	278
PTU_310	KF454271	Lycopus europaeus	85.00	280
PTU_311	KF454118	Origanum vulgare	98.68	302
PTU_311	KX166553	Thymus pulegioides	91.75	303
PTU_311	KF454518	Thymus marschallianus	91.72	302
PTU_311	KC295045	Thymus mongolicus	91.39	302
PTU_312	KF454118	Origanum vulgare	97.01	301
PTU_312	KX166553	Thymus pulegioides	90.73	302
PTU_312	KF454518	Thymus marschallianus	90.70	301
PTU_312	KF454516	Thymus altaicus	90.73	302
PTU_313	KX166238	Solanum nigrum	90.87	263
PTU_313	KF454049	Solanum nigrum	90.87	263
PTU_313	KY652843	Solanum nigrum	90.49	263
PTU_313	316986388	Capsicum frutescens	95.35	215
PTU_314	KX096059	Ocimum basilicum	89.05	274
PTU_314	KX096056	Ocimum americanum	88.77	276
PTU_314	KF454238	Ocimum basilicum	88.09	277
PTU_314	KU203783	Dorcoceras hygrometricum	94.38	89
PTU_315	KX166238	Solanum nigrum	91.22	262
PTU_315	KF454049	Solanum nigrum	91.22	262
PTU_315	KY652843	Solanum nigrum	90.84	262
PTU_315	KX346971	Solanum lyratum	88.93	262

qseqid	sseqid	stitle	pident	length
PTU_316	KX166238	Solanum nigrum	94.68	263
PTU_316	KF454049	Solanum nigrum	94.68	263
PTU_316	KY652843	Solanum nigrum	93.92	263
PTU_316	KF454093	Lycium ruthenicum	91.94	273
PTU_317	KF454079	Convolvulus arvensis	98.97	291
PTU_317	KF454077	Convolvulus arvensis	98.97	291
PTU_317	KF454072	Convolvulus arvensis	98.63	291
PTU_317	586598656	Convolvulus arvensis	98.11	212
PTU_318	KF454449	Xanthium strumarium	99.66	293
PTU_318	KF241289	Xanthium sibiricum	99.32	293
PTU_318	KF454446	Xanthium sibiricum	98.63	293
PTU_318	KF767534	Helianthus annuus	92.65	272
PTU_319	KF241280	Arnebia euchroma	98.28	291
PTU_319	KX166735	Tragopogon pratensis	97.59	291
PTU_319	317451650	Tragopogon porrifolius	98.57	210
PTU_319	317451649	Tragopogon porrifolius	98.57	210
PTU_320	KX096059	Ocimum basilicum	92.28	272
PTU_320	KX096056	Ocimum americanum	90.91	275
PTU_320	KF454238	Ocimum basilicum	90.58	276
PTU_320	KU203783	Dorcoceras hygrometricum	96.59	88
PTU_321	KX166238	Solanum nigrum	92.02	263
PTU_321	KF454049	Solanum nigrum	92.02	263
PTU_321	KY652843	Solanum nigrum	91.63	263
PTU_321	316986388	Capsicum frutescens	95.79	214
PTU_322	KX166479	Taraxacum sp. CF-2016	100.00	294
PTU_322	KY860926	Taraxacum officinale	99.66	294
PTU_322	KX166811	Taraxacum sp. CF-2016	99.66	294

qseqid	sseqid	stitle	pident	length
PTU_322	KY671128	Taraxacum amplum	99.32	294
PTU_323	KX096056	Ocimum americanum	93.48	276
PTU_323	KX096059	Ocimum basilicum	93.45	275
PTU_323	KF454238	Ocimum basilicum	92.42	277
PTU_323	HF542817	Capurodendron sp. US-2012	96.70	91
PTU_324	KX166238	Solanum nigrum	95.06	263
PTU_324	KF454049	Solanum nigrum	95.06	263
PTU_324	KY652843	Solanum nigrum	94.68	263
PTU_324	316986388	Capsicum frutescens	99.07	215
PTU_325	KF454238	Ocimum basilicum	92.23	206
PTU_325	KF454238	Ocimum basilicum	100.00	48
PTU_325	KX096056	Ocimum americanum	91.71	205
PTU_325	KX096056	Ocimum americanum	100.00	48
PTU_325	KX096059	Ocimum basilicum	90.20	204
PTU_325	KX096059	Ocimum basilicum	100.00	48
PTU_325	380719853	Plectranthus caninus	93.07	101
PTU_326	KX096056	Ocimum americanum	90.84	273
PTU_326	KX096059	Ocimum basilicum	90.44	272
PTU_326	KF454238	Ocimum basilicum	91.12	259
PTU_326	KY858250	Alsobia dianthiflora	78.66	253
PTU_327	KX096059	Ocimum basilicum	97.06	272
PTU_327	KF454238	Ocimum basilicum	94.22	277
PTU_327	KX096056	Ocimum americanum	93.14	277
PTU_327	JF301414	Martianthus leucocephalus	82.35	272
PTU_328	KF454072	Convolvulus arvensis	97.94	291
PTU_328	KF454079	Convolvulus arvensis	97.59	291
PTU_328	KF454077	Convolvulus arvensis	97.59	291

qseqid	sseqid	stitle	pident	length
PTU_328	586598656	Convolvulus arvensis	98.11	212
PTU_329	KX166238	Solanum nigrum	90.87	263
PTU_329	KF454049	Solanum nigrum	90.87	263
PTU_329	KY652843	Solanum nigrum	90.49	263
PTU_329	316986388	Capsicum frutescens	95.35	215
PTU_330	KX165842	Linaria vulgaris	98.95	285
PTU_330	KT031859	Linaria genistifolia	98.60	285
PTU_330	KT031864	Linaria japonica	98.25	285
PTU_330	KT031866	Linaria michauxii	97.89	285
PTU_331	KX166238	Solanum nigrum	93.00	257
PTU_331	KF454049	Solanum nigrum	93.00	257
PTU_331	KY652843	Solanum nigrum	92.61	257
PTU_331	316986388	Capsicum frutescens	96.26	214
PTU_332	KX166238	Solanum nigrum	94.92	256
PTU_332	KF454049	Solanum nigrum	94.92	256
PTU_332	KY652843	Solanum nigrum	94.53	256
PTU_332	KX282258	Lycium shawii	92.52	254
PTU_333	KX166238	Solanum nigrum	93.10	261
PTU_333	KY652843	Solanum nigrum	92.72	261
PTU_333	KF454049	Solanum nigrum	92.72	261
PTU_333	KX656898	Lycium barbarum	90.98	255
PTU_334	KX165689	Dactylis glomerata	98.58	282
PTU_334	KX166349	Festuca arundinacea	91.70	277
PTU_334	KP205450	Festuca arundinacea	91.70	277
PTU_334	KX166352	Festuca ovina	91.94	273
PTU_335	KX166151	Poa trivialis	99.64	281
PTU_335	KT948627	Alopecurus myosuroides	99.29	281

qseqid	sseqid	stitle	pident	length
PTU_335	KX166725	Poa compressa	96.06	279
PTU_335	KX166880	Poa nemoralis	95.70	279
PTU_336	KX166238	Solanum nigrum	93.16	263
PTU_336	KF454049	Solanum nigrum	93.16	263
PTU_336	KY652843	Solanum nigrum	92.78	263
PTU_336	KF454093	Lycium ruthenicum	90.44	272
PTU_337	KX166238	Solanum nigrum	92.37	262
PTU_337	KF454049	Solanum nigrum	92.37	262
PTU_337	KY652843	Solanum nigrum	91.98	262
PTU_337	316986388	Capsicum frutescens	95.81	215
PTU_338	KX166238	Solanum nigrum	94.70	264
PTU_338	KF454049	Solanum nigrum	94.30	263
PTU_338	KY652843	Solanum nigrum	93.94	264
PTU_338	KF454093	Lycium ruthenicum	93.44	259
PTU_339	KF454118	Origanum vulgare	97.35	302
PTU_339	KX166553	Thymus pulegioides	90.43	303
PTU_339	KF454518	Thymus marschallianus	90.40	302
PTU_339	KC295045	Thymus mongolicus	90.07	302
PTU_340	KX166238	Solanum nigrum	93.54	263
PTU_340	KF454049	Solanum nigrum	93.54	263
PTU_340	KY652843	Solanum nigrum	93.16	263
PTU_340	316986388	Capsicum frutescens	97.20	214
PTU_341	KX166238	Solanum nigrum	92.78	263
PTU_341	KF454049	Solanum nigrum	92.78	263
PTU_341	KY652843	Solanum nigrum	92.40	263
PTU_341	316986388	Capsicum frutescens	97.20	214
PTU_342	KX166238	Solanum nigrum	94.25	261

qseqid	sseqid	stitle	pident	length
PTU_342	KF454049	Solanum nigrum	94.25	261
PTU_342	KY652843	Solanum nigrum	93.49	261
PTU_342	KX282258	Lycium shawii	92.25	258
PTU_343	KF454118	Origanum vulgare	96.36	302
PTU_343	KX166553	Thymus pulegioides	90.46	304
PTU_343	KF454518	Thymus marschallianus	90.43	303
PTU_343	KF454516	Thymus altaicus	90.43	303
PTU_344	KX096059	Ocimum basilicum	92.67	273
PTU_344	KX096056	Ocimum americanum	91.64	275
PTU_344	KF454238	Ocimum basilicum	90.58	276
PTU_344	KU203796	Damrongia orientalis	98.90	91
PTU_345	KF530289	Polygonum aviculare	100.00	266
PTU_345	KX166588	Polygonum aviculare	99.62	266
PTU_345	KX166814	Polygonum arenastrum	94.72	265
PTU_345	392612351	Polygonum aviculare	100.00	187
PTU_346	KX166238	Solanum nigrum	94.66	262
PTU_346	KF454049	Solanum nigrum	94.66	262
PTU_346	KY652843	Solanum nigrum	93.89	262
PTU_346	KF454093	Lycium ruthenicum	93.41	258
PTU_347	KX166238	Solanum nigrum	94.68	263
PTU_347	KF454049	Solanum nigrum	94.68	263
PTU_347	KY652843	Solanum nigrum	93.92	263
PTU_347	KF454093	Lycium ruthenicum	91.94	273
PTU_348	KF454238	Ocimum basilicum	96.38	276
PTU_348	KX096056	Ocimum americanum	96.00	275
PTU_348	KX096059	Ocimum basilicum	94.89	274
PTU_348	KY858250	Alsobia dianthiflora	95.28	106

qseqid	sseqid	stitle	pident	length
PTU_349	KX166292	Knautia arvensis	96.18	288
PTU_349	594615212	Knautia degenii	100.00	207
PTU_349	594615213	Knautia degenii	100.00	207
PTU_349	594615207	Knautia integrifolia	98.55	207
PTU_350	KJ131560	Triticum turgidum	100.00	285
PTU_350	KJ131569	Triticum turgidum	99.65	285
PTU_350	KJ131565	Triticum aestivum	99.65	285
PTU_350	KJ131556	Triticum turgidum	99.65	285
PTU_351	KX166238	Solanum nigrum	93.54	263
PTU_351	KY652843	Solanum nigrum	93.16	263
PTU_351	KF454049	Solanum nigrum	93.16	263
PTU_351	316986388	Capsicum frutescens	97.20	214
PTU_352	KX166495	Logfia gallica	97.56	287
PTU_352	KX166280	Gnaphalium supinum	97.56	287
PTU_352	KX282156	Filago pyramidata	97.56	287
PTU_352	KX166911	Logfia minima	97.21	287
PTU_353	AB851489	Erigeron annuus	95.57	271
PTU_353	KX166456	Erigeron borealis	94.07	270
PTU_353	4731984	Conyza bonariensis	99.48	193
PTU_353	4731987	Erigeron rosulatus	97.93	193
PTU_354	KX282324	Phragmites australis	98.58	281
PTU_354	LC085881	Phragmites japonicus	97.86	281
PTU_354	KX167579	Molinia caerulea	98.18	274
PTU_354	HQ329791	Eragrostis walteri	95.19	270
PTU_355	KM210331	Lavandula angustifolia	86.83	281
PTU_355	KT220701	Perilla setoyensis	84.53	278
PTU_355	KX166755	Salvia pratensis	84.12	277

qseqid	sseqid	stitle	pident	length
PTU_355	KT220699	Perilla citriodora	84.34	281
PTU_356	KX166238	Solanum nigrum	96.09	256
PTU_356	KF454049	Solanum nigrum	96.09	256
PTU_356	KY652843	Solanum nigrum	95.31	256
PTU_356	KF454093	Lycium ruthenicum	92.28	272
PTU_357	KF454296	Daucus carota	91.47	293
PTU_357	KF454292	Daucus carota	91.13	293
PTU_357	FJ150184	Daucus carota	91.29	287
PTU_357	X17534	Daucus carota	90.78	293
PTU_358	KX282394	Salvia spinosa	98.59	284
PTU_358	KX282393	Salvia spinosa	97.54	284
PTU_358	KX166755	Salvia pratensis	96.84	285
PTU_358	KX282392	Salvia lanigera	96.14	285
PTU_359	KX282291	Nitraria retusa	86.18	275
PTU_359	KX282289	Nitraria retusa	86.18	275
PTU_359	83596260	Nitraria roborowskii	91.11	180
PTU_359	82734708	Nitraria praevisa	90.56	180
PTU_360	KX166238	Solanum nigrum	92.02	263
PTU_360	KF454049	Solanum nigrum	92.02	263
PTU_360	KY652843	Solanum nigrum	91.63	263
PTU_360	KX346971	Solanum lyratum	90.08	262
PTU_361	KT031859	Linaria genistifolia	99.65	285
PTU_361	KX165842	Linaria vulgaris	99.30	285
PTU_361	KT031866	Linaria michauxii	98.95	285
PTU_361	KT031870	Linaria salangensis	98.60	285
PTU_362	KF454072	Convolvulus arvensis	98.28	291
PTU_362	KF454079	Convolvulus arvensis	97.94	291

qseqid	sseqid	stitle	pident	length
PTU_362	KF454077	Convolvulus arvensis	97.94	291
PTU_362	586598656	Convolvulus arvensis	98.58	212
PTU_363	KF454049	Solanum nigrum	95.06	263
PTU_363	KX166238	Solanum nigrum	94.30	263
PTU_363	KY652843	Solanum nigrum	93.92	263
PTU_363	316986388	Capsicum frutescens	98.14	215
PTU_364	KX166238	Solanum nigrum	94.30	263
PTU_364	KF454049	Solanum nigrum	94.30	263
PTU_364	KY652843	Solanum nigrum	93.92	263
PTU_364	316986388	Capsicum frutescens	98.60	215
PTU_365	KX165886	Bromus erectus	98.58	281
PTU_365	KX165557	Bromus sterilis	97.51	281
PTU_365	KX282048	Bromus tectorum	97.15	281
PTU_365	KX282047	Bromus tectorum	97.15	281
PTU_366	KX166238	Solanum nigrum	93.89	262
PTU_366	KF454049	Solanum nigrum	93.89	262
PTU_366	KY652843	Solanum nigrum	93.13	262
PTU_366	KF454093	Lycium ruthenicum	91.54	272
PTU_367	KF454118	Origanum vulgare	93.09	304
PTU_367	KX166553	Thymus pulegioides	92.46	305
PTU_367	KF454518	Thymus marschallianus	92.43	304
PTU_367	KF454516	Thymus altaicus	92.08	303
PTU_368	KX166238	Solanum nigrum	94.30	263
PTU_368	KF454049	Solanum nigrum	94.30	263
PTU_368	KY652843	Solanum nigrum	93.92	263
PTU_368	316986388	Capsicum frutescens	98.14	215
PTU_369	KX096056	Ocimum americanum	93.45	275

qseqid	sseqid	stitle	pident	length
PTU_369	KX096059	Ocimum basilicum	93.07	274
PTU_369	KF454238	Ocimum basilicum	92.39	276
PTU_369	FM163243	Callicarpa furfuracea	79.23	284
PTU_370	26190454	Nepeta cataria	85.37	123
PTU_370	26190448	Nepeta crassifolia	85.12	121
PTU_370	67078901	Justicia americana	84.44	90
PTU_370	68235657	Aphelandra castanifolia	84.21	95
PTU_371	KX282107	Dichanthium annulatum	92.28	285
PTU_371	KX689356	Chrysopogon argutus	92.28	285
PTU_371	KX282217	Imperata cylindrica	92.28	285
PTU_371	KF184927	Saccharum hybrid cultivar	92.28	285
PTU_372	KX166238	Solanum nigrum	92.78	263
PTU_372	KF454049	Solanum nigrum	92.78	263
PTU_372	KY652843	Solanum nigrum	92.40	263
PTU_372	316986388	Capsicum frutescens	96.26	214
PTU_373	KX166238	Solanum nigrum	93.94	264
PTU_373	KF454049	Solanum nigrum	93.54	263
PTU_373	KY652843	Solanum nigrum	93.18	264
PTU_373	KF454093	Lycium ruthenicum	91.58	273
PTU_374	KX166238	Solanum nigrum	93.92	263
PTU_374	KF454049	Solanum nigrum	93.92	263
PTU_374	KY652843	Solanum nigrum	93.16	263
PTU_374	KX346971	Solanum lyratum	92.37	262
PTU_375	KX166238	Solanum nigrum	93.92	263
PTU_375	KF454049	Solanum nigrum	93.92	263
PTU_375	KY652843	Solanum nigrum	93.16	263
PTU_375	KF454093	Lycium ruthenicum	91.58	273

qseqid	sseqid	stitle	pident	length
PTU_376	KX282401	Lomelosia palestina	99.33	297
PTU_376	KX282400	Lomelosia palestina	98.64	295
PTU_376	KX282398	Lomelosia olivieri	96.61	295
PTU_376	KX166055	Scabiosa columbaria	95.61	296
PTU_377	KX166238	Solanum nigrum	92.75	262
PTU_377	KF454049	Solanum nigrum	92.75	262
PTU_377	KY652843	Solanum nigrum	92.37	262
PTU_377	KX282258	Lycium shawii	90.77	260
PTU_378	KX282189	Helianthemum lippii	93.18	264
PTU_378	KX282184	Helianthemum kahiricum	93.18	264
PTU_378	GU327673	Helianthemum syriacum	92.42	264
PTU_378	GU327674	Helianthemum syriacum	92.05	264
PTU_379	KX166238	Solanum nigrum	93.92	263
PTU_379	KF454049	Solanum nigrum	93.92	263
PTU_379	KY652843	Solanum nigrum	93.16	263
PTU_379	KF454093	Lycium ruthenicum	91.58	273
PTU_380	KX166238	Solanum nigrum	93.16	263
PTU_380	KF454049	Solanum nigrum	93.16	263
PTU_380	KY652843	Solanum nigrum	92.40	263
PTU_380	KF454093	Lycium ruthenicum	90.84	273
PTU_381	AB851489	Erigeron annuus	95.57	271
PTU_381	KX166456	Erigeron borealis	94.81	270
PTU_381	4731984	Conyza bonariensis	100.00	193
PTU_381	4731987	Erigeron rosulatus	98.45	193
PTU_382	KX166238	Solanum nigrum	91.44	257
PTU_382	KF454049	Solanum nigrum	91.44	257
PTU_382	KY652843	Solanum nigrum	91.05	257

qseqid	sseqid	stitle	pident	length
PTU_382	316986388	Capsicum frutescens	93.95	215
PTU_383	KX096056	Ocimum americanum	90.22	276
PTU_383	KX096059	Ocimum basilicum	90.15	274
PTU_383	KF454238	Ocimum basilicum	89.89	277
PTU_383	380719853	Plectranthus caninus	95.00	100
PTU_384	KX282189	Helianthemum lippii	92.80	264
PTU_384	KX282184	Helianthemum kahiricum	92.80	264
PTU_384	GU327673	Helianthemum syriacum	92.05	264
PTU_384	GU327674	Helianthemum syriacum	91.67	264
PTU_385	KX096059	Ocimum basilicum	94.14	273
PTU_385	KF454238	Ocimum basilicum	93.14	277
PTU_385	KX096056	Ocimum americanum	92.73	275
PTU_385	JF301414	Martianthus leucocephalus	81.32	273
PTU_386	KJ131562	Aegilops biuncialis	98.60	285
PTU_386	KX281956	Aegilops kotschyi	98.25	285
PTU_386	KJ131563	Aegilops triuncialis	98.25	285
PTU_386	KX281958	Aegilops triuncialis	97.89	285
PTU_387	KF241289	Xanthium sibiricum	100.00	293
PTU_387	KF454449	Xanthium strumarium	99.66	293
PTU_387	KF454446	Xanthium sibiricum	99.32	293
PTU_387	KF767534	Helianthus annuus	91.47	293
PTU_388	KX282189	Helianthemum lippii	93.56	264
PTU_388	KX282184	Helianthemum kahiricum	93.56	264
PTU_388	GU327673	Helianthemum syriacum	93.18	264
PTU_388	KC698933	Helianthemum hirtum	93.16	263
PTU_389	LT593966	Brachiaria fragrans	92.22	270
PTU_389	KX689291	Digitaria horizontalis	92.19	269

qseqid	sseqid	stitle	pident	length
PTU_389	LT593967	Chasechloa egregia	91.08	269
PTU_389	LT593967	Chasechloa egregia	91.08	269
PTU_390	166062977	Echinochloa crus-galli var. formosensis	100.00	203
PTU_390	166062976	Echinochloa crus-galli var. praticola	100.00	203
PTU_390	157886934	Echinochloa crus-galli	99.03	207
PTU_390	7271144	Echinochloa colona	99.03	207
PTU_391	KX282217	Imperata cylindrica	92.96	284
PTU_391	KX282107	Dichanthium annulatum	92.25	284
PTU_391	KF184927	Saccharum hybrid cultivar	91.93	285
PTU_391	KX689356	Chrysopogon argutus	90.53	285
PTU_392	KX165886	Bromus erectus	97.86	281
PTU_392	KX165557	Bromus sterilis	96.80	281
PTU_392	KX282048	Bromus tectorum	96.44	281
PTU_392	KX282047	Bromus tectorum	96.44	281
PTU_393	86197791	Scorzonera laciniata	99.51	205
PTU_393	86197790	Scorzonera cana	99.02	204
PTU_393	KF454416	Scorzonera parviflora	87.85	288
PTU_393	KF454420	Scorzonera mongolica	88.73	275
PTU_394	KF454118	Origanum vulgare	95.68	301
PTU_394	KX166553	Thymus pulegioides	90.10	303
PTU_394	KF454518	Thymus marschallianus	90.07	302
PTU_394	KF454516	Thymus altaicus	90.10	303
PTU_395	KX166238	Solanum nigrum	92.72	261
PTU_395	KY652843	Solanum nigrum	92.34	261
PTU_395	KF454049	Solanum nigrum	92.34	261
PTU_395	KX656898	Lycium barbarum	90.59	255
PTU_396	KX166238	Solanum nigrum	93.89	262

qseqid	sseqid	stitle	pident	length
PTU_396	KF454049	Solanum nigrum	93.89	262
PTU_396	KY652843	Solanum nigrum	93.51	262
PTU_396	KX282258	Lycium shawii	91.73	266
PTU_397	KX166238	Solanum nigrum	95.06	263
PTU_397	KF454049	Solanum nigrum	95.06	263
PTU_397	KY652843	Solanum nigrum	94.68	263
PTU_397	316986388	Capsicum frutescens	99.07	215
PTU_398	KX166151	Poa trivialis	99.29	281
PTU_398	KT948627	Alopecurus myosuroides	98.93	281
PTU_398	KX166725	Poa compressa	95.70	279
PTU_398	KX166880	Poa nemoralis	95.34	279
PTU_399	KX166238	Solanum nigrum	91.37	255
PTU_399	KY652843	Solanum nigrum	90.98	255
PTU_399	KF454049	Solanum nigrum	90.98	255
PTU_399	KX282258	Lycium shawii	88.76	258
PTU_400	KX096056	Ocimum americanum	93.45	275
PTU_400	KX096059	Ocimum basilicum	93.43	274
PTU_400	KF454238	Ocimum basilicum	92.39	276
PTU_400	HF542817	Capurodendron sp. US-2012	96.70	91
PTU_401	KF454118	Origanum vulgare	97.68	302
PTU_401	KX166553	Thymus pulegioides	91.42	303
PTU_401	KF454518	Thymus marschallianus	91.39	302
PTU_401	KC295045	Thymus mongolicus	91.06	302
PTU_402	KF454238	Ocimum basilicum	98.56	277
PTU_402	KX096056	Ocimum americanum	97.48	278
PTU_402	KX096059	Ocimum basilicum	96.39	277
PTU_402	JF301414	Martianthus leucocephalus	83.52	273

qseqid	sseqid	stitle	pident	length
PTU_403	KF454118	Origanum vulgare	97.01	301
PTU_403	KX166553	Thymus pulegioides	91.09	303
PTU_403	KF454518	Thymus marschallianus	91.06	302
PTU_403	KF454516	Thymus altaicus	91.06	302
PTU_404	KX282108	Digitaria ciliaris	98.93	280
PTU_404	KX689291	Digitaria horizontalis	97.86	280
PTU_404	480312832	Salvia tesquicola	100.00	201
PTU_404	157886935	Digitaria sanguinalis	100.00	201
PTU_405	KX166238	Solanum nigrum	93.10	261
PTU_405	KF454049	Solanum nigrum	93.10	261
PTU_405	KY652843	Solanum nigrum	92.34	261
PTU_405	KX346971	Solanum lyratum	91.54	260
PTU_406	524846388	Tagetes minuta	90.78	206
PTU_406	524846381	Tagetes parryi	87.56	217
PTU_406	524846393	Tagetes multiflora	86.79	212
PTU_406	524846392	Tagetes moorei	86.45	214
PTU_407	KF454079	Convolvulus arvensis	97.94	291
PTU_407	KF454072	Convolvulus arvensis	97.59	291
PTU_407	KF454077	Convolvulus arvensis	97.25	291
PTU_407	586598656	Convolvulus arvensis	96.70	212
PTU_408	MF171466	Ipomoea sp. GO-2017	98.61	288
PTU_408	JQ916065	Ipomoea batatas	98.26	288
PTU_408	514856968	Ipomoea triloba	99.52	209
PTU_408	514856965	Ipomoea triloba	99.52	209
PTU_409	459256895	Echinochloa frumentacea	100.00	205
PTU_409	560592089	Echinochloa colona	100.00	205
PTU_409	459256898	Echinochloa frumentacea	100.00	205

qseqid	sseqid	stitle	pident	length
PTU_409	7271143	Echinochloa crus-galli	99.51	206
PTU_410	KF454118	Origanum vulgare	99.34	302
PTU_410	KX166553	Thymus pulegioides	91.75	303
PTU_410	KF454518	Thymus marschallianus	91.72	302
PTU_410	KC295045	Thymus mongolicus	91.39	302
PTU_411	KX166238	Solanum nigrum	93.92	263
PTU_411	KF454049	Solanum nigrum	93.92	263
PTU_411	KY652843	Solanum nigrum	93.54	263
PTU_411	316986388	Capsicum frutescens	98.14	215
PTU_412	KX166238	Solanum nigrum	92.40	263
PTU_412	KF454049	Solanum nigrum	92.40	263
PTU_412	KY652843	Solanum nigrum	92.02	263
PTU_412	316986388	Capsicum frutescens	95.79	214
PTU_413	524846388	Tagetes minuta	90.78	206
PTU_413	524846381	Tagetes parryi	87.56	217
PTU_413	524846393	Tagetes multiflora	86.79	212
PTU_413	524846392	Tagetes moorei	86.45	214
PTU_414	KX167151	Teucrium scorodonia	91.21	273
PTU_414	KX167889	Teucrium scordium	88.93	271
PTU_414	560592068	Teucrium leucocladum	96.84	190
PTU_414	FM200110	Vitex tripinnata	87.64	259
PTU_415	GU327673	Helianthemum syriacum	90.31	227
PTU_415	KX282189	Helianthemum lippii	90.27	226
PTU_415	KX282184	Helianthemum kahiricum	90.27	226
PTU_415	GU327674	Helianthemum syriacum	89.87	227
PTU_416	KX166238	Solanum nigrum	93.49	261
PTU_416	KF454049	Solanum nigrum	93.49	261

qseqid	sseqid	stitle	pident	length
PTU_416	KY652843	Solanum nigrum	93.10	261
PTU_416	316986388	Capsicum frutescens	96.74	215
PTU_417	KX166238	Solanum nigrum	94.27	262
PTU_417	KF454049	Solanum nigrum	94.27	262
PTU_417	KY652843	Solanum nigrum	93.89	262
PTU_417	KX282258	Lycium shawii	92.11	266
PTU_418	KX282217	Imperata cylindrica	92.96	284
PTU_418	KX282107	Dichanthium annulatum	92.25	284
PTU_418	KF184927	Saccharum hybrid cultivar	91.58	285
PTU_418	KX689356	Chrysopogon argutus	90.53	285
PTU_419	KF454118	Origanum vulgare	99.34	302
PTU_419	KX166553	Thymus pulegioides	91.42	303
PTU_419	KF454518	Thymus marschallianus	91.39	302
PTU_419	KC295045	Thymus mongolicus	91.06	302
PTU_420	KX167814	Mentha pulegium	94.04	302
PTU_420	KC591663	Acanthomintha lanceolata	92.33	300
PTU_420	KC591662	Acanthomintha ilicifolia	91.67	300
PTU_420	KC295040	Ziziphora clinopodioides	91.06	302
PTU_421	KX166238	Solanum nigrum	95.06	263
PTU_421	KF454049	Solanum nigrum	95.06	263
PTU_421	KY652843	Solanum nigrum	94.68	263
PTU_421	KX346971	Solanum lyratum	92.75	262
PTU_422	KX166238	Solanum nigrum	93.54	263
PTU_422	KF454049	Solanum nigrum	93.54	263
PTU_422	KY652843	Solanum nigrum	93.16	263
PTU_422	316986388	Capsicum frutescens	97.21	215
PTU_423	KX166238	Solanum nigrum	91.63	263

qseqid	sseqid	stitle	pident	length
PTU_423	KF454049	Solanum nigrum	91.25	263
PTU_423	KY652843	Solanum nigrum	90.87	263
PTU_423	KX282258	Lycium shawii	90.73	259
PTU_424	KX166238	Solanum nigrum	95.06	263
PTU_424	KF454049	Solanum nigrum	95.06	263
PTU_424	KY652843	Solanum nigrum	94.30	263
PTU_424	KF454093	Lycium ruthenicum	91.94	273
PTU_425	KY652843	Solanum nigrum	92.06	252
PTU_425	KX166238	Solanum nigrum	91.67	252
PTU_425	KF454049	Solanum nigrum	91.67	252
PTU_425	KX282258	Lycium shawii	88.76	267
PTU_426	KX166238	Solanum nigrum	92.40	250
PTU_426	KF454049	Solanum nigrum	92.40	250
PTU_426	KY652843	Solanum nigrum	92.00	250
PTU_426	KX282258	Lycium shawii	88.76	267
PTU_427	KX096059	Ocimum basilicum	96.70	273
PTU_427	KF454238	Ocimum basilicum	93.88	278
PTU_427	KX096056	Ocimum americanum	92.81	278
PTU_427	KR531700	Alangium kurzii	96.94	98
PTU_428	KC129434	Austrostipa verticillata	94.87	273
PTU_428	KX282442	Stipa capensis	94.16	274
PTU_428	KC129444	Nassella megapotamia	93.80	274
PTU_428	KC129453	Nassella megapotamia	92.34	274
PTU_429	KX166238	Solanum nigrum	89.69	262
PTU_429	KF454049	Solanum nigrum	89.69	262
PTU_429	KY652843	Solanum nigrum	88.93	262
PTU_429	KF454093	Lycium ruthenicum	86.92	260

qseqid	sseqid	stitle	pident	length
PTU_430	KX166238	Solanum nigrum	93.51	262
PTU_430	KF454049	Solanum nigrum	93.51	262
PTU_430	KY652843	Solanum nigrum	93.13	262
PTU_430	316986388	Capsicum frutescens	96.74	215
PTU_431	KX166814	Polygonum arenastrum	99.25	265
PTU_431	KF530289	Polygonum aviculare	95.47	265
PTU_431	KX166588	Polygonum aviculare	95.09	265
PTU_431	284811160	Polygonum rurivagum	100.00	182
PTU_432	KX167323	Lactuca saligna	99.66	293
PTU_432	KX166092	Lactuca saligna	99.66	293
PTU_432	KF454303	Iris ruthenica	97.95	293
PTU_432	KM210323	Lactuca sativa	97.61	293
PTU_433	KX166238	Solanum nigrum	93.87	261
PTU_433	KF454049	Solanum nigrum	93.87	261
PTU_433	KY652843	Solanum nigrum	93.10	261
PTU_433	KX346971	Solanum lyratum	92.31	260
PTU_434	KX166238	Solanum nigrum	93.92	263
PTU_434	KF454049	Solanum nigrum	93.92	263
PTU_434	KY652843	Solanum nigrum	93.16	263
PTU_434	KF454093	Lycium ruthenicum	90.66	289
PTU_435	KF454118	Origanum vulgare	99.27	137
PTU_435	KF454118	Origanum vulgare	95.28	127
PTU_435	KX166553	Thymus pulegioides	94.78	134
PTU_435	KX166553	Thymus pulegioides	89.76	127
PTU_435	KF454518	Thymus marschallianus	94.74	133
PTU_435	KF454518	Thymus marschallianus	89.76	127
PTU_435	KC295045	Thymus mongolicus	94.74	133

qseqid	sseqid	stitle	pident	length
PTU_435	KC295045	Thymus mongolicus	88.98	127
PTU_436	KX166238	Solanum nigrum	95.44	263
PTU_436	KF454049	Solanum nigrum	95.44	263
PTU_436	KY652843	Solanum nigrum	94.68	263
PTU_436	KF454093	Lycium ruthenicum	92.31	273
PTU_437	KX166238	Solanum nigrum	93.10	261
PTU_437	KF454049	Solanum nigrum	93.10	261
PTU_437	KY652843	Solanum nigrum	92.72	261
PTU_437	KX346971	Solanum lyratum	90.77	260
PTU_438	KX166238	Solanum nigrum	90.49	263
PTU_438	KF454049	Solanum nigrum	90.49	263
PTU_438	KY652843	Solanum nigrum	90.11	263
PTU_438	316986388	Capsicum frutescens	94.39	214
PTU_439	KF454118	Origanum vulgare	97.34	301
PTU_439	KX166553	Thymus pulegioides	90.40	302
PTU_439	KF454518	Thymus marschallianus	90.37	301
PTU_439	KF454516	Thymus altaicus	90.37	301
PTU_440	KX167079	Oxybasis rubra	98.63	292
PTU_440	KX166129	Oxybasis glauca	97.95	292
PTU_440	KX167081	Oxybasis urbica	96.58	292
PTU_440	KX166888	Oxybasis glauca	94.86	292
PTU_441	KF454238	Ocimum basilicum	90.15	274
PTU_441	KF454054	Solanum kitagawae	94.32	88
PTU_441	378406759	Hyptis heterodon	85.40	137
PTU_441	JF301454	Eplingiella fruticosa	84.67	137
PTU_442	KX281075	Eragrostis curvula	91.84	282
PTU_442	KX281067	Eragrostis curvula	91.49	282

qseqid	sseqid	stitle	pident	length
PTU_442	KX281069	Eragrostis curvula	90.78	282
PTU_442	KX281068	Eragrostis curvula	90.78	282
PTU_443	KY652843	Solanum nigrum	92.19	256
PTU_443	KF454049	Solanum nigrum	92.22	257
PTU_443	KX166238	Solanum nigrum	91.80	256
PTU_443	KX346971	Solanum lyratum	90.62	256
PTU_444	KF454646	Nigella damascena	97.82	275
PTU_444	KF454645	Nigella glandulifera	97.45	275
PTU_444	KF454648	Nigella glandulifera	97.09	275
PTU_444	KF454644	Nigella glandulifera	97.09	275
PTU_445	KX166495	Logfia gallica	97.54	284
PTU_445	KX166280	Gnaphalium supinum	97.54	284
PTU_445	KX282156	Filago pyramidata	97.54	284
PTU_445	KX166911	Logfia minima	97.18	284
PTU_446	KJ131556	Triticum turgidum	99.65	285
PTU_446	KJ131570	Triticum spelta	99.30	285
PTU_446	KJ131560	Triticum turgidum	99.30	285
PTU_446	KJ131569	Triticum turgidum	98.95	285
PTU_447	KX096059	Ocimum basilicum	91.88	271
PTU_447	KX096056	Ocimum americanum	91.58	273
PTU_447	KF454238	Ocimum basilicum	90.88	274
PTU_447	KU203796	Damrongia orientalis	97.80	91
PTU_448	KY652843	Solanum nigrum	93.00	257
PTU_448	KX166238	Solanum nigrum	92.61	257
PTU_448	KF454049	Solanum nigrum	92.61	257
PTU_448	KX282258	Lycium shawii	90.35	259
PTU_449	KX166238	Solanum nigrum	93.92	263

qseqid	sseqid	stitle	pident	length
PTU_449	KF454049	Solanum nigrum	93.92	263
PTU_449	KY652843	Solanum nigrum	93.16	263
PTU_449	KF454093	Lycium ruthenicum	91.58	273
PTU_450	NA	NA	NA	NA
PTU_451	KX166238	Solanum nigrum	92.40	263
PTU_451	KF454049	Solanum nigrum	92.40	263
PTU_451	KY652843	Solanum nigrum	91.63	263
PTU_451	KF454093	Lycium ruthenicum	91.12	259
PTU_452	KX166238	Solanum nigrum	91.63	263
PTU_452	KF454049	Solanum nigrum	91.63	263
PTU_452	KY652843	Solanum nigrum	91.25	263
PTU_452	KU724227	Withania somnifera	89.39	264
PTU_453	KX166238	Solanum nigrum	91.67	264
PTU_453	KY652843	Solanum nigrum	91.29	264
PTU_453	KF454049	Solanum nigrum	91.32	265
PTU_453	316986388	Capsicum frutescens	95.33	214
PTU_454	MF171486	Ipomoea carnea	90.68	279
PTU_454	MF171643	Ipomoea marabensis	89.51	286
PTU_454	MF171463	Ipomoea sp. GO-2017	89.08	284
PTU_454	MF171725	Ipomoea marabensis	90.30	268
PTU_455	KY652843	Solanum nigrum	99.64	274
PTU_455	KX166238	Solanum nigrum	99.63	272
PTU_455	KF454049	Solanum nigrum	98.16	272
PTU_455	KX346971	Solanum lyratum	93.48	276
PTU_456	KX166238	Solanum nigrum	90.11	263
PTU_456	KF454049	Solanum nigrum	90.11	263
PTU_456	KY652843	Solanum nigrum	89.73	263

qseqid	sseqid	stitle	pident	length
PTU_456	316986388	Capsicum frutescens	93.93	214
PTU_457	KF454079	Convolvulus arvensis	98.26	288
PTU_457	KF454072	Convolvulus arvensis	97.92	288
PTU_457	KF454077	Convolvulus arvensis	97.57	288
PTU_457	586598656	Convolvulus arvensis	97.13	209
PTU_458	KX165886	Bromus erectus	98.22	281
PTU_458	KX165557	Bromus sterilis	97.86	281
PTU_458	KX282048	Bromus tectorum	97.51	281
PTU_458	KX282047	Bromus tectorum	97.51	281
PTU_459	KX166238	Solanum nigrum	93.92	263
PTU_459	KF454049	Solanum nigrum	93.92	263
PTU_459	KY652843	Solanum nigrum	93.54	263
PTU_459	KX282258	Lycium shawii	90.03	291
PTU_460	KF454118	Origanum vulgare	96.35	301
PTU_460	KX166553	Thymus pulegioides	90.73	302
PTU_460	KF454518	Thymus marschallianus	90.70	301
PTU_460	KF454516	Thymus altaicus	90.70	301
PTU_461	KX166397	Calystegia soldanella	90.48	294
PTU_461	LC085876	Calystegia hederacea	90.14	294
PTU_461	KX165837	Calystegia sepium	89.80	294
PTU_461	KF454079	Convolvulus arvensis	89.04	292
PTU_462	KF454118	Origanum vulgare	94.70	302
PTU_462	KX166553	Thymus pulegioides	88.82	304
PTU_462	KF454518	Thymus marschallianus	88.78	303
PTU_462	56068995	Origanum vulgare	93.15	219
PTU_463	KX096056	Ocimum americanum	93.43	274
PTU_463	KX096059	Ocimum basilicum	93.41	273

qseqid	sseqid	stitle	pident	length
PTU_463	KF454238	Ocimum basilicum	92.73	275
PTU_463	HF542817	Capurodendron sp. US-2012	97.80	91
PTU_464	316986388	Capsicum frutescens	98.14	215
PTU_464	316986383	Capsicum sp. "Bhut Jolokia"	98.14	215
PTU_464	316986386	Capsicum sp. "Bhut Jolokia"	98.14	215
PTU_464	316986387	Capsicum chinense	98.14	215
PTU_465	KF454049	Solanum nigrum	93.89	262
PTU_465	KX166238	Solanum nigrum	93.49	261
PTU_465	KY652843	Solanum nigrum	93.10	261
PTU_465	KX282258	Lycium shawii	91.92	260
PTU_466	663084878	Amaranthus tuberculatus	98.56	209
PTU_466	663084873	Amaranthus capensis	98.56	209
PTU_466	663084912	Amaranthus tuberculatus	98.56	209
PTU_466	663084944	Amaranthus blitoides	98.56	209
PTU_467	KX282197	Herniaria hirsuta	99.63	267
PTU_467	KX282196	Herniaria hirsuta	99.63	267
PTU_467	KX167479	Herniaria glabra	99.25	268
PTU_467	KX167683	Herniaria ciliolata	98.88	268
PTU_468	KF454238	Ocimum basilicum	95.98	174
PTU_468	KX096056	Ocimum americanum	94.22	173
PTU_468	KX096059	Ocimum basilicum	94.15	171
PTU_468	378406818	Hyptis odorata	90.58	138
PTU_469	KX166238	Solanum nigrum	93.16	263
PTU_469	KF454049	Solanum nigrum	93.16	263
PTU_469	KY652843	Solanum nigrum	92.78	263
PTU_469	316986388	Capsicum frutescens	96.73	214
PTU_470	KF454118	Origanum vulgare	98.34	302

qseqid	sseqid	stitle	pident	length
PTU_470	KX166553	Thymus pulegioides	90.43	303
PTU_470	KF454518	Thymus marschallianus	90.40	302
PTU_470	KC295045	Thymus mongolicus	90.07	302
PTU_471	KX096056	Ocimum americanum	92.42	277
PTU_471	KX096059	Ocimum basilicum	92.03	276
PTU_471	KF454238	Ocimum basilicum	91.37	278
PTU_471	DQ154051	Chrysophyllum roxburghii	96.74	92
PTU_472	KX282324	Phragmites australis	99.29	280
PTU_472	LC085881	Phragmites japonicus	98.21	280
PTU_472	KX167579	Molinia caerulea	96.72	274
PTU_472	HQ329791	Eragrostis walteri	94.44	270
PTU_473	KX166238	Solanum nigrum	89.31	262
PTU_473	KF454049	Solanum nigrum	89.31	262
PTU_473	KY652843	Solanum nigrum	88.55	262
PTU_473	KF454093	Lycium ruthenicum	86.97	261
PTU_474	KX096056	Ocimum americanum	92.36	275
PTU_474	KX096059	Ocimum basilicum	92.34	274
PTU_474	KF454238	Ocimum basilicum	91.67	276
PTU_474	DQ154051	Chrysophyllum roxburghii	97.80	91
PTU_475	GU327675	Helianthemum almeriense	97.67	86
PTU_475	AB743843	Oxalis corniculata	97.67	86
PTU_475	KR532667	Toona ciliata	97.67	86
PTU_475	KC698936	Helianthemum marifolium	97.67	86
PTU_476	KY652843	Solanum nigrum	93.39	257
PTU_476	KX166238	Solanum nigrum	93.00	257
PTU_476	KF454049	Solanum nigrum	93.00	257
PTU_476	KX282258	Lycium shawii	90.73	259

qseqid	sseqid	stitle	pident	length
PTU_477	KX166238	Solanum nigrum	93.16	263
PTU_477	KF454049	Solanum nigrum	93.16	263
PTU_477	KY652843	Solanum nigrum	92.78	263
PTU_477	316986388	Capsicum frutescens	96.73	214
PTU_478	KX166238	Solanum nigrum	94.68	263
PTU_478	KF454049	Solanum nigrum	94.68	263
PTU_478	KY652843	Solanum nigrum	94.30	263
PTU_478	316986388	Capsicum frutescens	98.13	214
PTU_479	KX166238	Solanum nigrum	90.49	263
PTU_479	KF454049	Solanum nigrum	90.49	263
PTU_479	KY652843	Solanum nigrum	90.11	263
PTU_479	316986388	Capsicum frutescens	94.88	215
PTU_480	KX166238	Solanum nigrum	91.57	261
PTU_480	KF454049	Solanum nigrum	91.57	261
PTU_480	316986388	Capsicum frutescens	96.23	212
PTU_480	316986383	Capsicum sp. “Bhut Jolokia”	96.23	212
PTU_481	KF454072	Convolvulus arvensis	97.94	291
PTU_481	KF454079	Convolvulus arvensis	97.59	291
PTU_481	KF454077	Convolvulus arvensis	97.59	291
PTU_481	586598656	Convolvulus arvensis	99.06	212
PTU_482	KT031859	Linaria genistifolia	99.65	285
PTU_482	KX165842	Linaria vulgaris	99.30	285
PTU_482	KT031866	Linaria michauxii	98.95	285
PTU_482	KT031870	Linaria salangensis	98.60	285
PTU_483	KX166238	Solanum nigrum	91.80	256
PTU_483	KY652843	Solanum nigrum	91.41	256
PTU_483	KF454049	Solanum nigrum	90.98	255

qseqid	sseqid	stitle	pident	length
PTU_483	KX282258	Lycium shawii	88.76	258
PTU_484	KX166238	Solanum nigrum	91.63	263
PTU_484	KF454049	Solanum nigrum	91.63	263
PTU_484	KY652843	Solanum nigrum	91.25	263
PTU_484	KU724227	Withania somnifera	89.39	264
PTU_485	KX166238	Solanum nigrum	94.68	263
PTU_485	KF454049	Solanum nigrum	94.68	263
PTU_485	KY652843	Solanum nigrum	94.30	263
PTU_485	KX282258	Lycium shawii	92.51	267
PTU_486	KX166238	Solanum nigrum	91.25	263
PTU_486	KF454049	Solanum nigrum	90.87	263
PTU_486	KY652843	Solanum nigrum	90.49	263
PTU_486	KX282258	Lycium shawii	90.35	259
PTU_487	KX166238	Solanum nigrum	93.89	262
PTU_487	KF454049	Solanum nigrum	93.89	262
PTU_487	KY652843	Solanum nigrum	93.51	262
PTU_487	KX282258	Lycium shawii	92.11	266
PTU_488	KX096056	Ocimum americanum	90.55	275
PTU_488	KX096059	Ocimum basilicum	90.15	274
PTU_488	KF454238	Ocimum basilicum	89.86	276
PTU_488	JF301414	Martianthus leucocephalus	86.86	137
PTU_489	KX096059	Ocimum basilicum	92.80	264
PTU_489	KX096056	Ocimum americanum	92.48	266
PTU_489	KF454238	Ocimum basilicum	91.76	267
PTU_489	JF301414	Martianthus leucocephalus	80.15	267
PTU_490	KX166238	Solanum nigrum	93.00	257
PTU_490	KF454049	Solanum nigrum	93.00	257

qseqid	sseqid	stitle	pident	length
PTU_490	KY652843	Solanum nigrum	92.61	257
PTU_490	KF454093	Lycium ruthenicum	87.85	288
PTU_491	KX166238	Solanum nigrum	93.92	148
PTU_491	KX166238	Solanum nigrum	97.62	84
PTU_491	KF454049	Solanum nigrum	93.92	148
PTU_491	KF454049	Solanum nigrum	97.62	84
PTU_491	KY652843	Solanum nigrum	93.24	148
PTU_491	KY652843	Solanum nigrum	96.43	84
PTU_491	KF454093	Lycium ruthenicum	93.10	145
PTU_491	KF454093	Lycium ruthenicum	92.92	113
PTU_492	KX166238	Solanum nigrum	91.63	263
PTU_492	KF454049	Solanum nigrum	91.63	263
PTU_492	KY652843	Solanum nigrum	91.25	263
PTU_492	KF454093	Lycium ruthenicum	90.73	259
PTU_493	26190454	Nepeta cataria	85.37	123
PTU_493	26190448	Nepeta crassifolia	85.12	121
PTU_493	67078901	Justicia americana	84.44	90
PTU_493	68235657	Aphelandra castanifolia	84.21	95
PTU_494	KX166238	Solanum nigrum	92.37	262
PTU_494	KF454049	Solanum nigrum	92.37	262
PTU_494	KY652843	Solanum nigrum	91.98	262
PTU_494	KX282258	Lycium shawii	90.38	260
PTU_495	KX166238	Solanum nigrum	94.27	262
PTU_495	KF454049	Solanum nigrum	94.27	262
PTU_495	KY652843	Solanum nigrum	93.89	262
PTU_495	KX282258	Lycium shawii	92.11	266
PTU_496	X86848	Silene dichotoma	96.88	288

qseqid	sseqid	stitle	pident	length
PTU_496	KY624401	Vaccaria hispanica	97.19	285
PTU_496	KX167123	Silene latifolia	96.18	288
PTU_496	KX166380	Silene vulgaris	97.45	274
PTU_497	KX166238	Solanum nigrum	92.80	250
PTU_497	KF454049	Solanum nigrum	92.80	250
PTU_497	KY652843	Solanum nigrum	92.40	250
PTU_497	KX282258	Lycium shawii	89.14	267
PTU_498	KX166238	Solanum nigrum	93.92	263
PTU_498	KF454049	Solanum nigrum	93.92	263
PTU_498	KY652843	Solanum nigrum	93.54	263
PTU_498	KF454093	Lycium ruthenicum	92.66	259
PTU_499	KX165483	Petrorhagia nanteuilii	97.47	277
PTU_499	KX165484	Petrorhagia nanteuilii	97.11	277
PTU_499	KX166595	Dianthus deltoides	92.14	280
PTU_499	JF421492	Dianthus chinensis	92.09	278
PTU_500	KX096059	Ocimum basilicum	91.54	272
PTU_500	KX096056	Ocimum americanum	90.51	274
PTU_500	KF454238	Ocimum basilicum	90.55	275
PTU_500	KU203796	Damrongia orientalis	95.60	91
PTU_501	KF241280	Arnebia euchroma	97.59	291
PTU_501	KX166735	Tragopogon pratensis	96.91	291
PTU_501	317451650	Tragopogon porrifolius	98.10	210
PTU_501	317451649	Tragopogon porrifolius	98.10	210
PTU_502	KX096056	Ocimum americanum	90.23	174
PTU_502	KF454238	Ocimum basilicum	91.02	167
PTU_502	KX096059	Ocimum basilicum	90.96	166
PTU_502	378406718	Hyptis argyrophylla	96.00	100

qseqid	sseqid	stitle	pident	length
PTU_503	KY652843	Solanum nigrum	90.60	266
PTU_503	KX166238	Solanum nigrum	90.23	266
PTU_503	KF454049	Solanum nigrum	90.23	266
PTU_503	KF454093	Lycium ruthenicum	89.13	276
PTU_504	KX166238	Solanum nigrum	94.68	263
PTU_504	KF454049	Solanum nigrum	94.68	263
PTU_504	KY652843	Solanum nigrum	93.92	263
PTU_504	KF454093	Lycium ruthenicum	93.05	259
PTU_505	KF241285	Cichorium glandulosum	94.18	292
PTU_505	KX166690	Arnoseris minima	92.81	292
PTU_505	KX167641	Arnoseris minima	92.81	292
PTU_505	AY879164	Hieracium rubrum	90.41	292
PTU_506	295152696	Diplachne fusca subsp. uninervia	99.49	198
PTU_506	139539096	Diplachne fusca subsp. uninervia	98.99	199
PTU_506	109693380	Diplachne fusca	99.48	193
PTU_506	409690869	Diplachne fusca subsp. muelleri	99.47	189
PTU_507	KX166397	Calystegia soldanella	86.32	285
PTU_507	LC085876	Calystegia hederacea	85.96	285
PTU_507	KX165837	Calystegia sepium	85.61	285
PTU_507	KX282079	Convolvulus cephalopodus	85.51	283
PTU_508	KF454118	Origanum vulgare	99.34	302
PTU_508	KX166553	Thymus pulegioides	91.42	303
PTU_508	KF454518	Thymus marschallianus	91.39	302
PTU_508	KC295045	Thymus mongolicus	91.06	302
PTU_509	KX282181	Haloxylon salicornicum	91.97	274
PTU_509	KX282180	Haloxylon salicornicum	91.97	274
PTU_509	KX282182	Haloxylon salicornicum	91.61	274

qseqid	sseqid	stitle	pident	length
PTU_509	KX282091	Cornulaca monacantha	90.88	274
PTU_510	KX166631	Erodium moschatum	99.67	301
PTU_510	KX166633	Erodium cicutarium	99.00	301
PTU_510	KX166760	Erodium cicutarium	98.67	301
PTU_510	KX166632	Erodium lebelii	98.34	301
PTU_511	KX166910	Crepis paludosa	94.20	276
PTU_511	KX166909	Crepis paludosa	93.84	276
PTU_511	KX166494	Crepis praemorsa	92.47	292
PTU_511	KX165611	Lapsana communis	92.10	291
PTU_512	KF454475	Carum buriaticum	90.41	292
PTU_512	KX165508	Sison segetum	90.17	295
PTU_512	KX167096	Sison amomum	90.10	293
PTU_512	KM210314	Trachyspermum ammi	90.00	290
PTU_513	317451640	Tragopogon dubius	96.97	132
PTU_513	317451640	Tragopogon dubius	100.00	47
PTU_513	317451639	Tragopogon dubius	96.97	132
PTU_513	317451639	Tragopogon dubius	100.00	47
PTU_513	317451638	Tragopogon dubius	96.97	132
PTU_513	317451638	Tragopogon dubius	100.00	47
PTU_513	317451651	Tragopogon angustifolius	96.97	132
PTU_513	317451651	Tragopogon angustifolius	100.00	47
PTU_514	KX166238	Solanum nigrum	94.68	263
PTU_514	KF454049	Solanum nigrum	94.68	263
PTU_514	KY652843	Solanum nigrum	93.92	263
PTU_514	KF454093	Lycium ruthenicum	93.44	259
PTU_515	KX166238	Solanum nigrum	93.92	263
PTU_515	KF454049	Solanum nigrum	93.92	263

qseqid	sseqid	stitle	pident	length
PTU_515	KY652843	Solanum nigrum	93.16	263
PTU_515	KF454093	Lycium ruthenicum	91.58	273
PTU_516	KX282323	Cenchrus ramosissimus	91.79	280
PTU_516	536720085	Setaria italica	100.00	206
PTU_516	536720075	Setaria italica	100.00	206
PTU_516	536720090	Setaria italica	100.00	206
PTU_517	KY624383	Phlomoides rotata	91.70	289
PTU_517	KX165827	Lamium album	92.03	276
PTU_517	KF861974	Lamium album	92.03	276
PTU_517	KX166736	Stachys sylvatica	90.55	275
PTU_518	KX166238	Solanum nigrum	91.22	262
PTU_518	KF454049	Solanum nigrum	91.22	262
PTU_518	KY652843	Solanum nigrum	90.84	262
PTU_518	316986388	Capsicum frutescens	94.88	215
PTU_519	KX096056	Ocimum americanum	91.67	276
PTU_519	KX096059	Ocimum basilicum	91.27	275
PTU_519	KF454238	Ocimum basilicum	90.97	277
PTU_519	378406718	Hyptis argyrophylla	87.59	137
PTU_520	KX166238	Solanum nigrum	92.40	263
PTU_520	KF454049	Solanum nigrum	92.40	263
PTU_520	KY652843	Solanum nigrum	92.02	263
PTU_520	KX282258	Lycium shawii	90.42	261
PTU_521	KX166238	Solanum nigrum	94.23	260
PTU_521	KF454049	Solanum nigrum	94.23	260
PTU_521	KY652843	Solanum nigrum	93.85	260
PTU_521	KX282258	Lycium shawii	92.61	257
PTU_522	KX096059	Ocimum basilicum	91.58	273

qseqid	sseqid	stitle	pident	length
PTU_522	KF454238	Ocimum basilicum	89.61	279
PTU_522	KX096056	Ocimum americanum	89.21	278
PTU_522	378406723	Hyptis lythroides	88.80	125
PTU_523	KX096059	Ocimum basilicum	94.83	271
PTU_523	KF454238	Ocimum basilicum	93.86	277
PTU_523	KX096056	Ocimum americanum	93.12	276
PTU_523	JF301414	Martianthus leucocephalus	81.99	272
PTU_524	KY968830	Bidens alba	98.28	290
PTU_524	KY968833	Bidens pilosa	99.26	269
PTU_524	18028503	Bidens cronquistii	99.51	206
PTU_524	KX165434	Bidens cernua	92.13	267
PTU_525	KX282181	Haloxylon salicornicum	92.34	274
PTU_525	KX282180	Haloxylon salicornicum	92.31	273
PTU_525	KX282182	Haloxylon salicornicum	91.94	273
PTU_525	KX282091	Cornulaca monacantha	91.24	274
PTU_526	KC129434	Austrostipa verticillata	94.87	273
PTU_526	KX282442	Stipa capensis	94.16	274
PTU_526	KC129444	Nassella megapotamia	93.80	274
PTU_526	KC129453	Nassella megapotamia	92.34	274
PTU_527	KF454118	Origanum vulgare	99.01	302
PTU_527	KX166553	Thymus pulegioides	91.75	303
PTU_527	KF454518	Thymus marschallianus	91.72	302
PTU_527	KC295045	Thymus mongolicus	91.39	302
PTU_528	KX096059	Ocimum basilicum	90.37	270
PTU_528	KX096056	Ocimum americanum	87.96	274
PTU_528	KF454238	Ocimum basilicum	87.27	275
PTU_528	KU203783	Dorcoceras hygrometricum	95.45	88

qseqid	sseqid	stitle	pident	length
PTU_529	KX166238	Solanum nigrum	93.89	262
PTU_529	KF454049	Solanum nigrum	93.89	262
PTU_529	KY652843	Solanum nigrum	93.51	262
PTU_529	KF454093	Lycium ruthenicum	92.64	258
PTU_530	KF454118	Origanum vulgare	92.38	302
PTU_530	KX166553	Thymus pulegioides	86.47	303
PTU_530	KF454518	Thymus marschallianus	86.47	303
PTU_530	KC295045	Thymus mongolicus	86.14	303
PTU_531	KX166238	Solanum nigrum	92.80	250
PTU_531	KF454049	Solanum nigrum	92.80	250
PTU_531	KY652843	Solanum nigrum	92.40	250
PTU_531	KX282258	Lycium shawii	89.51	267
PTU_532	KX166238	Solanum nigrum	93.54	263
PTU_532	KF454049	Solanum nigrum	93.54	263
PTU_532	KY652843	Solanum nigrum	93.16	263
PTU_532	KX282258	Lycium shawii	91.51	259
PTU_533	KX166532	Phleum pratense	97.86	281
PTU_533	KX167314	Phleum phleoides	97.51	281
PTU_533	KX166691	Phleum alpinum	96.80	281
PTU_533	EU792358	Nicoraepoa subenervis	96.43	280
PTU_534	KX166238	Solanum nigrum	88.97	263
PTU_534	KF454049	Solanum nigrum	88.97	263
PTU_534	KY652843	Solanum nigrum	88.59	263
PTU_534	316986388	Capsicum frutescens	92.56	215
PTU_535	LT593966	Brachiaria fragrans	91.48	270
PTU_535	LT593967	Chasechloa egregia	90.33	269
PTU_535	LT593967	Chasechloa egregia	90.33	269

qseqid	sseqid	stitle	pident	length
PTU_535	KX689291	Digitaria horizontalis	90.33	269
PTU_536	KX166238	Solanum nigrum	90.87	263
PTU_536	KF454049	Solanum nigrum	90.87	263
PTU_536	KY652843	Solanum nigrum	90.49	263
PTU_536	316986388	Capsicum frutescens	94.86	214
PTU_537	KX166238	Solanum nigrum	93.92	263
PTU_537	KF454049	Solanum nigrum	93.92	263
PTU_537	KY652843	Solanum nigrum	93.16	263
PTU_537	KX346971	Solanum lyratum	92.37	262
PTU_538	KX165883	Cirsium arvense	100.00	288
PTU_538	KX167061	Cirsium tuberosum	97.55	286
PTU_538	KX167382	Cirsium acaule	97.20	286
PTU_538	KX167205	Cirsium dissectum	97.20	286
PTU_539	KY652843	Solanum nigrum	92.40	263
PTU_539	KX166238	Solanum nigrum	92.40	263
PTU_539	KF454049	Solanum nigrum	92.40	263
PTU_539	KX282258	Lycium shawii	91.39	267
PTU_540	KF241280	Arnebia euchroma	98.28	291
PTU_540	KX166735	Tragopogon pratensis	97.59	291
PTU_540	KF454418	Scorzonera pseudodivaricata	91.04	279
PTU_540	317451650	Tragopogon porrifolius	98.57	210
PTU_541	KF454049	Solanum nigrum	92.02	263
PTU_541	KX166238	Solanum nigrum	91.60	262
PTU_541	KY652843	Solanum nigrum	91.22	262
PTU_541	KU724227	Withania somnifera	90.11	263
PTU_542	KX166238	Solanum nigrum	92.34	261
PTU_542	KF454049	Solanum nigrum	92.34	261

qseqid	sseqid	stitle	pident	length
PTU_542	KY652843	Solanum nigrum	91.57	261
PTU_542	316986388	Capsicum frutescens	96.28	215
PTU_543	KX165768	Arrhenatherum elatius	100.00	281
PTU_543	KX167057	Calamagrostis epigeios	94.68	282
PTU_543	KX167325	Agrostis vinealis	93.62	282
PTU_543	KX165555	Ammophila arenaria	93.62	282
PTU_544	KY968830	Bidens alba	94.14	290
PTU_544	KY968833	Bidens pilosa	95.91	269
PTU_544	5771500	Bidens aurea	99.02	205
PTU_544	2329931	Bidens andicola	99.50	201
PTU_545	KX166238	Solanum nigrum	93.54	263
PTU_545	KF454049	Solanum nigrum	93.54	263
PTU_545	KY652843	Solanum nigrum	93.16	263
PTU_545	KX282258	Lycium shawii	91.76	267
PTU_546	KX166238	Solanum nigrum	91.63	263
PTU_546	KF454049	Solanum nigrum	91.63	263
PTU_546	KY652843	Solanum nigrum	91.25	263
PTU_546	KX282258	Lycium shawii	90.66	257
PTU_547	NA	NA	NA	NA
PTU_548	KT948617	Chenopodium album	98.63	293
PTU_548	KX166014	Chenopodium ficifolium	94.58	295
PTU_548	311235766	Chenopodium album	100.00	220
PTU_548	KX165819	Halimione portulacoides	92.61	284
PTU_549	KX096056	Ocimum americanum	92.34	274
PTU_549	KF454238	Ocimum basilicum	92.36	275
PTU_549	KX096059	Ocimum basilicum	91.94	273
PTU_549	JF301414	Martianthus leucocephalus	80.22	273

qseqid	sseqid	stitle	pident	length
PTU_550	KX166238	Solanum nigrum	94.68	263
PTU_550	KF454049	Solanum nigrum	94.68	263
PTU_550	KY652843	Solanum nigrum	93.92	263
PTU_550	KF454093	Lycium ruthenicum	92.31	273
PTU_551	KX166238	Solanum nigrum	91.98	262
PTU_551	KF454049	Solanum nigrum	91.98	262
PTU_551	KY652843	Solanum nigrum	91.60	262
PTU_551	316986388	Capsicum frutescens	95.35	215
PTU_552	KT031859	Linaria genistifolia	98.95	285
PTU_552	KX165842	Linaria vulgaris	98.60	285
PTU_552	KT031866	Linaria michauxii	98.25	285
PTU_552	KT031870	Linaria salangensis	97.89	285
PTU_553	KM210331	Lavandula angustifolia	87.19	281
PTU_553	KT220701	Perilla setoyensis	84.53	278
PTU_553	KX166755	Salvia pratensis	84.12	277
PTU_553	KT220699	Perilla citriodora	83.99	281
PTU_554	KX096059	Ocimum basilicum	91.61	274
PTU_554	KX096056	Ocimum americanum	90.97	277
PTU_554	KF454238	Ocimum basilicum	89.93	278
PTU_554	380719853	Plectranthus caninus	87.69	130
PTU_555	KX166238	Solanum nigrum	92.34	261
PTU_555	KF454049	Solanum nigrum	92.34	261
PTU_555	KY652843	Solanum nigrum	91.95	261
PTU_555	KX656898	Lycium barbarum	90.31	258
PTU_556	KX166238	Solanum nigrum	94.68	263
PTU_556	KF454049	Solanum nigrum	94.68	263
PTU_556	KY652843	Solanum nigrum	93.92	263

qseqid	sseqid	stitle	pident	length
PTU_556	KF454093	Lycium ruthenicum	91.58	273
PTU_557	KX166238	Solanum nigrum	95.06	263
PTU_557	KF454049	Solanum nigrum	95.06	263
PTU_557	KY652843	Solanum nigrum	94.68	263
PTU_557	KX346971	Solanum lyratum	92.75	262
PTU_558	KX165891	Sanguisorba officinalis	94.14	273
PTU_558	JF421542	Sanguisorba officinalis	93.77	273
PTU_558	JF421541	Sanguisorba officinalis	93.77	273
PTU_558	KX166425	Potentilla erecta	91.27	275
PTU_559	KF241280	Arnebia euchroma	98.02	252
PTU_559	KX166735	Tragopogon pratensis	97.62	252
PTU_559	KX282414	Scorzonera tortuosissima	89.41	255
PTU_559	KF454418	Scorzonera pseudodivaricata	89.33	253
PTU_560	KY652843	Solanum nigrum	93.00	257
PTU_560	KX166238	Solanum nigrum	92.61	257
PTU_560	KF454049	Solanum nigrum	92.61	257
PTU_560	KF454093	Lycium ruthenicum	89.38	273
PTU_561	KX096056	Ocimum americanum	92.75	276
PTU_561	KF454238	Ocimum basilicum	91.73	278
PTU_561	KX096059	Ocimum basilicum	91.30	276
PTU_561	GU726292	Anisomeles indica	97.80	91
PTU_562	KF454238	Ocimum basilicum	90.91	275
PTU_562	KX096059	Ocimum basilicum	90.15	274
PTU_562	KX096056	Ocimum americanum	89.86	276
PTU_562	KU203783	Dorcoceras hygrometricum	98.89	90
PTU_563	153865420	Heliotropium curassavicum	99.51	205
PTU_563	KC878557	Heliotropium indicum	90.48	273

qseqid	sseqid	stitle	pident	length
PTU_563	KX282193	Heliotropium bacciferum	83.68	288
PTU_563	10440949	Tournefortia acutiflora	91.26	206
PTU_564	KX166238	Solanum nigrum	93.54	263
PTU_564	KF454049	Solanum nigrum	93.54	263
PTU_564	KY652843	Solanum nigrum	92.78	263
PTU_564	KX346971	Solanum lyratum	92.34	261
PTU_565	KF454118	Origanum vulgare	96.35	301
PTU_565	KX166553	Thymus pulegioides	91.09	303
PTU_565	KF454518	Thymus marschallianus	91.06	302
PTU_565	KF454516	Thymus altaicus	91.09	303
PTU_566	KX096056	Ocimum americanum	92.75	276
PTU_566	KX096059	Ocimum basilicum	92.36	275
PTU_566	KF454238	Ocimum basilicum	91.70	277
PTU_566	JF301483	Hyptis pulegioides	79.12	273
PTU_567	KF454238	Ocimum basilicum	96.01	276
PTU_567	KX096056	Ocimum americanum	95.64	275
PTU_567	KX096059	Ocimum basilicum	94.53	274
PTU_567	GU726292	Anisomeles indica	79.26	270
PTU_568	LT593966	Brachiaria fragrans	89.47	285
PTU_568	LT593967	Chasechloa egregia	88.85	269
PTU_568	LT593967	Chasechloa egregia	88.85	269
PTU_568	KX689291	Digitaria horizontalis	89.22	269
PTU_569	KY652843	Solanum nigrum	90.80	261
PTU_569	KX166238	Solanum nigrum	90.42	261
PTU_569	KF454049	Solanum nigrum	90.42	261
PTU_569	KX282258	Lycium shawii	89.53	258
PTU_570	KX282041	Brachypodium distachyon	98.61	288

qseqid	sseqid	stitle	pident	length
PTU_570	KX282040	Brachypodium distachyon	98.61	288
PTU_570	KX167503	Brachypodium pinnatum	96.45	282
PTU_570	3282429	Brachypodium rupestre	96.48	199
PTU_571	KX166238	Solanum nigrum	94.68	263
PTU_571	KF454049	Solanum nigrum	94.68	263
PTU_571	KY652843	Solanum nigrum	93.92	263
PTU_571	KF454093	Lycium ruthenicum	93.05	259
PTU_572	KY652843	Solanum nigrum	92.58	256
PTU_572	KF454049	Solanum nigrum	92.61	257
PTU_572	KX166238	Solanum nigrum	92.19	256
PTU_572	KX346971	Solanum lyratum	91.02	256
PTU_573	KX166238	Solanum nigrum	94.30	263
PTU_573	KF454049	Solanum nigrum	94.30	263
PTU_573	KY652843	Solanum nigrum	93.54	263
PTU_573	KF454093	Lycium ruthenicum	91.58	273
PTU_574	KX166238	Solanum nigrum	90.59	255
PTU_574	KF454049	Solanum nigrum	90.59	255
PTU_574	KY652843	Solanum nigrum	90.20	255
PTU_574	KX282258	Lycium shawii	88.54	253
PTU_575	KX167641	Arnoseris minima	91.85	270
PTU_575	KX166690	Arnoseris minima	91.85	270
PTU_575	KF241285	Cichorium glandulosum	89.69	291
PTU_575	KX166501	Hieracium sp. CF-2016	90.30	268
PTU_576	KX096059	Ocimum basilicum	88.64	273
PTU_576	KF454238	Ocimum basilicum	87.36	277
PTU_576	KX096056	Ocimum americanum	86.28	277
PTU_576	JF301483	Hyptis pulegioides	78.75	273

qseqid	sseqid	stitle	pident	length
PTU_577	KX096056	Ocimum americanum	86.13	274
PTU_577	KF454238	Ocimum basilicum	87.20	250
PTU_577	KX534371	Ocimum basilicum	77.70	269
PTU_577	JF301414	Martianthus leucocephalus	84.06	138
PTU_578	KF454238	Ocimum basilicum	98.96	193
PTU_578	KX096056	Ocimum americanum	95.88	194
PTU_578	KX096059	Ocimum basilicum	93.26	193
PTU_578	KY858250	Alsobia dianthiflora	96.97	99
PTU_579	KY652843	Solanum nigrum	91.67	252
PTU_579	KX166238	Solanum nigrum	91.27	252
PTU_579	KF454049	Solanum nigrum	91.27	252
PTU_579	KX282258	Lycium shawii	88.39	267
PTU_580	KX166238	Solanum nigrum	92.61	257
PTU_580	KF454049	Solanum nigrum	92.61	257
PTU_580	KY652843	Solanum nigrum	92.22	257
PTU_580	316986388	Capsicum frutescens	95.81	215
PTU_581	KJ131557	Secale cereale	99.65	284
PTU_581	JF489233	Secale cereale	98.94	284
PTU_581	KX165429	Elymus repens	96.10	282
PTU_581	KX166524	Elymus athericus	95.74	282
PTU_582	KX689335	Sesuvium ayresii	93.33	270
PTU_582	82697158	Trianthema portulacastrum	97.95	195
PTU_582	83715840	Trianthema turgidifolia	93.40	197
PTU_582	82697161	Trianthema ufoensis	91.88	197
PTU_583	KX166238	Solanum nigrum	90.87	263
PTU_583	KF454049	Solanum nigrum	90.87	263
PTU_583	KY652843	Solanum nigrum	90.49	263

qseqid	sseqid	stitle	pident	length
PTU_583	316986388	Capsicum frutescens	94.42	215
PTU_584	316986388	Capsicum frutescens	94.42	215
PTU_584	316986383	Capsicum sp. “Bhut Jolokia”	94.42	215
PTU_584	316986386	Capsicum sp. “Bhut Jolokia”	94.42	215
PTU_584	316986387	Capsicum chinense	94.42	215
PTU_585	KU724217	Plantago lanceolata	98.16	272
PTU_585	KF454409	Plantago lanceolata	97.79	272
PTU_585	KX282338	Plantago lanceolata	94.42	269
PTU_585	KF454388	Plantago minuta	96.48	256
PTU_586	KY968831	Cenchrus purpureus	92.25	284
PTU_586	KX282065	Cenchrus ciliaris	88.58	289
PTU_586	KY968838	Melinis repens	88.50	287
PTU_586	KX282323	Cenchrus ramosissimus	88.77	276
PTU_587	KF454049	Solanum nigrum	93.16	263
PTU_587	KX166238	Solanum nigrum	92.40	263
PTU_587	KY652843	Solanum nigrum	92.02	263
PTU_587	KX282258	Lycium shawii	90.35	259
PTU_588	KX166238	Solanum nigrum	92.02	263
PTU_588	KF454049	Solanum nigrum	92.02	263
PTU_588	KY652843	Solanum nigrum	91.63	263
PTU_588	316986388	Capsicum frutescens	96.23	212
PTU_589	KX166238	Solanum nigrum	93.92	263
PTU_589	KF454049	Solanum nigrum	93.92	263
PTU_589	KY652843	Solanum nigrum	93.16	263
PTU_589	KX346971	Solanum lyratum	92.37	262
PTU_590	KX165815	Phalaris arundinacea	94.33	282
PTU_590	KX165555	Ammophila arenaria	92.93	283

qseqid	sseqid	stitle	pident	length
PTU_590	KX167939	Calamagrostis purpurea	92.58	283
PTU_590	KX167952	Calamagrostis purpurea	92.58	283
PTU_591	KX282324	Phragmites australis	99.29	281
PTU_591	LC085881	Phragmites japonicus	98.22	281
PTU_591	KX167579	Molinia caerulea	95.99	274
PTU_591	HQ329791	Eragrostis walteri	94.44	270
PTU_592	KX096056	Ocimum americanum	92.75	276
PTU_592	KX096059	Ocimum basilicum	92.36	275
PTU_592	KF454238	Ocimum basilicum	91.70	277
PTU_592	JF301483	Hyptis pulegioides	78.75	273
PTU_593	KU350154	Arbutus canariensis	98.66	298
PTU_593	KX165505	Arctous alpina	89.30	299
PTU_593	KU350164	Comarostaphylis diversifolia	89.26	298
PTU_593	KF419121	Comarostaphylis arbutoides	89.35	291
PTU_594	KF454118	Origanum vulgare	96.01	301
PTU_594	KX166553	Thymus pulegioides	90.40	302
PTU_594	KF454518	Thymus marschallianus	90.37	301
PTU_594	KF454516	Thymus altaicus	90.37	301
PTU_595	KF454079	Convolvulus arvensis	97.59	291
PTU_595	KF454072	Convolvulus arvensis	97.25	291
PTU_595	KF454077	Convolvulus arvensis	96.91	291
PTU_595	586598656	Convolvulus arvensis	96.23	212
PTU_596	KY652843	Solanum nigrum	99.64	274
PTU_596	KX166238	Solanum nigrum	99.63	272
PTU_596	KF454049	Solanum nigrum	98.16	272
PTU_596	KX346971	Solanum lyratum	93.48	276
PTU_597	524846388	Tagetes minuta	91.26	206

qseqid	sseqid	stitle	pident	length
PTU_597	524846381	Tagetes parryi	88.02	217
PTU_597	524846392	Tagetes moorei	86.92	214
PTU_597	524846393	Tagetes multiflora	86.79	212
PTU_598	KF454087	Hordeum vulgare	94.04	285
PTU_598	KJ606357	Hordeum vulgare	93.33	285
PTU_598	KX166524	Elymus athericus	92.63	285
PTU_598	KX282206	Hordeum murinum	92.58	283
PTU_599	KF241280	Arnebia euchroma	97.94	291
PTU_599	KX166735	Tragopogon pratensis	97.25	291
PTU_599	317451650	Tragopogon porrifolius	98.10	210
PTU_599	317451649	Tragopogon porrifolius	98.10	210
PTU_600	KX282189	Helianthemum lippii	91.29	264
PTU_600	KX282184	Helianthemum kahiricum	91.29	264
PTU_600	GU327673	Helianthemum syriacum	90.91	264
PTU_600	KC698933	Helianthemum hirtum	90.87	263
PTU_601	KX096056	Ocimum americanum	92.36	275
PTU_601	KX096059	Ocimum basilicum	91.61	274
PTU_601	KF454238	Ocimum basilicum	91.30	276
PTU_601	KY858250	Alsobia dianthiflora	77.43	257
PTU_602	KX096059	Ocimum basilicum	89.80	255
PTU_602	KF454238	Ocimum basilicum	88.51	261
PTU_602	KX096056	Ocimum americanum	88.46	260
PTU_602	JF301483	Hyptis pulegioides	96.84	95
PTU_603	KX096059	Ocimum basilicum	92.65	272
PTU_603	KX096056	Ocimum americanum	91.64	275
PTU_603	KF454238	Ocimum basilicum	91.30	276
PTU_603	378406718	Hyptis argyrophylla	86.16	159

qseqid	sseqid	stitle	pident	length
PTU_604	480312834	Salvia x sylvestris	99.55	221
PTU_604	KX166755	Salvia pratensis	92.53	281
PTU_604	480312807	Salvia officinalis	99.10	221
PTU_604	359456544	Salvia officinalis	98.63	219
PTU_605	JF831213	Muehlenbeckia gracillima	91.54	260
PTU_605	JF831210	K.L.Wilson & Makinson	90.73	259
PTU_605	JF831211	Muehlenbeckia diclina	89.27	261
PTU_605	JF831221	K.L.Wilson & Makinson	88.59	263
PTU_606	316986388	Capsicum frutescens	96.76	216
PTU_606	316986383	Capsicum sp. “Bhut Jolokia”	96.76	216
PTU_606	316986386	Capsicum sp. “Bhut Jolokia”	96.76	216
PTU_606	316986387	Capsicum chinense	96.76	216
PTU_607	KX166238	Solanum nigrum	93.90	246
PTU_607	KY652843	Solanum nigrum	93.50	246
PTU_607	KF454049	Solanum nigrum	92.65	245
PTU_607	316986388	Capsicum frutescens	95.81	215
PTU_608	KX166238	Solanum nigrum	94.66	262
PTU_608	KF454049	Solanum nigrum	94.66	262
PTU_608	KY652843	Solanum nigrum	94.27	262
PTU_608	KX282258	Lycium shawii	92.48	266
PTU_609	KX166238	Solanum nigrum	92.25	258
PTU_609	KY652843	Solanum nigrum	91.86	258
PTU_609	KF454049	Solanum nigrum	91.83	257
PTU_609	KX282258	Lycium shawii	89.58	259
PTU_610	KX165891	Sanguisorba officinalis	94.10	271
PTU_610	JF421542	Sanguisorba officinalis	93.73	271
PTU_610	JF421541	Sanguisorba officinalis	93.73	271

qseqid	sseqid	stitle	pident	length
PTU_610	KX166425	Potentilla erecta	91.21	273
PTU_611	KF454238	Ocimum basilicum	95.29	276
PTU_611	KX096056	Ocimum americanum	94.93	276
PTU_611	KX096059	Ocimum basilicum	94.16	274
PTU_611	JF301483	Hyptis pulegioides	80.95	273
PTU_612	KX166019	Chenopodiastrum murale	100.00	281
PTU_612	KX166125	Chenopodiastrum hybridum	90.85	295
PTU_612	KX167079	Oxybasis rubra	90.10	293
PTU_612	KX167081	Oxybasis urbica	89.38	292
PTU_613	KX282104	Cynodon dactylon	96.07	280
PTU_613	KP205451	Cynodon dactylon	95.71	280
PTU_613	3850293	Cynodon dactylon	94.92	197
PTU_613	3850289	Cynodon dactylon	94.42	197
PTU_614	KU350154	Arbutus canariensis	95.35	301
PTU_614	KX165505	Arctous alpina	87.46	303
PTU_614	KU350164	Comarostaphylis diversifolia	87.04	301
PTU_614	KF419121	Comarostaphylis arbutoides	87.41	294
PTU_615	KF241280	Arnebia euchroma	98.63	291
PTU_615	KX166735	Tragopogon pratensis	97.94	291
PTU_615	317451650	Tragopogon porrifolius	99.52	210
PTU_615	317451649	Tragopogon porrifolius	99.52	210
PTU_616	KX167455	Geranium rotundifolium	99.33	299
PTU_616	KX166784	Geranium pratense	93.98	299
PTU_616	KY624391	Geranium wilfordii	93.65	299
PTU_616	KX166786	Geranium columbinum	93.33	300
PTU_617	KJ131558	Triticum monococcum	99.65	284
PTU_617	KX165429	Elymus repens	98.23	282

qseqid	sseqid	stitle	pident	length
PTU_617	KX166703	Elymus caninus	97.88	283
PTU_617	KX166702	Elymus caninus	97.88	283
PTU_618	KF241280	Arnebia euchroma	97.59	291
PTU_618	KX166735	Tragopogon pratensis	96.91	291
PTU_618	317451650	Tragopogon porrifolius	98.57	210
PTU_618	317451649	Tragopogon porrifolius	98.57	210
PTU_619	KX166238	Solanum nigrum	89.27	261
PTU_619	KF454049	Solanum nigrum	89.27	261
PTU_619	KY652843	Solanum nigrum	88.89	261
PTU_619	KX282258	Lycium shawii	87.98	258
PTU_620	KX096056	Ocimum americanum	95.64	275
PTU_620	KF454238	Ocimum basilicum	95.29	276
PTU_620	KX096059	Ocimum basilicum	94.53	274
PTU_620	KX534371	Ocimum basilicum	83.56	292
PTU_621	KX096056	Ocimum americanum	95.29	276
PTU_621	KF454238	Ocimum basilicum	94.95	277
PTU_621	KX096059	Ocimum basilicum	94.18	275
PTU_621	KX534371	Ocimum basilicum	83.04	289
PTU_622	KJ131558	Triticum monococcum	100.00	284
PTU_622	KX165429	Elymus repens	98.58	282
PTU_622	KX166703	Elymus caninus	98.23	283
PTU_622	KX166702	Elymus caninus	98.23	283
PTU_623	KX096056	Ocimum americanum	92.36	275
PTU_623	KX096059	Ocimum basilicum	91.97	274
PTU_623	KF454238	Ocimum basilicum	91.67	276
PTU_623	378406720	Hyptis glomerata	87.68	138
PTU_624	KX282217	Imperata cylindrica	93.31	284

qseqid	sseqid	stitle	pident	length
PTU_624	KX282107	Dichanthium annulatum	92.61	284
PTU_624	KF184927	Saccharum hybrid cultivar	91.93	285
PTU_624	KX689356	Chrysopogon argutus	90.88	285
PTU_625	KF454118	Origanum vulgare	96.01	301
PTU_625	KX166553	Thymus pulegioides	89.07	302
PTU_625	KF454518	Thymus marschallianus	89.04	301
PTU_625	KF454516	Thymus altaicus	89.04	301
PTU_626	KX167430	Misopates orontium	98.92	277
PTU_626	AY591288	Antirrhinum majus	93.21	265
PTU_626	KT031917	Albraunia foveopilosa	90.23	266
PTU_626	KT031891	Chaenorhinum spicatum	90.23	266
PTU_627	KX096056	Ocimum americanum	90.70	172
PTU_627	KF454238	Ocimum basilicum	91.52	165
PTU_627	378406718	Hyptis argyrophylla	87.02	131
PTU_627	378406721	Hyptis halimifolia	87.02	131
PTU_628	204306817	Corchorus olitorius	100.00	222
PTU_628	83282752	Corchorus olitorius	100.00	222
PTU_628	83282751	Corchorus capsularis	91.93	223
PTU_628	219881458	Corchorus pseudo-olitorius	91.52	224
PTU_629	KF454118	Origanum vulgare	97.67	301
PTU_629	KX166553	Thymus pulegioides	90.73	302
PTU_629	KF454518	Thymus marschallianus	90.70	301
PTU_629	KC295045	Thymus mongolicus	90.37	301
PTU_630	KX167455	Geranium rotundifolium	98.66	299
PTU_630	KX166784	Geranium pratense	93.98	299
PTU_630	KY624391	Geranium wilfordii	93.65	299
PTU_630	KX166786	Geranium columbinum	93.33	300

qseqid	sseqid	stitle	pident	length
PTU_631	KX165842	Linaria vulgaris	99.30	285
PTU_631	KT031859	Linaria genistifolia	98.95	285
PTU_631	KT031864	Linaria japonica	98.60	285
PTU_631	KT031866	Linaria michauxii	98.25	285
PTU_632	KF241280	Arnebia euchroma	97.59	291
PTU_632	KX166735	Tragopogon pratensis	96.91	291
PTU_632	317451650	Tragopogon porrifolius	98.10	210
PTU_632	317451649	Tragopogon porrifolius	98.10	210
PTU_633	KF241280	Arnebia euchroma	97.59	291
PTU_633	KX166735	Tragopogon pratensis	96.91	291
PTU_633	317451650	Tragopogon porrifolius	98.10	210
PTU_633	317451649	Tragopogon porrifolius	98.10	210
PTU_634	KX166238	Solanum nigrum	94.27	262
PTU_634	KF454049	Solanum nigrum	94.27	262
PTU_634	KY652843	Solanum nigrum	93.51	262
PTU_634	KF454093	Lycium ruthenicum	93.02	258
PTU_635	KX166200	Malva neglecta	100.00	296
PTU_635	KX166208	Malva neglecta	99.66	296
PTU_635	KX165952	Malva sylvestris	99.66	296
PTU_635	KX166471	Malva arborea	98.65	296
PTU_636	KY968830	Bidens alba	97.93	290
PTU_636	KY968833	Bidens pilosa	98.88	269
PTU_636	KX165434	Bidens cernua	92.13	267
PTU_636	18028503	Bidens cronquistii	98.54	206
PTU_637	KX166238	Solanum nigrum	91.63	263
PTU_637	KF454049	Solanum nigrum	91.63	263
PTU_637	KY652843	Solanum nigrum	91.25	263

qseqid	sseqid	stitle	pident	length
PTU_637	KX282258	Lycium shawii	90.35	259
PTU_638	KX166238	Solanum nigrum	92.02	263
PTU_638	KF454049	Solanum nigrum	92.02	263
PTU_638	KY652843	Solanum nigrum	91.63	263
PTU_638	316986388	Capsicum frutescens	95.35	215
PTU_639	KX165842	Linaria vulgaris	99.65	285
PTU_639	KT031859	Linaria genistifolia	99.30	285
PTU_639	KT031864	Linaria japonica	98.95	285
PTU_639	KT031866	Linaria michauxii	98.60	285
PTU_640	KF241280	Arnebia euchroma	98.28	290
PTU_640	KX166735	Tragopogon pratensis	97.59	290
PTU_640	KX282414	Scorzonera tortuosissima	90.75	281
PTU_640	KF454418	Scorzonera pseudodivaricata	90.65	278
PTU_641	KX166238	Solanum nigrum	93.00	257
PTU_641	KF454049	Solanum nigrum	93.00	257
PTU_641	KY652843	Solanum nigrum	92.61	257
PTU_641	KX346971	Solanum lyratum	90.62	256
PTU_642	KX096059	Ocimum basilicum	92.62	271
PTU_642	KF454238	Ocimum basilicum	90.61	277
PTU_642	KX096056	Ocimum americanum	90.58	276
PTU_642	KU203783	Dorcoceras hygrometricum	97.70	87
PTU_643	KX166238	Solanum nigrum	91.63	263
PTU_643	KF454049	Solanum nigrum	91.63	263
PTU_643	KY652843	Solanum nigrum	91.25	263
PTU_643	316986388	Capsicum frutescens	95.33	214
PTU_644	KX166238	Solanum nigrum	95.15	227
PTU_644	KF454049	Solanum nigrum	94.42	233

qseqid	sseqid	stitle	pident	length
PTU_644	KY652843	Solanum nigrum	94.71	227
PTU_644	AC215459	Solanum lycopersicum	91.95	236
PTU_645	KY021162	Trifolium resupinatum	100.00	282
PTU_645	KX165849	Trifolium fragiferum	98.58	282
PTU_645	KX165754	Trifolium ornithopodioides	96.11	283
PTU_645	KX165653	Trifolium medium	94.76	286
PTU_646	KX096059	Ocimum basilicum	90.74	270
PTU_646	KX096056	Ocimum americanum	89.09	275
PTU_646	KF454238	Ocimum basilicum	89.13	276
PTU_646	KU203783	Dorcoceras hygrometricum	95.56	90
PTU_647	KF454118	Origanum vulgare	92.72	302
PTU_647	KX166553	Thymus pulegioides	86.80	303
PTU_647	KF454518	Thymus marschallianus	86.80	303
PTU_647	KC295045	Thymus mongolicus	86.47	303
PTU_648	KF454297	Gypsophila altissima	92.03	276
PTU_648	KF454299	Gypsophila altissima	91.67	276
PTU_648	KX282174	Gypsophila capillaris	88.41	276
PTU_648	JF421553	Vaccaria hispanica	88.64	273
PTU_649	KX166586	Eryngium campestre	99.66	291
PTU_649	KX165875	Eryngium campestre	99.31	291
PTU_649	KX167950	Eryngium maritimum	97.83	277
PTU_649	KX166587	Eryngium maritimum	97.47	277
PTU_650	KF241280	Arnebia euchroma	97.94	291
PTU_650	KX166735	Tragopogon pratensis	97.25	291
PTU_650	KF454418	Scorzonera pseudodivaricata	90.68	279
PTU_650	317451650	Tragopogon porrifolius	98.10	210
PTU_651	KF241280	Arnebia euchroma	98.28	291

qseqid	sseqid	stitle	pident	length
PTU_651	KX166735	Tragopogon pratensis	97.59	291
PTU_651	317451650	Tragopogon porrifolius	99.52	210
PTU_651	317451649	Tragopogon porrifolius	99.52	210
PTU_652	KX166238	Solanum nigrum	92.78	263
PTU_652	KF454049	Solanum nigrum	92.78	263
PTU_652	KY652843	Solanum nigrum	92.02	263
PTU_652	KF454093	Lycium ruthenicum	91.51	259
PTU_653	193735350	Rosmarinus officinalis	99.53	211
PTU_653	KF454271	Lycopus europaeus	89.47	285
PTU_653	KX166755	Salvia pratensis	90.15	274
PTU_653	KX282392	Salvia lanigera	89.82	275
PTU_654	KX166238	Solanum nigrum	88.93	262
PTU_654	KF454049	Solanum nigrum	88.93	262
PTU_654	KY652843	Solanum nigrum	88.55	262
PTU_654	316986388	Capsicum frutescens	92.02	213
PTU_655	KX166238	Solanum nigrum	91.63	263
PTU_655	KF454049	Solanum nigrum	91.63	263
PTU_655	KY652843	Solanum nigrum	91.25	263
PTU_655	316986388	Capsicum frutescens	95.79	214
PTU_656	KX096056	Ocimum americanum	93.09	275
PTU_656	KX096059	Ocimum basilicum	92.70	274
PTU_656	KF454238	Ocimum basilicum	92.39	276
PTU_656	JF301483	Hyptis pulegioides	80.73	275
PTU_657	86197791	Scorzonera laciniata	99.02	205
PTU_657	86197790	Scorzonera cana	98.53	204
PTU_657	KF454420	Scorzonera mongolica	89.09	275
PTU_657	KF454416	Scorzonera parviflora	87.85	288

qseqid	sseqid	stitle	pident	length
PTU_658	KX166200	Malva neglecta	99.66	296
PTU_658	KX166208	Malva neglecta	99.32	296
PTU_658	KX165952	Malva sylvestris	99.32	296
PTU_658	KX166471	Malva arborea	98.31	296
PTU_659	KU350154	Arbutus canariensis	97.00	300
PTU_659	KX165505	Arctous alpina	89.40	302
PTU_659	KU350164	Comarostaphylis diversifolia	88.33	300
PTU_659	KF419121	Comarostaphylis arbutoides	88.40	293
PTU_660	KF454118	Origanum vulgare	92.72	302
PTU_660	KX166553	Thymus pulegioides	88.81	277
PTU_660	KF454518	Thymus marschallianus	88.81	277
PTU_660	KF454516	Thymus altaicus	88.45	277
PTU_661	KX165692	Veronica fruticans	88.74	222
PTU_661	KX166103	Veronica fruticans	88.29	222
PTU_661	KX165593	Veronica agrestis	87.27	220
PTU_661	LC027918	Veronica persica	86.82	220
PTU_662	KX096056	Ocimum americanum	91.24	274
PTU_662	KX096059	Ocimum basilicum	90.84	273
PTU_662	KF454238	Ocimum basilicum	90.18	275
PTU_662	KU203796	Damrongia orientalis	95.92	98
PTU_663	KX096056	Ocimum americanum	94.18	275
PTU_663	KX096059	Ocimum basilicum	94.16	274
PTU_663	KF454238	Ocimum basilicum	93.12	276
PTU_663	JF301483	Hyptis pulegioides	80.43	276
PTU_664	KX166238	Solanum nigrum	90.80	261
PTU_664	KF454049	Solanum nigrum	90.80	261
PTU_664	KY652843	Solanum nigrum	90.42	261

qseqid	sseqid	stitle	pident	length
PTU_664	KX282258	Lycium shawii	88.76	258
PTU_665	KX282228	Triticum monococcum	90.28	288
PTU_665	KF184927	Saccharum hybrid cultivar	90.18	285
PTU_665	KX282107	Dichanthium annulatum	89.82	285
PTU_665	KX689356	Chrysopogon argutus	89.82	285
PTU_666	KF241280	Arnebia euchroma	97.59	291
PTU_666	KX166735	Tragopogon pratensis	96.91	291
PTU_666	317451650	Tragopogon porrifolius	97.62	210
PTU_666	317451649	Tragopogon porrifolius	97.62	210
PTU_667	KX165593	Veronica agrestis	97.83	276
PTU_667	KX165590	Veronica agrestis	97.46	276
PTU_667	LC027918	Veronica persica	96.06	279
PTU_667	KT948624	Veronica persica	96.06	279
PTU_668	KX166397	Calystegia soldanella	90.48	294
PTU_668	LC085876	Calystegia hederacea	90.14	294
PTU_668	KX165837	Calystegia sepium	89.80	294
PTU_668	KF454079	Convolvulus arvensis	89.04	292
PTU_669	KF454118	Origanum vulgare	96.36	302
PTU_669	KX166553	Thymus pulegioides	90.76	303
PTU_669	KF454518	Thymus marschallianus	90.73	302
PTU_669	KF454516	Thymus altaicus	90.76	303
PTU_670	KX166238	Solanum nigrum	89.73	263
PTU_670	KF454049	Solanum nigrum	89.73	263
PTU_670	KY652843	Solanum nigrum	89.35	263
PTU_670	KX346971	Solanum lyratum	87.79	262
PTU_671	KX166238	Solanum nigrum	92.02	263
PTU_671	KF454049	Solanum nigrum	92.02	263

qseqid	sseqid	stitle	pident	length
PTU_671	KY652843	Solanum nigrum	91.63	263
PTU_671	KX282258	Lycium shawii	91.89	259
PTU_672	KX167057	Calamagrostis epigeios	97.87	282
PTU_672	KX165555	Ammophila arenaria	96.45	282
PTU_672	KX167939	Calamagrostis purpurea	95.39	282
PTU_672	KX167952	Calamagrostis purpurea	95.39	282
PTU_673	184186926	Spinacia oleracea	99.09	220
PTU_673	3132699	Spinacia oleracea	99.07	214
PTU_673	KT948637	Chenopodium sp. JD-2016	89.32	281
PTU_673	KX166014	Chenopodium ficifolium	88.21	280
PTU_674	KF454049	Solanum nigrum	92.34	261
PTU_674	KX166238	Solanum nigrum	91.57	261
PTU_674	KY652843	Solanum nigrum	91.19	261
PTU_674	316986388	Capsicum frutescens	94.88	215
PTU_675	KX096059	Ocimum basilicum	88.89	270
PTU_675	KF454238	Ocimum basilicum	88.04	276
PTU_675	KX096056	Ocimum americanum	87.64	275
PTU_675	JF301483	Hyptis pulegioides	96.70	91
PTU_676	JN187957	Atriplex sp. Yeelirrie Station	93.86	293
PTU_676	KX165622	Atriplex littoralis	93.52	293
PTU_676	KX165621	Atriplex littoralis	93.52	293
PTU_676	JN187961	Atriplex sp. Yeelirrie Station	93.17	293
PTU_677	KX166505	Crepis foetida	98.98	295
PTU_677	KX166910	Crepis paludosa	92.54	295
PTU_677	KX166909	Crepis paludosa	92.20	295
PTU_677	KX166259	Crepis mollis	89.83	295
PTU_678	KF241280	Arnebia euchroma	98.28	291

qseqid	sseqid	stitle	pident	length
PTU_678	KX166735	Tragopogon pratensis	97.59	291
PTU_678	317451650	Tragopogon porrifolius	99.52	209
PTU_678	317451649	Tragopogon porrifolius	99.52	209
PTU_679	KX282181	Haloxylon salicornicum	91.70	277
PTU_679	KX282180	Haloxylon salicornicum	91.70	277
PTU_679	KX282182	Haloxylon salicornicum	91.34	277
PTU_679	KX282091	Cornulaca monacantha	90.61	277
PTU_680	110589867	Bromus ayacuchensis	93.68	190
PTU_680	38566408	Bromus segetum	93.68	190
PTU_680	38566421	Bromus berteroanus	93.23	192
PTU_680	38566422	Bromus gunckelii	93.23	192
PTU_681	KU350154	Arbutus canariensis	95.70	302
PTU_681	KX165505	Arctous alpina	88.12	303
PTU_681	KU350164	Comarostaphylis diversifolia	87.42	302
PTU_681	KF419121	Comarostaphylis arbutoides	87.80	295

Session Info

```
sessionInfo()
```

```
## R version 3.4.3 (2017-11-30)
## Platform: x86_64-apple-darwin17.3.0 (64-bit)
## Running under: macOS High Sierra 10.13.3
##
## Matrix products: default
## BLAS: /System/Library/Frameworks/Accelerate.framework/Versions/A/Frameworks/v
## LAPACK: /System/Library/Frameworks/Accelerate.framework/Versions/A/Frameworks
##
## locale:
## [1] C
##
```

```
## attached base packages:
## [1] stats     graphics  grDevices utils     datasets  methods   base
##
## other attached packages:
##  [1] bindrcpp_0.2       viridis_0.4.1      viridisLite_0.3.0
##  [4] wordcloud_2.5      RColorBrewer_1.1-2 forcats_0.2.0
##  [7] dplyr_0.7.4        purrr_0.2.4        readr_1.1.1
## [10] tidyr_0.8.0        tibble_1.4.2       tidyverse_1.2.1
## [13] stringr_1.3.0      knitr_1.20         ggplot2_2.2.1
##
## loaded via a namespace (and not attached):
##  [1] tidyselect_0.2.3 slam_0.1-42      reshape2_1.4.3   haven_1.1.1
##  [5] lattice_0.20-35  colorspace_1.3-2 htmltools_0.3.6  yaml_2.1.18
##  [9] rlang_0.2.0      pillar_1.1.0     foreign_0.8-69   glue_1.2.0
## [13] modelr_0.1.1     readxl_1.0.0     bindr_0.1        plyr_1.8.4
## [17] munsell_0.4.3    gtable_0.2.0     cellranger_1.1.0 rvest_0.3.2
## [21] psych_1.7.8      evaluate_0.10.1  labeling_0.3     parallel_3.4.3
## [25] highr_0.6        broom_0.4.3      Rcpp_0.12.15     scales_0.5.0
## [29] backports_1.1.2  jsonlite_1.5     gridExtra_2.3    mnormt_1.5-5
## [33] hms_0.4.1        digest_0.6.15    stringi_1.1.7    grid_3.4.3
## [37] rprojroot_1.3-2  cli_1.0.0        tools_3.4.3      magrittr_1.5
## [41] lazyeval_0.2.1   crayon_1.3.4     pkgconfig_2.0.1  xml2_1.1.1
## [45] lubridate_1.7.1  assertthat_0.2.0 rmarkdown_1.9    httr_1.3.1
## [49] rstudioapi_0.7   R6_2.2.2         nlme_3.1-131     compiler_3.4.3
```
