# Supplementary material for: DNA Metabarcoding for Quality Control of Basil, Oregano, and Paprika
Source: Front Plant Sci. 2021 Jun 4;12:665618. doi: 10.3389/fpls.2021.665618 (PMC8213367; doi:10.3389/fpls.2021.665618)
Supplement: Supplementary File 4 — DNA metabarcoding results for product identification. [file Data_Sheet_4.PDF]

S 4.1 DNA metabarcoding based identification of species products labeled as containing paprika

Sample ID	Capsicum annuum	Wind pollinated species	Wind spread species	Other ingredients
**KR18-03**	1	1	0	1
**KR18-04**	1	0	0	0
**KR18-07**	0	1	0	0
**KR18-13**	1	1	0	0
**KR18-17**	1	0	0	0
**KR18-19**	1	0	0	0
**KR18-23**	0	1	0	0
**KR18-26**	1	1	0	0
**KR18-28**	1	0	1	0
**KR18-34**	1	0	1	1
**KR18-38**	1	0	0	1
**KR18-45**	1	0	0	0
**KR18-58**	0	0	1	0

S 4.2 DNA metabarcoding based identification of species products labeled as containing oregano

Sample ID	Origanum vulgare	Wind pollinated species	Wind spread species	Other ingredients
**KR18-01**	0	0	0	1
**KR18-05**	1	0	1	1
**KR18-06**	1	0	1	1
**KR18-10**	1	0	1	1
**KR18-12**	1	0	1	1
**KR18-14**	1	0	0	1
**KR18-15**	1	0	0	0
**KR18-21**	1	1	0	1
**KR18-24**	1	0	0	1
**KR18-27**	1	0	1	1
**KR18-29**	1	0	1	0
**KR18-31**	1	0	0	1
**KR18-35^**	1	1	0	1
**KR18-36**	1	1	1	0
**KR18-41**	1	1	1	1
**KR18-42**	1	0	1	0
**KR18-44**	1	1	1	1
**KR18-47**	1	0	0	1
**KR18-48**	1	1	0	1
**KR18-50**	1	1	1	1
**KR18-55**	1	0	1	0
**KR18-57**	1	1	1	1
**KR18-61**	1	1	1	1
**KR18-62**	1	1	1	1

^ includes both basilikum and oregano according to the produkt label.

S 4.3 DNA metabarcoding based identification of species products labeled as containing basil

Sample ID	Ocimum basilicum$	Wind pollinated species	Wind spread species	Other ingredients
**KR18-02**	1	1	0	1
**KR18-08**	1	1	0	1
**KR18-11**	1	1	0	1
**KR18-18**	1	1	0	1
**KR18-20**	1	1	0	1
**KR18-22**	1	0	0	1
**KR18-25**	1	1	0	1
**KR18-30**	1	1	0	1
**KR18-32**	0	1	0	1
**KR18-35^**	1	1	0	1
**KR18-37**	1	1	0	1
**KR18-39**	1	0	0	1
**KR18-40**	1	0	0	1
**KR18-43**	1	1	0	1
**KR18-49**	1	1	0	1
**KR18-51**	0	1	1	1
**KR18-56**	1	0	0	1
**KR18-59**	1	1	0	1

$ Ocimum basilicum can not be discriminated by Ocimum americanum by using ITS2. It is unlikely that products contain Ocimum americanum.

^ Both basil and oregano are listed on the product label.
